# Supplementary material for: Firing rate adaptation affords place cell theta sweeps, phase precession, and procession
Source: eLife. 2024 Jul 22;12:RP87055. doi: 10.7554/eLife.87055 (PMC11262797; doi:10.7554/eLife.87055)
Supplement: Source code 1. [file elife-87055-code1.zip › code/Code_instruction.pdf]

北京大学
PEKING UNIVERSITY

Code instruction

The provided codes are written in Matlab and Python.

Here are some instructions for running the code:

(1) The names of the code files are self-explained, as to produce the figures in the main text. All the figures generated by the provided codes are also included in the supplementary information.

(2) To run Matlab code, users need to install the "signal processing toolbox" and ensure that the version of matlab is above Matlab2018.

(3) To run Python code, users need to install numpy and matplotlib packages. The python3.9 environment is recommended.
