# Supplementary figures and images for: Firing rate adaptation affords place cell theta sweeps, phase precession, and procession (part 2 of 2)
Source: eLife. 2024 Jul 22;12:RP87055. doi: 10.7554/eLife.87055 (PMC11262797; doi:10.7554/eLife.87055)

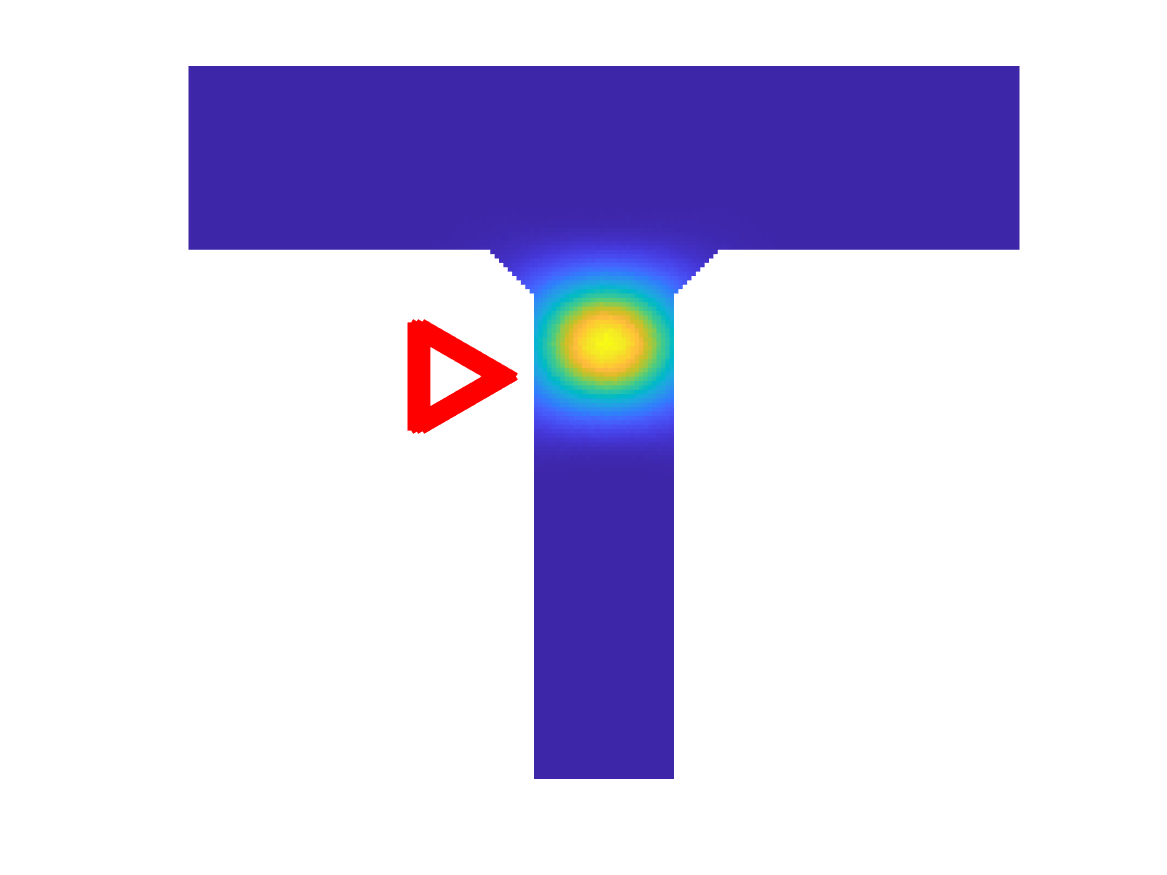

Supplement: Source code 1. [file elife-87055-code1.zip › code/fig5b_frames/85.bmp]

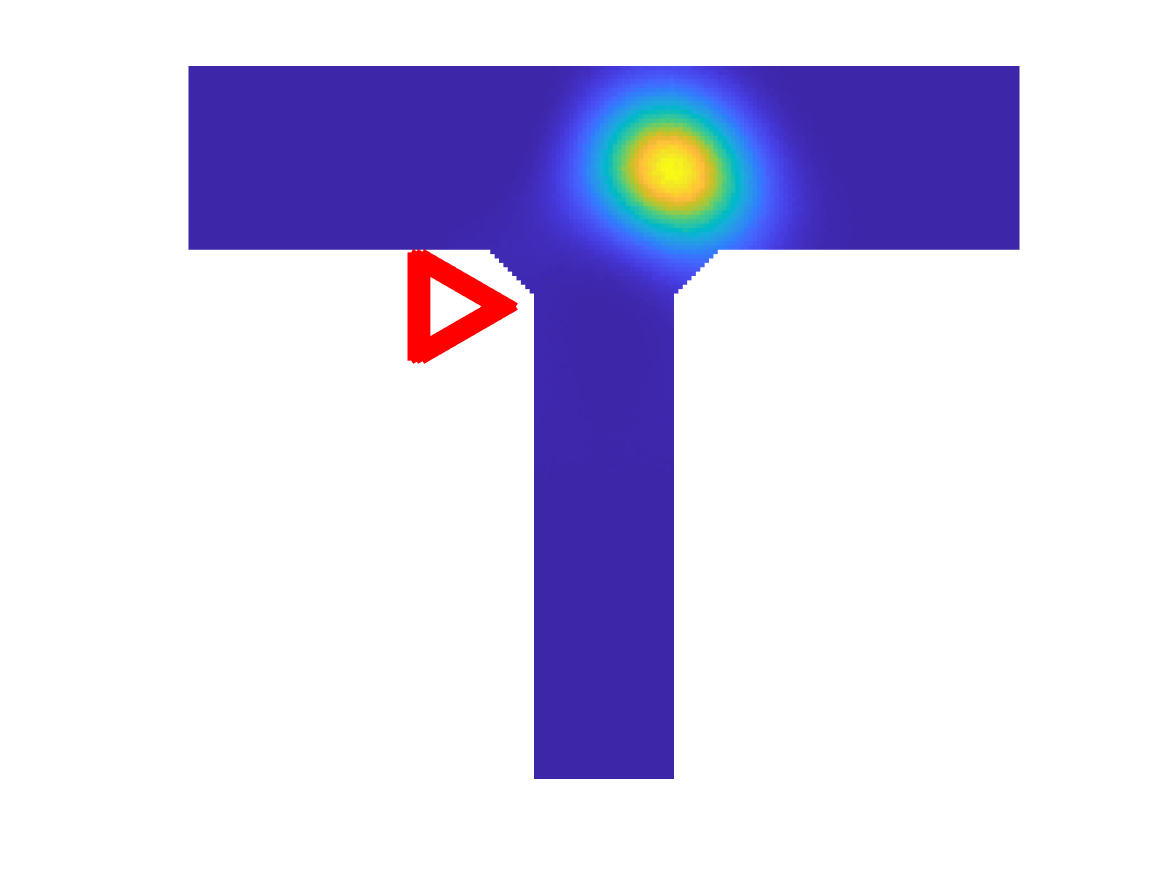

Supplement: Source code 1. [file elife-87055-code1.zip › code/fig5b_frames/190.bmp]

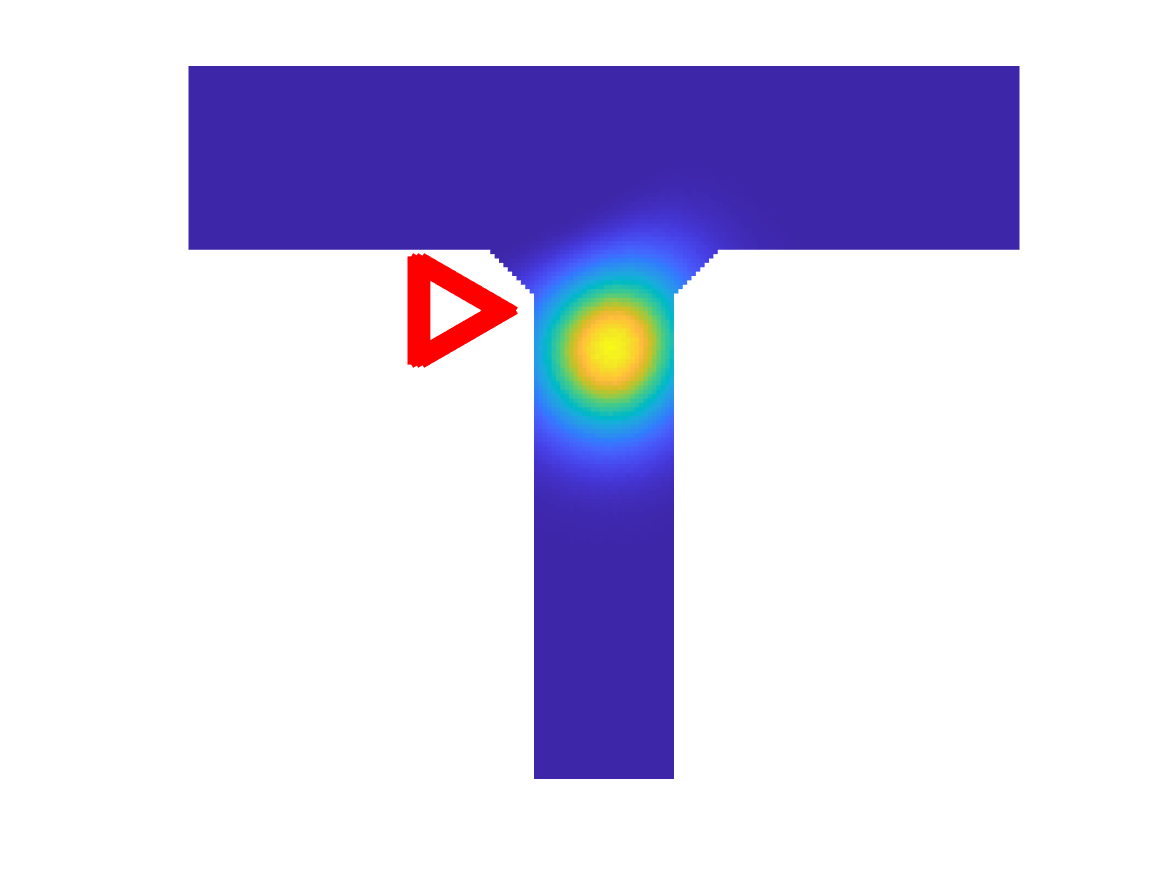

Supplement: Source code 1. [file elife-87055-code1.zip › code/fig5b_frames/184.bmp]

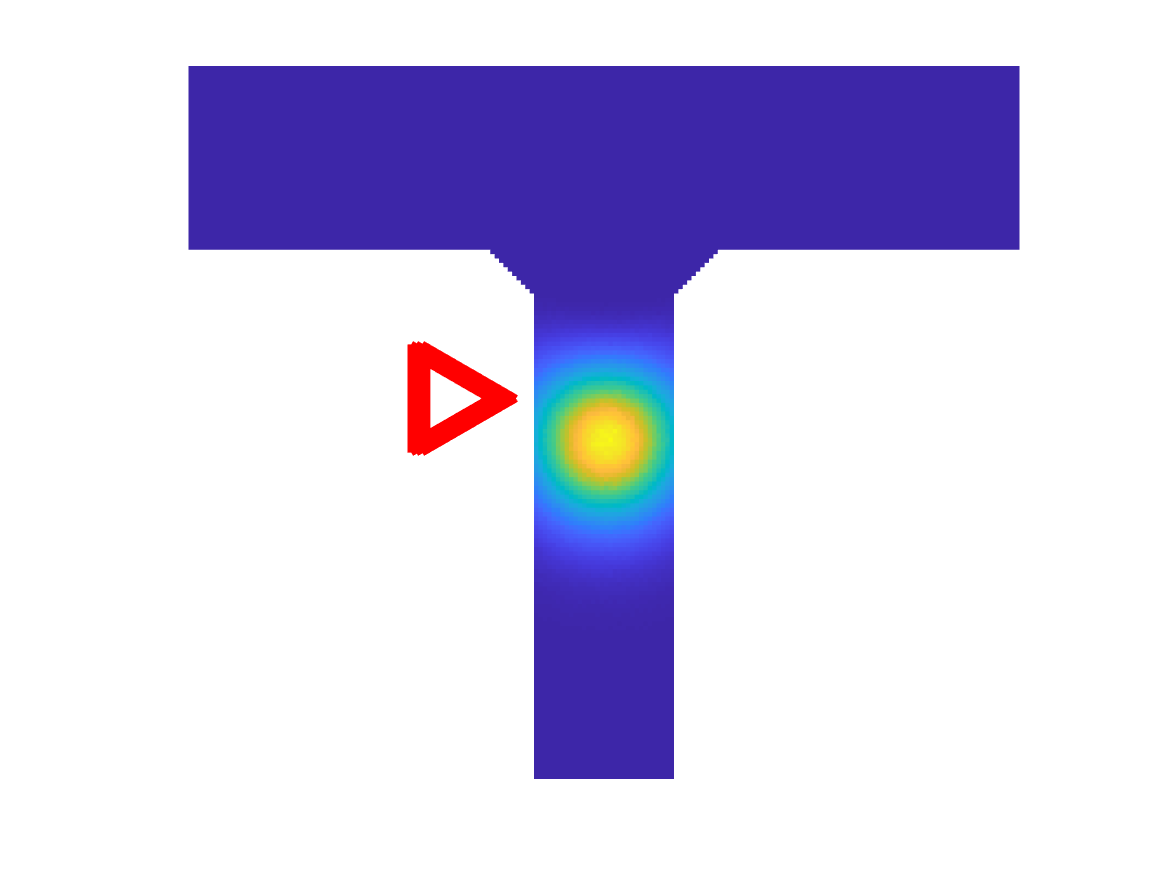

Supplement: Source code 1. [file elife-87055-code1.zip › code/fig5b_frames/52.bmp]

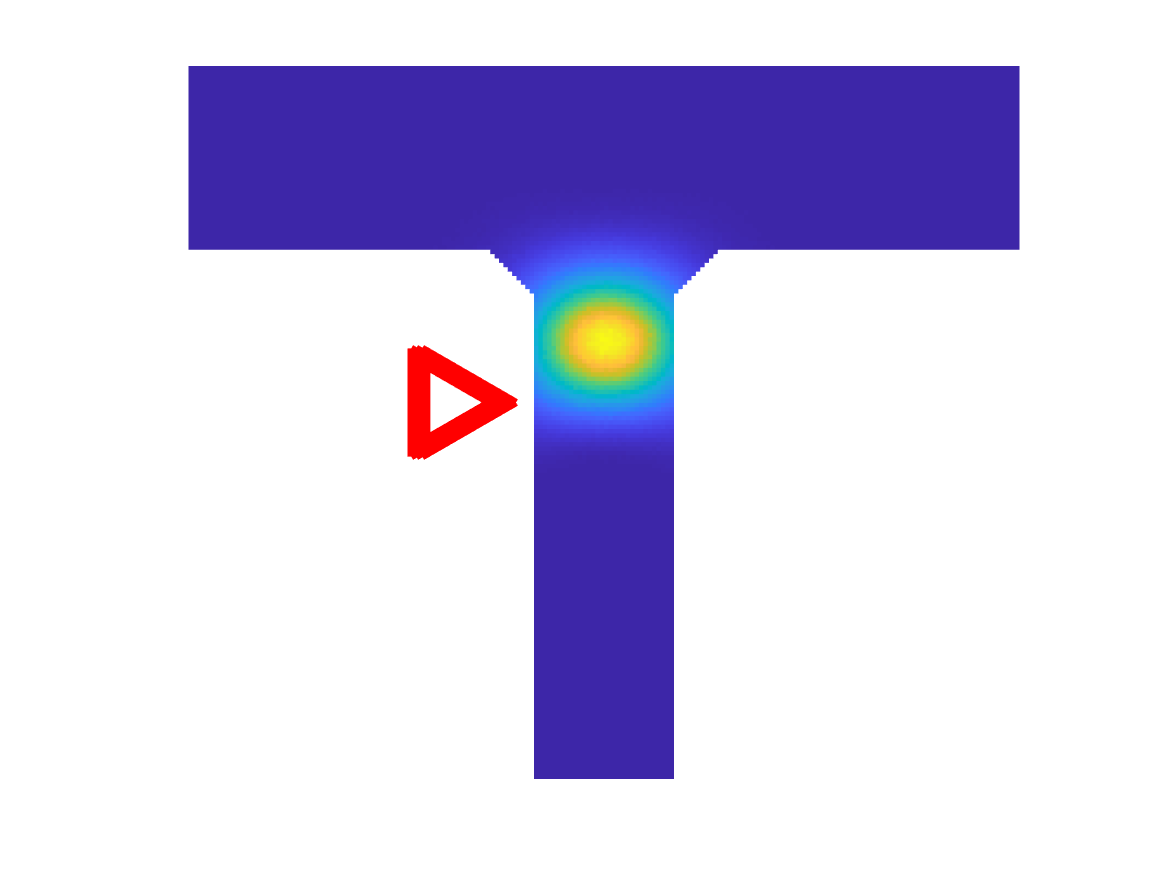

Supplement: Source code 1. [file elife-87055-code1.zip › code/fig5b_frames/46.bmp]

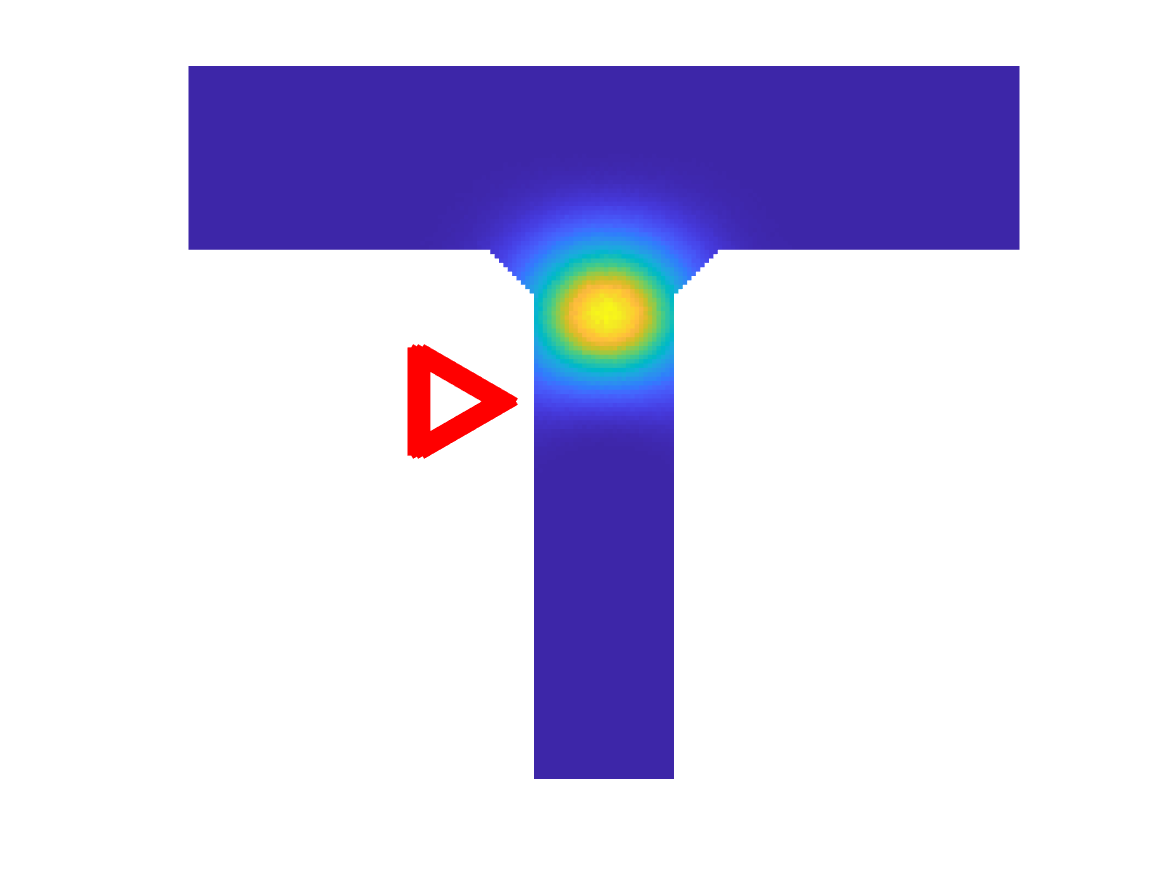

Supplement: Source code 1. [file elife-87055-code1.zip › code/fig5b_frames/47.bmp]

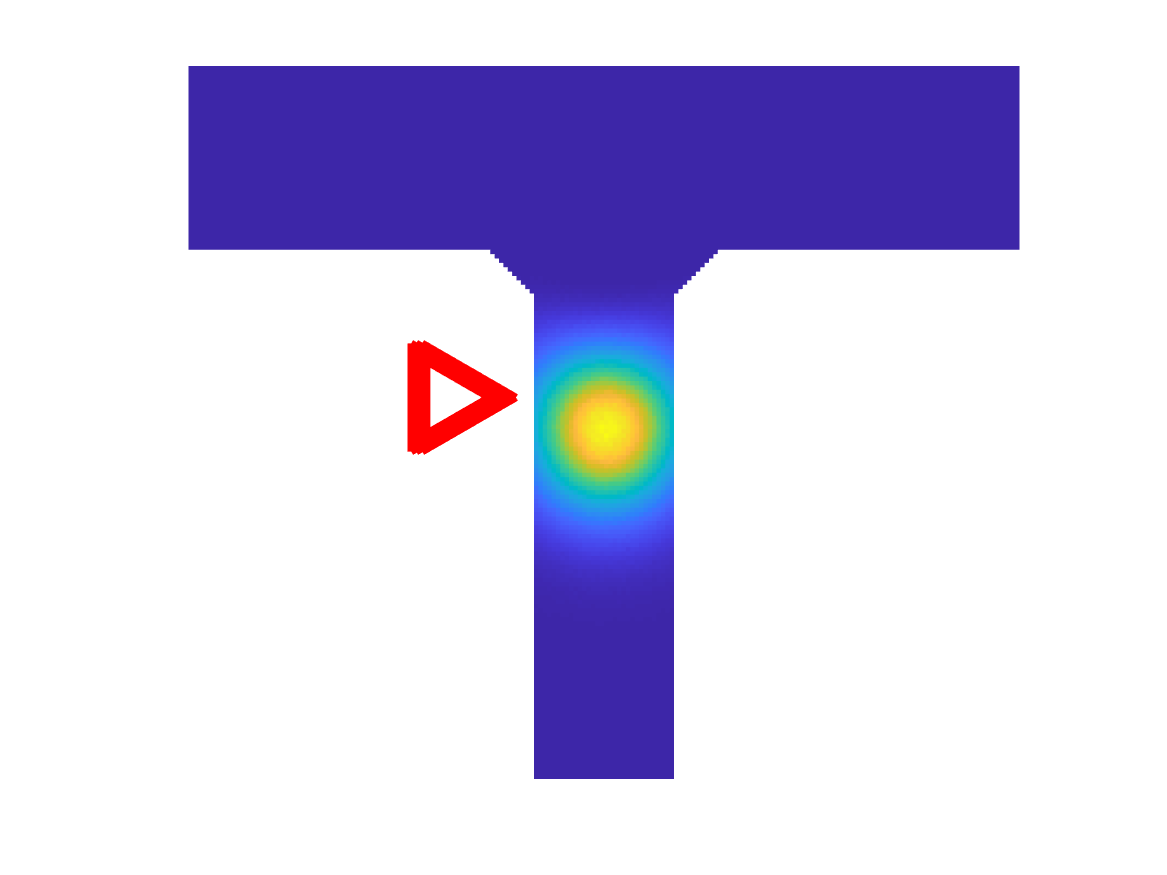

Supplement: Source code 1. [file elife-87055-code1.zip › code/fig5b_frames/53.bmp]

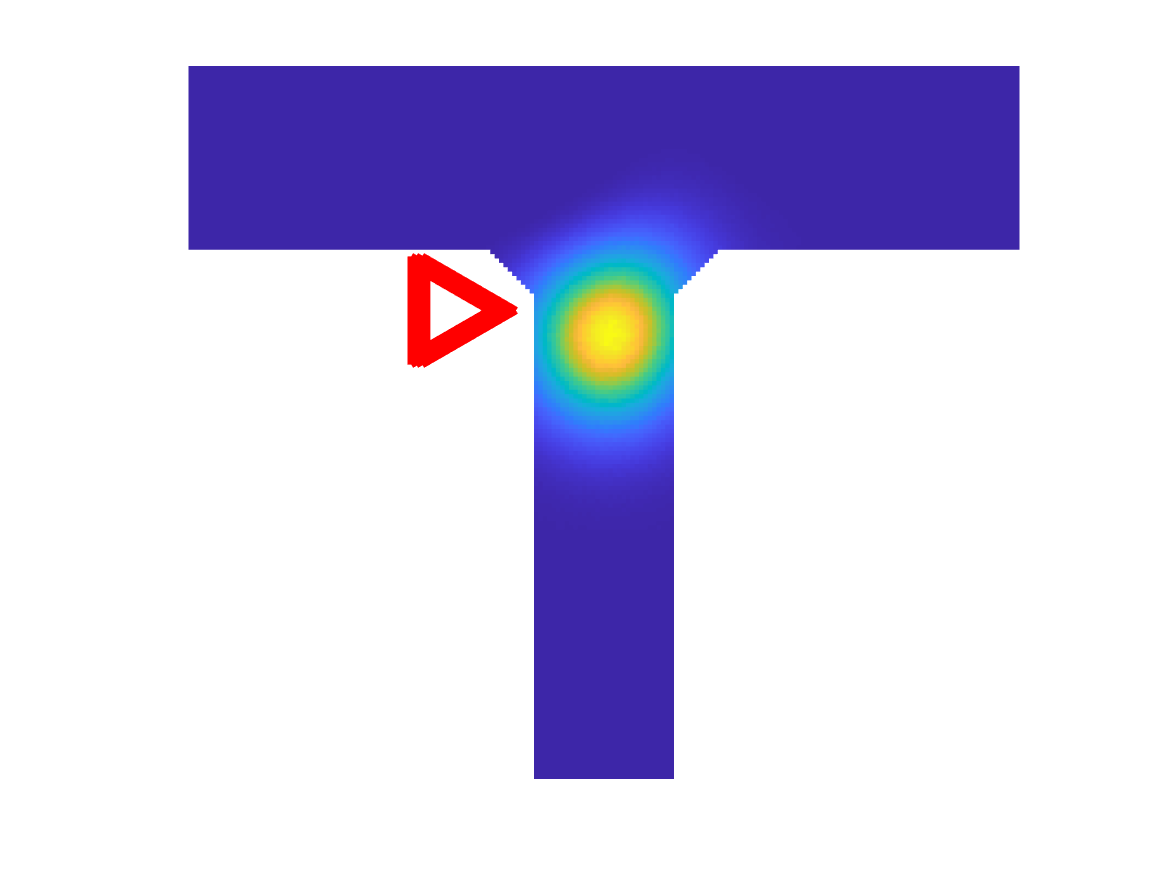

Supplement: Source code 1. [file elife-87055-code1.zip › code/fig5b_frames/185.bmp]

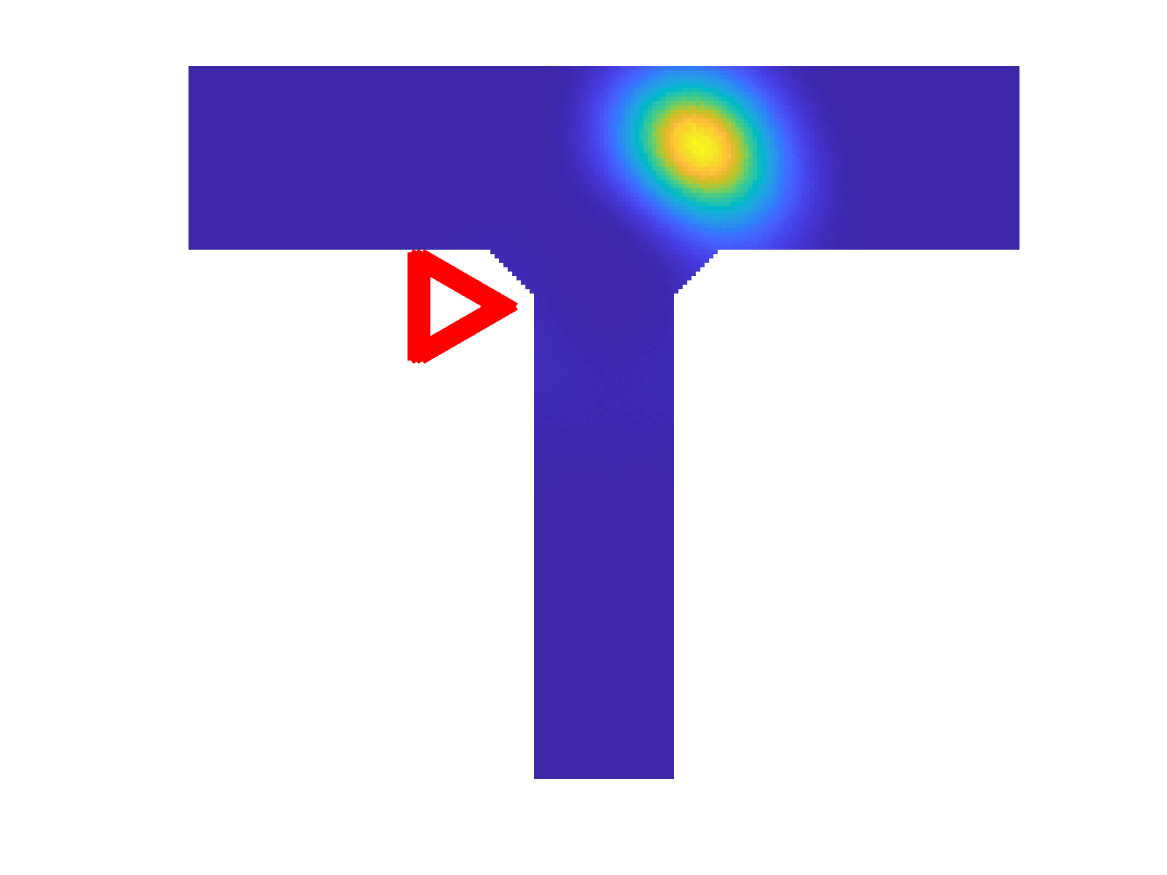

Supplement: Source code 1. [file elife-87055-code1.zip › code/fig5b_frames/191.bmp]

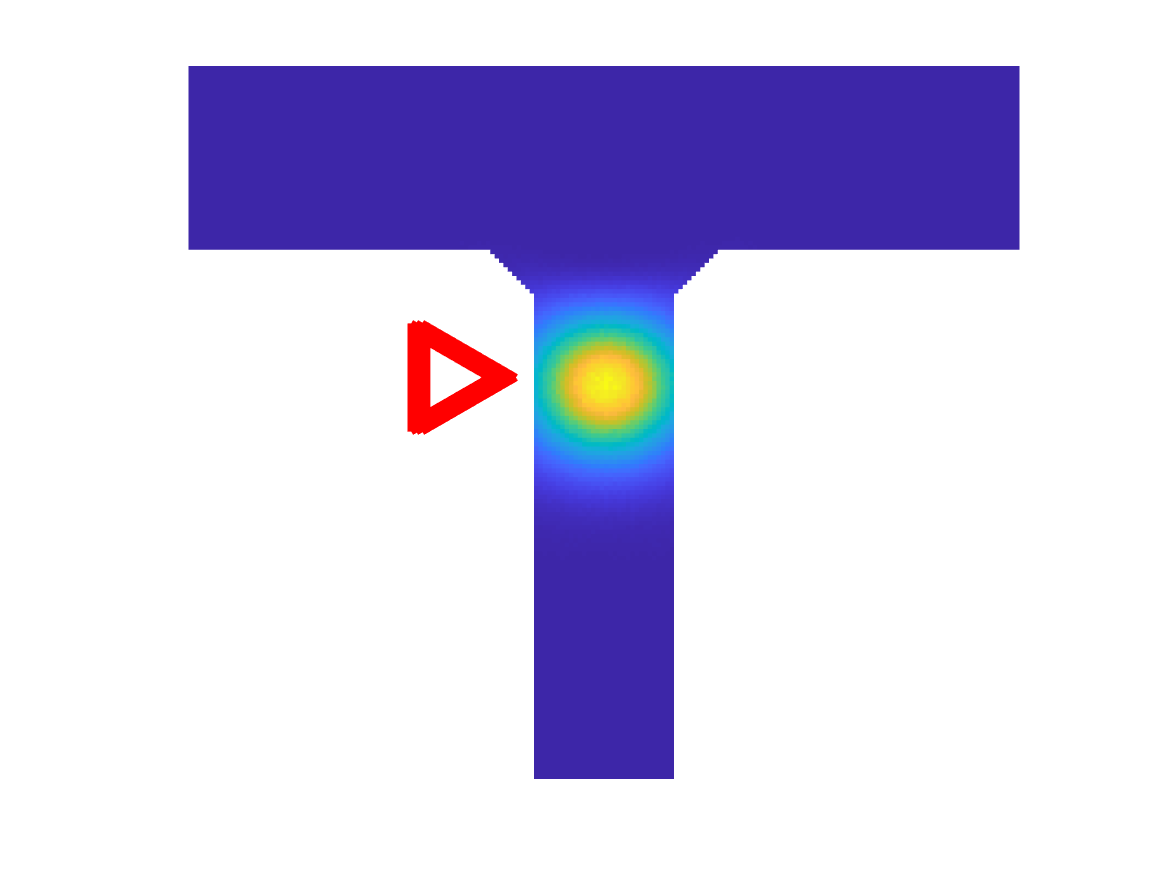

Supplement: Source code 1. [file elife-87055-code1.zip › code/fig5b_frames/84.bmp]

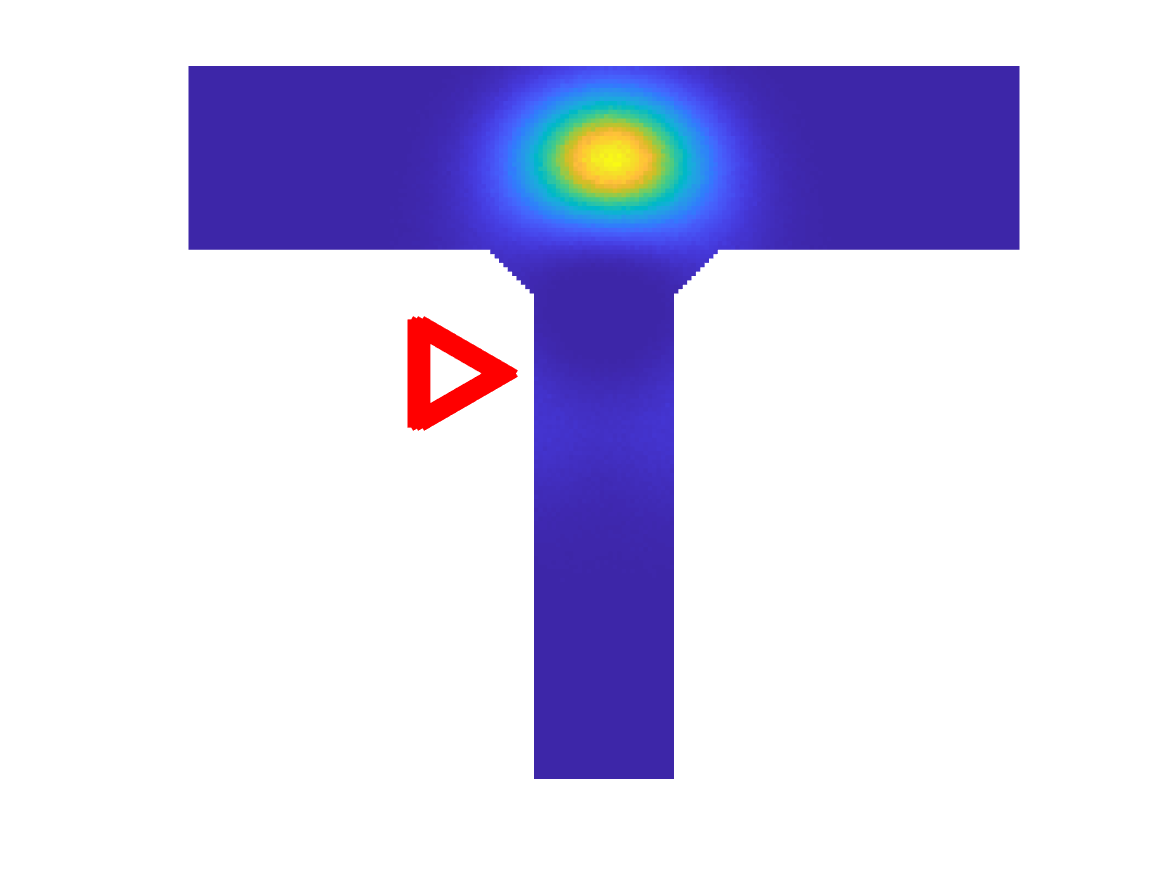

Supplement: Source code 1. [file elife-87055-code1.zip › code/fig5b_frames/90.bmp]

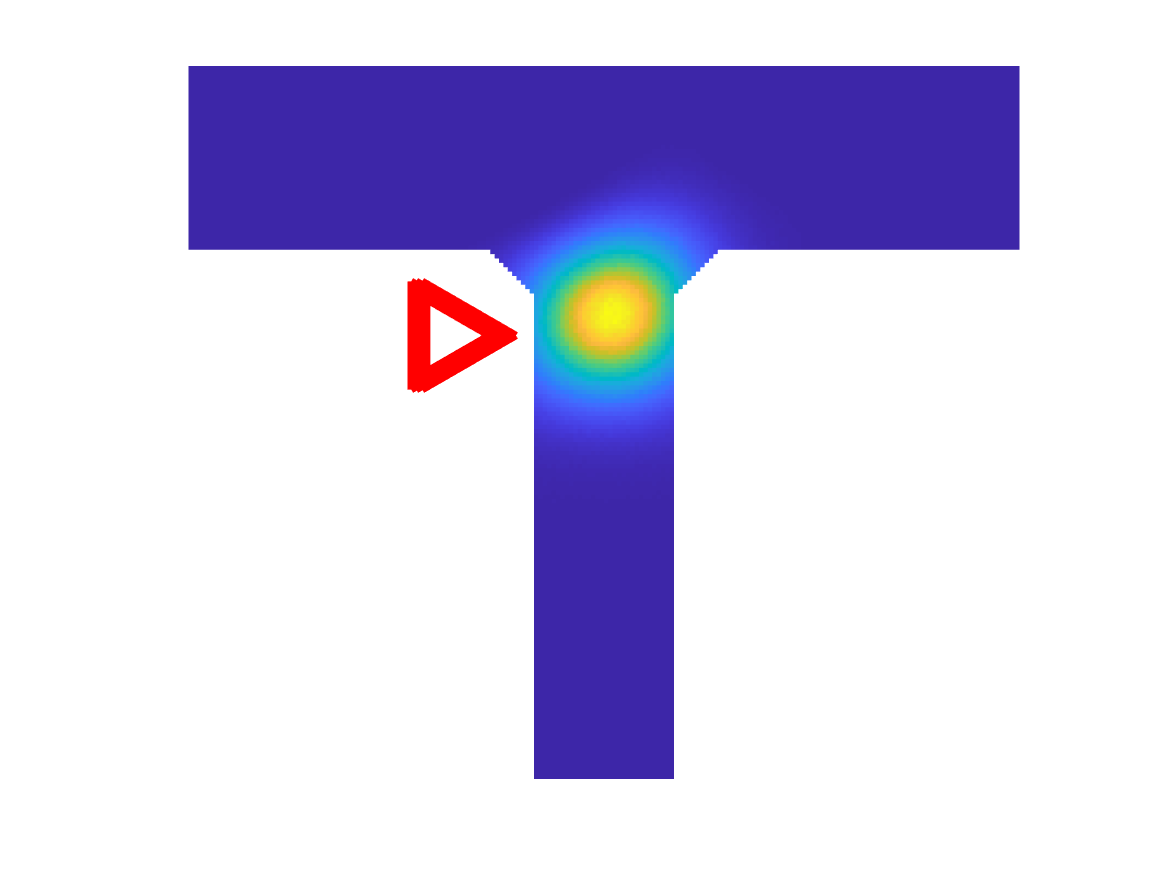

Supplement: Source code 1. [file elife-87055-code1.zip › code/fig5b_frames/146.bmp]

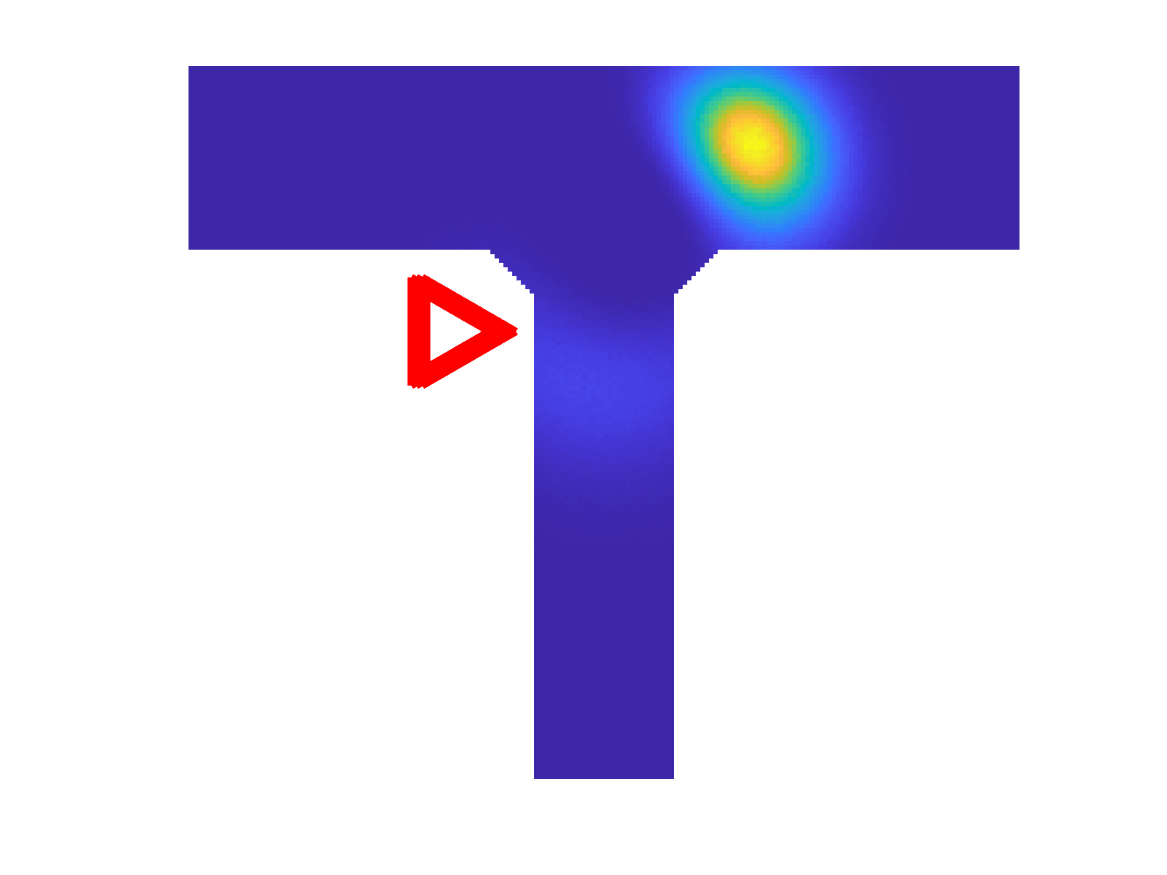

Supplement: Source code 1. [file elife-87055-code1.zip › code/fig5b_frames/152.bmp]

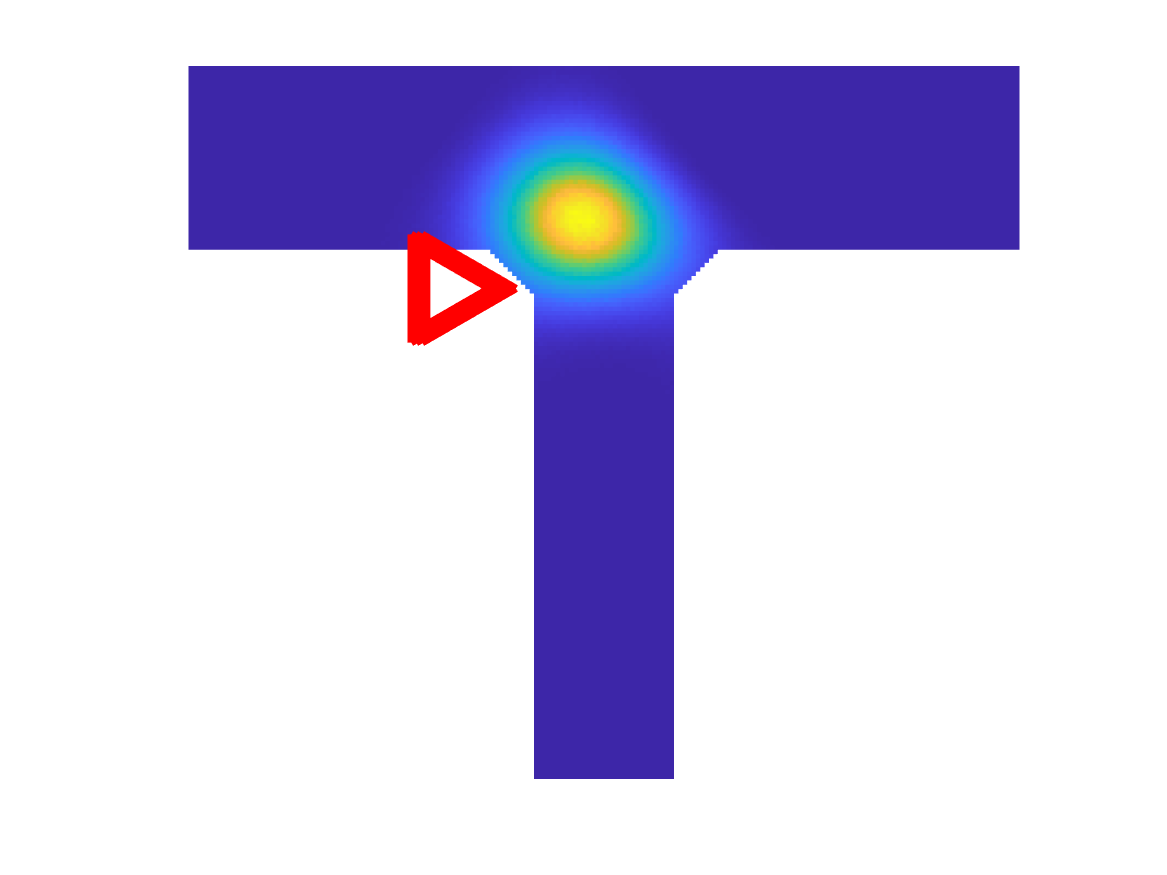

Supplement: Source code 1. [file elife-87055-code1.zip › code/fig5b_frames/218.bmp]

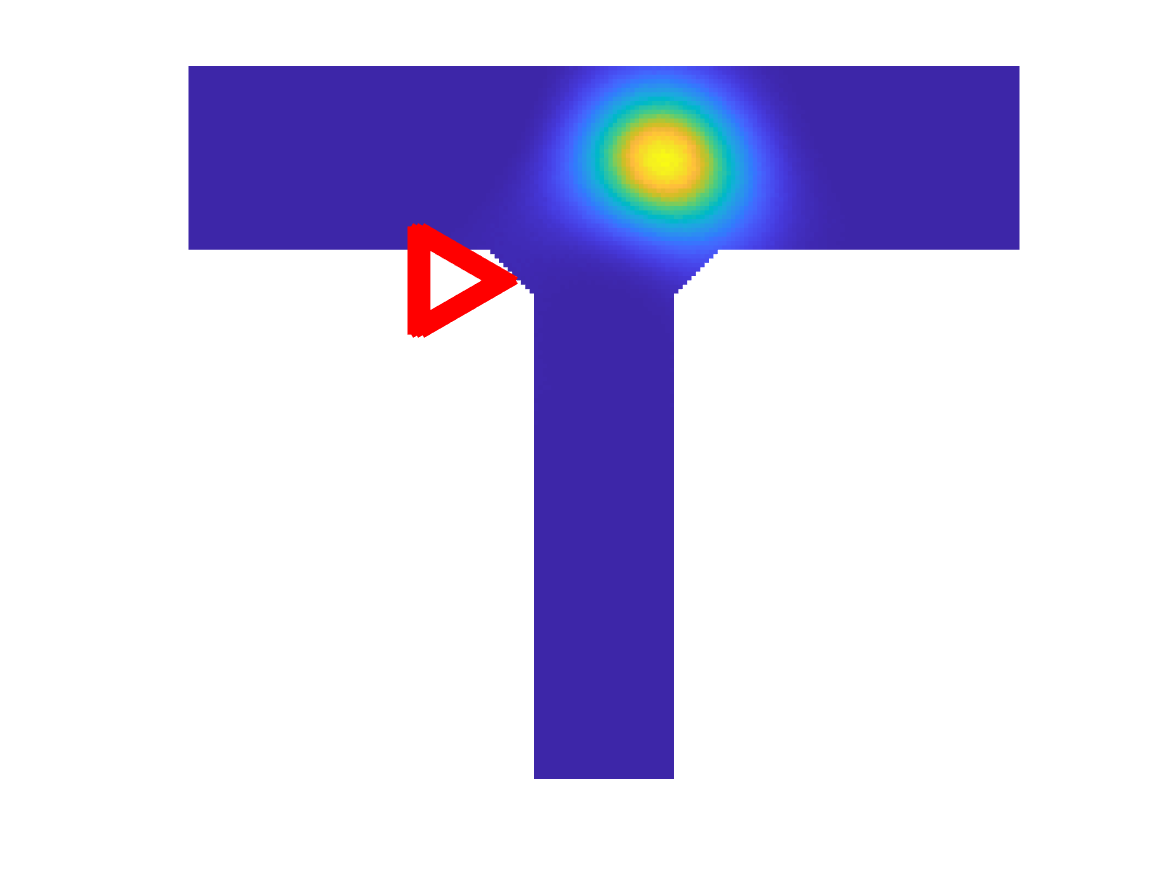

Supplement: Source code 1. [file elife-87055-code1.zip › code/fig5b_frames/230.bmp]

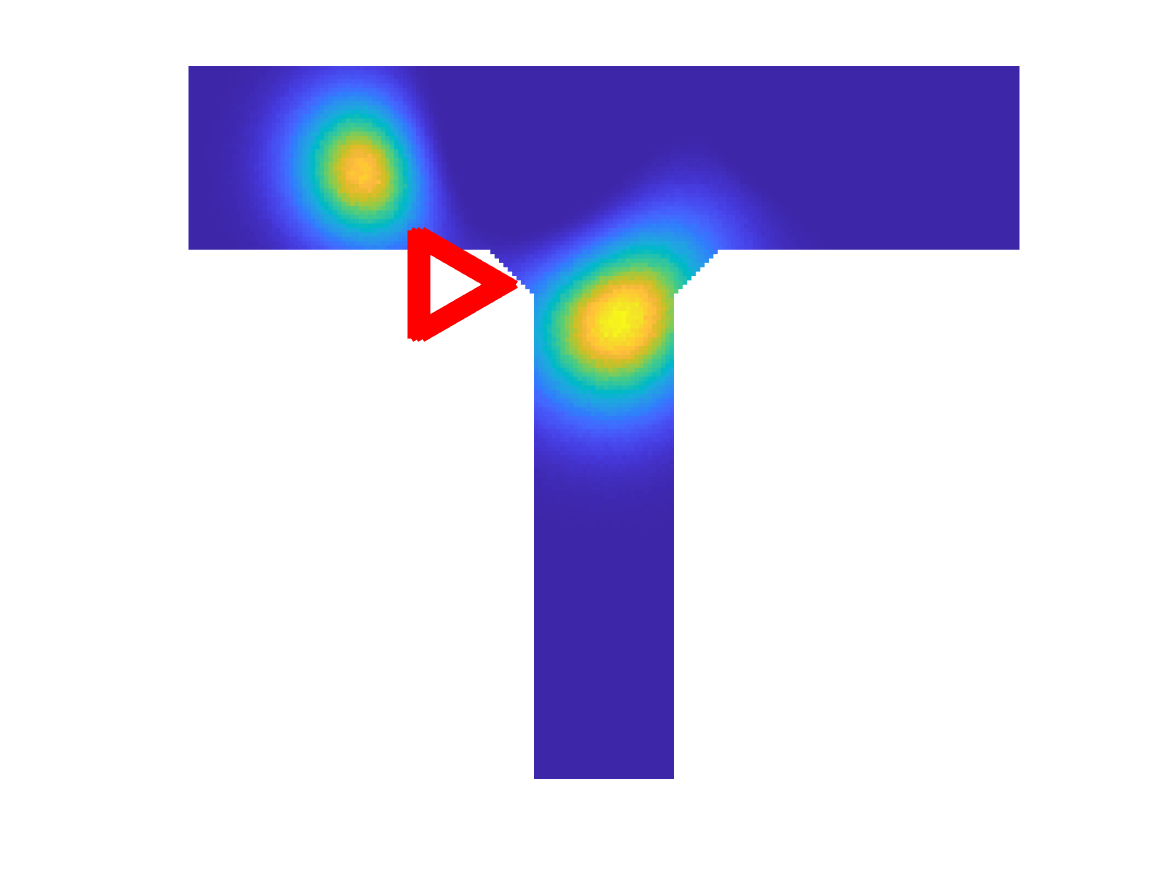

Supplement: Source code 1. [file elife-87055-code1.zip › code/fig5b_frames/224.bmp]

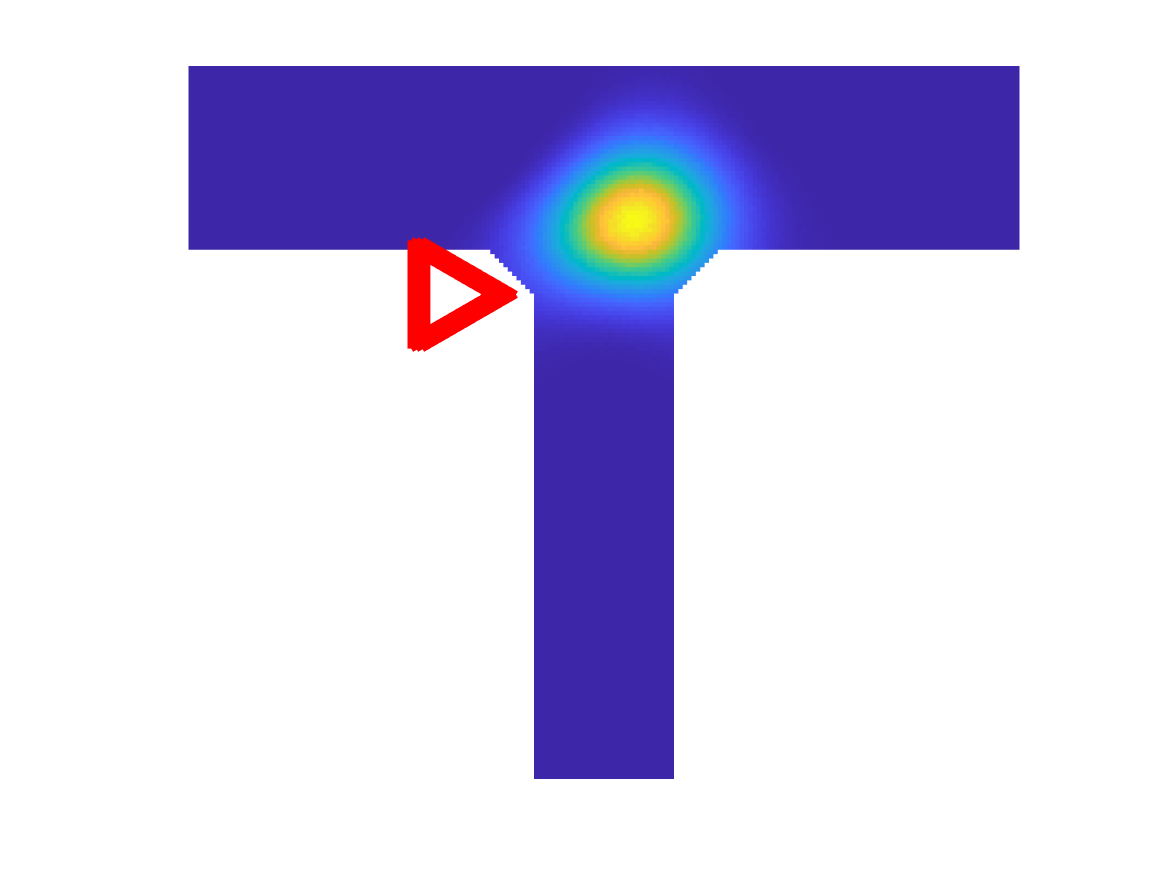

Supplement: Source code 1. [file elife-87055-code1.zip › code/fig5b_frames/208.bmp]

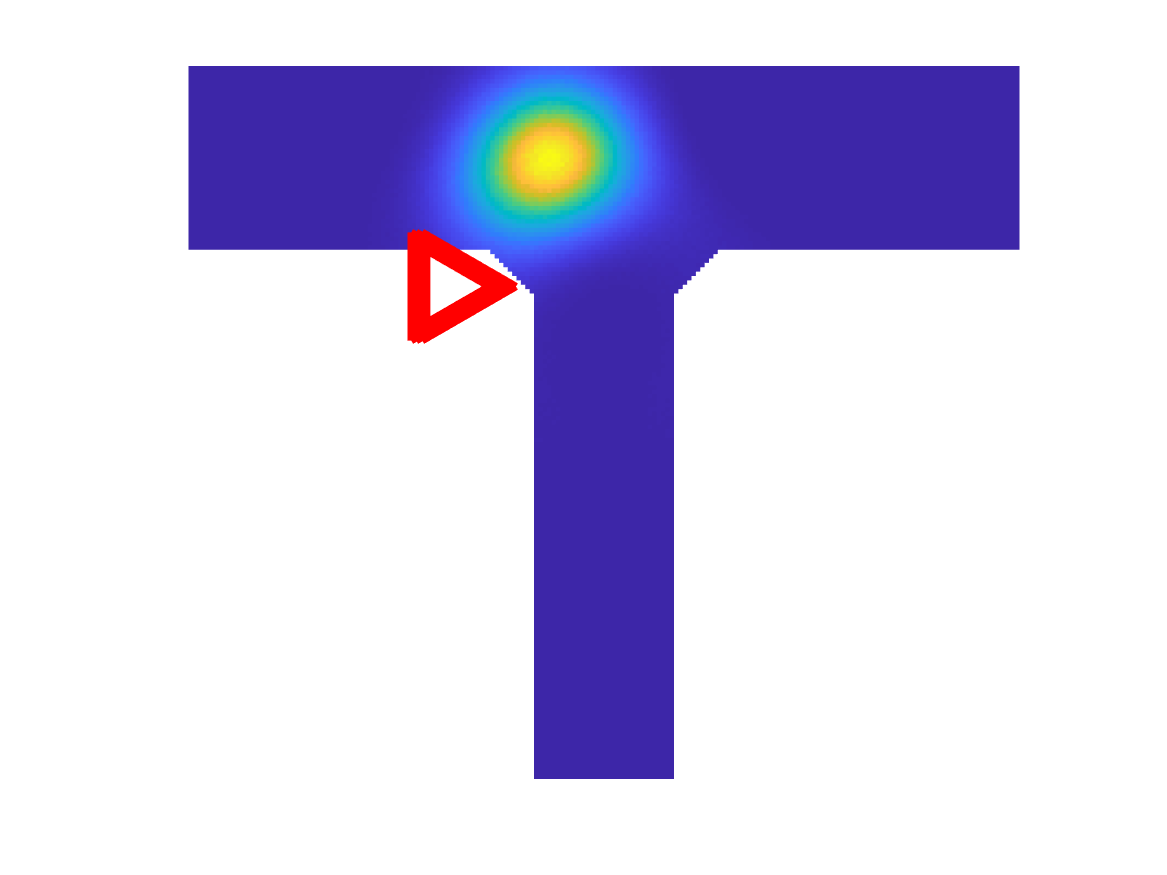

Supplement: Source code 1. [file elife-87055-code1.zip › code/fig5b_frames/220.bmp]

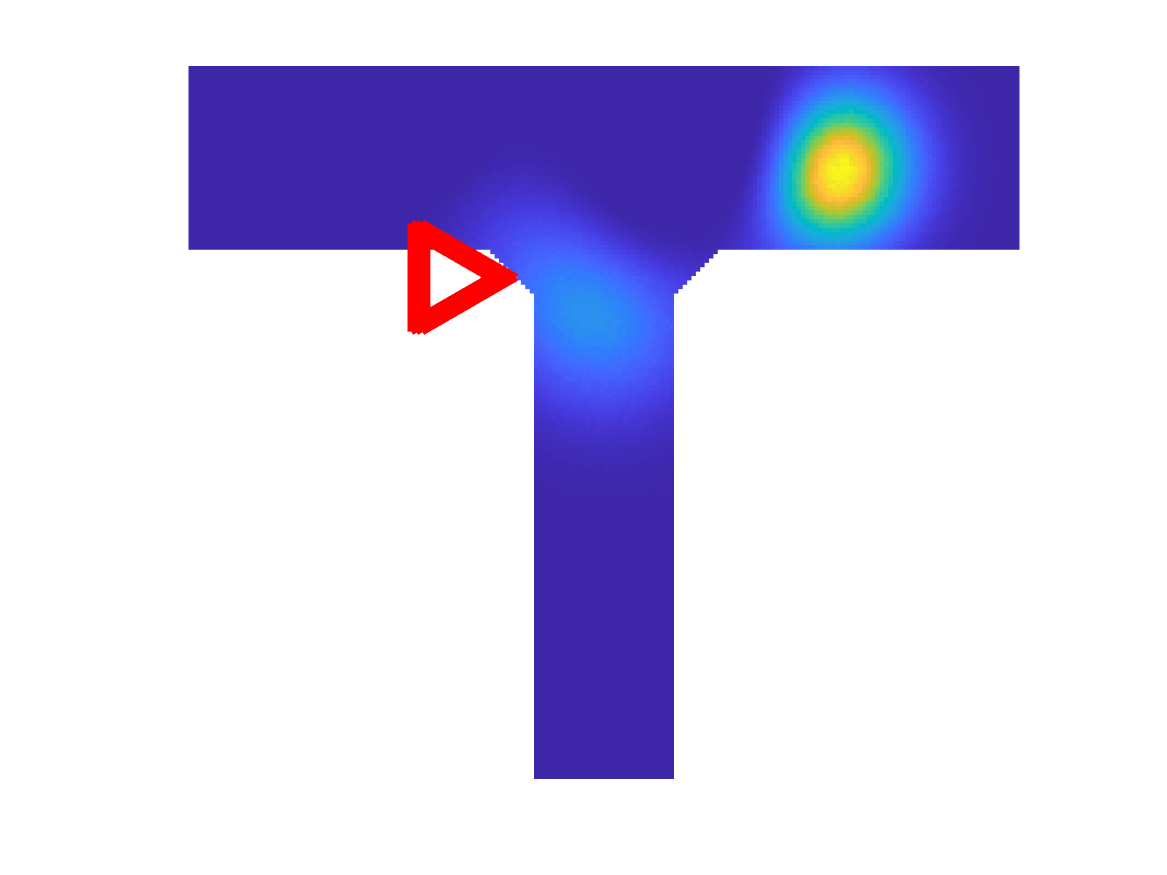

Supplement: Source code 1. [file elife-87055-code1.zip › code/fig5b_frames/234.bmp]

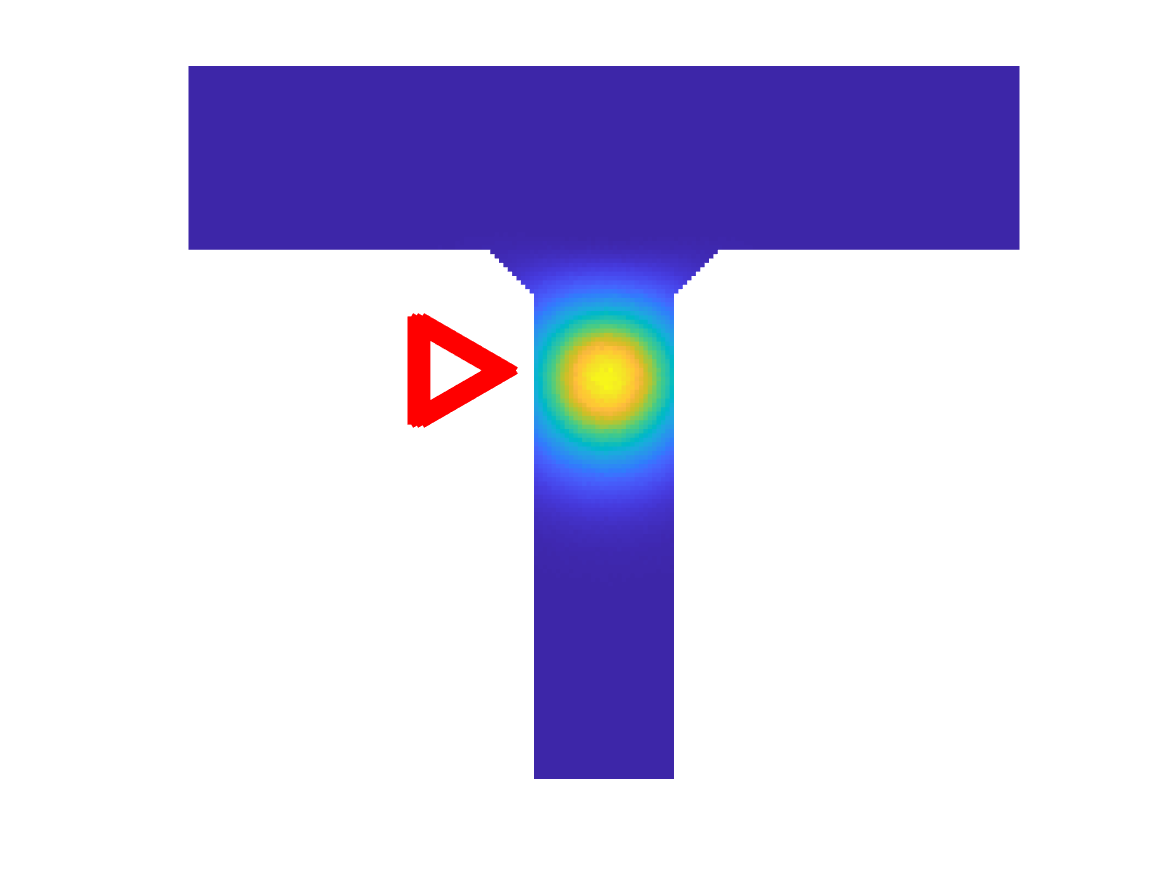

Supplement: Source code 1. [file elife-87055-code1.zip › code/fig5b_frames/94.bmp]

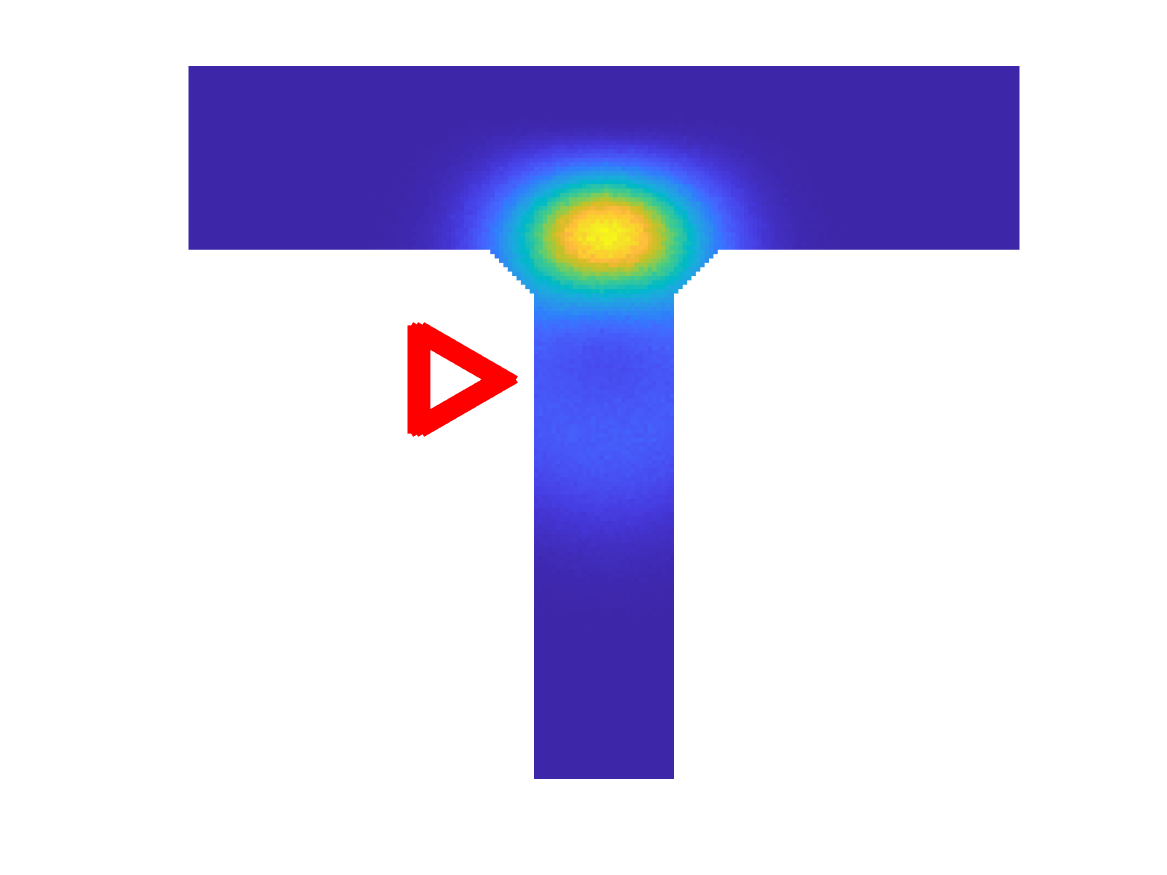

Supplement: Source code 1. [file elife-87055-code1.zip › code/fig5b_frames/80.bmp]

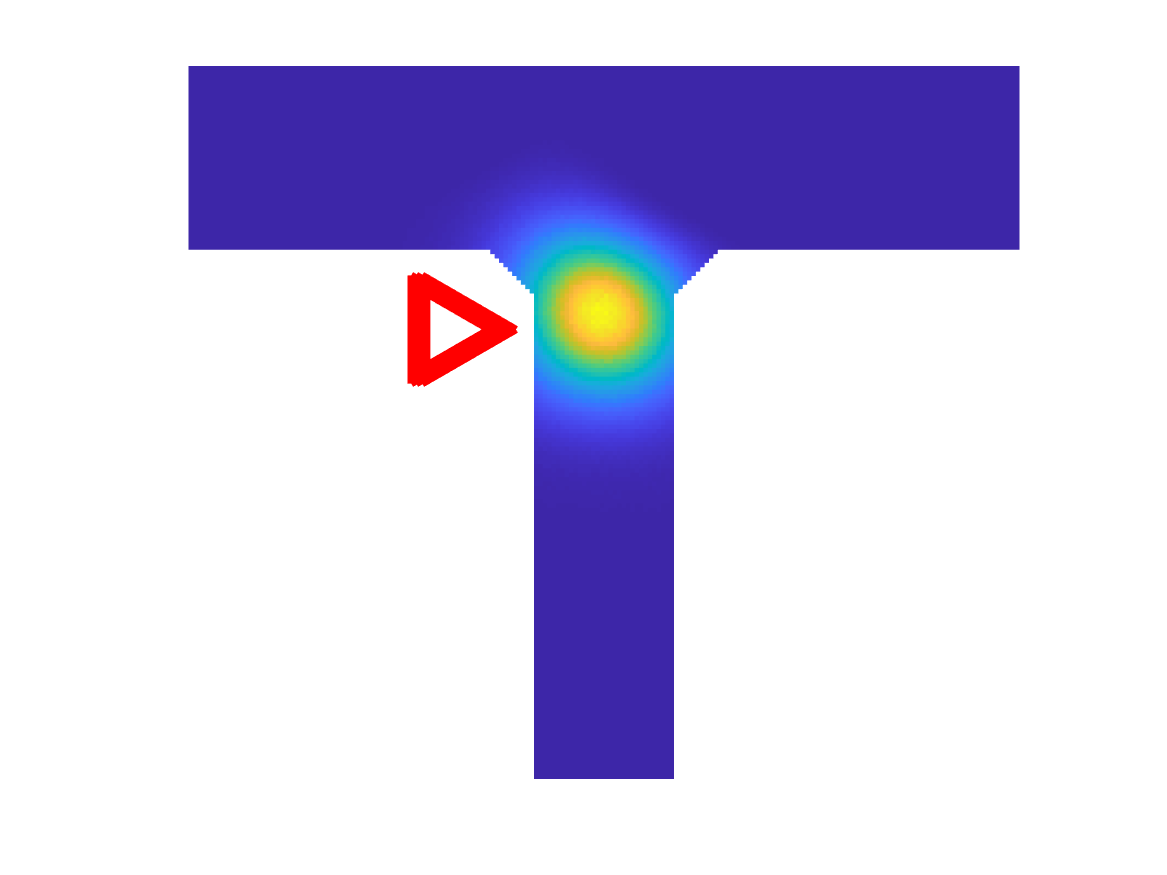

Supplement: Source code 1. [file elife-87055-code1.zip › code/fig5b_frames/156.bmp]

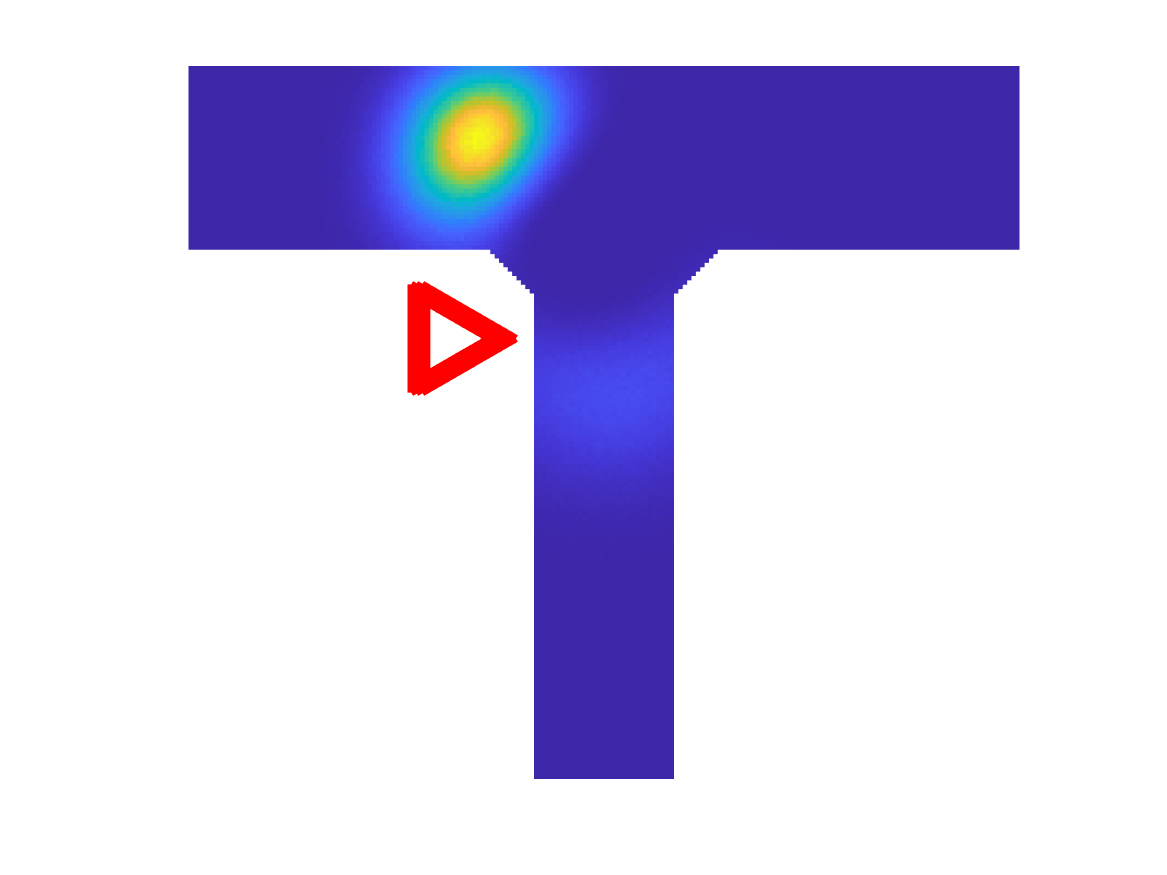

Supplement: Source code 1. [file elife-87055-code1.zip › code/fig5b_frames/142.bmp]

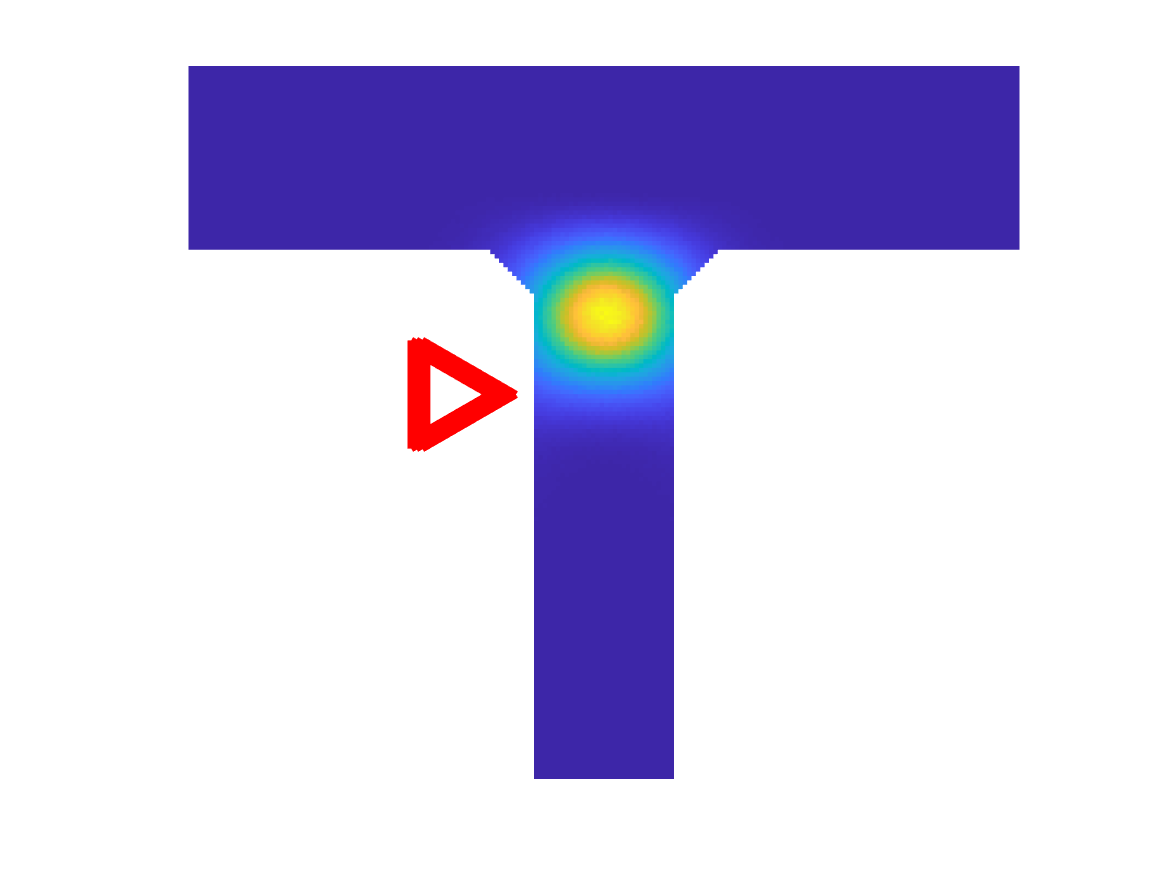

Supplement: Source code 1. [file elife-87055-code1.zip › code/fig5b_frames/57.bmp]

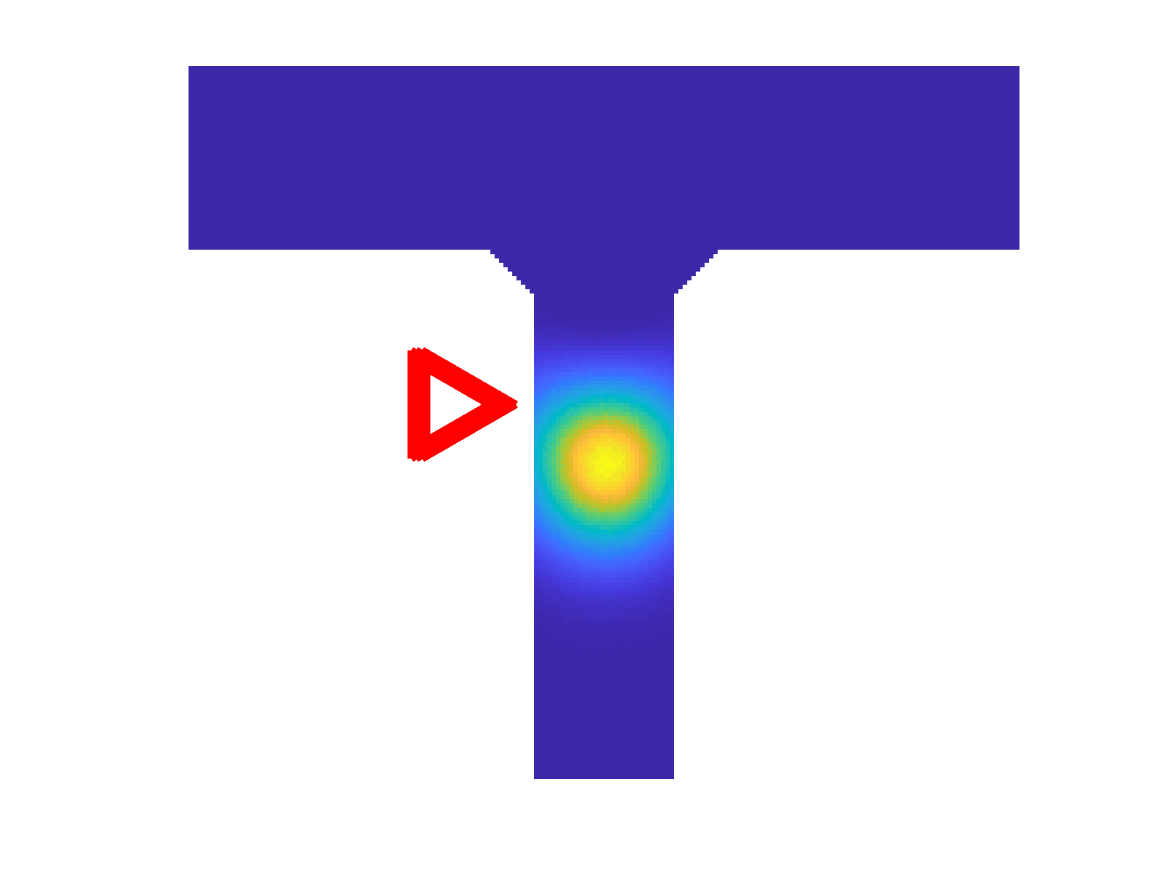

Supplement: Source code 1. [file elife-87055-code1.zip › code/fig5b_frames/43.bmp]

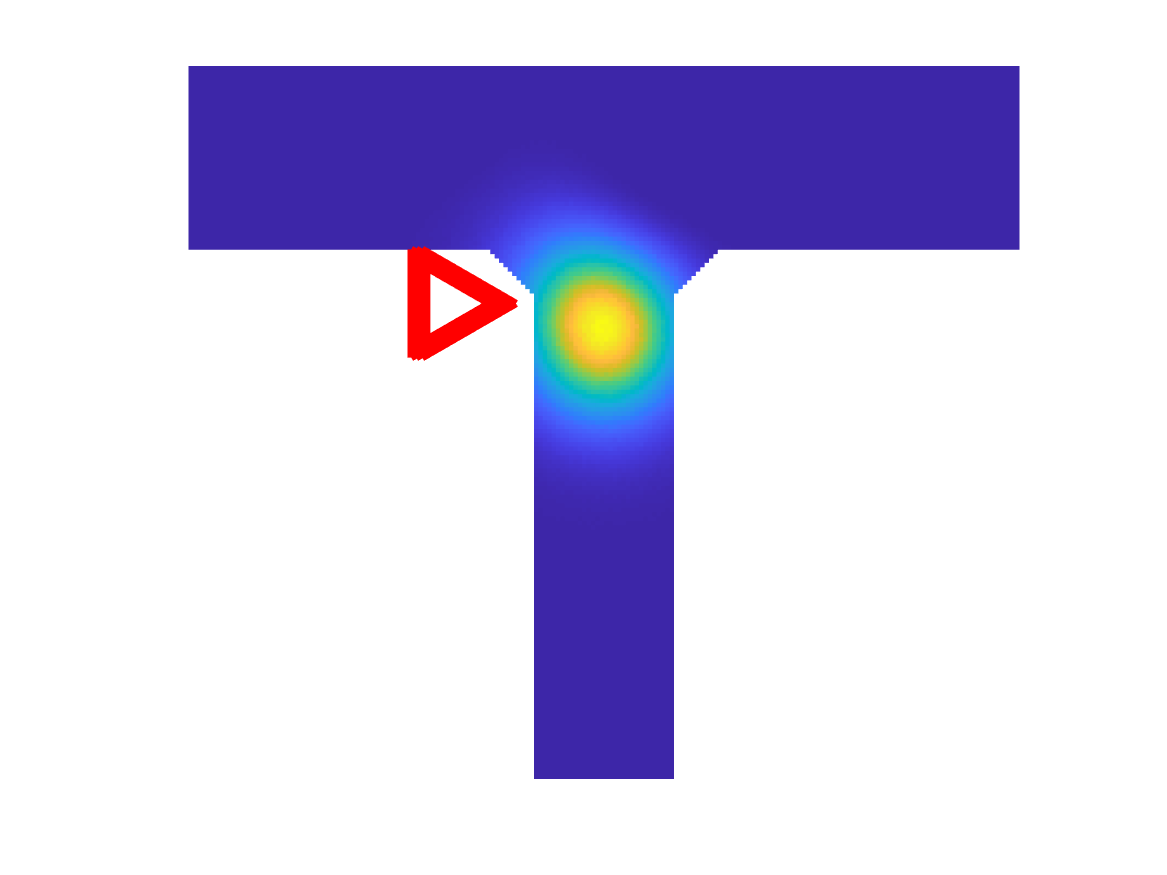

Supplement: Source code 1. [file elife-87055-code1.zip › code/fig5b_frames/195.bmp]

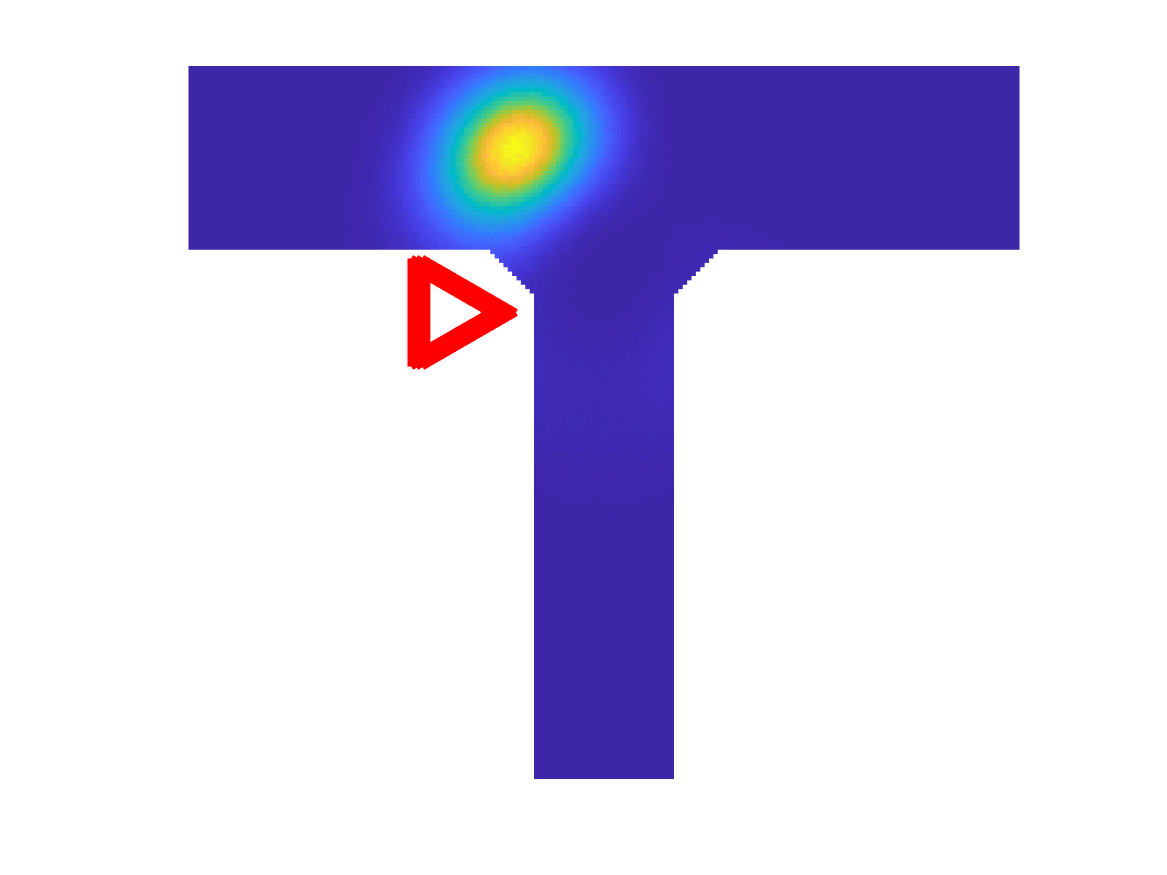

Supplement: Source code 1. [file elife-87055-code1.zip › code/fig5b_frames/181.bmp]

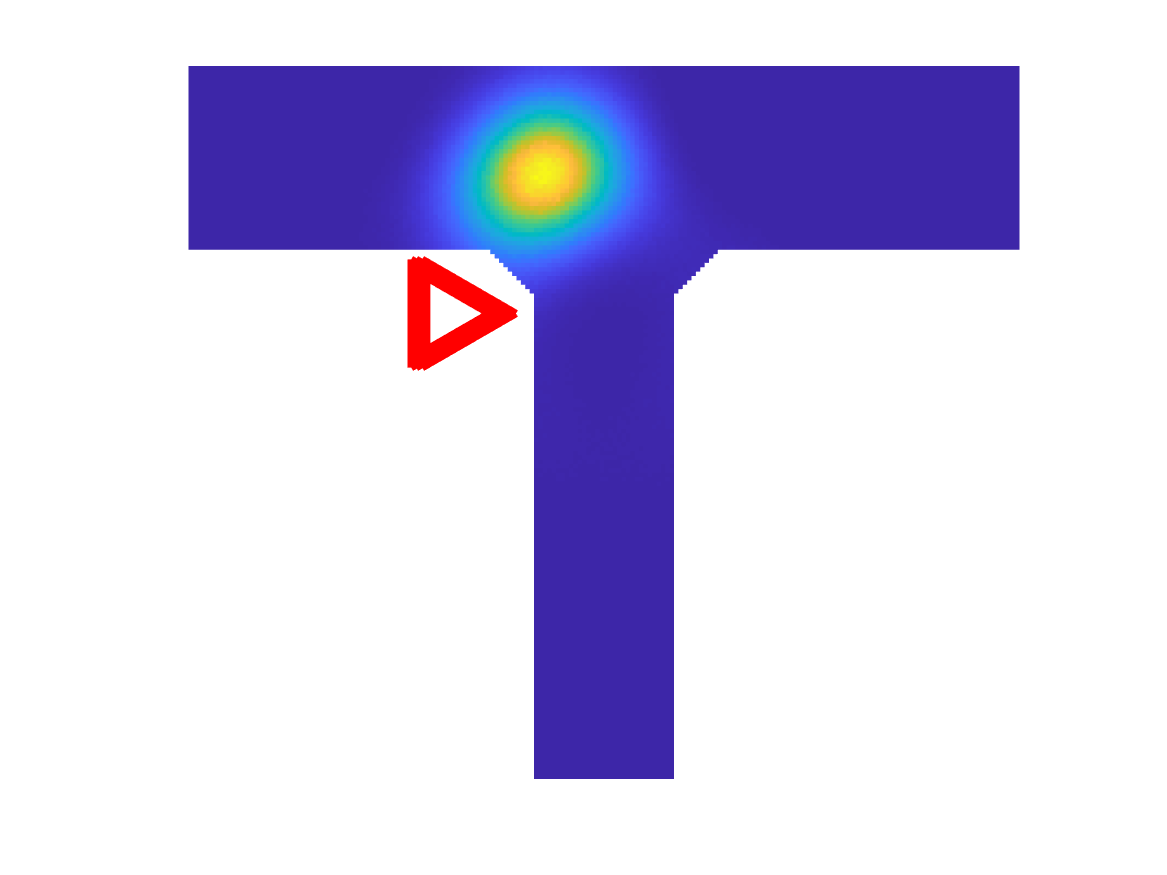

Supplement: Source code 1. [file elife-87055-code1.zip › code/fig5b_frames/180.bmp]

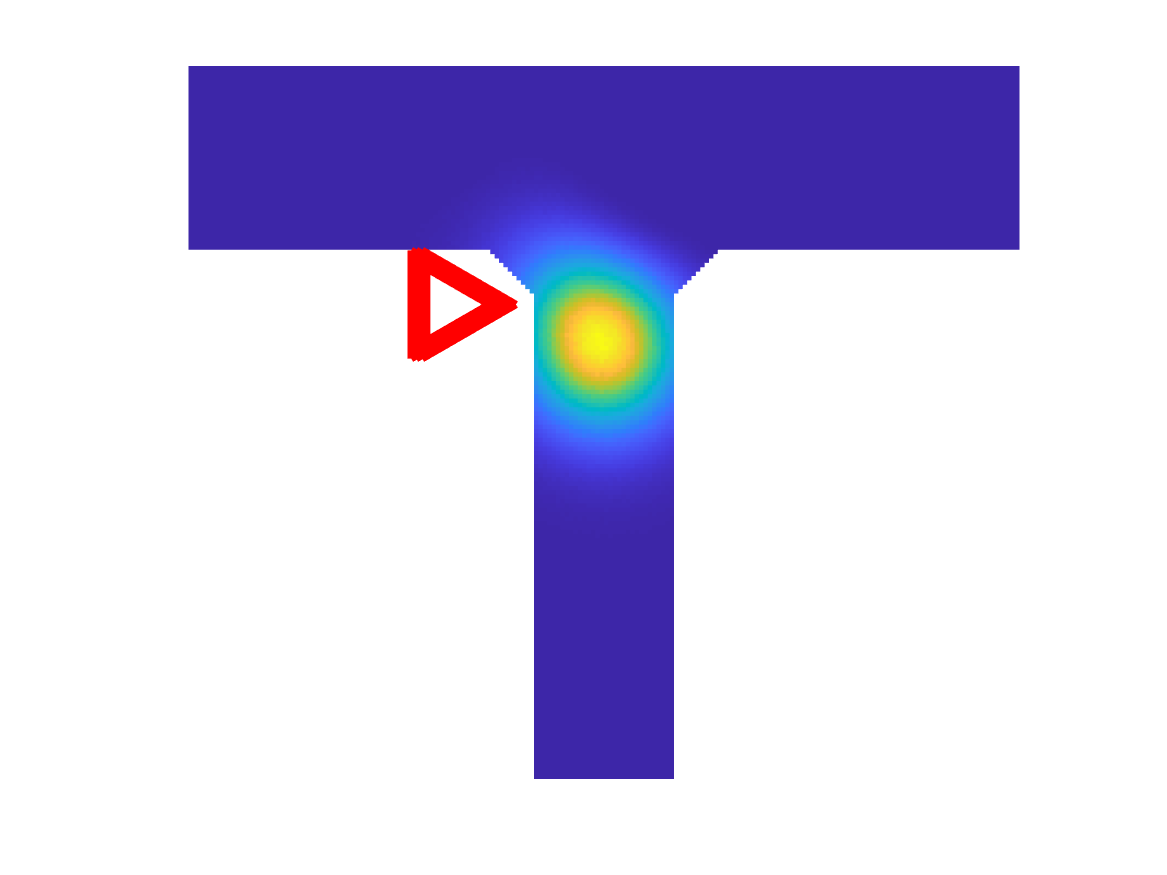

Supplement: Source code 1. [file elife-87055-code1.zip › code/fig5b_frames/194.bmp]

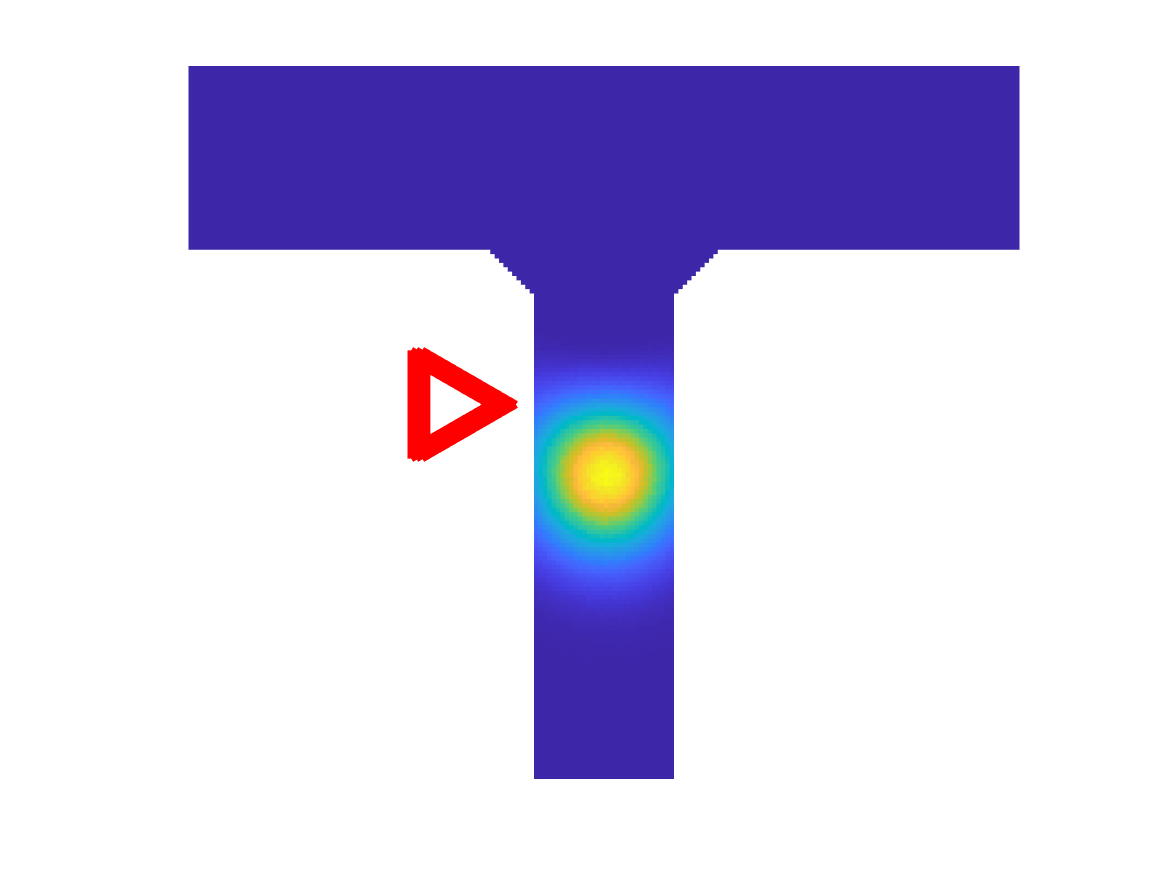

Supplement: Source code 1. [file elife-87055-code1.zip › code/fig5b_frames/42.bmp]

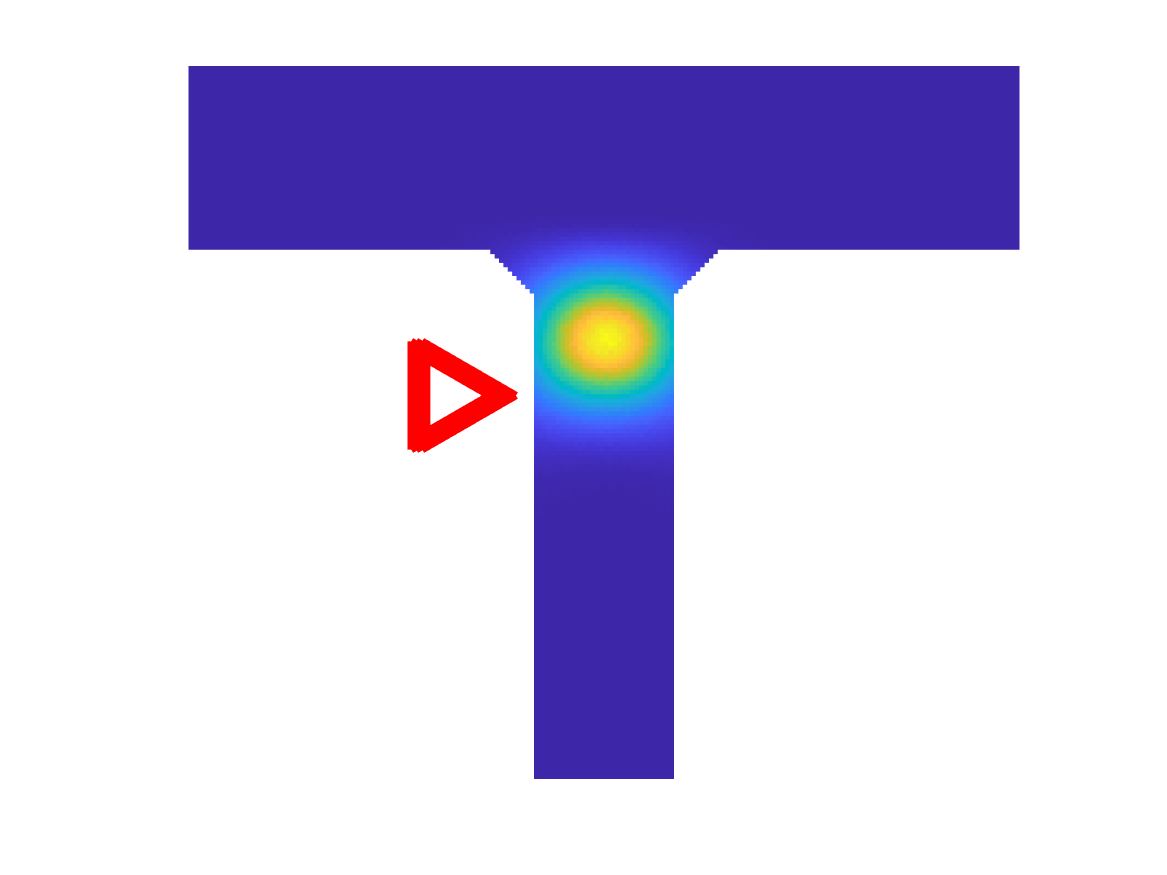

Supplement: Source code 1. [file elife-87055-code1.zip › code/fig5b_frames/56.bmp]

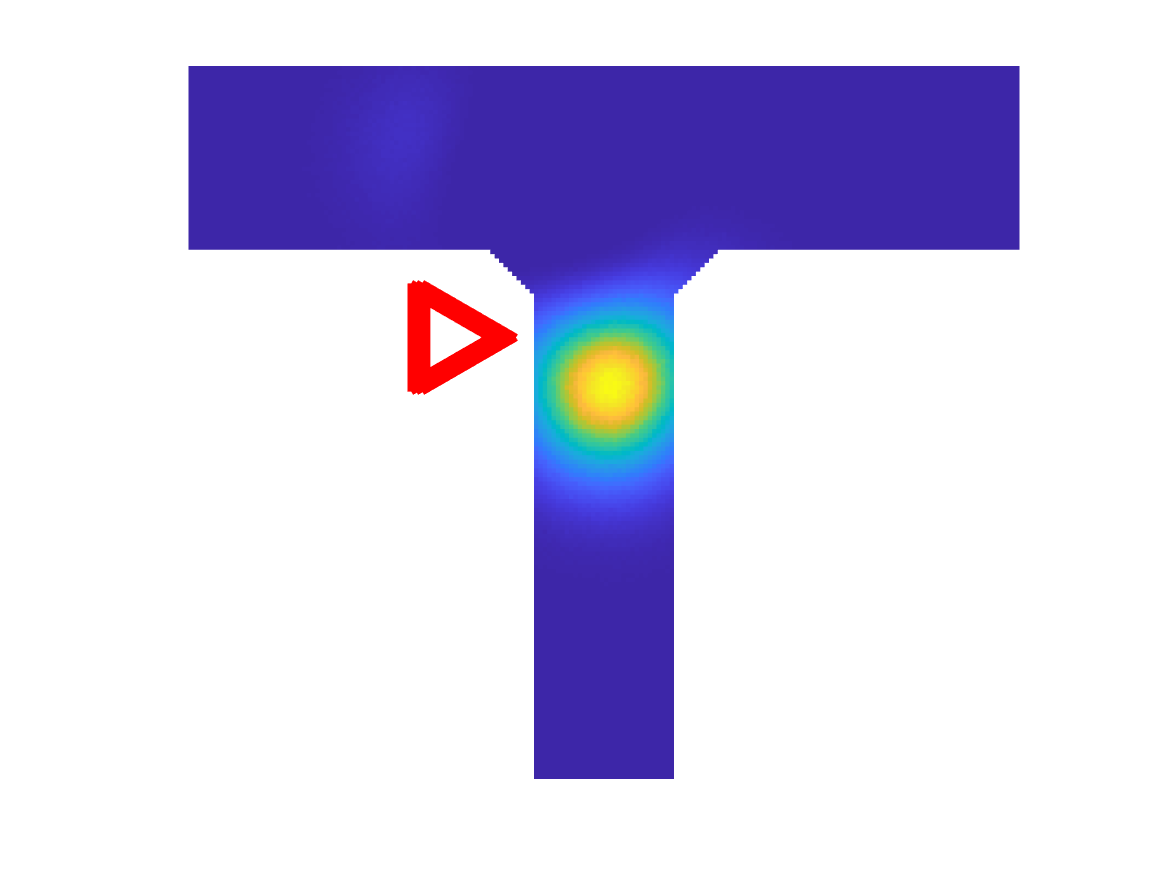

Supplement: Source code 1. [file elife-87055-code1.zip › code/fig5b_frames/143.bmp]

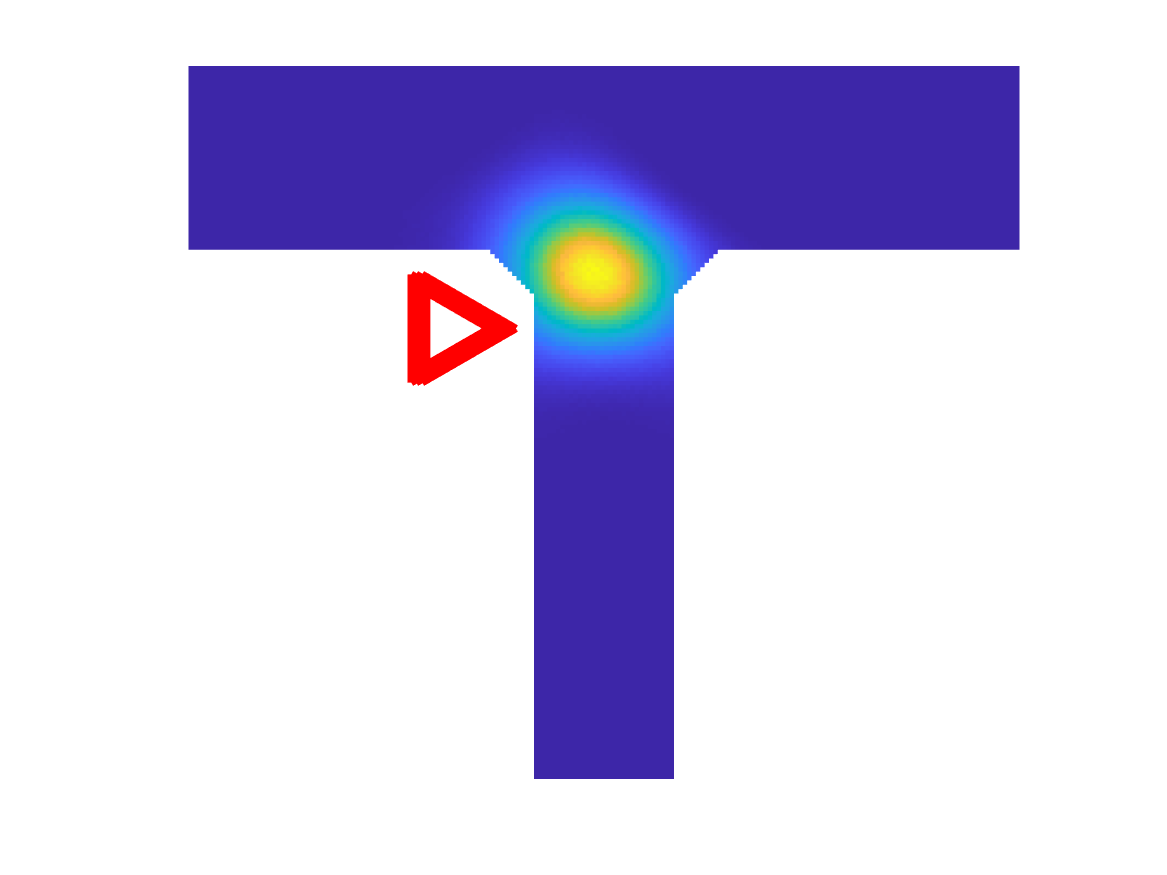

Supplement: Source code 1. [file elife-87055-code1.zip › code/fig5b_frames/157.bmp]

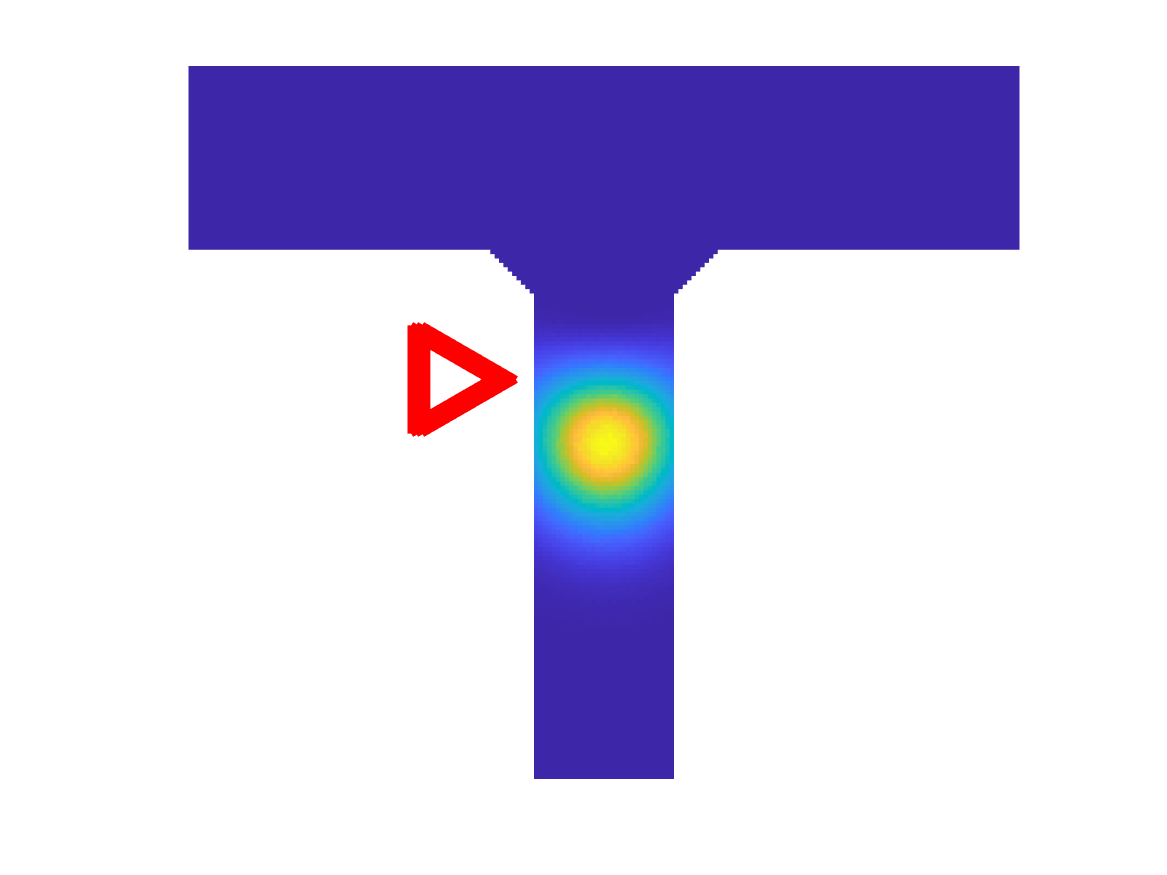

Supplement: Source code 1. [file elife-87055-code1.zip › code/fig5b_frames/81.bmp]

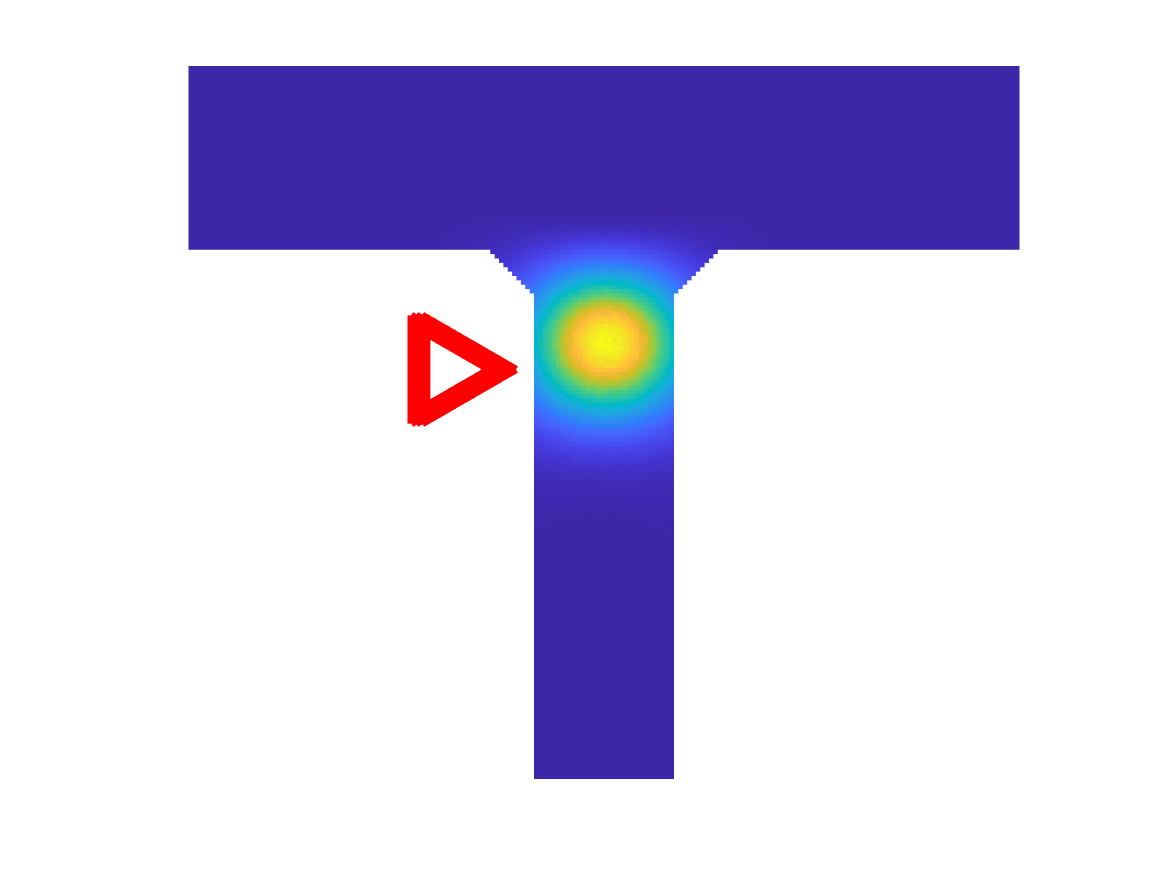

Supplement: Source code 1. [file elife-87055-code1.zip › code/fig5b_frames/95.bmp]

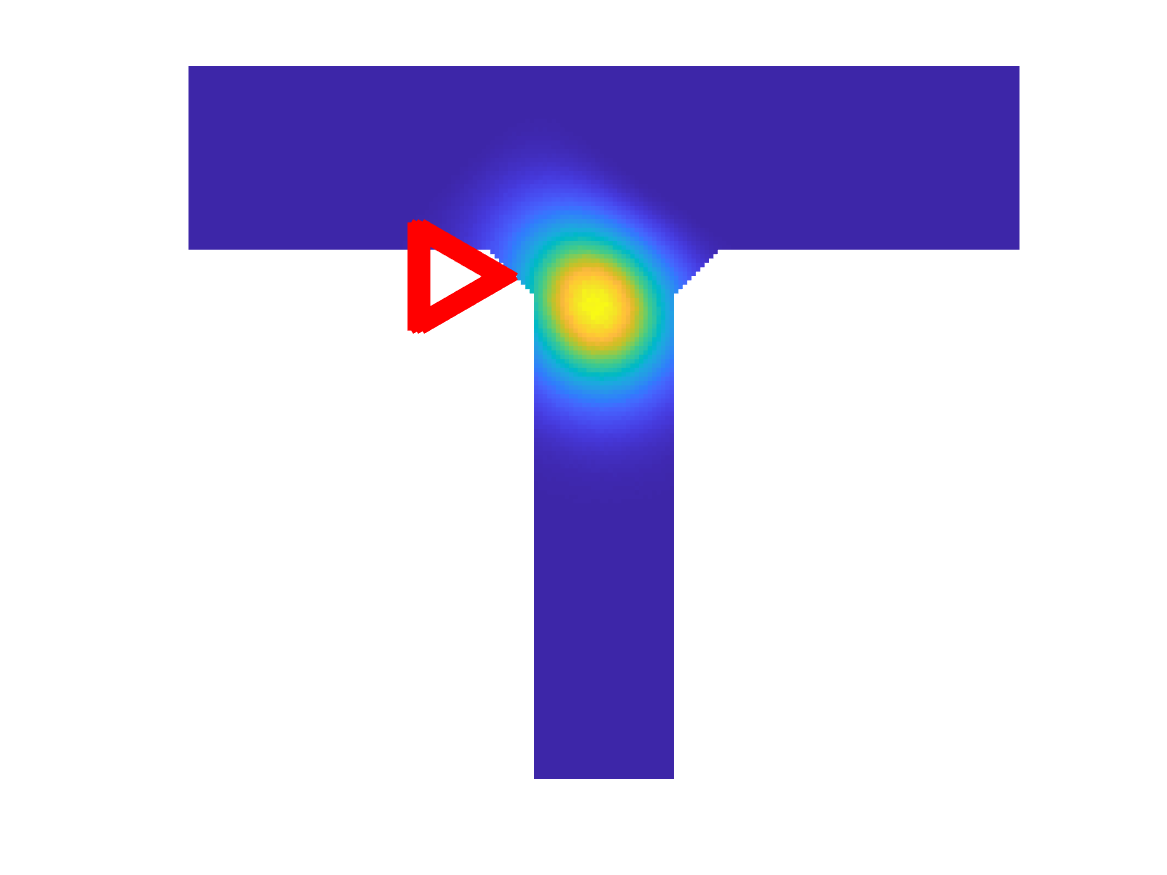

Supplement: Source code 1. [file elife-87055-code1.zip › code/fig5b_frames/235.bmp]

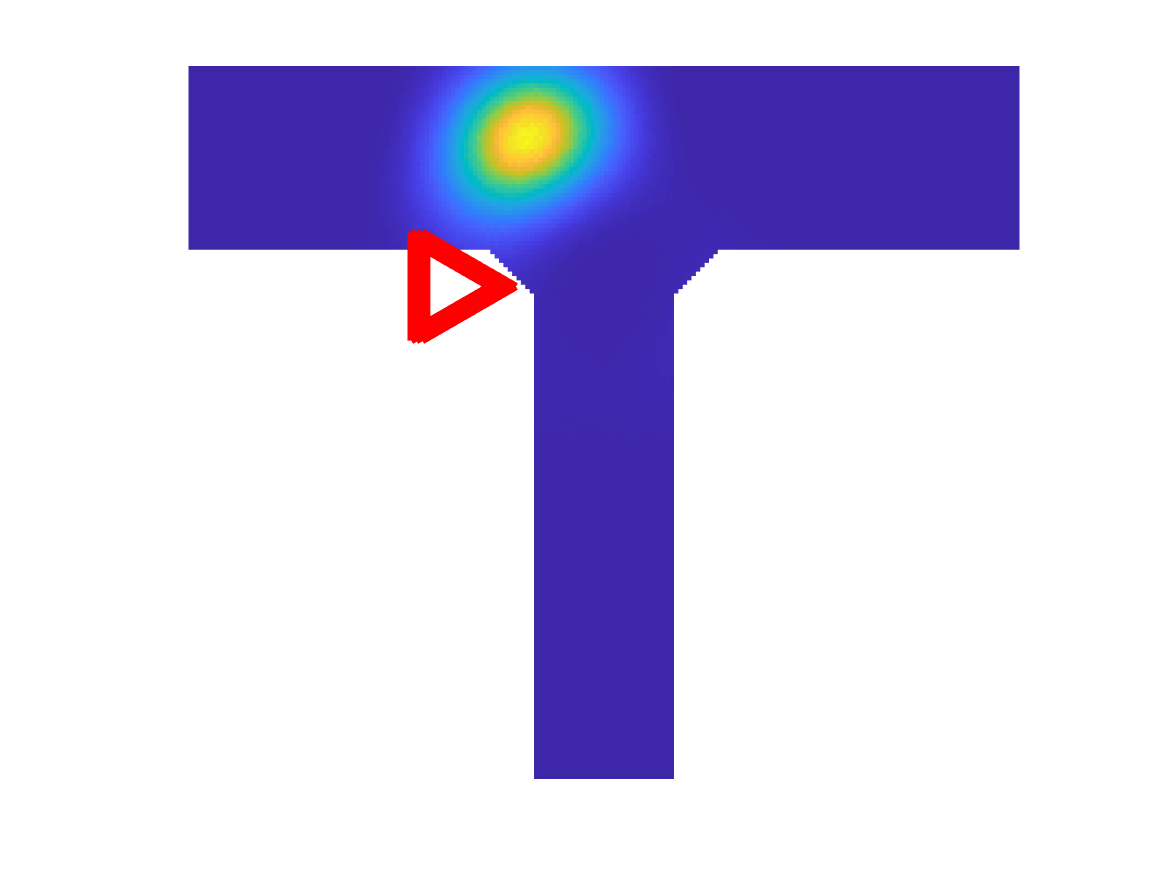

Supplement: Source code 1. [file elife-87055-code1.zip › code/fig5b_frames/221.bmp]

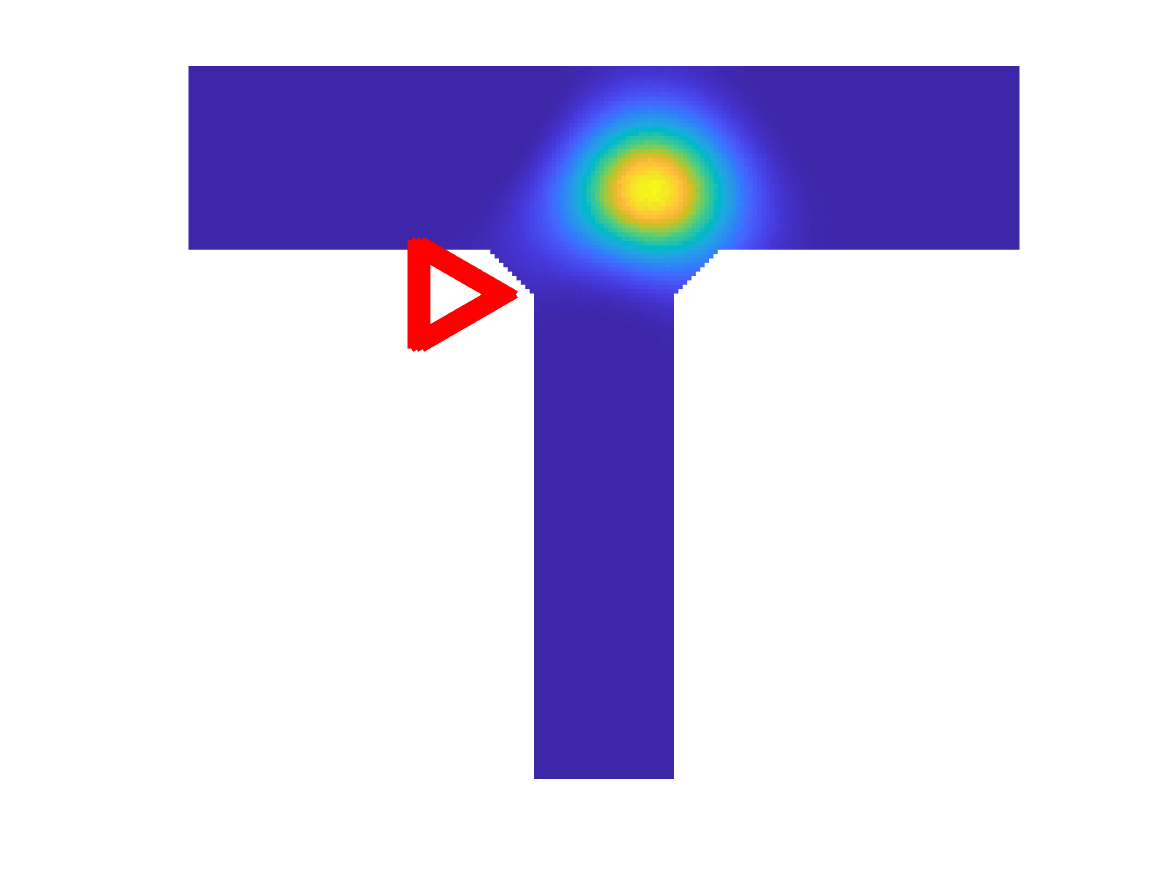

Supplement: Source code 1. [file elife-87055-code1.zip › code/fig5b_frames/209.bmp]

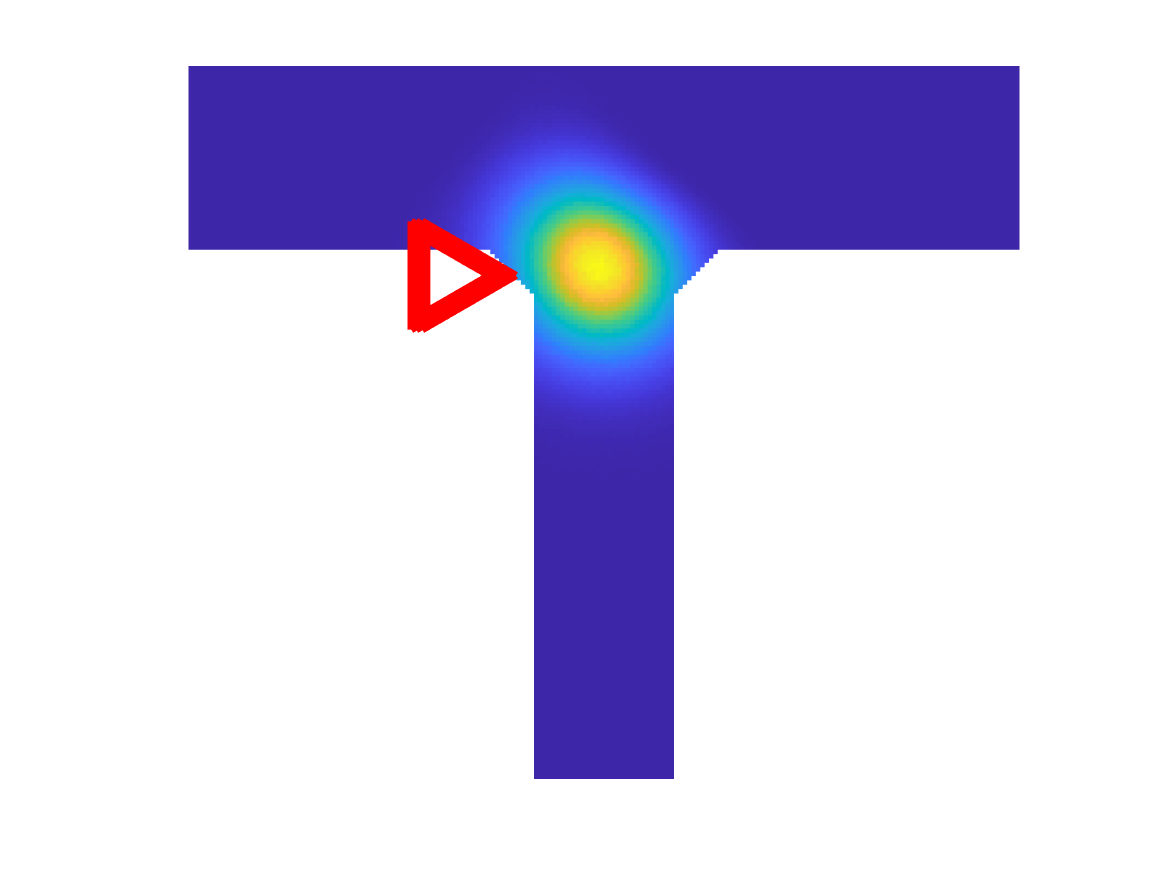

Supplement: Source code 1. [file elife-87055-code1.zip › code/fig5b_frames/237.bmp]

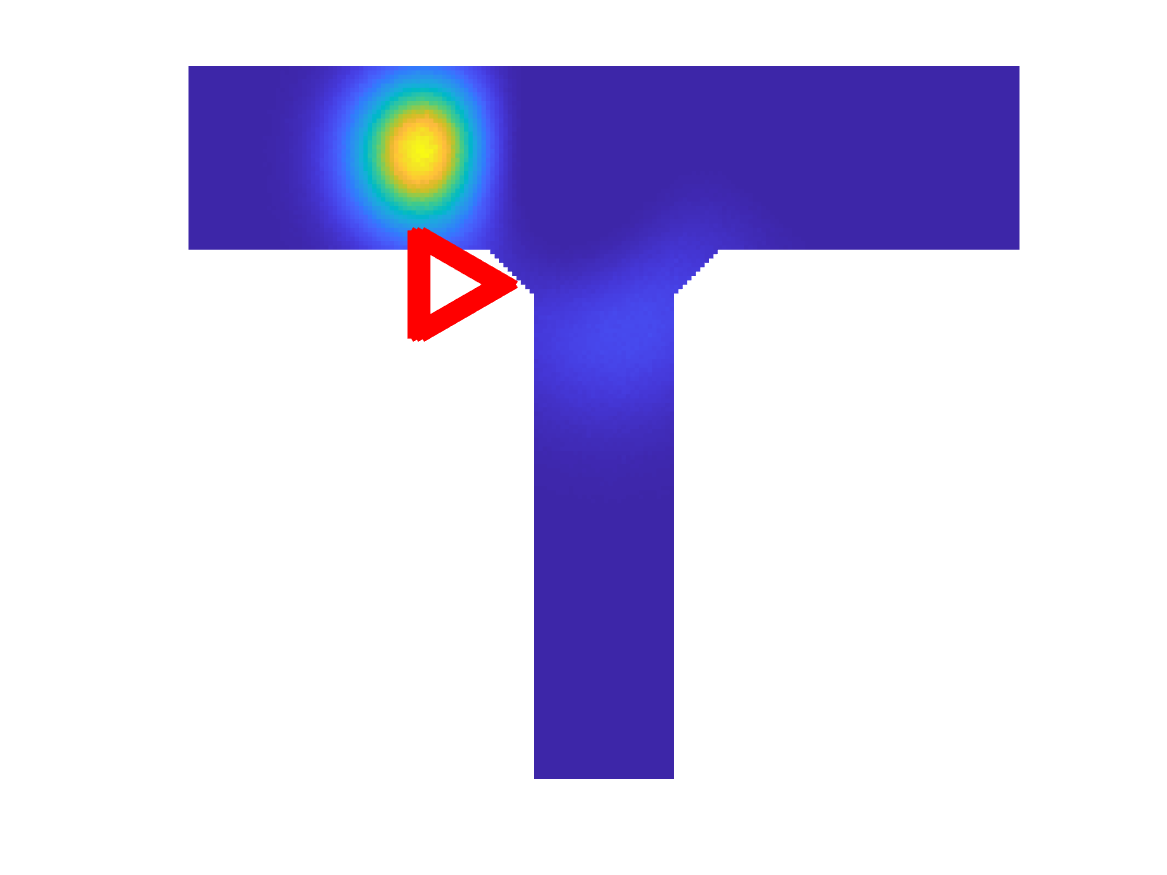

Supplement: Source code 1. [file elife-87055-code1.zip › code/fig5b_frames/223.bmp]

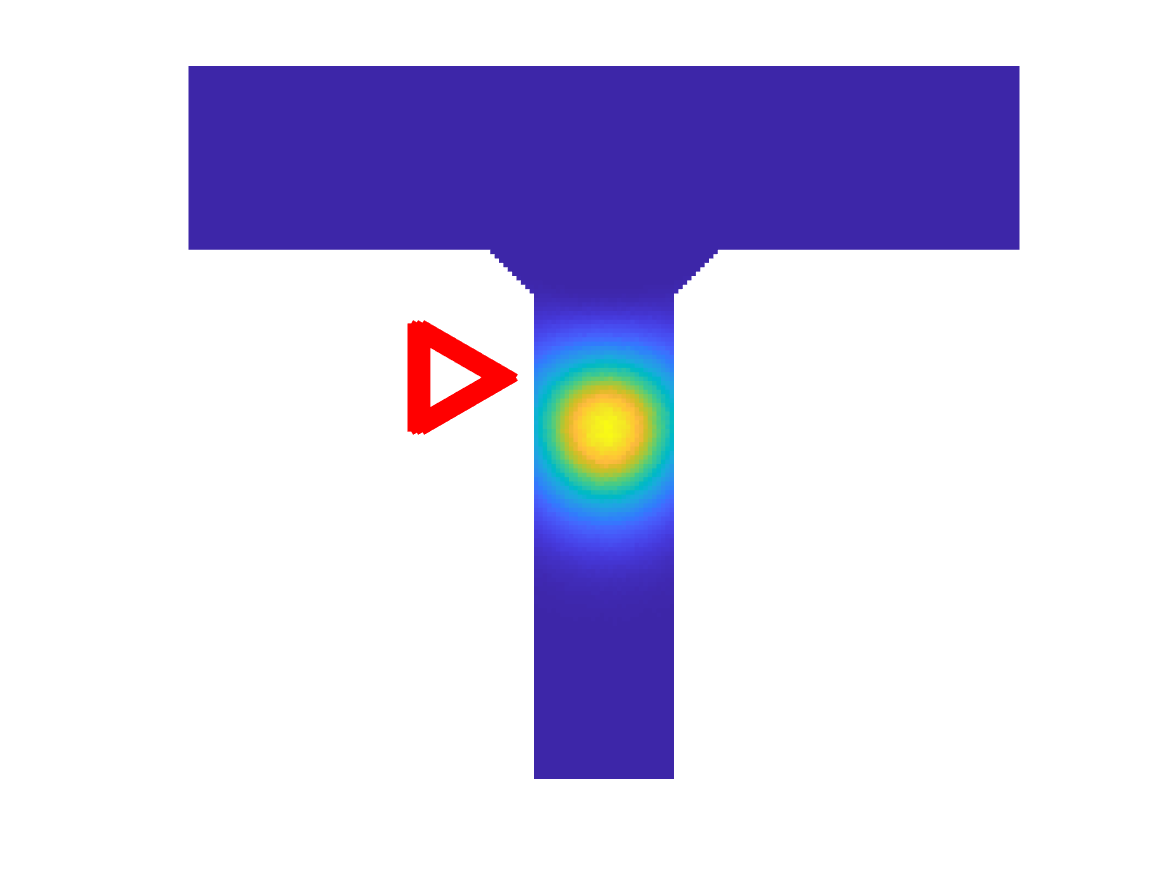

Supplement: Source code 1. [file elife-87055-code1.zip › code/fig5b_frames/83.bmp]

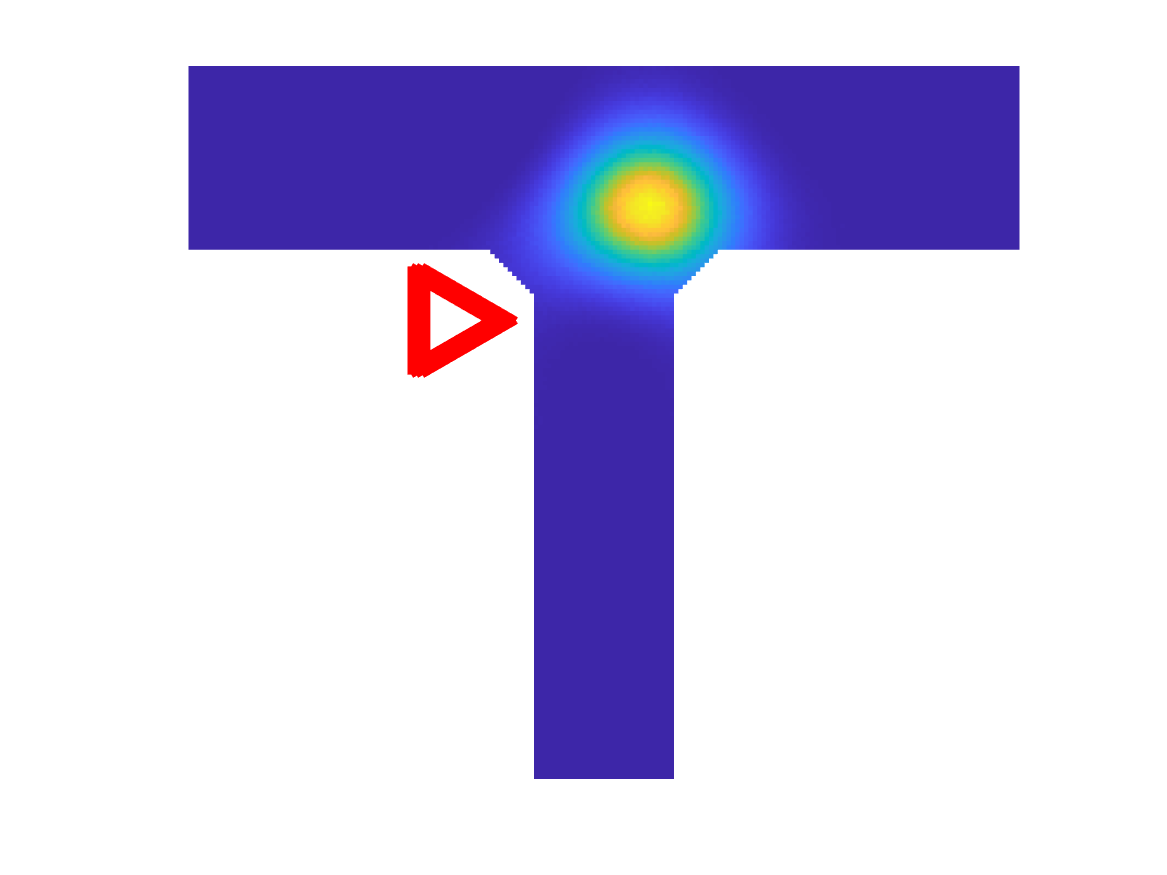

Supplement: Source code 1. [file elife-87055-code1.zip › code/fig5b_frames/169.bmp]

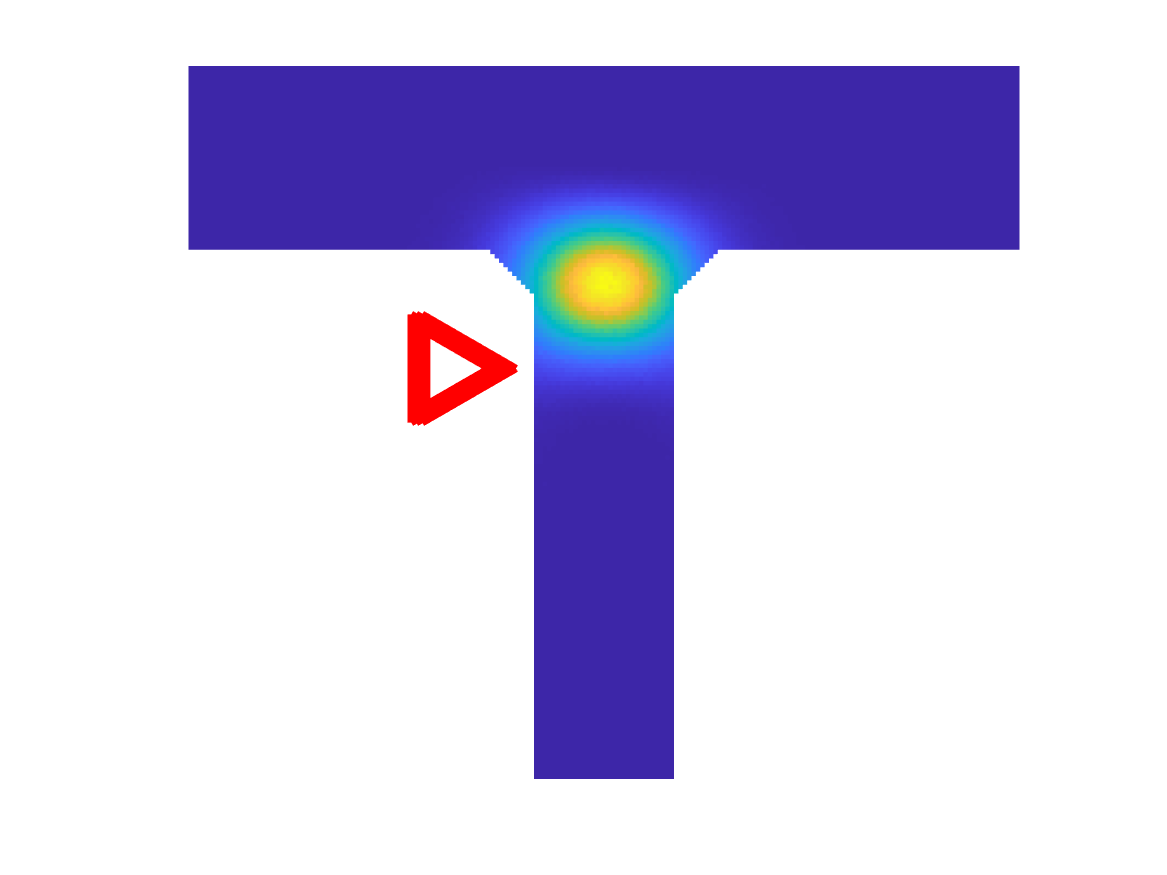

Supplement: Source code 1. [file elife-87055-code1.zip › code/fig5b_frames/97.bmp]

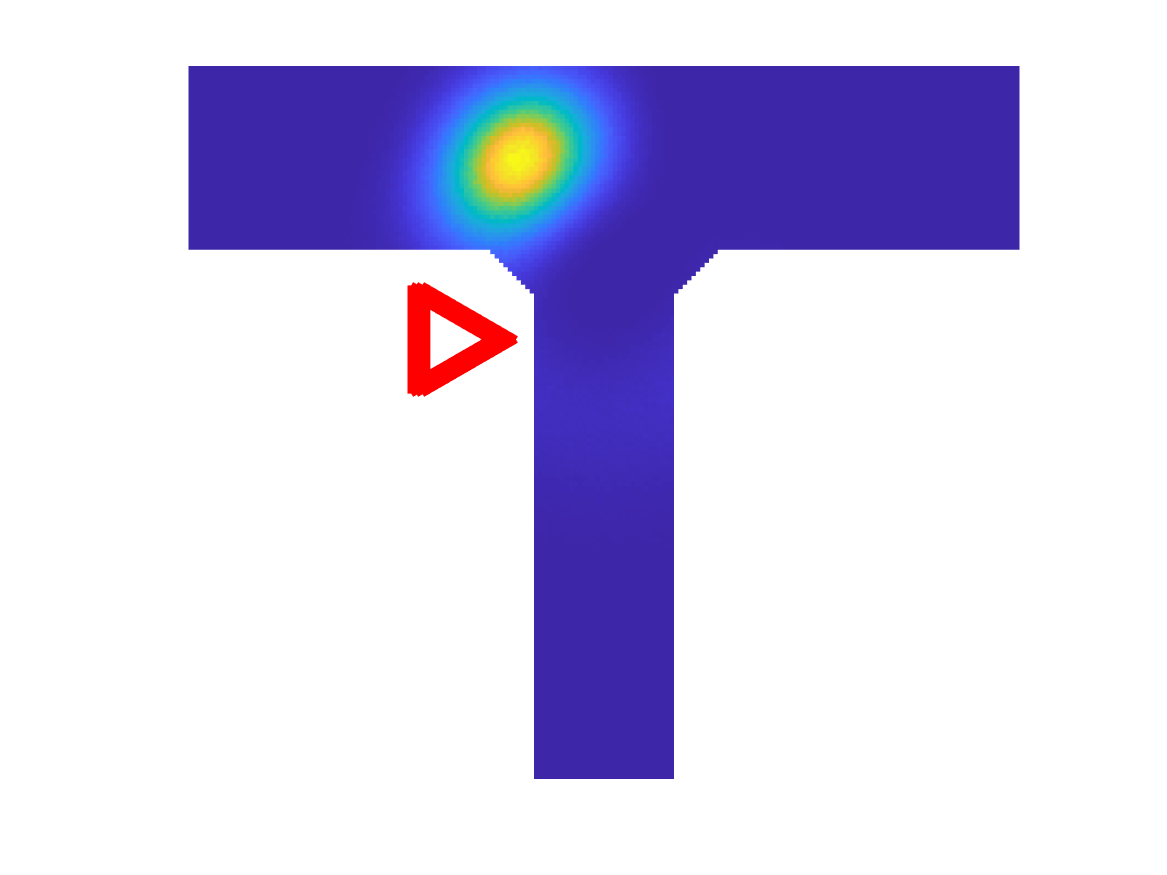

Supplement: Source code 1. [file elife-87055-code1.zip › code/fig5b_frames/141.bmp]

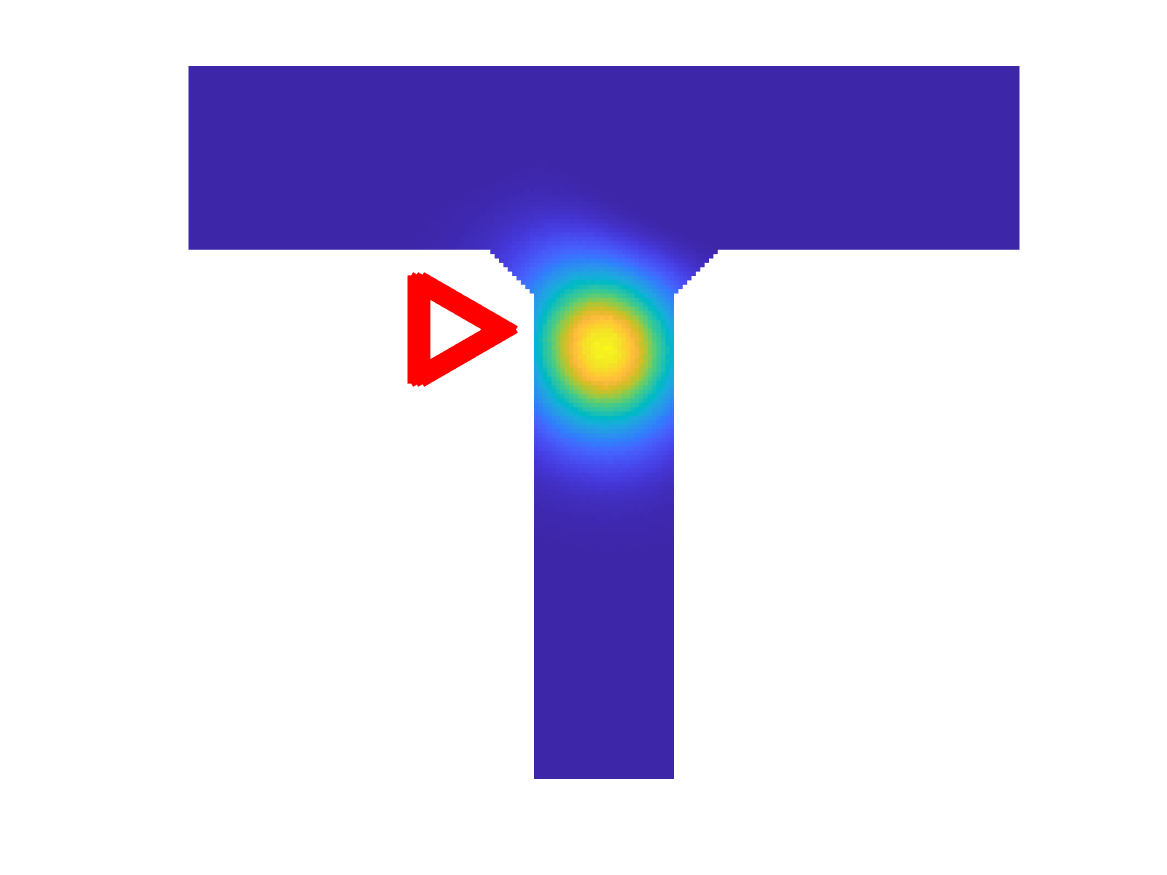

Supplement: Source code 1. [file elife-87055-code1.zip › code/fig5b_frames/155.bmp]

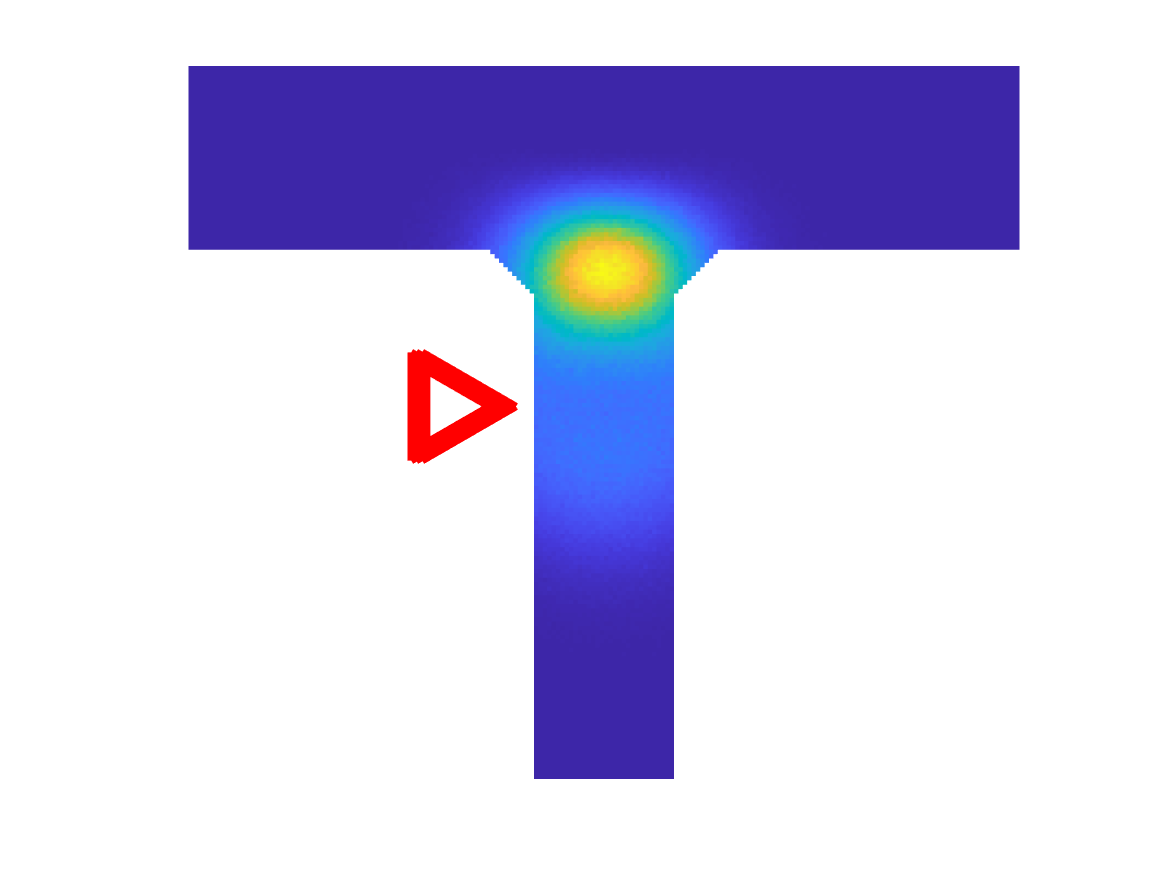

Supplement: Source code 1. [file elife-87055-code1.zip › code/fig5b_frames/40.bmp]

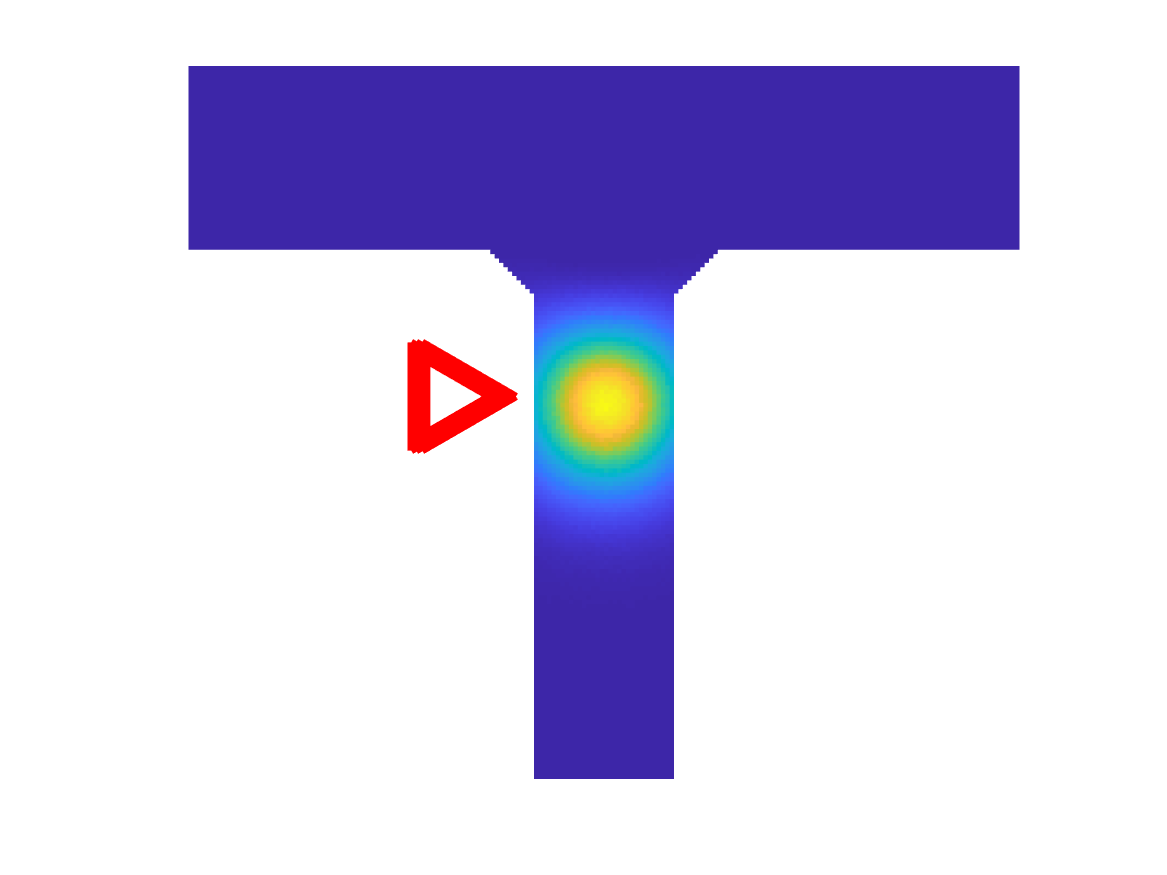

Supplement: Source code 1. [file elife-87055-code1.zip › code/fig5b_frames/54.bmp]

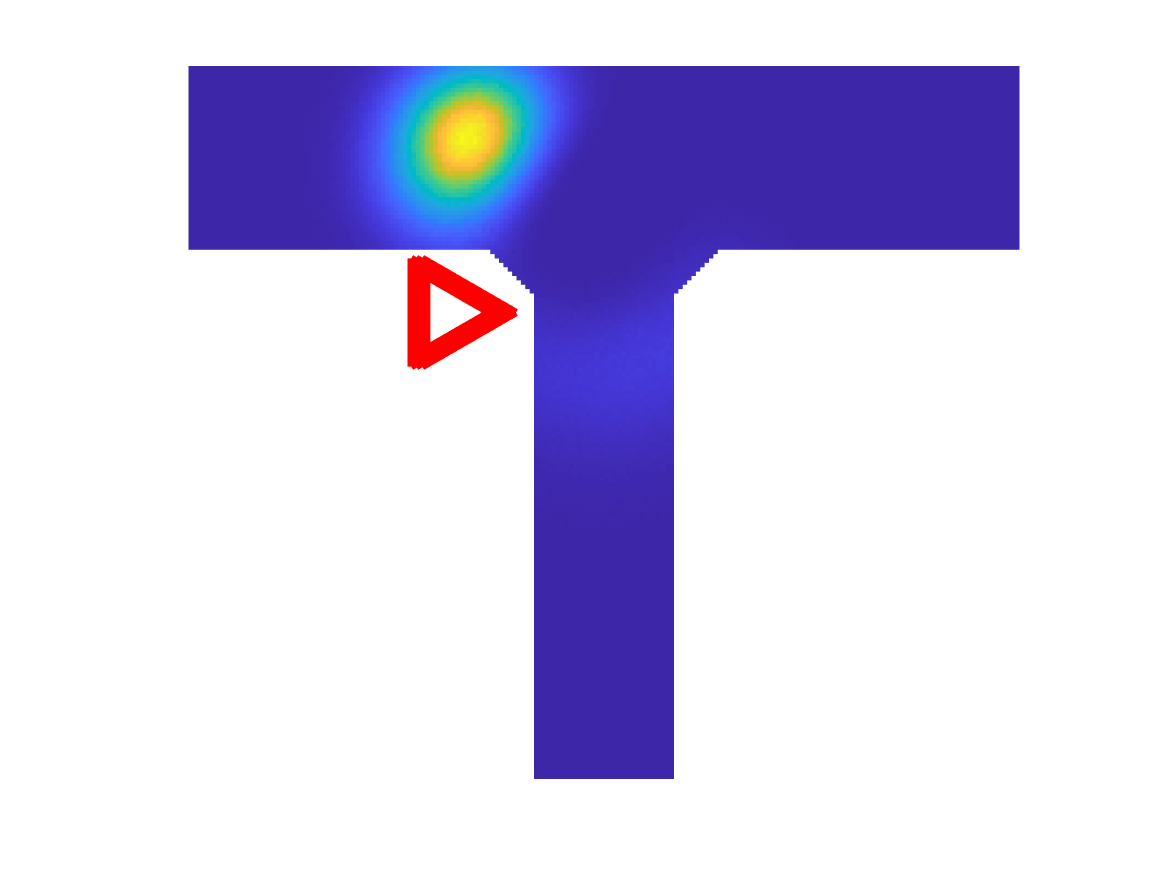

Supplement: Source code 1. [file elife-87055-code1.zip › code/fig5b_frames/182.bmp]

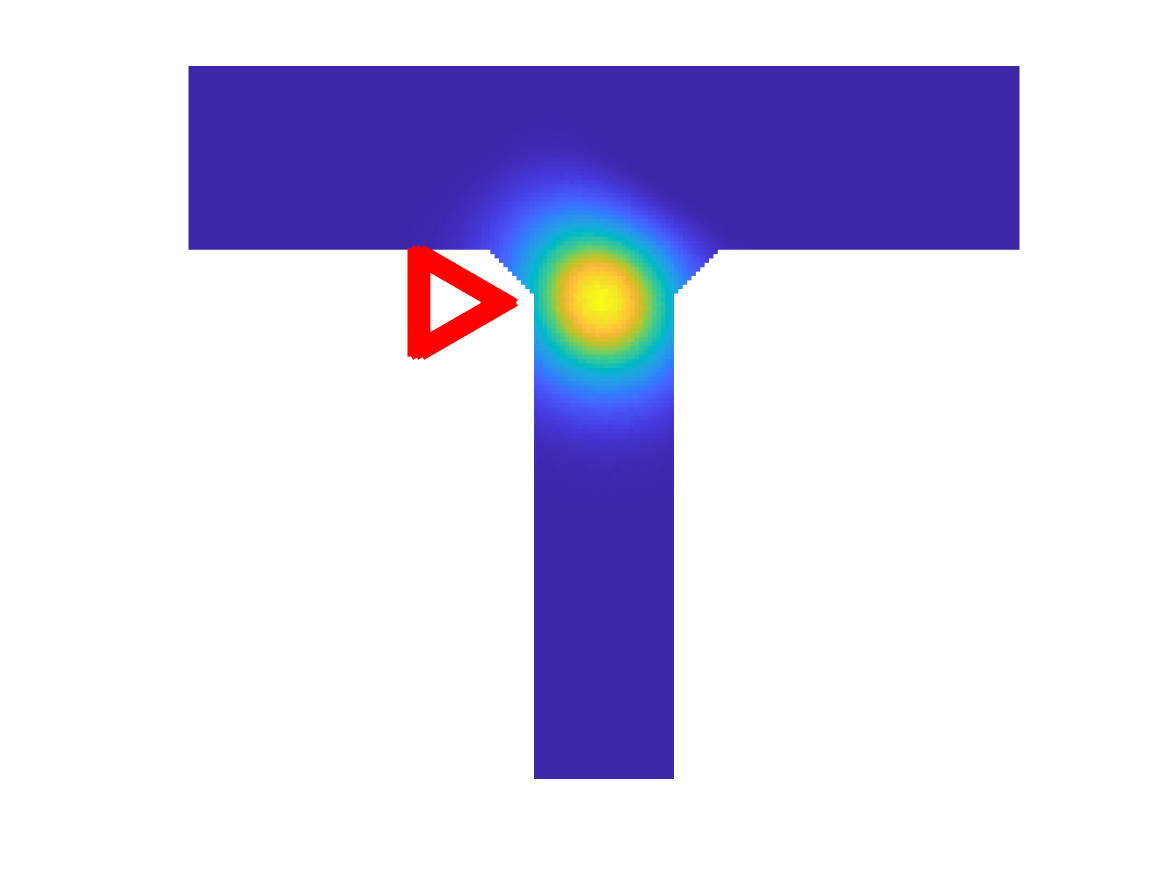

Supplement: Source code 1. [file elife-87055-code1.zip › code/fig5b_frames/196.bmp]

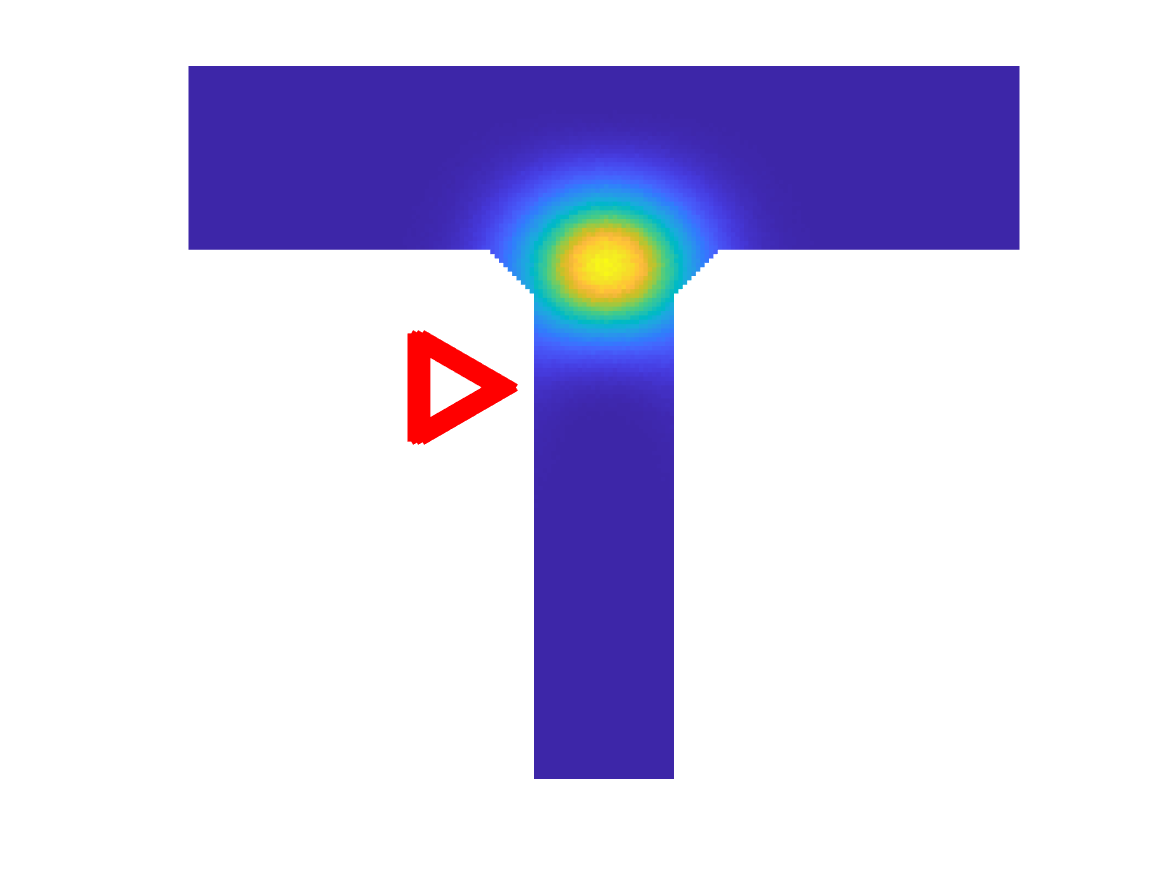

Supplement: Source code 1. [file elife-87055-code1.zip › code/fig5b_frames/68.bmp]

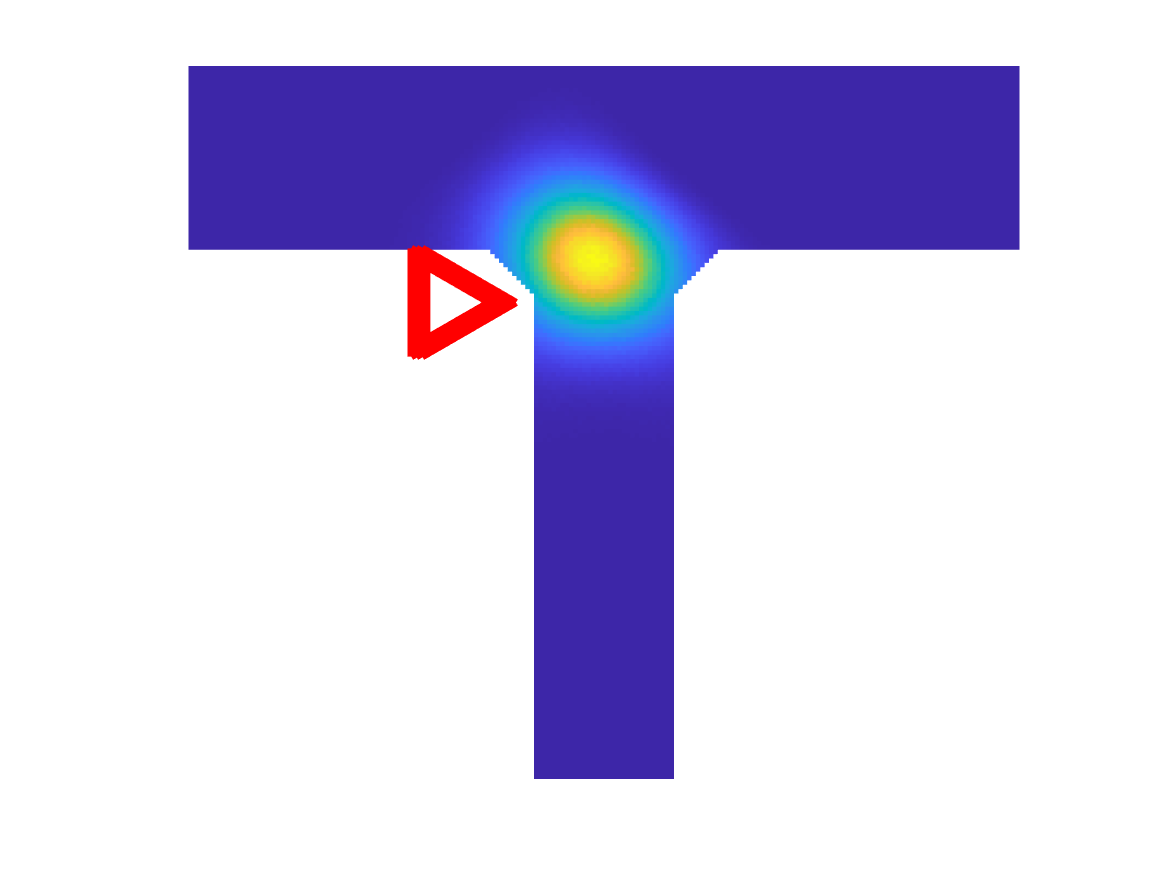

Supplement: Source code 1. [file elife-87055-code1.zip › code/fig5b_frames/197.bmp]

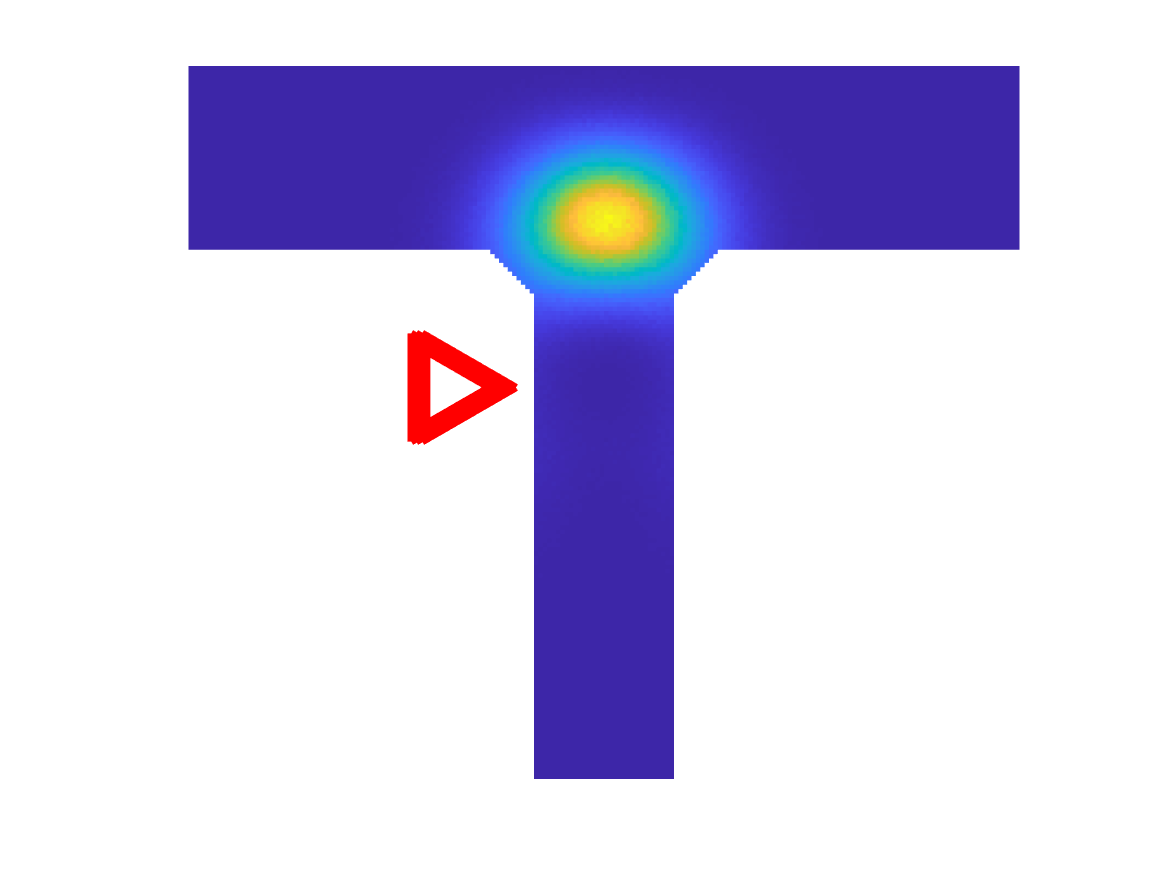

Supplement: Source code 1. [file elife-87055-code1.zip › code/fig5b_frames/69.bmp]

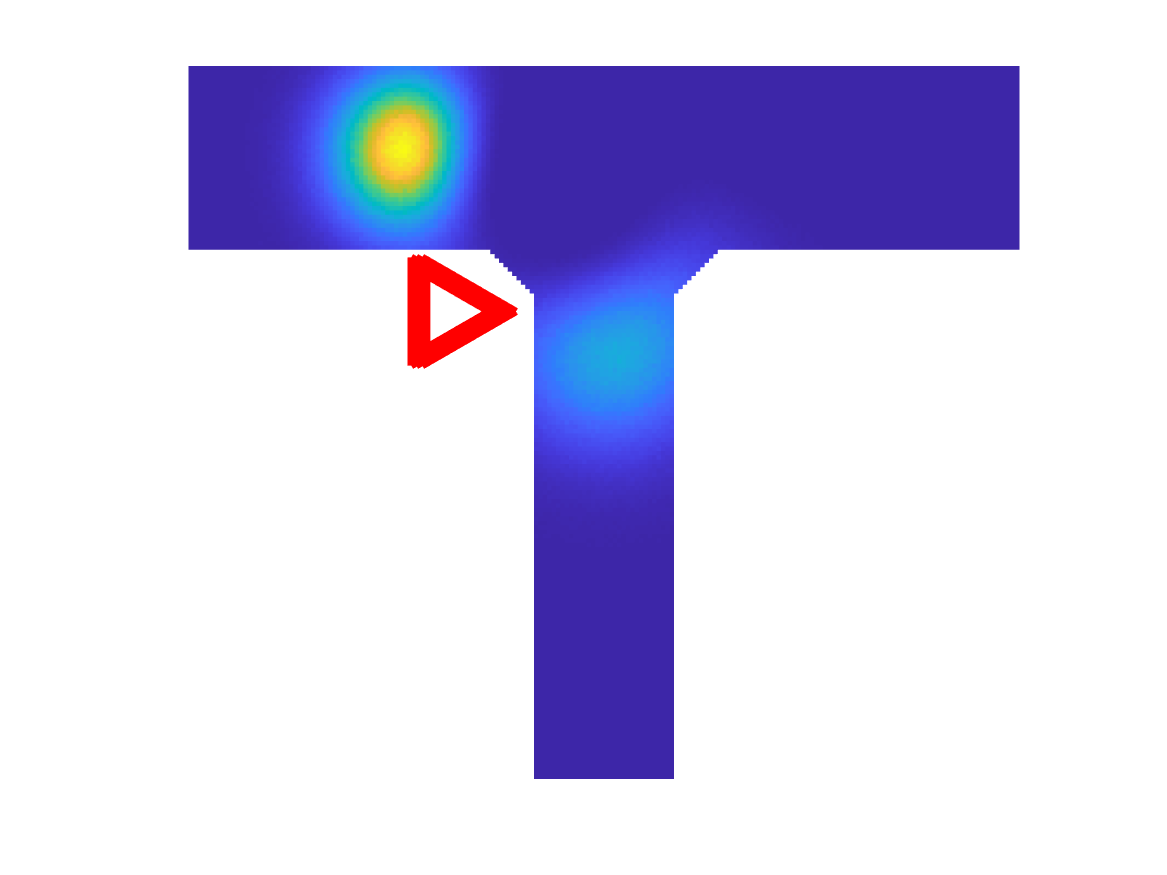

Supplement: Source code 1. [file elife-87055-code1.zip › code/fig5b_frames/183.bmp]

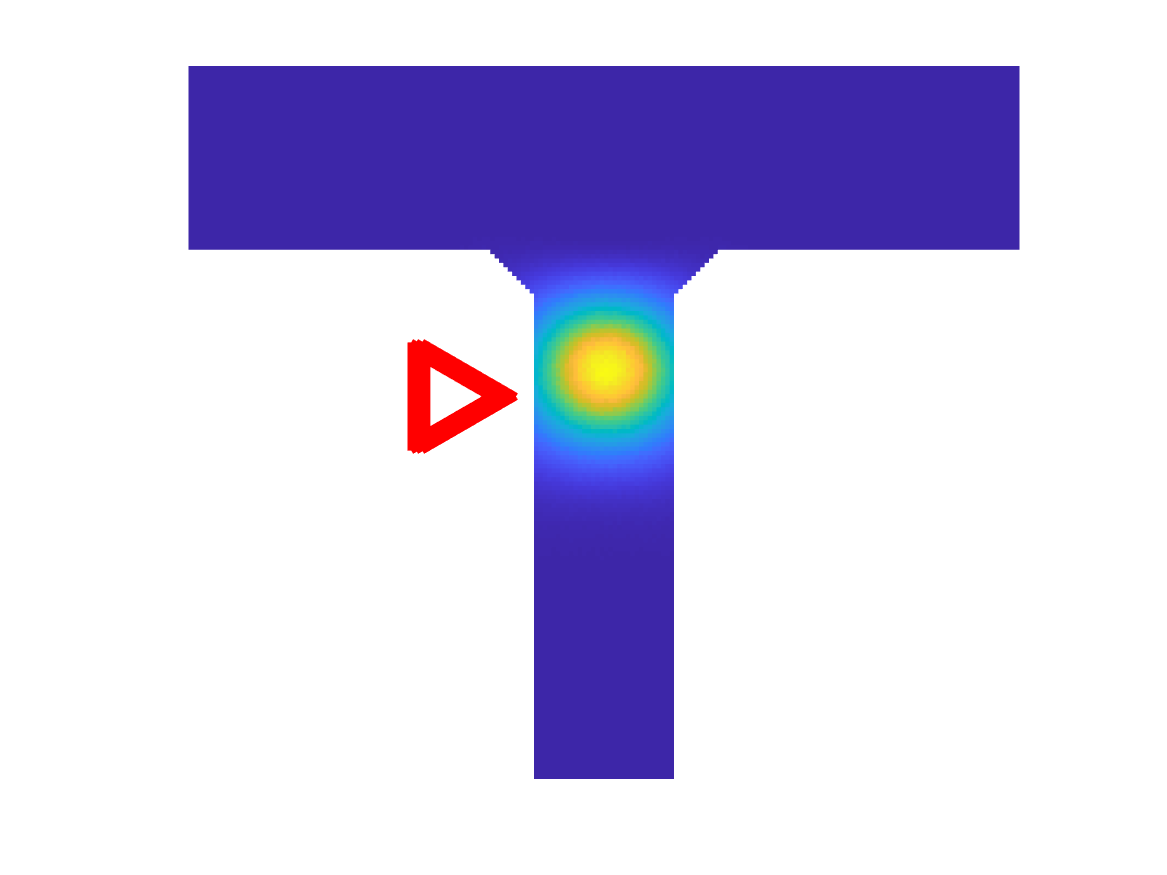

Supplement: Source code 1. [file elife-87055-code1.zip › code/fig5b_frames/55.bmp]

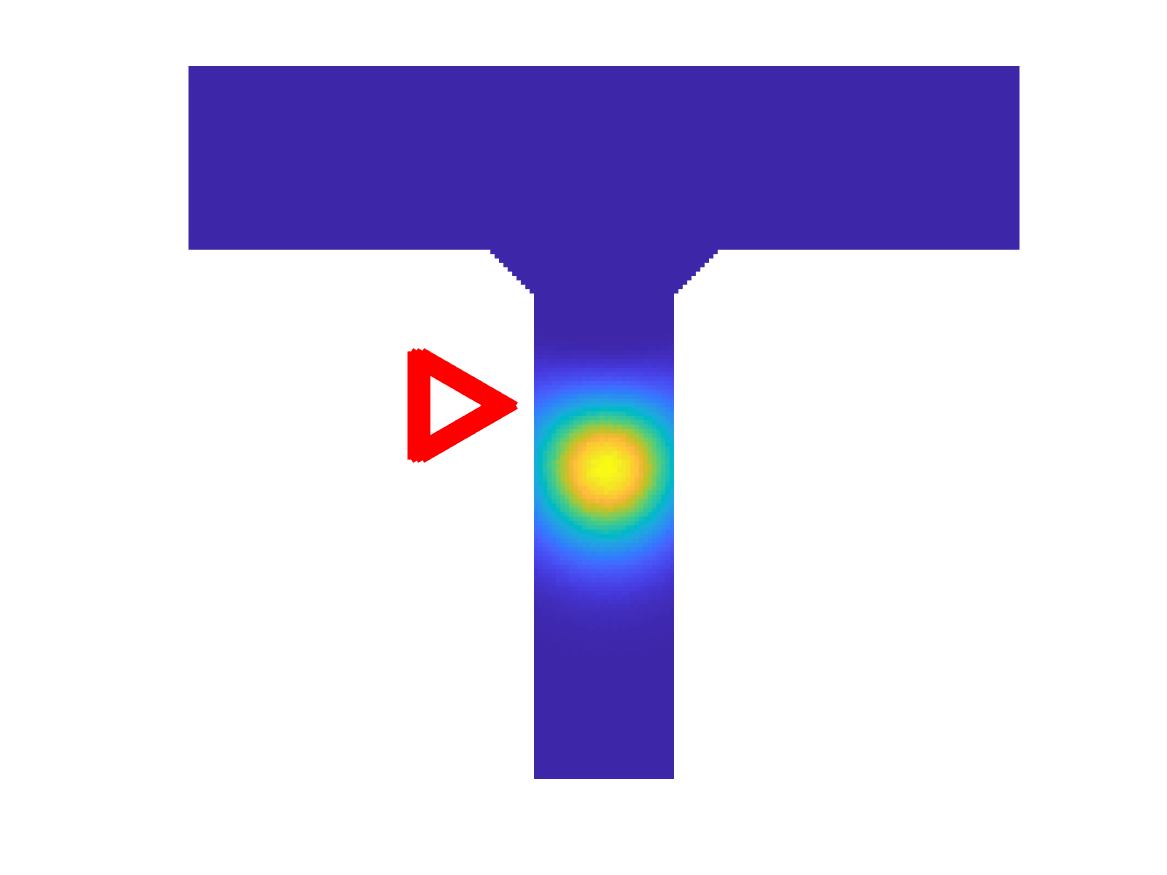

Supplement: Source code 1. [file elife-87055-code1.zip › code/fig5b_frames/41.bmp]

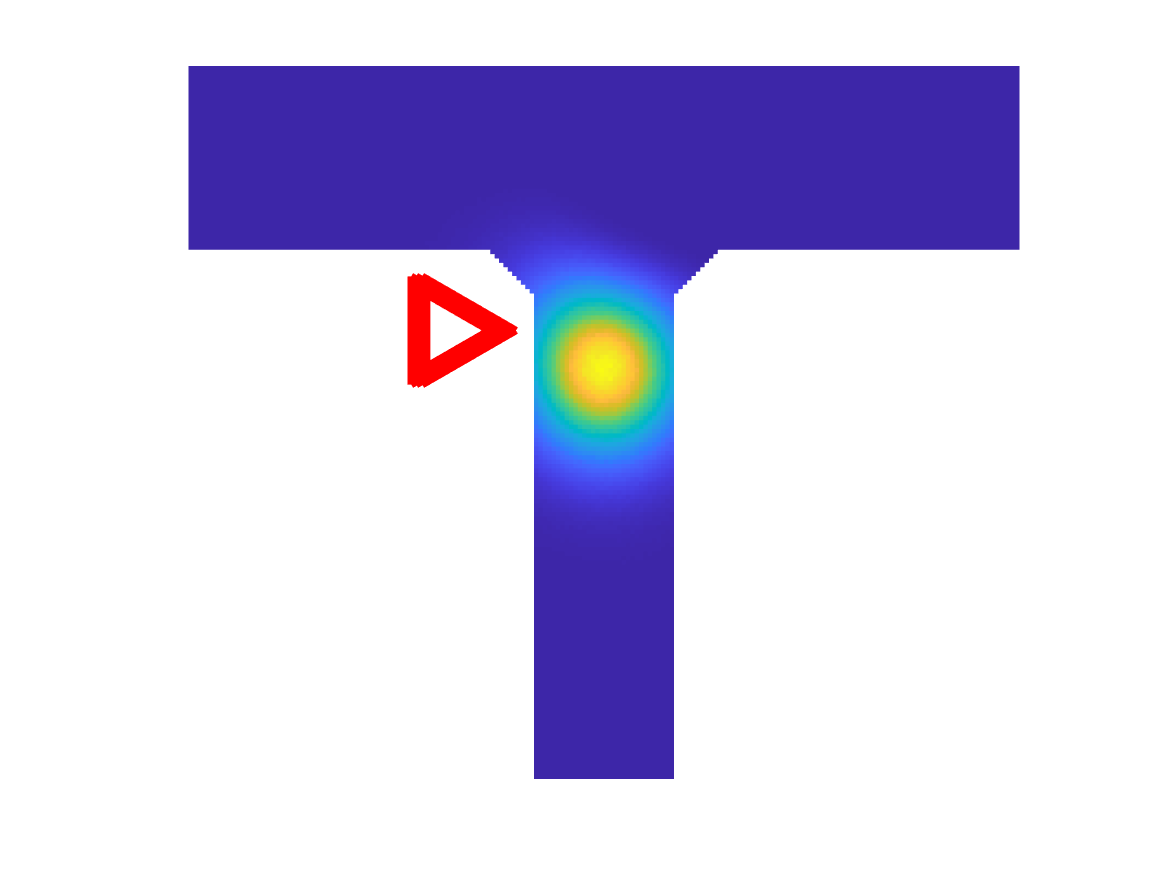

Supplement: Source code 1. [file elife-87055-code1.zip › code/fig5b_frames/154.bmp]

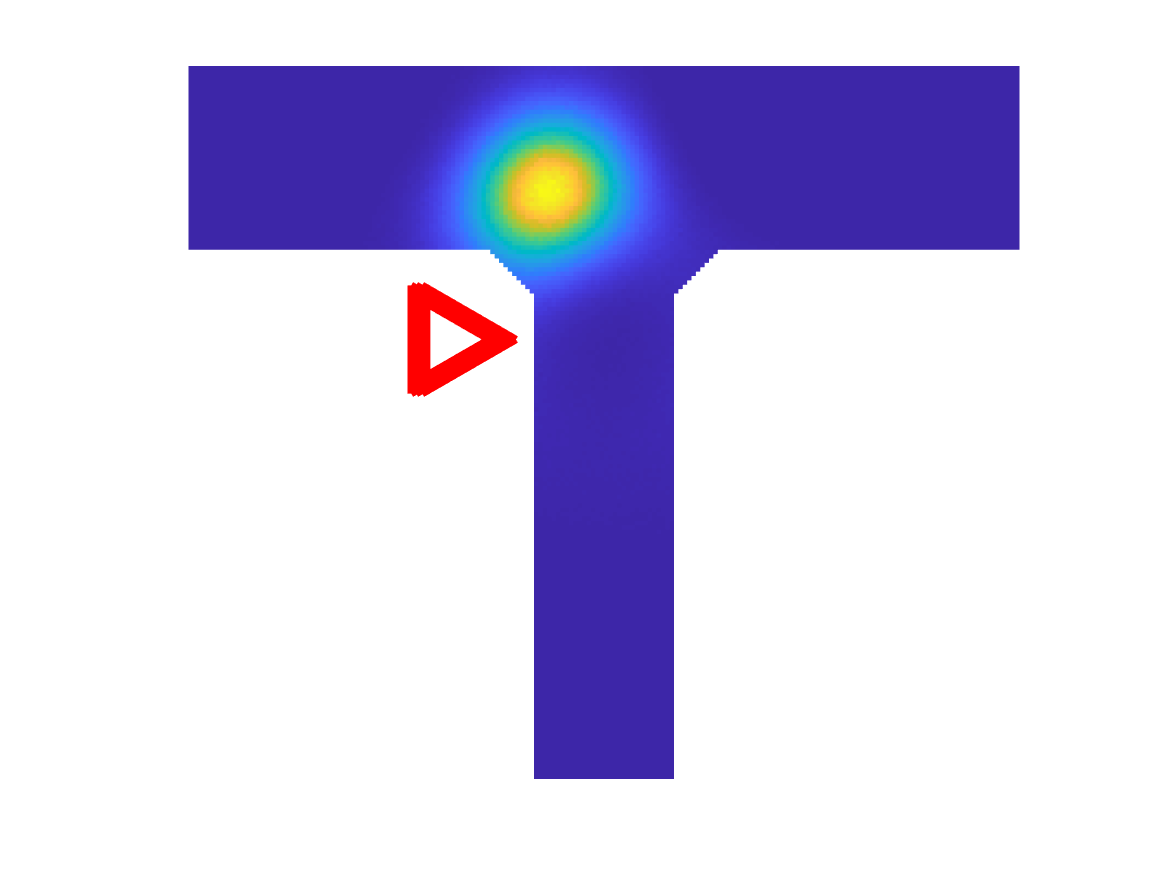

Supplement: Source code 1. [file elife-87055-code1.zip › code/fig5b_frames/140.bmp]

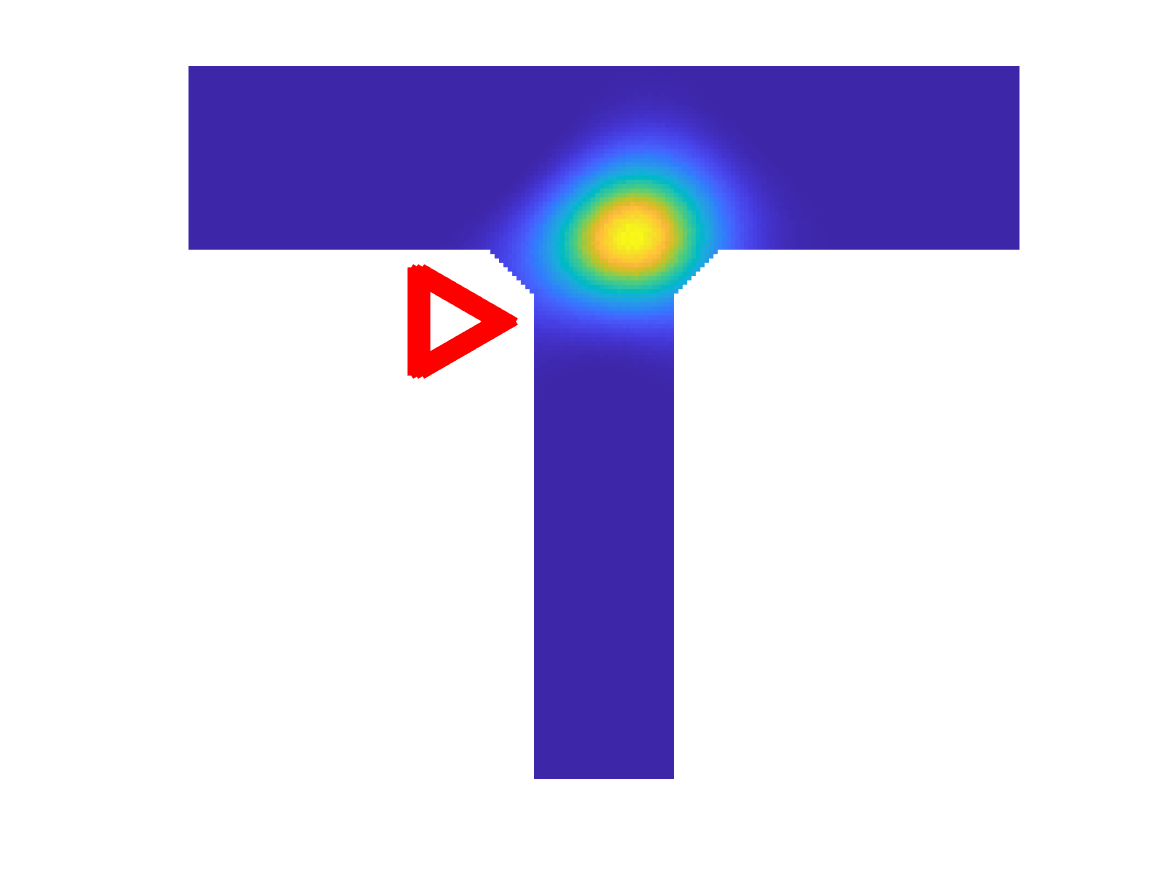

Supplement: Source code 1. [file elife-87055-code1.zip › code/fig5b_frames/168.bmp]

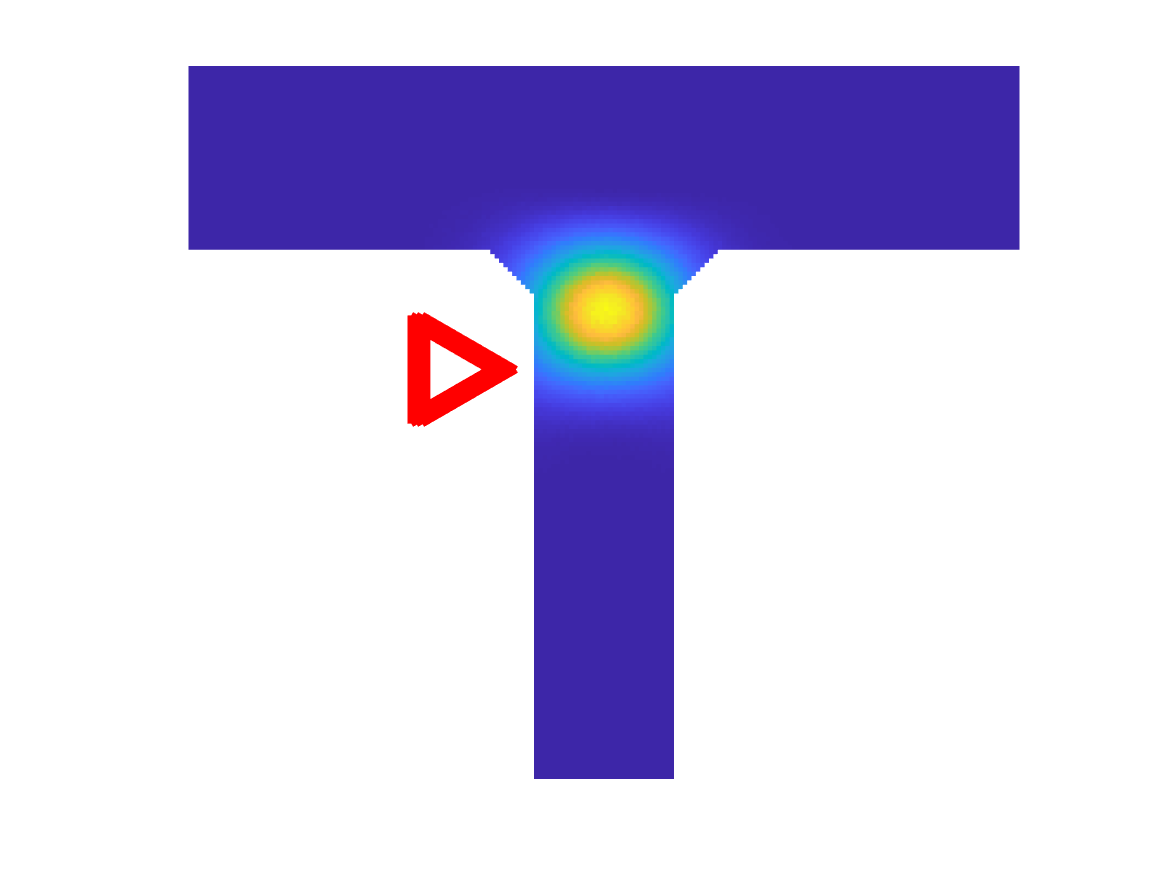

Supplement: Source code 1. [file elife-87055-code1.zip › code/fig5b_frames/96.bmp]

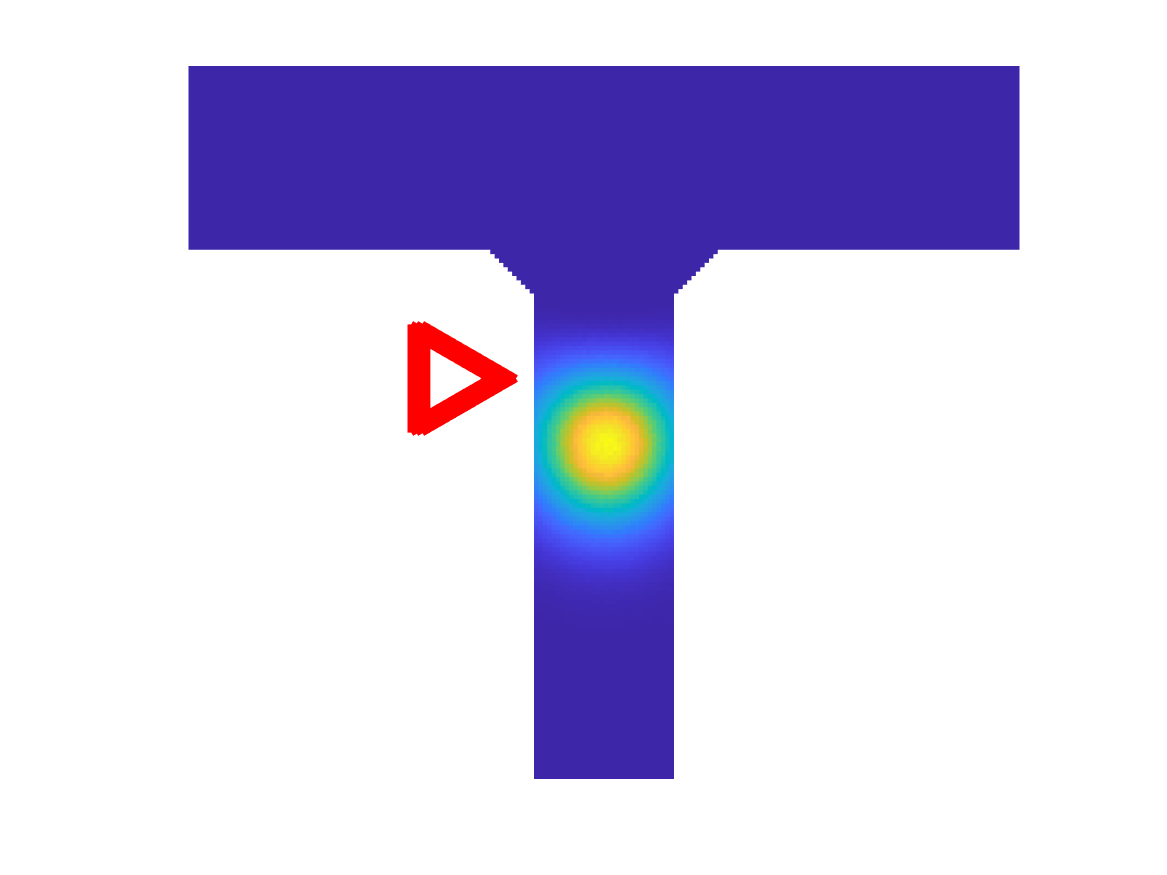

Supplement: Source code 1. [file elife-87055-code1.zip › code/fig5b_frames/82.bmp]

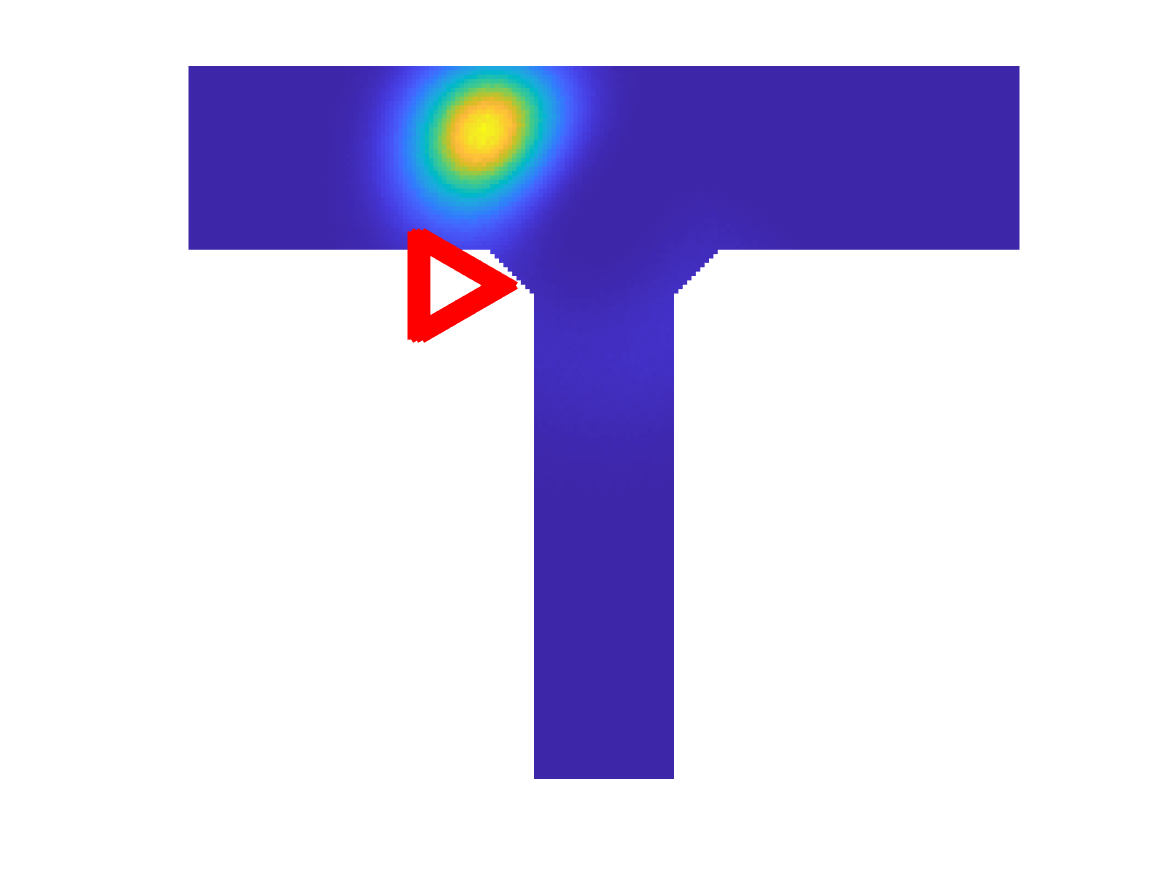

Supplement: Source code 1. [file elife-87055-code1.zip › code/fig5b_frames/222.bmp]

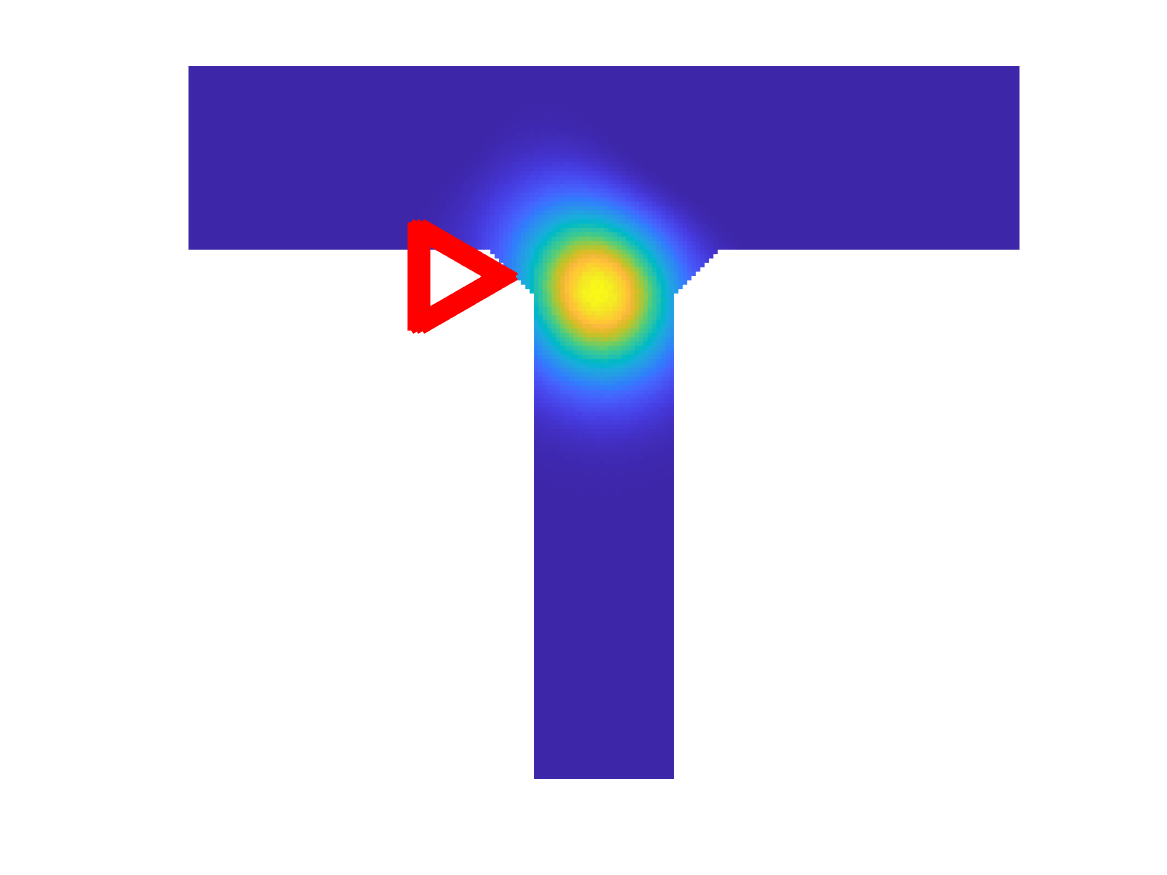

Supplement: Source code 1. [file elife-87055-code1.zip › code/fig5b_frames/236.bmp]

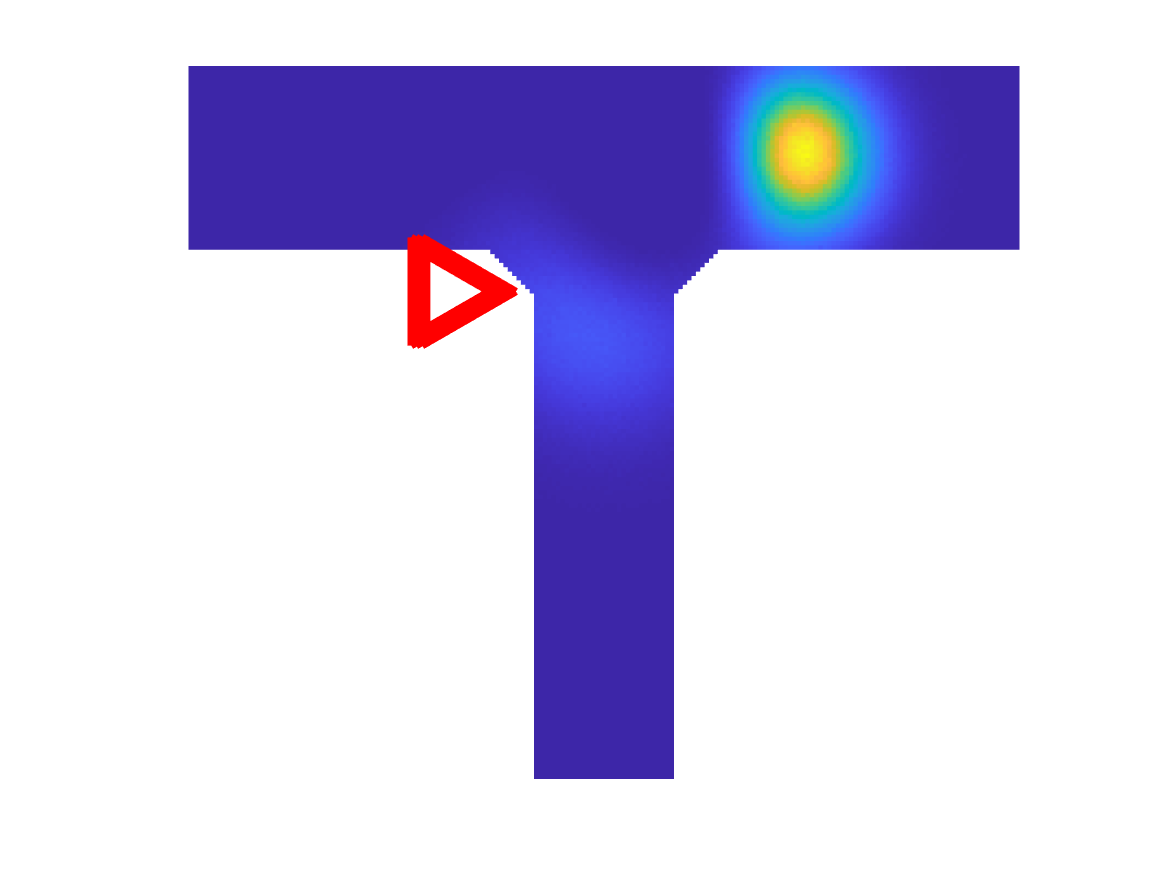

Supplement: Source code 1. [file elife-87055-code1.zip › code/fig5b_frames/213.bmp]

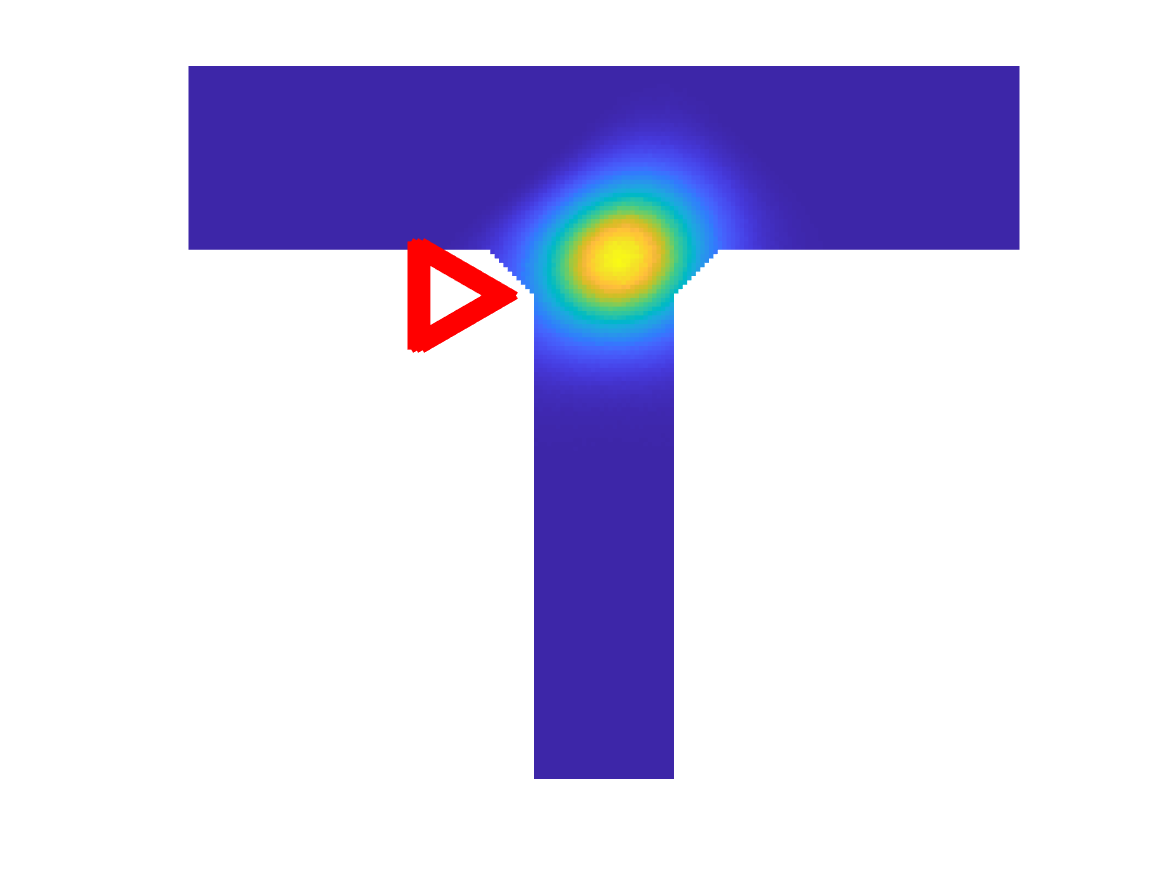

Supplement: Source code 1. [file elife-87055-code1.zip › code/fig5b_frames/207.bmp]

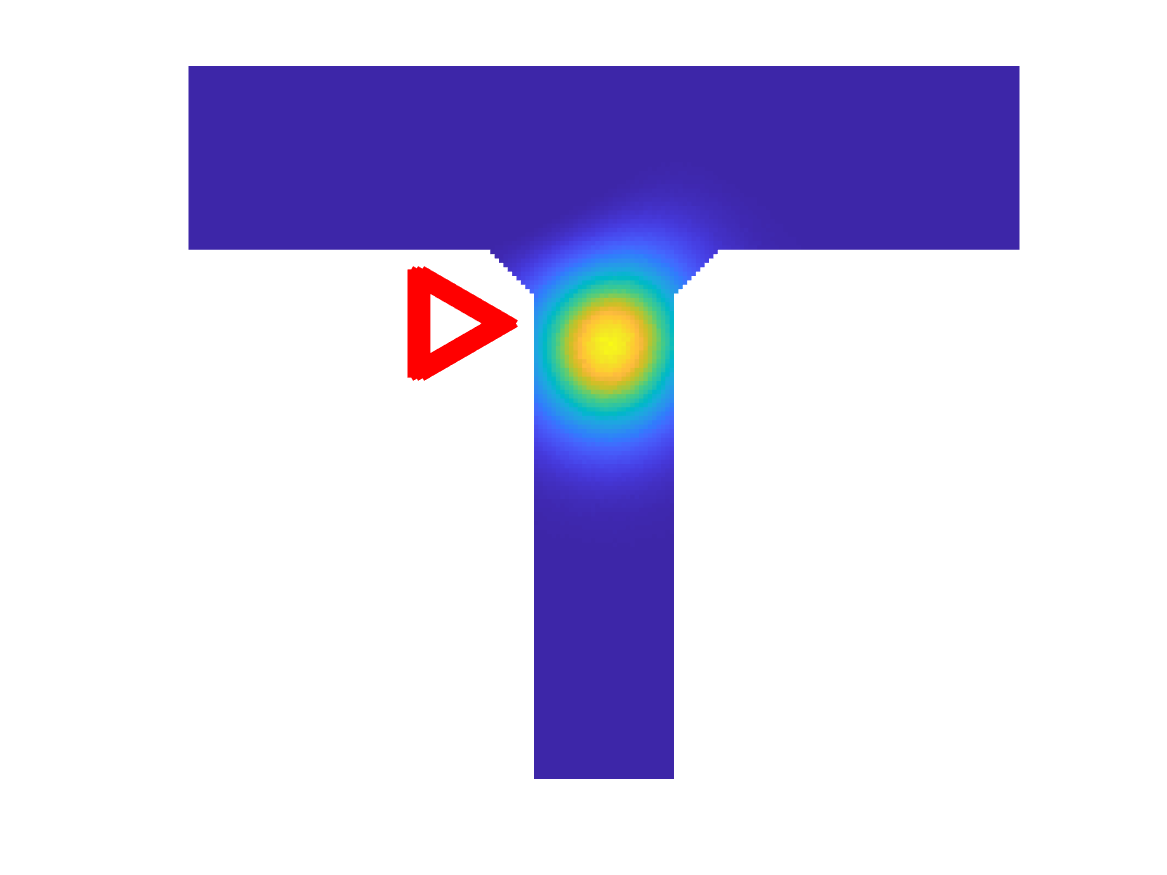

Supplement: Source code 1. [file elife-87055-code1.zip › code/fig5b_frames/165.bmp]

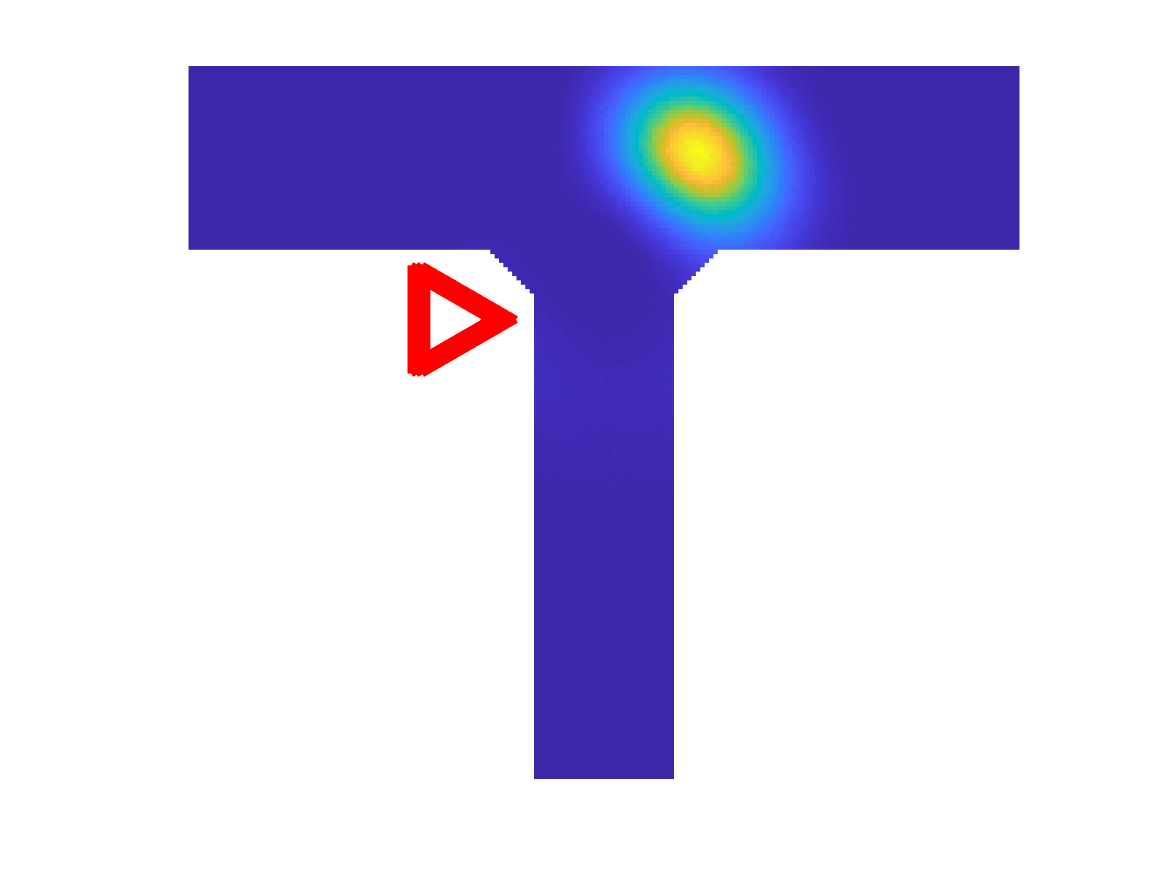

Supplement: Source code 1. [file elife-87055-code1.zip › code/fig5b_frames/171.bmp]

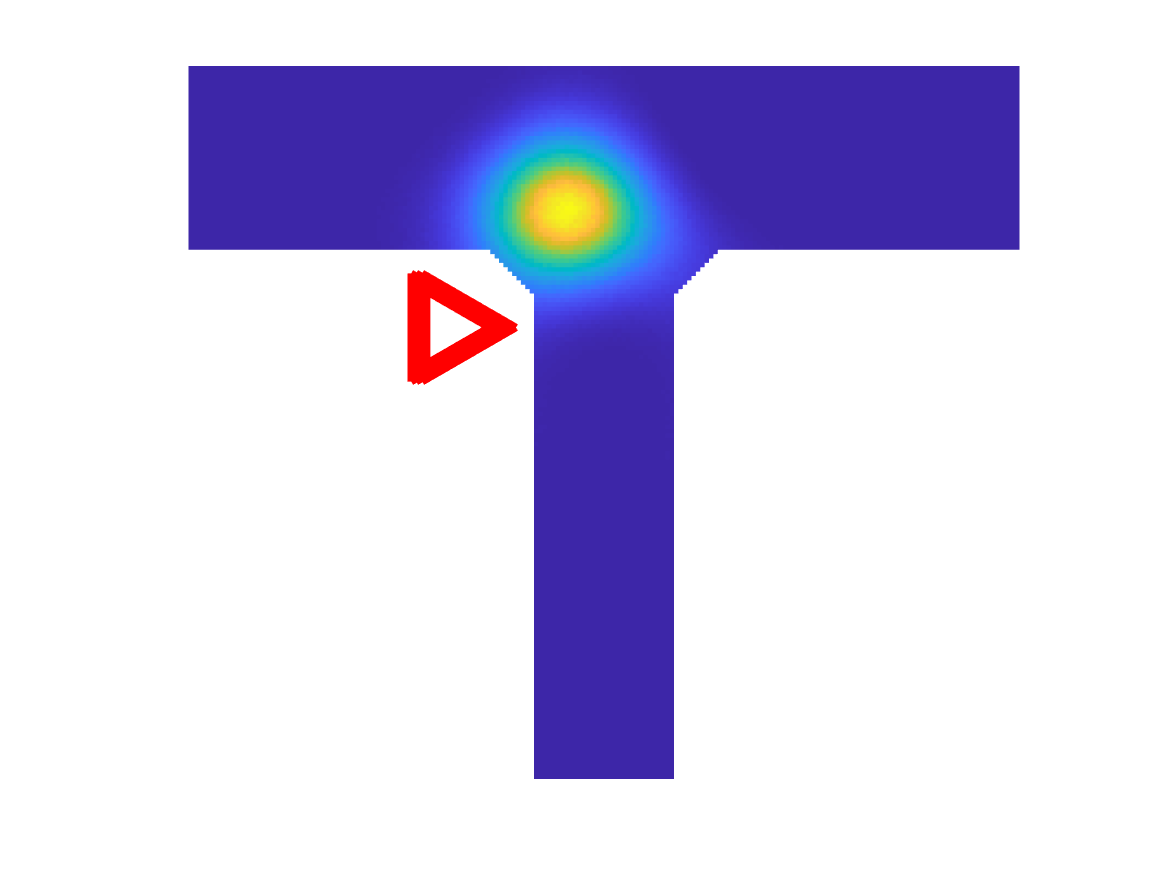

Supplement: Source code 1. [file elife-87055-code1.zip › code/fig5b_frames/159.bmp]

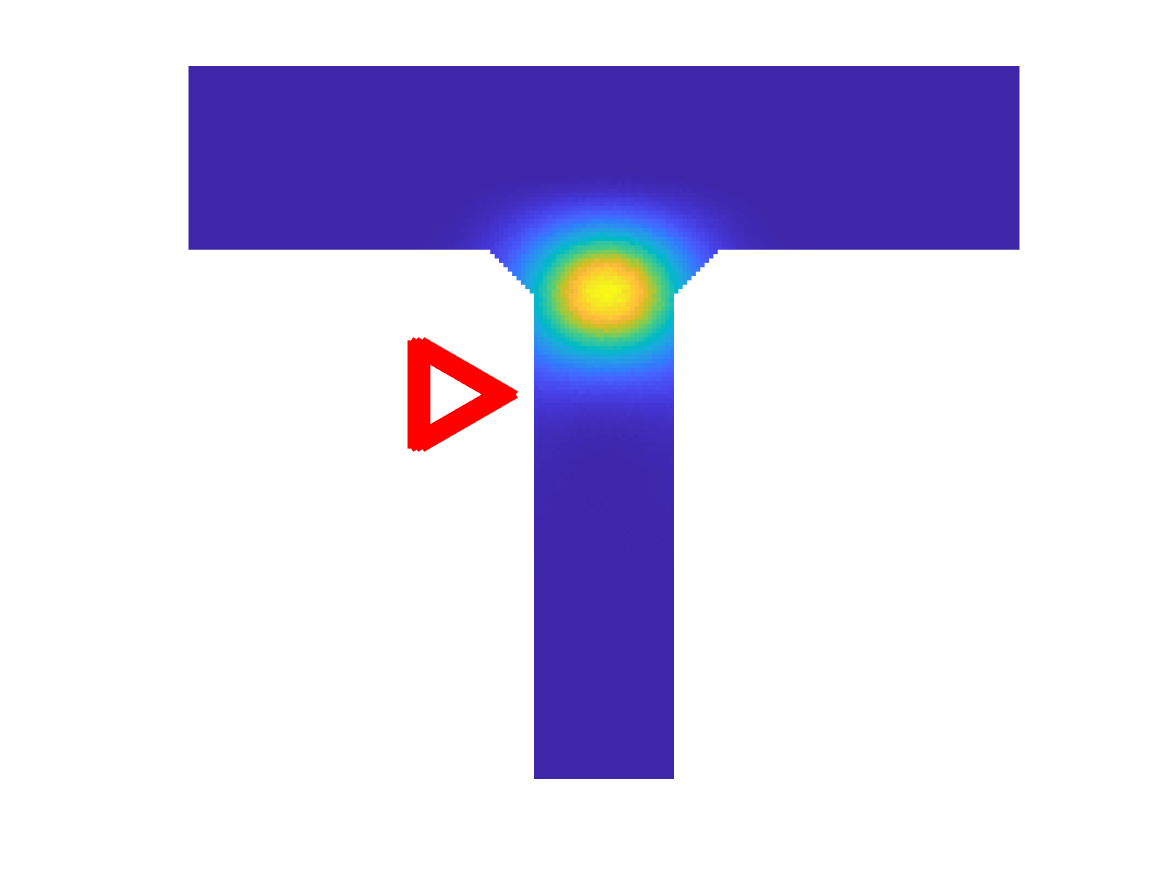

Supplement: Source code 1. [file elife-87055-code1.zip › code/fig5b_frames/58.bmp]

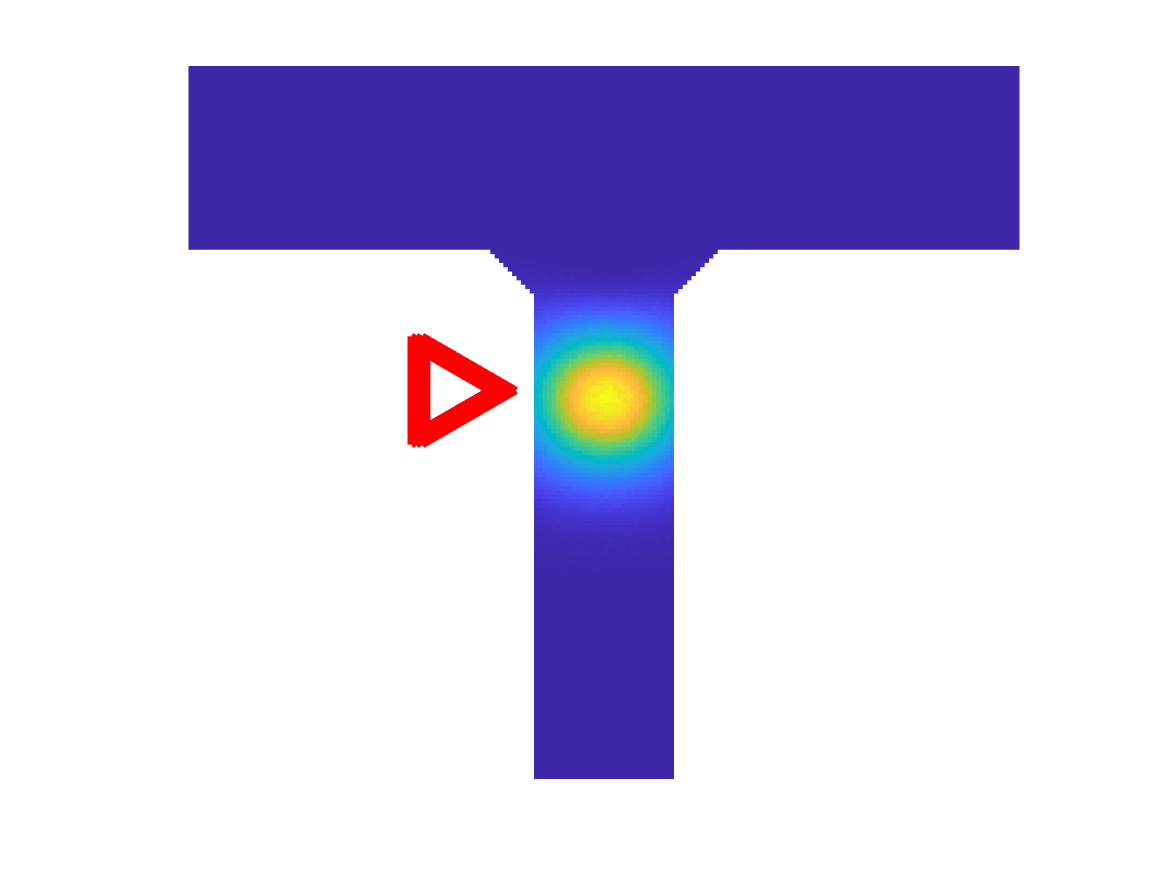

Supplement: Source code 1. [file elife-87055-code1.zip › code/fig5b_frames/64.bmp]

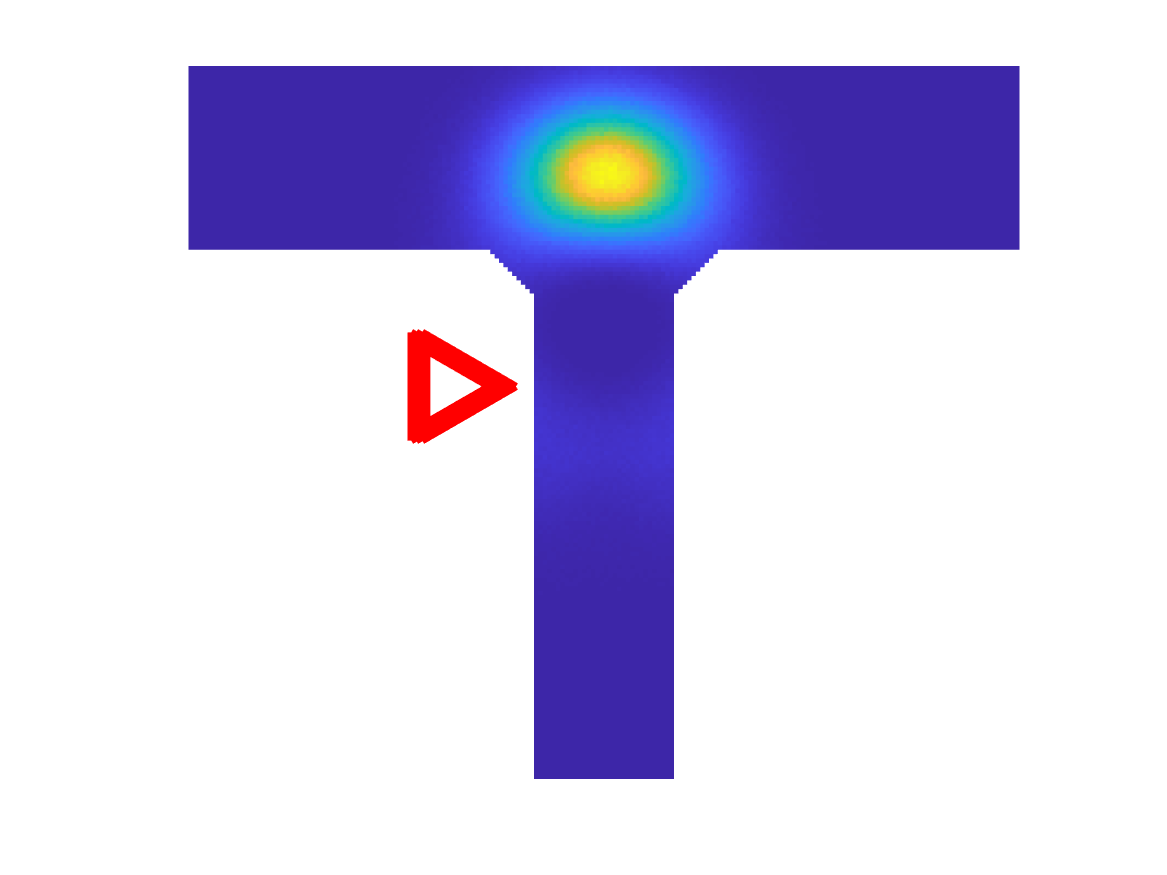

Supplement: Source code 1. [file elife-87055-code1.zip › code/fig5b_frames/70.bmp]

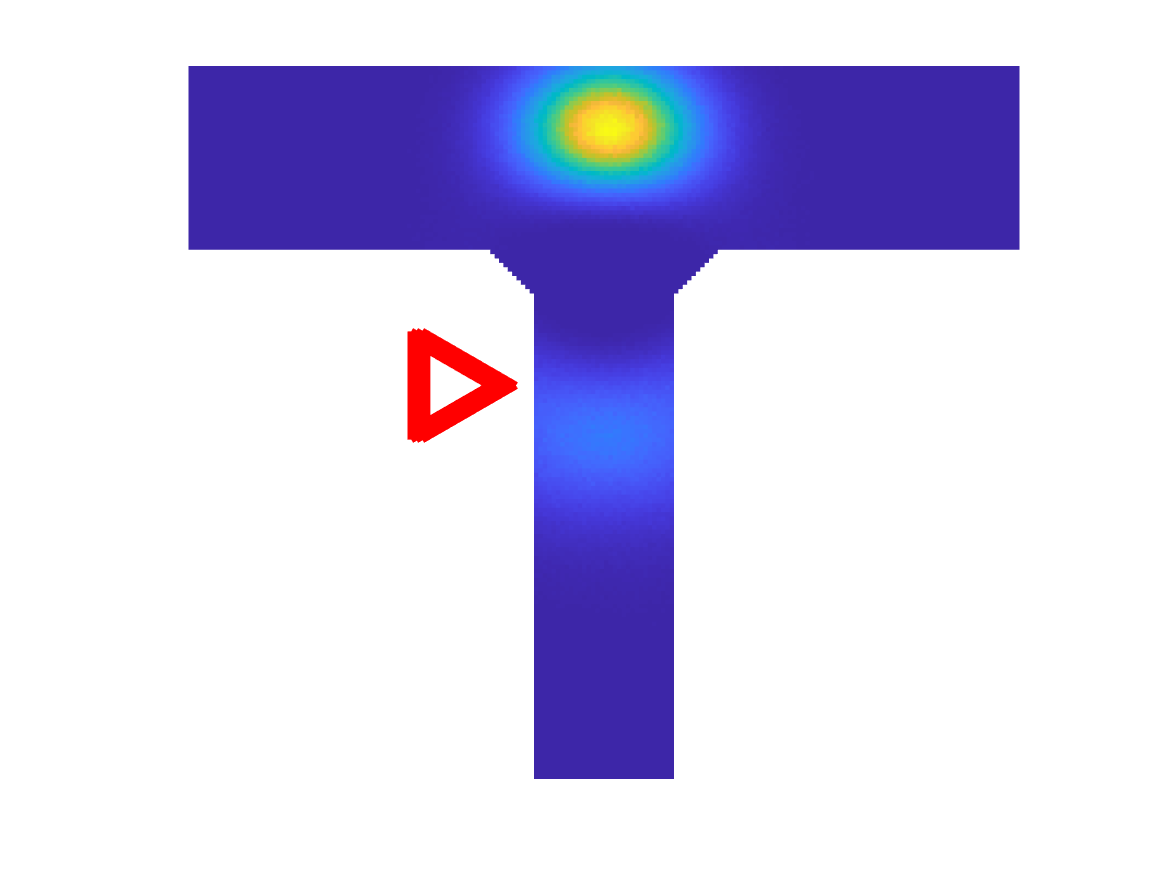

Supplement: Source code 1. [file elife-87055-code1.zip › code/fig5b_frames/71.bmp]

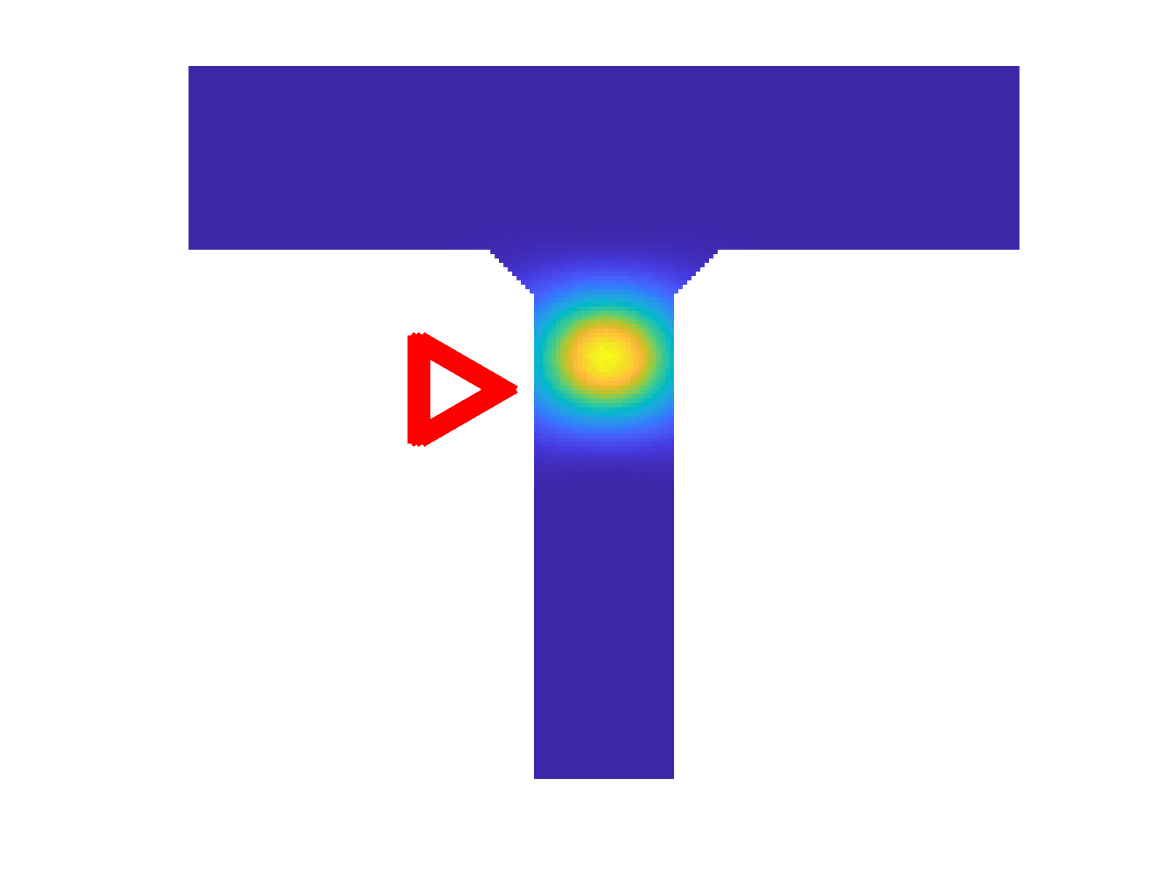

Supplement: Source code 1. [file elife-87055-code1.zip › code/fig5b_frames/65.bmp]

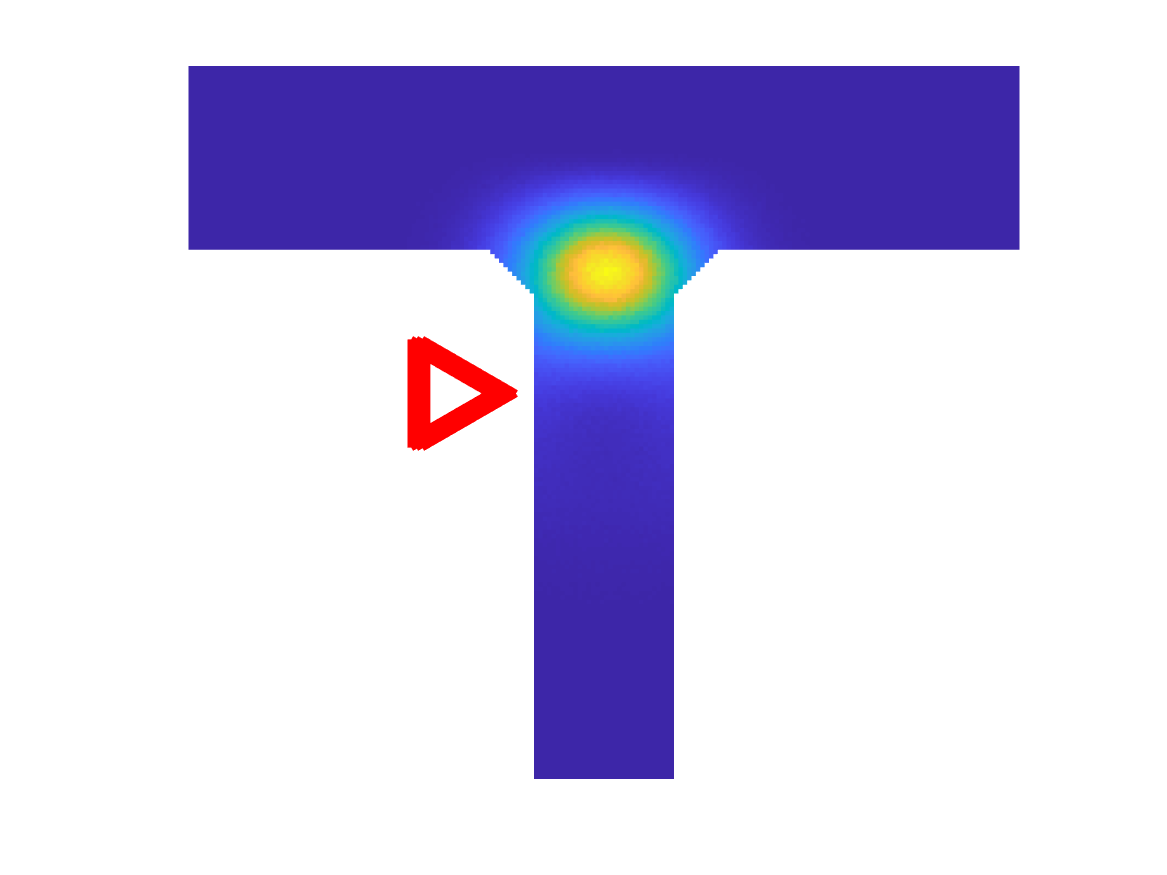

Supplement: Source code 1. [file elife-87055-code1.zip › code/fig5b_frames/59.bmp]

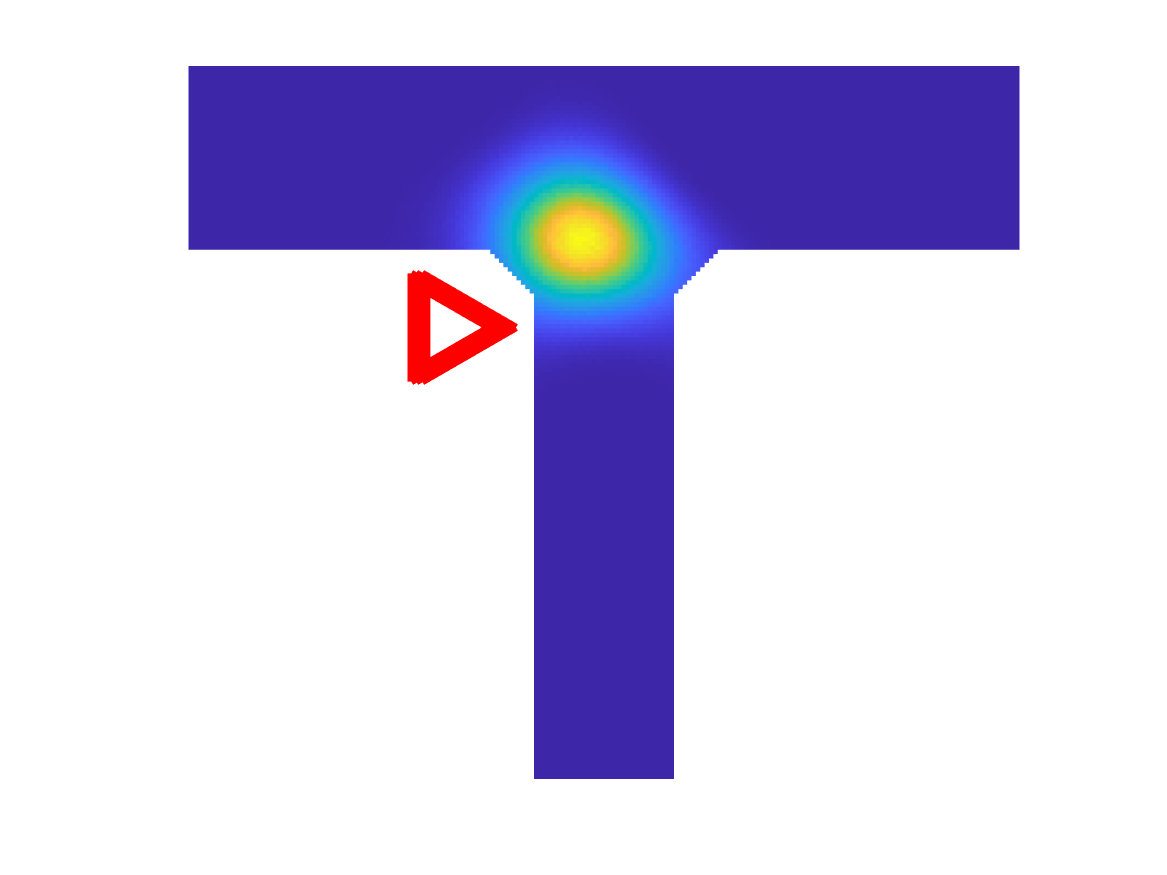

Supplement: Source code 1. [file elife-87055-code1.zip › code/fig5b_frames/158.bmp]

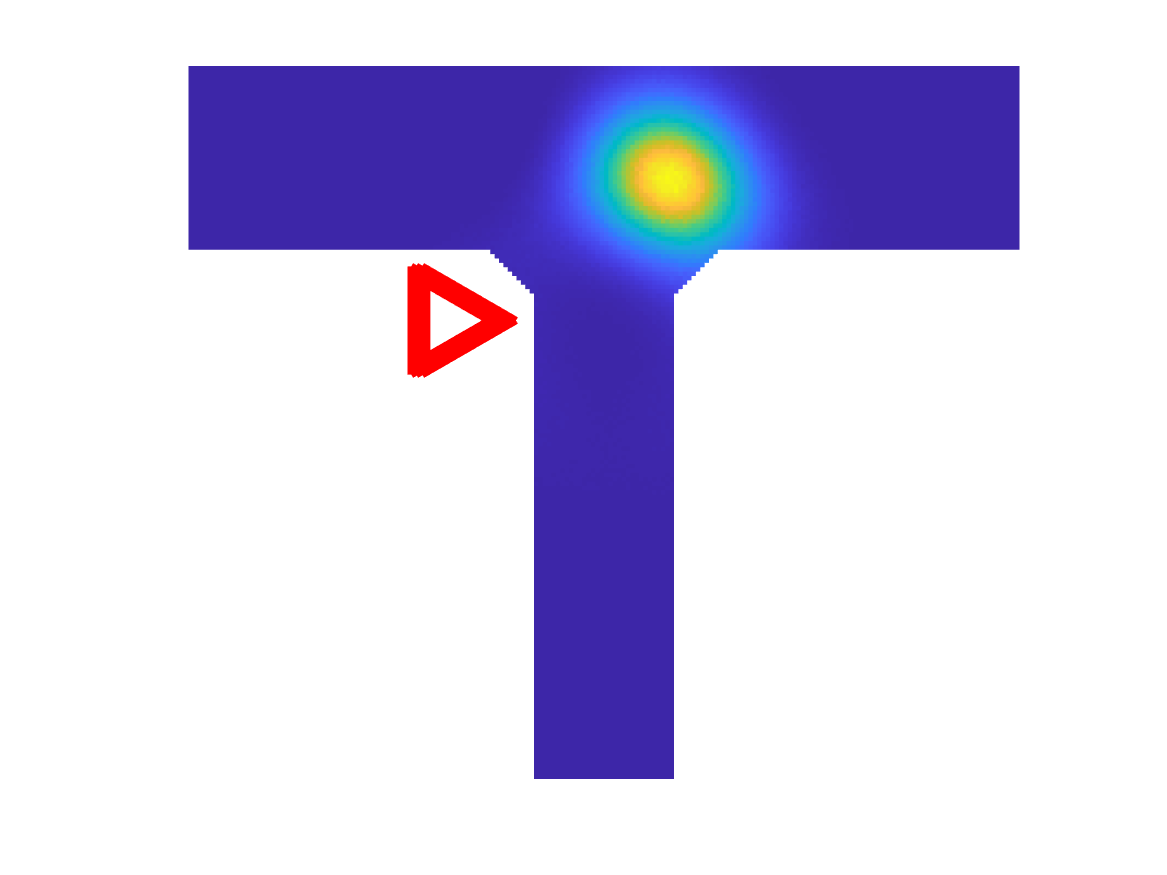

Supplement: Source code 1. [file elife-87055-code1.zip › code/fig5b_frames/170.bmp]

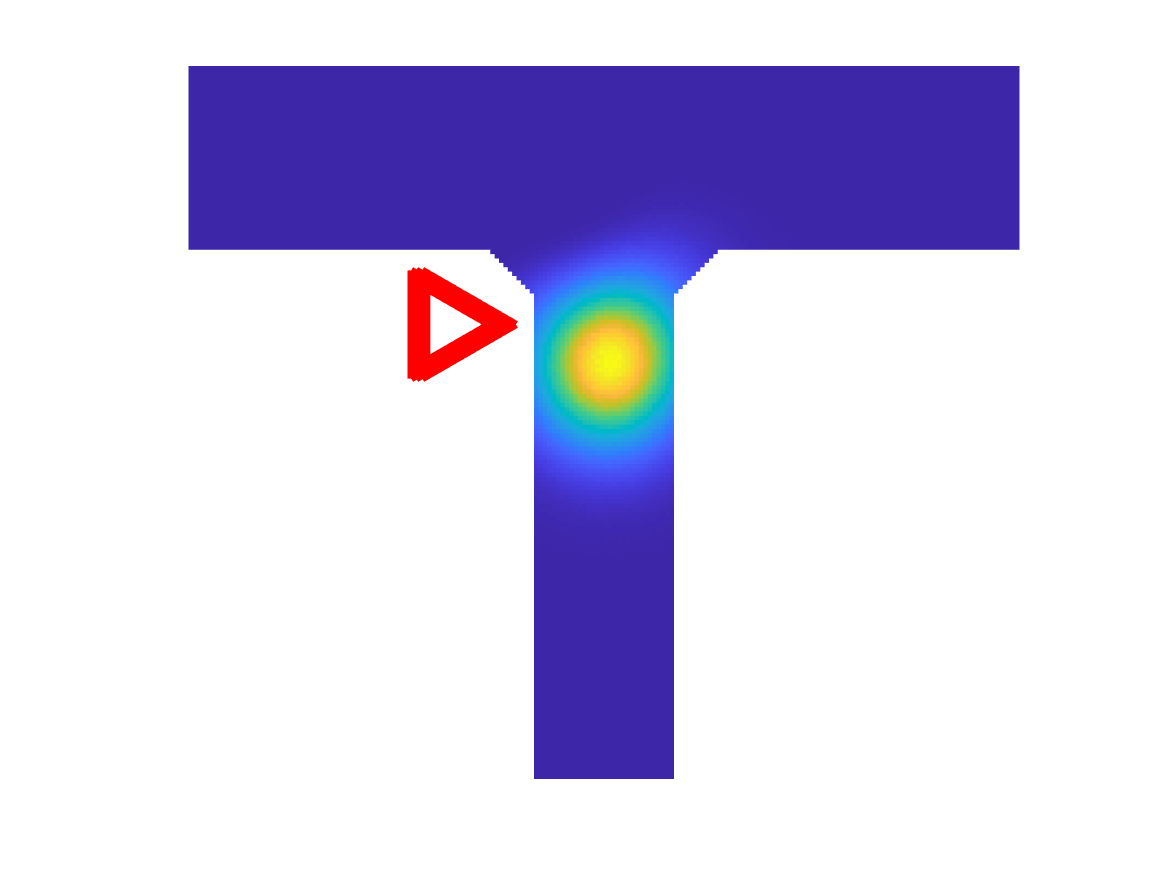

Supplement: Source code 1. [file elife-87055-code1.zip › code/fig5b_frames/164.bmp]

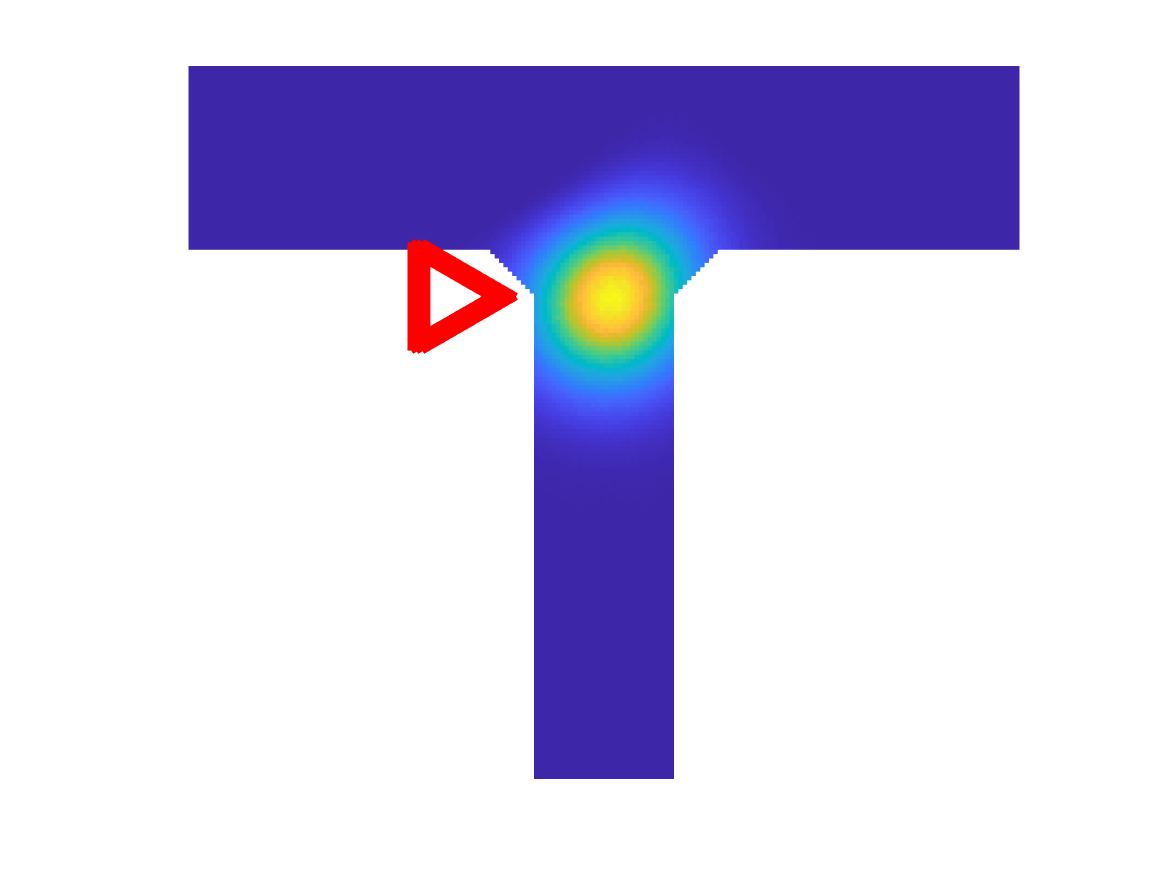

Supplement: Source code 1. [file elife-87055-code1.zip › code/fig5b_frames/206.bmp]

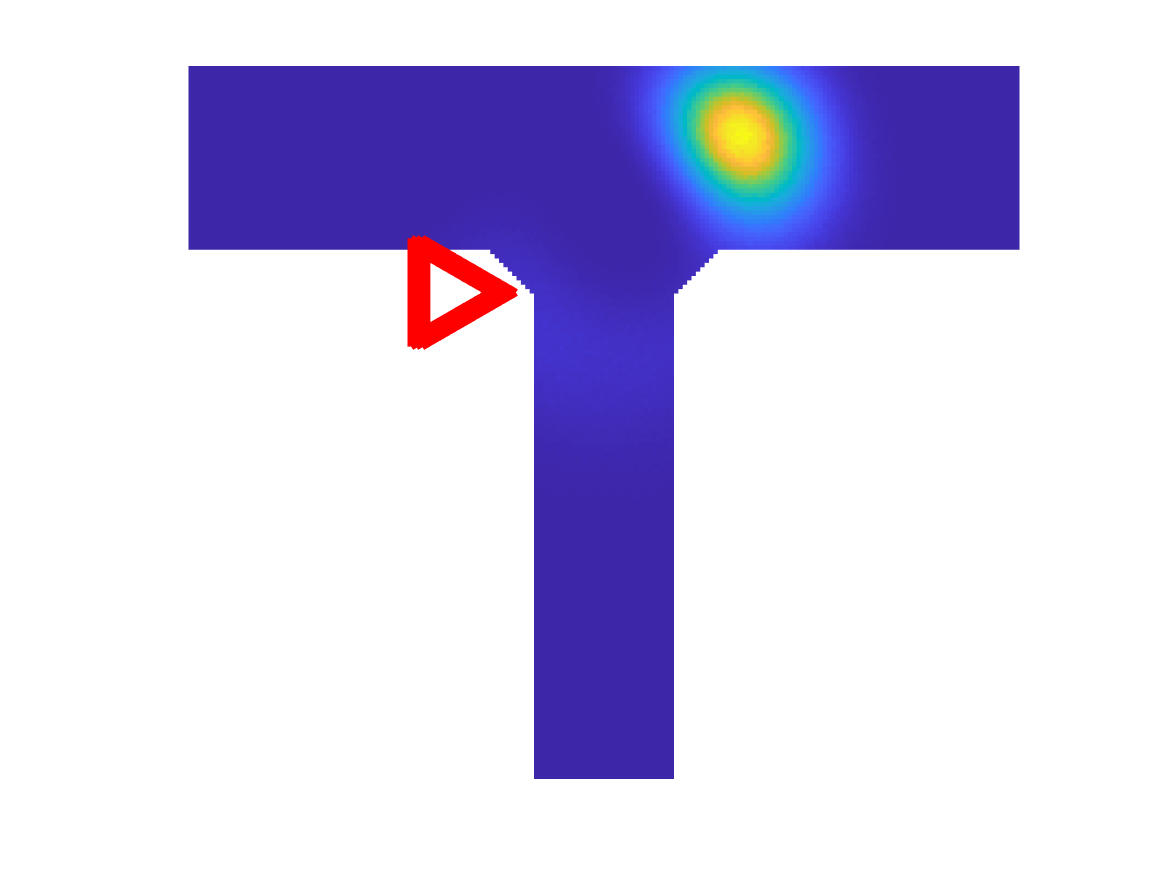

Supplement: Source code 1. [file elife-87055-code1.zip › code/fig5b_frames/212.bmp]

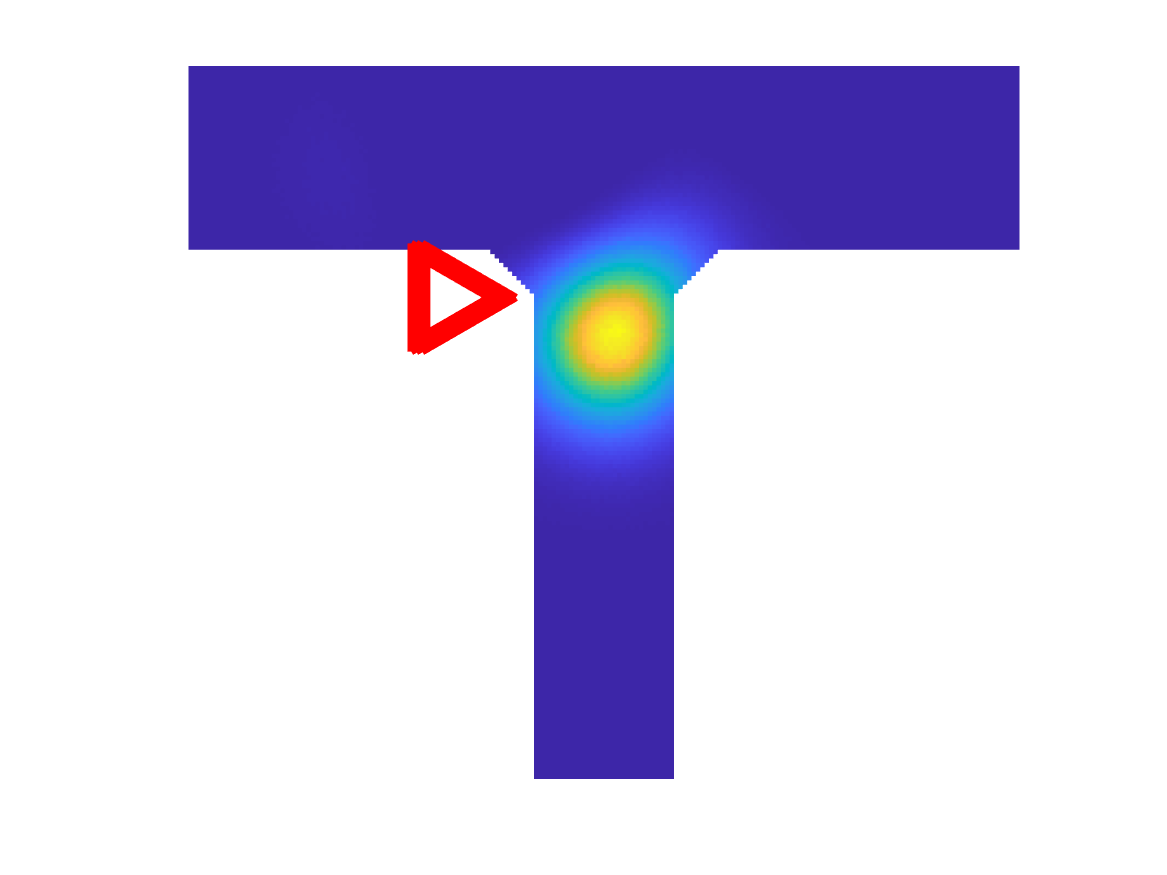

Supplement: Source code 1. [file elife-87055-code1.zip › code/fig5b_frames/204.bmp]

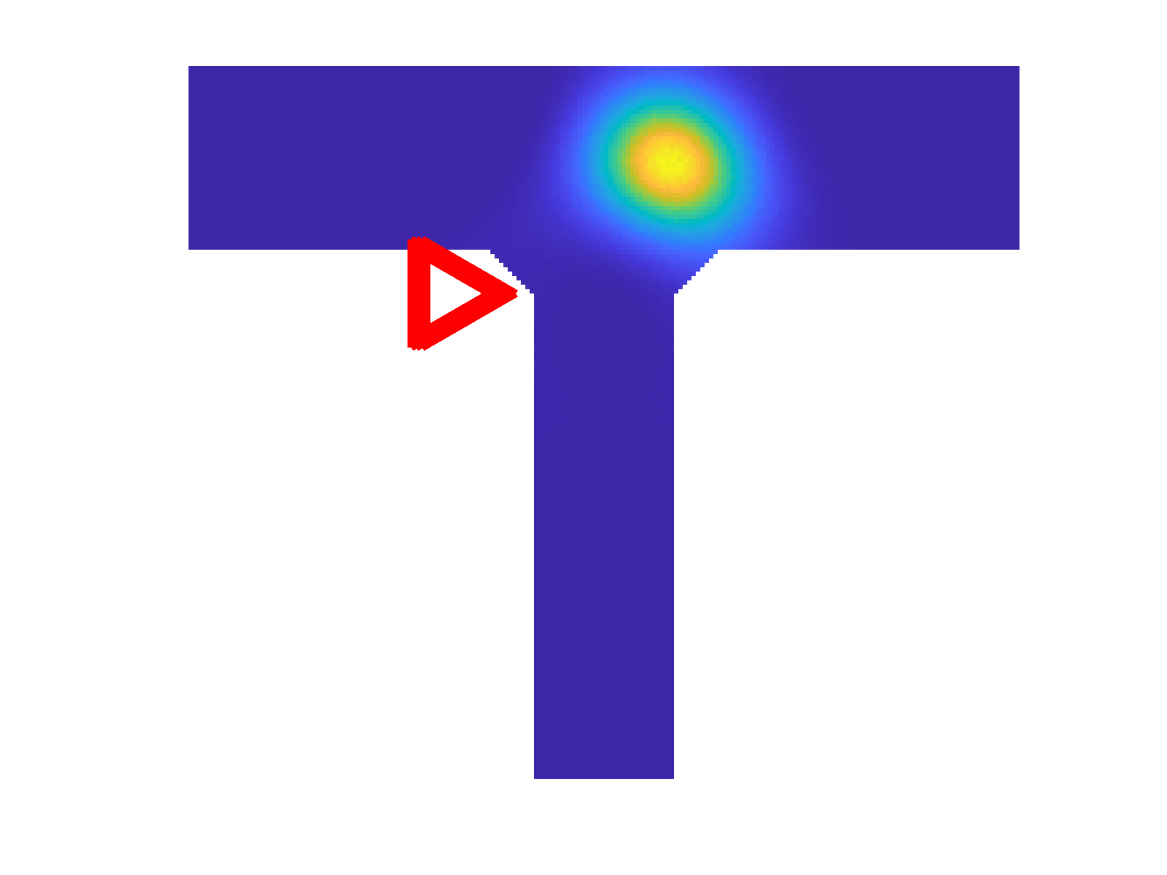

Supplement: Source code 1. [file elife-87055-code1.zip › code/fig5b_frames/210.bmp]

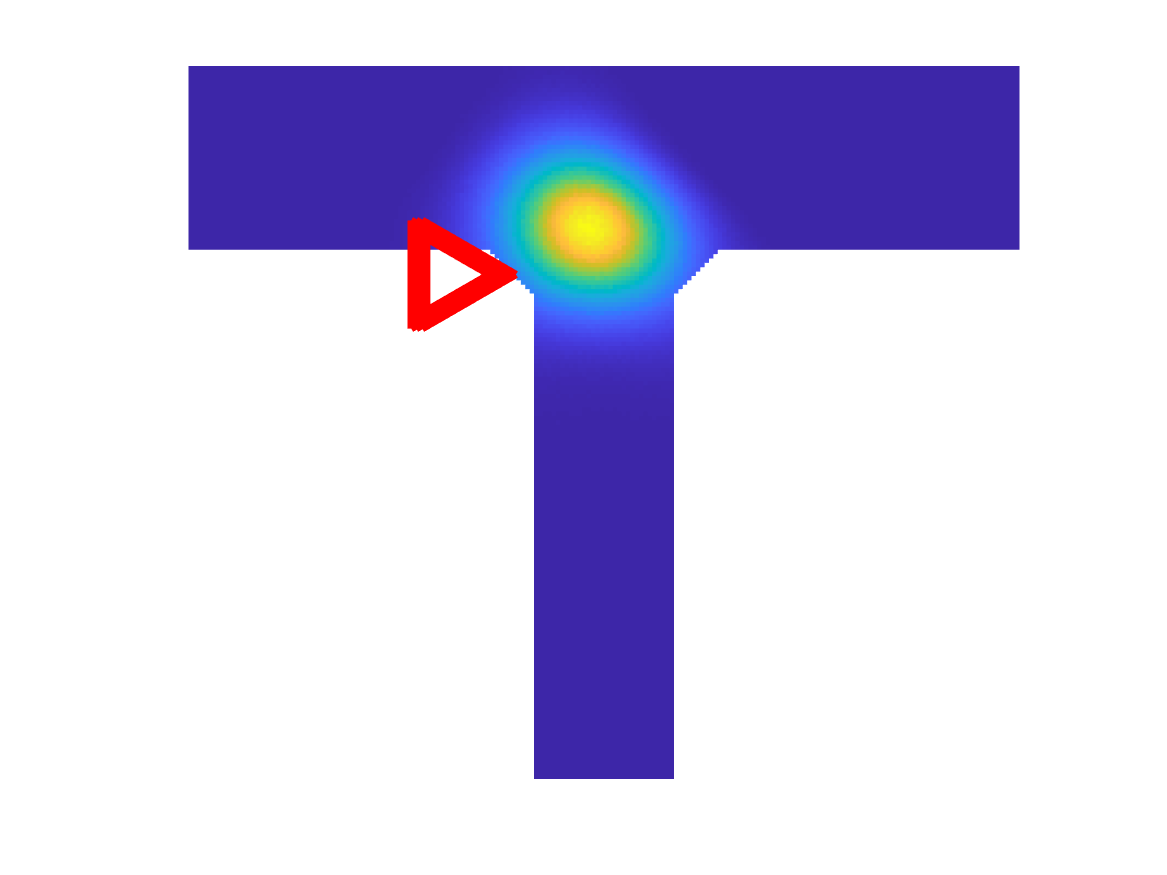

Supplement: Source code 1. [file elife-87055-code1.zip › code/fig5b_frames/238.bmp]

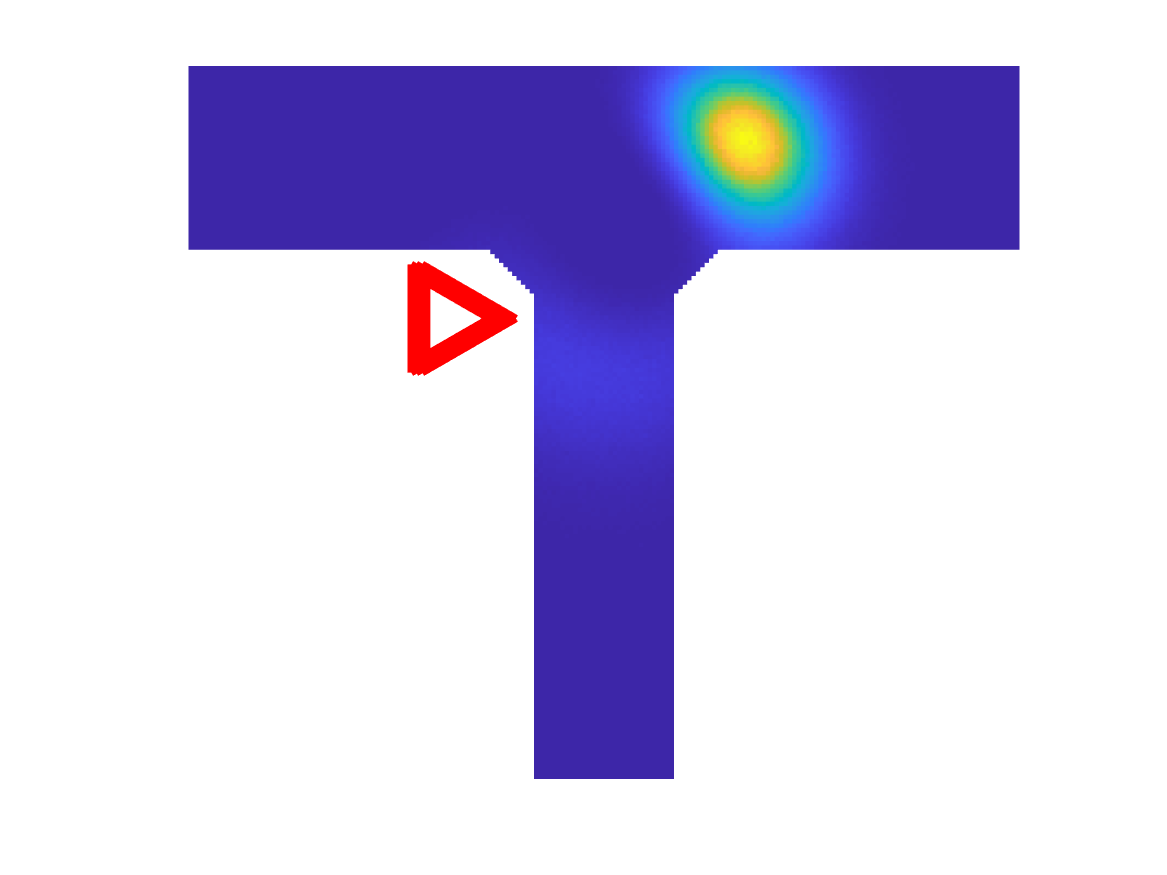

Supplement: Source code 1. [file elife-87055-code1.zip › code/fig5b_frames/172.bmp]

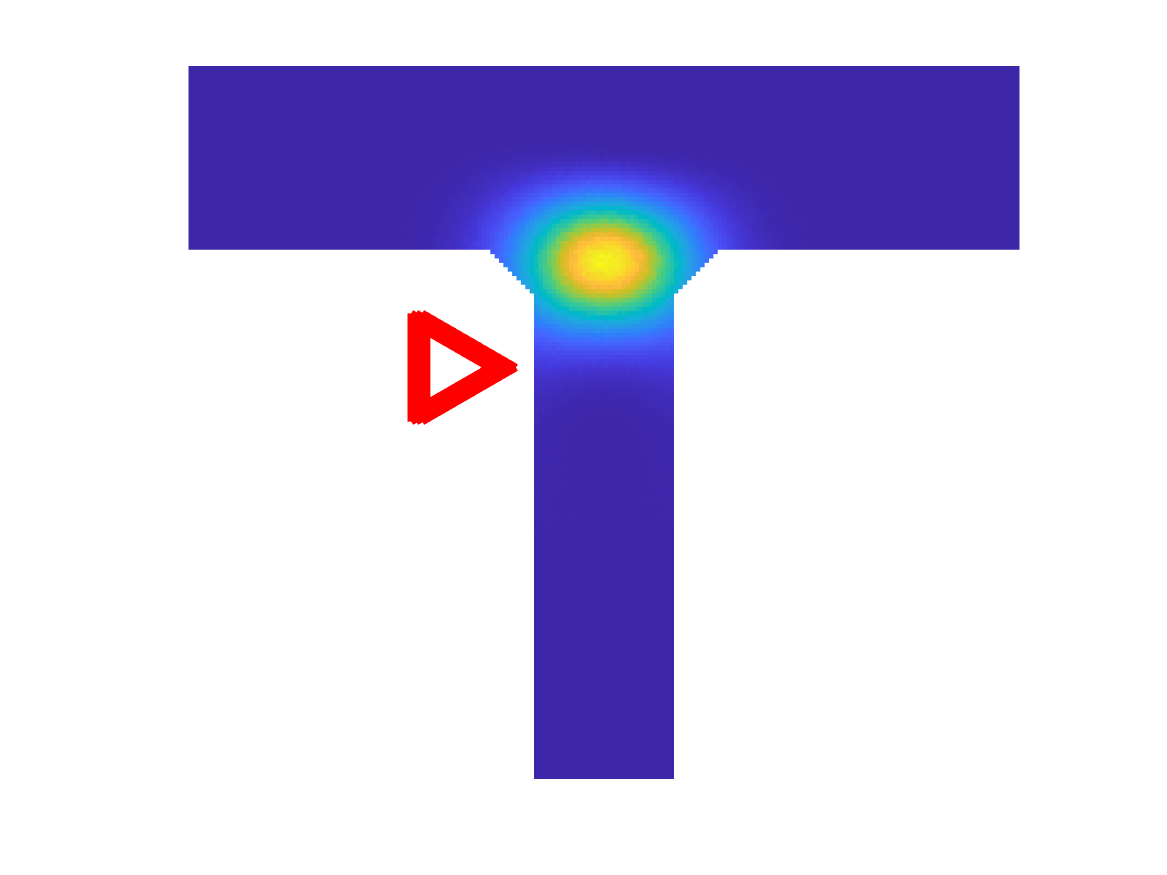

Supplement: Source code 1. [file elife-87055-code1.zip › code/fig5b_frames/98.bmp]

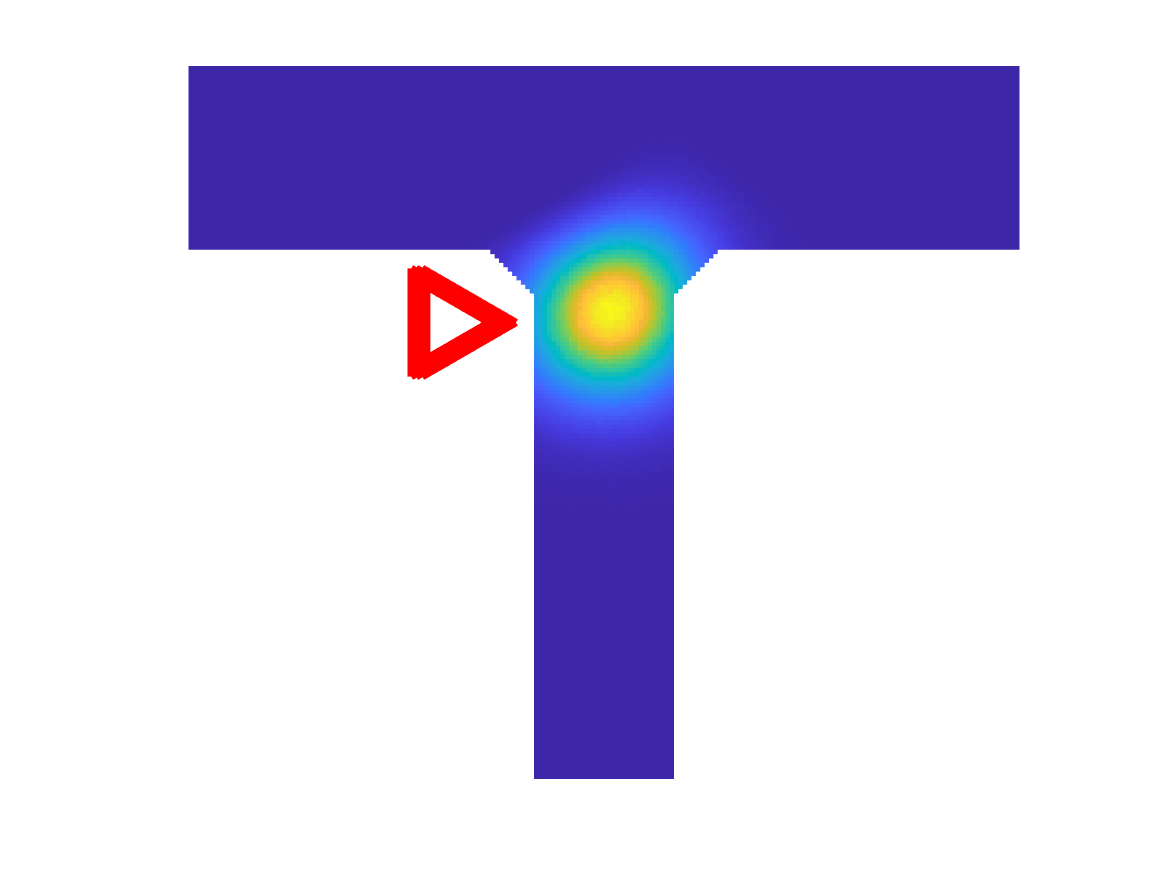

Supplement: Source code 1. [file elife-87055-code1.zip › code/fig5b_frames/166.bmp]

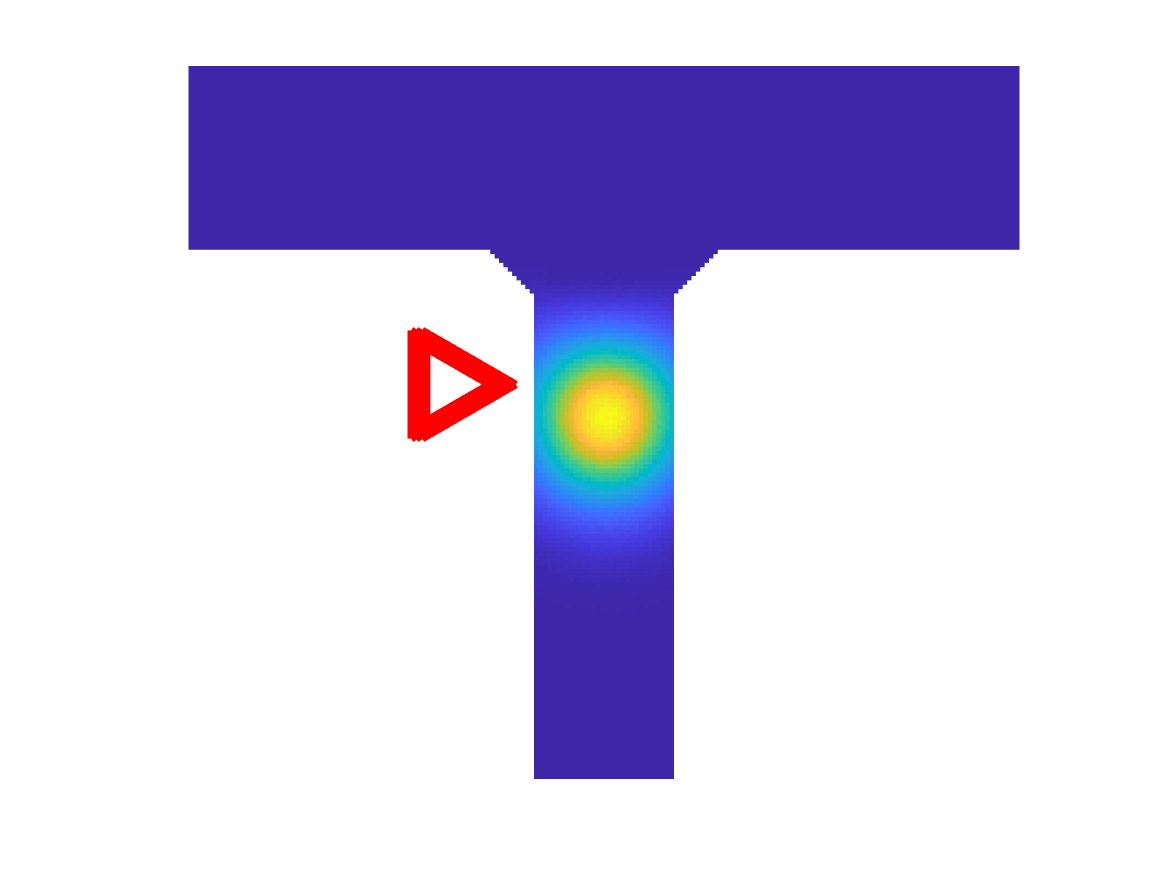

Supplement: Source code 1. [file elife-87055-code1.zip › code/fig5b_frames/73.bmp]

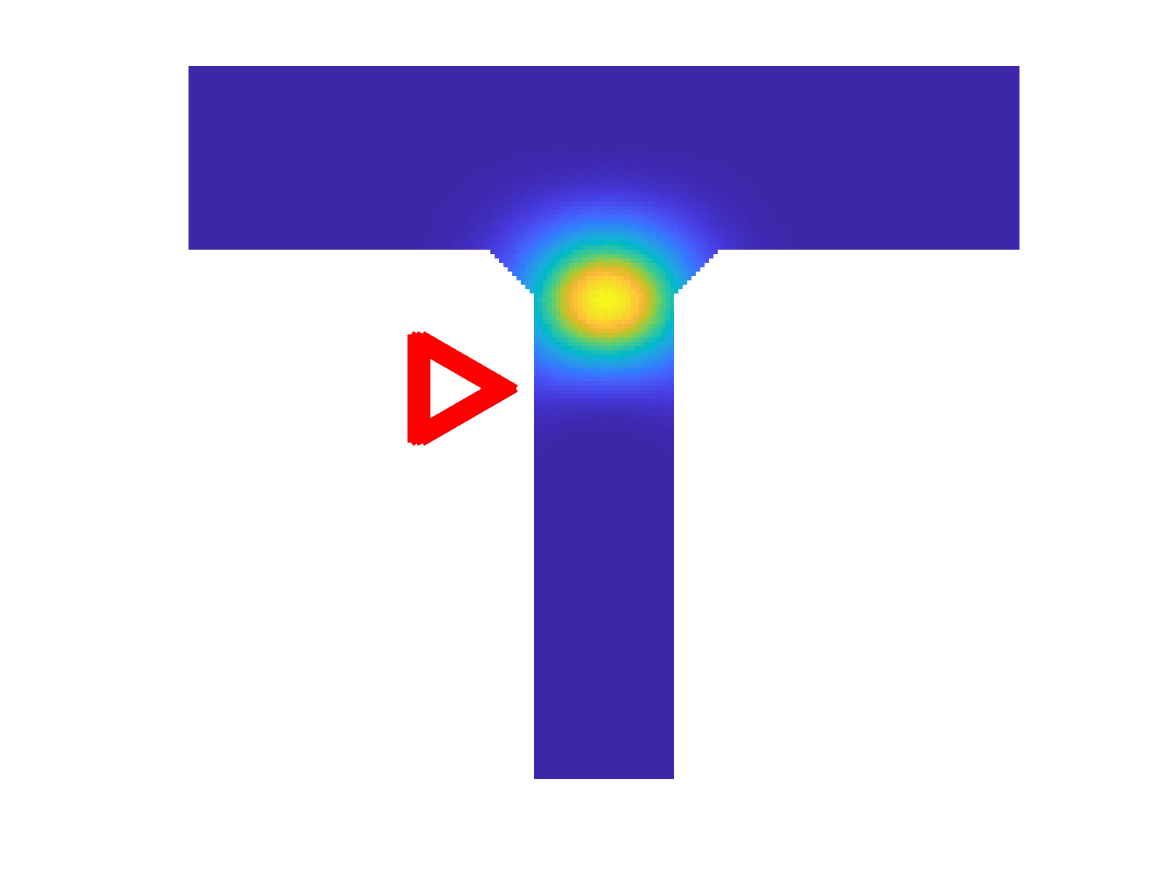

Supplement: Source code 1. [file elife-87055-code1.zip › code/fig5b_frames/67.bmp]

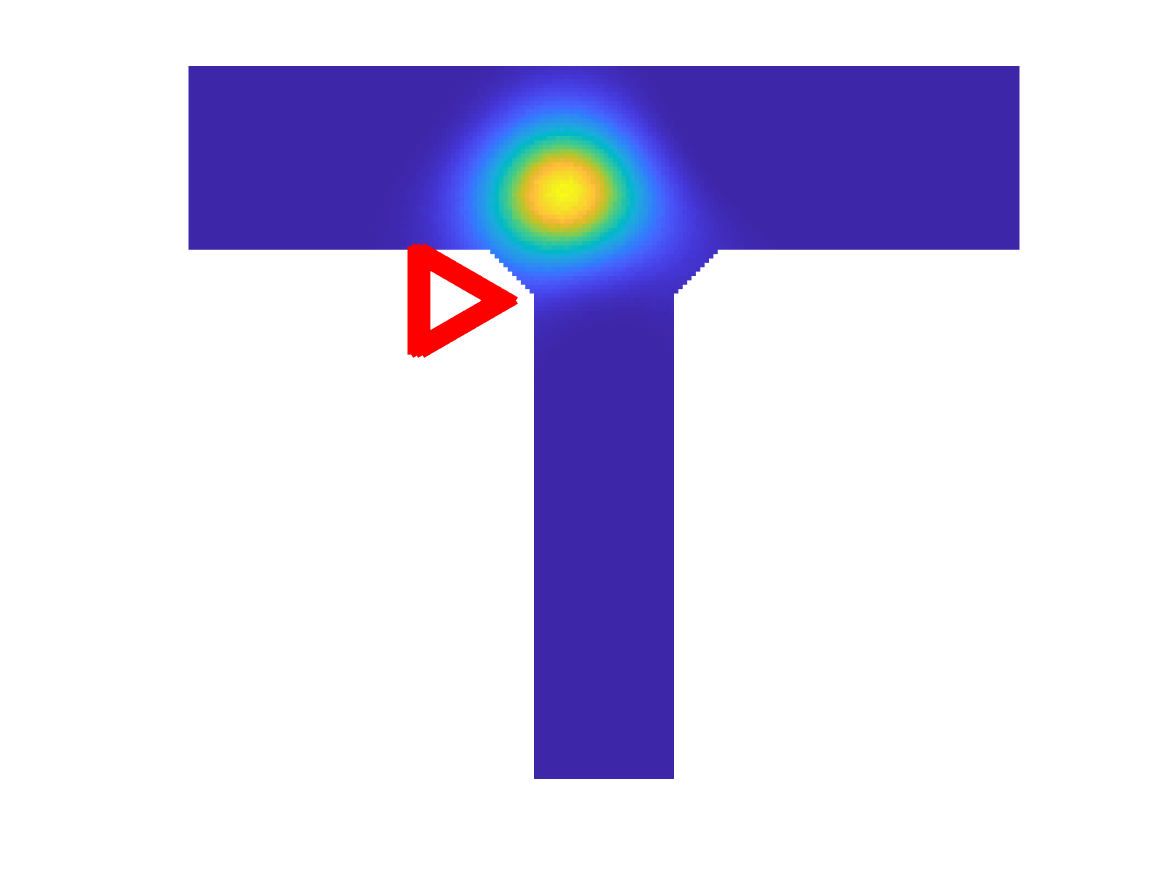

Supplement: Source code 1. [file elife-87055-code1.zip › code/fig5b_frames/199.bmp]

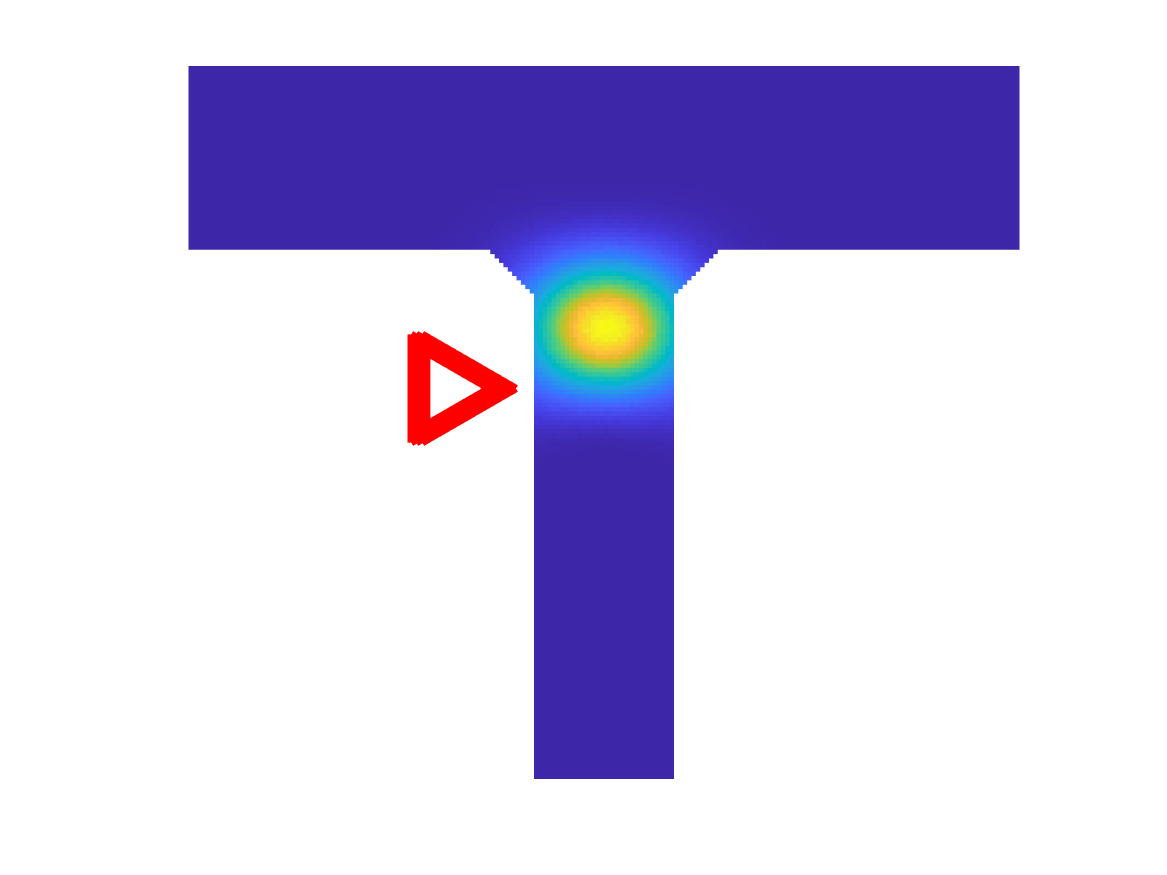

Supplement: Source code 1. [file elife-87055-code1.zip › code/fig5b_frames/66.bmp]

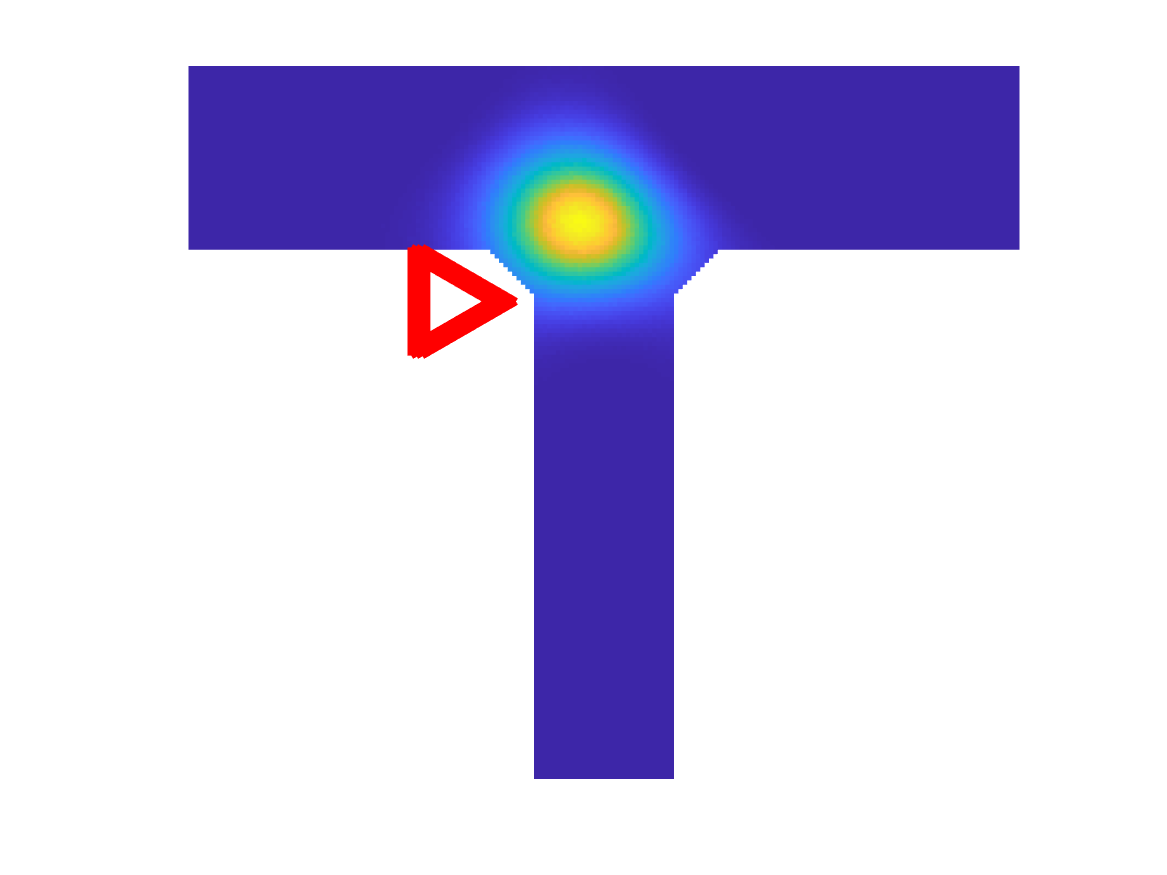

Supplement: Source code 1. [file elife-87055-code1.zip › code/fig5b_frames/198.bmp]

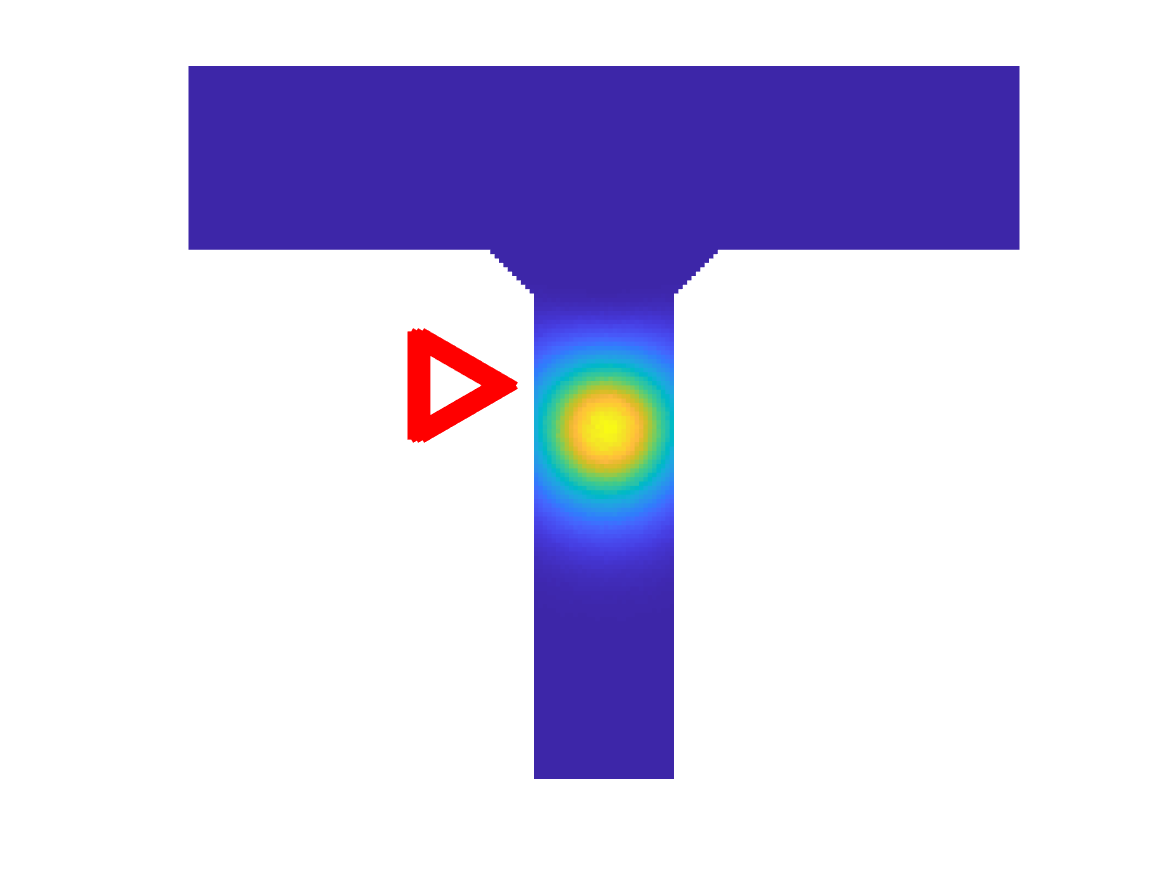

Supplement: Source code 1. [file elife-87055-code1.zip › code/fig5b_frames/72.bmp]
