# Supplementary figures and images for: Firing rate adaptation affords place cell theta sweeps, phase precession, and procession (part 1 of 2)
Source: eLife. 2024 Jul 22;12:RP87055. doi: 10.7554/eLife.87055 (PMC11262797; doi:10.7554/eLife.87055)

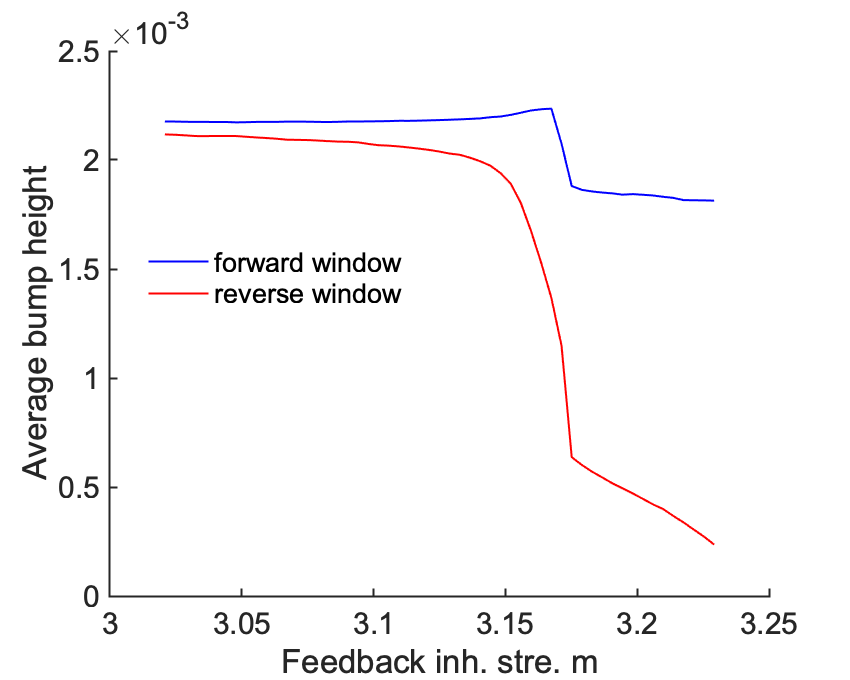

Supplement: Source code 1. [file elife-87055-code1.zip › code/fig4c.bmp]

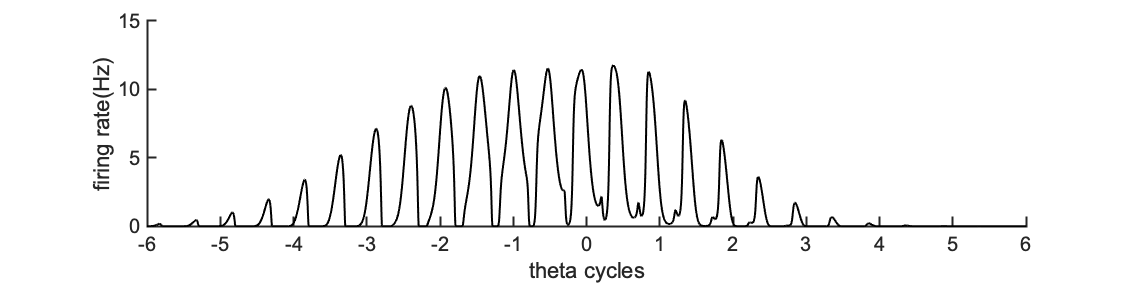

Supplement: Source code 1. [file elife-87055-code1.zip › code/fig4b.bmp]

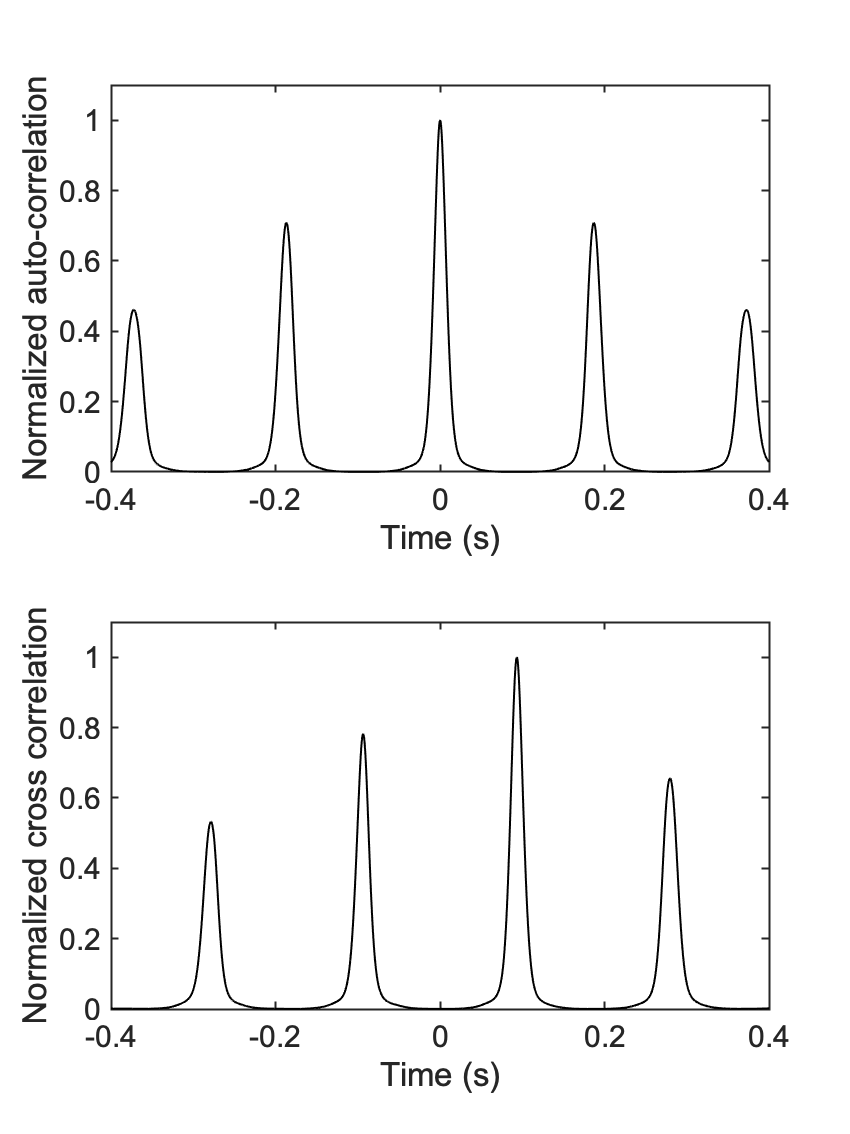

Supplement: Source code 1. [file elife-87055-code1.zip › code/fig5d.bmp]

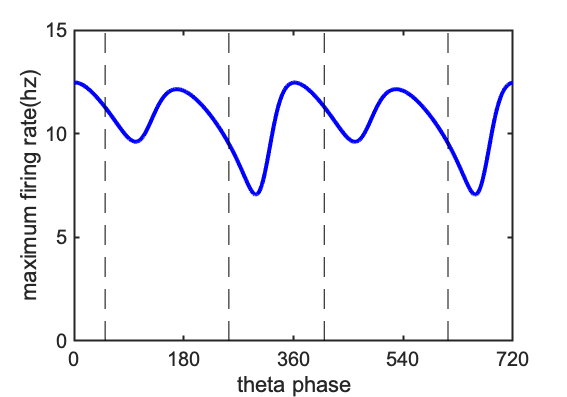

Supplement: Source code 1. [file elife-87055-code1.zip › code/fig4d_bimodal_curve.bmp]

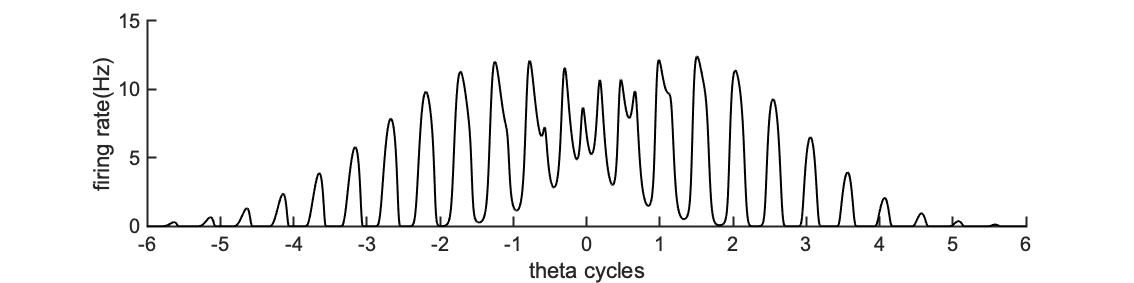

Supplement: Source code 1. [file elife-87055-code1.zip › code/fig4a.bmp]

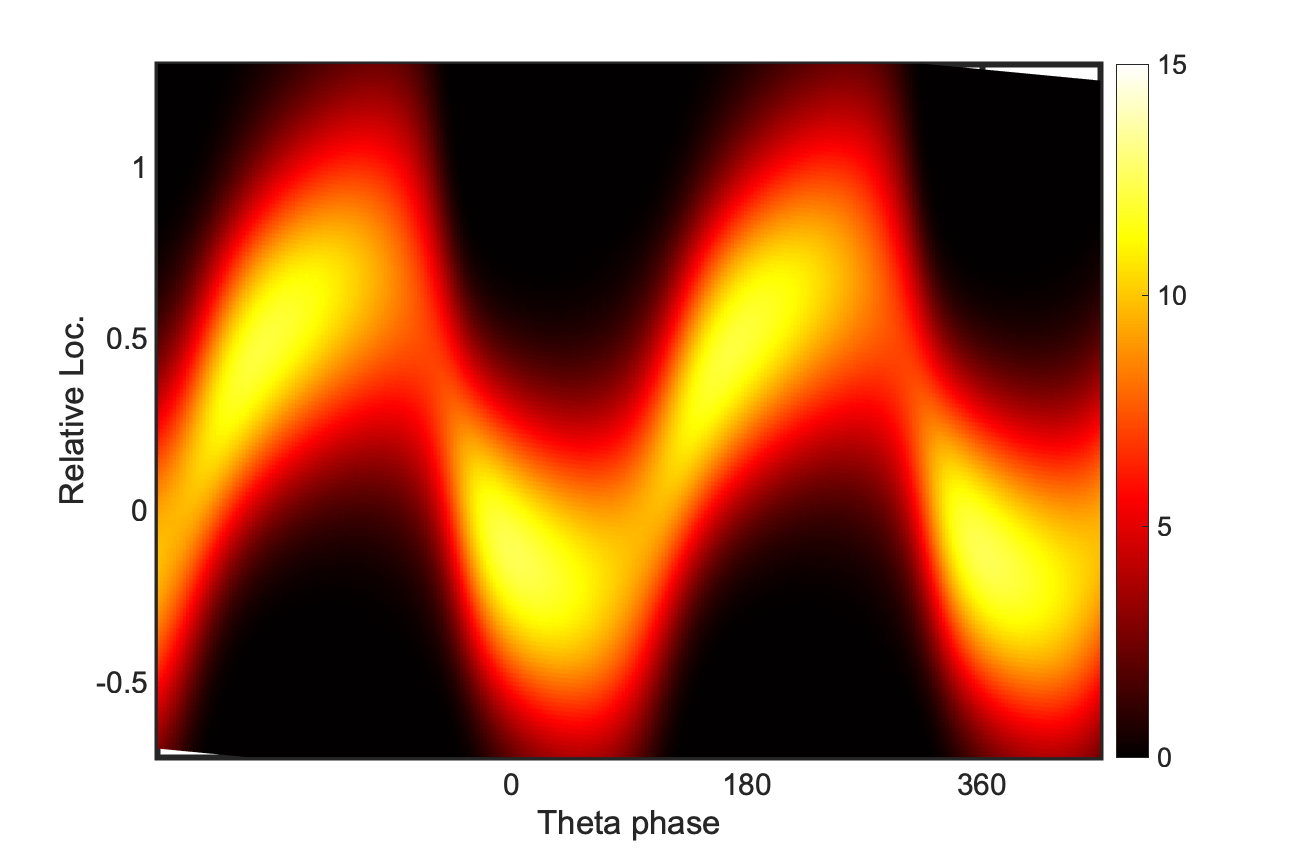

Supplement: Source code 1. [file elife-87055-code1.zip › code/fig4e.bmp]

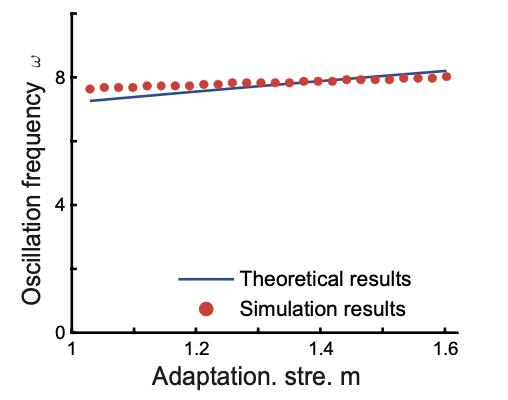

Supplement: Source code 1. [file elife-87055-code1.zip › code/fig2h.png]

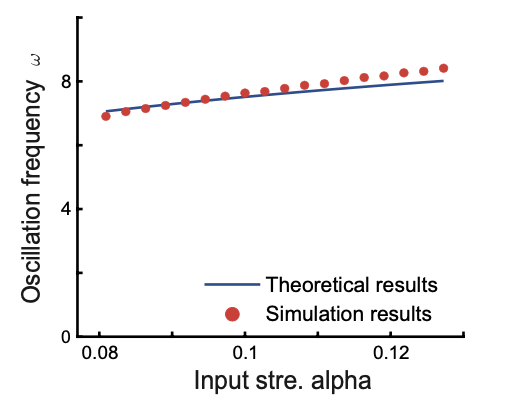

Supplement: Source code 1. [file elife-87055-code1.zip › code/fig2i.png]

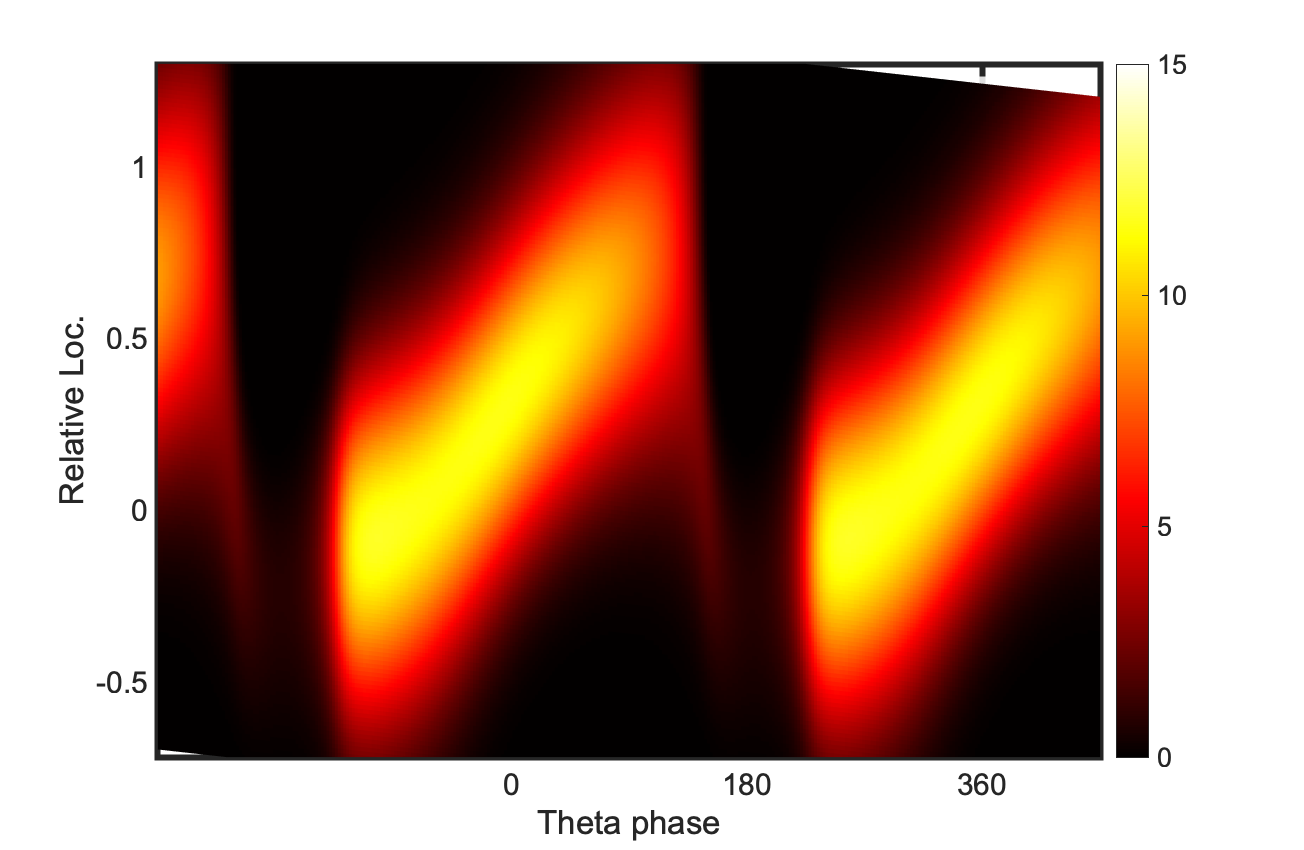

Supplement: Source code 1. [file elife-87055-code1.zip › code/fig4f.bmp]

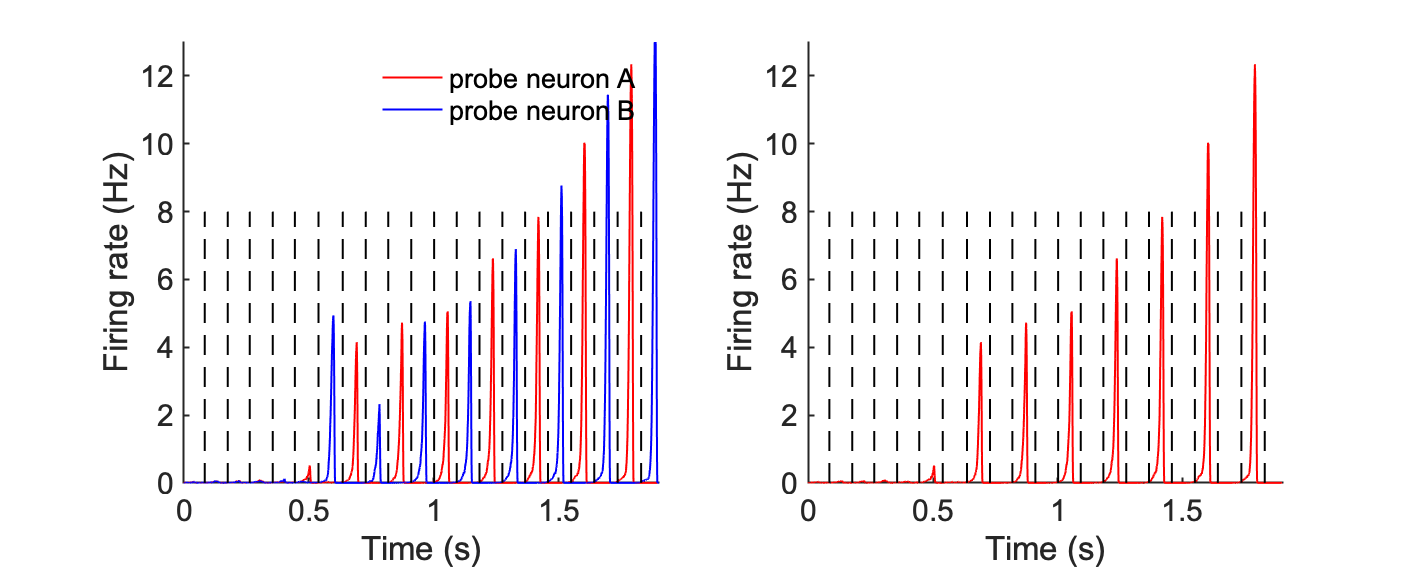

Supplement: Source code 1. [file elife-87055-code1.zip › code/fig5c.bmp]

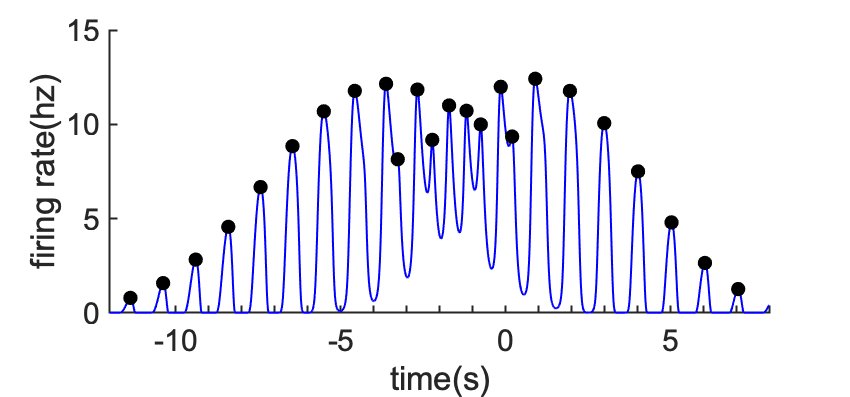

Supplement: Source code 1. [file elife-87055-code1.zip › code/fig3d.bmp]

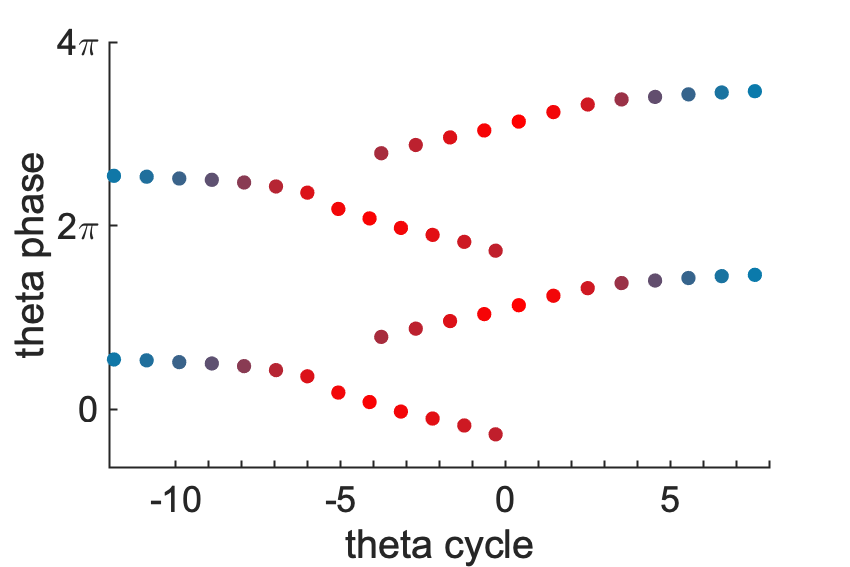

Supplement: Source code 1. [file elife-87055-code1.zip › code/fig3e.bmp]

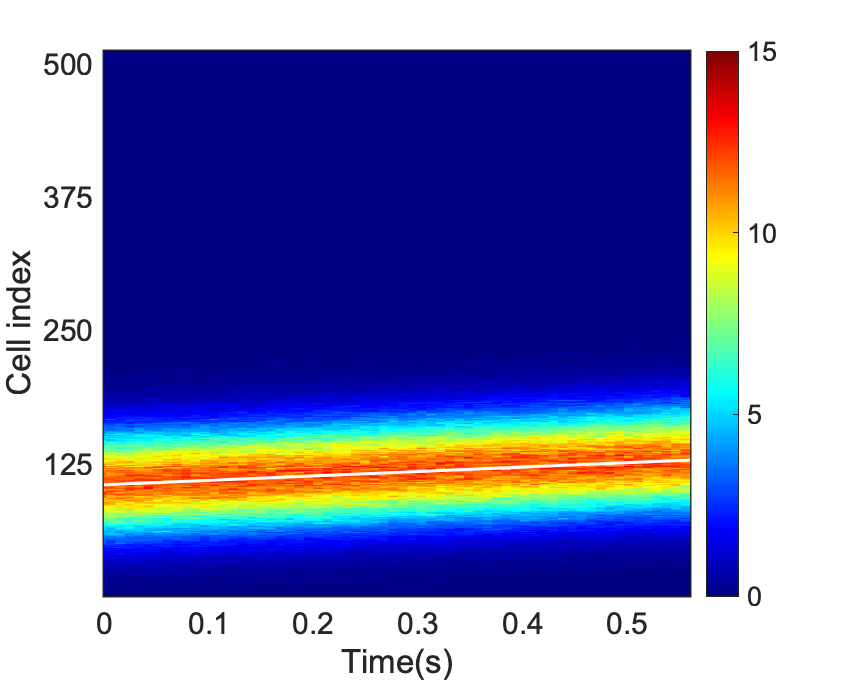

Supplement: Source code 1. [file elife-87055-code1.zip › code/fig2c.bmp]

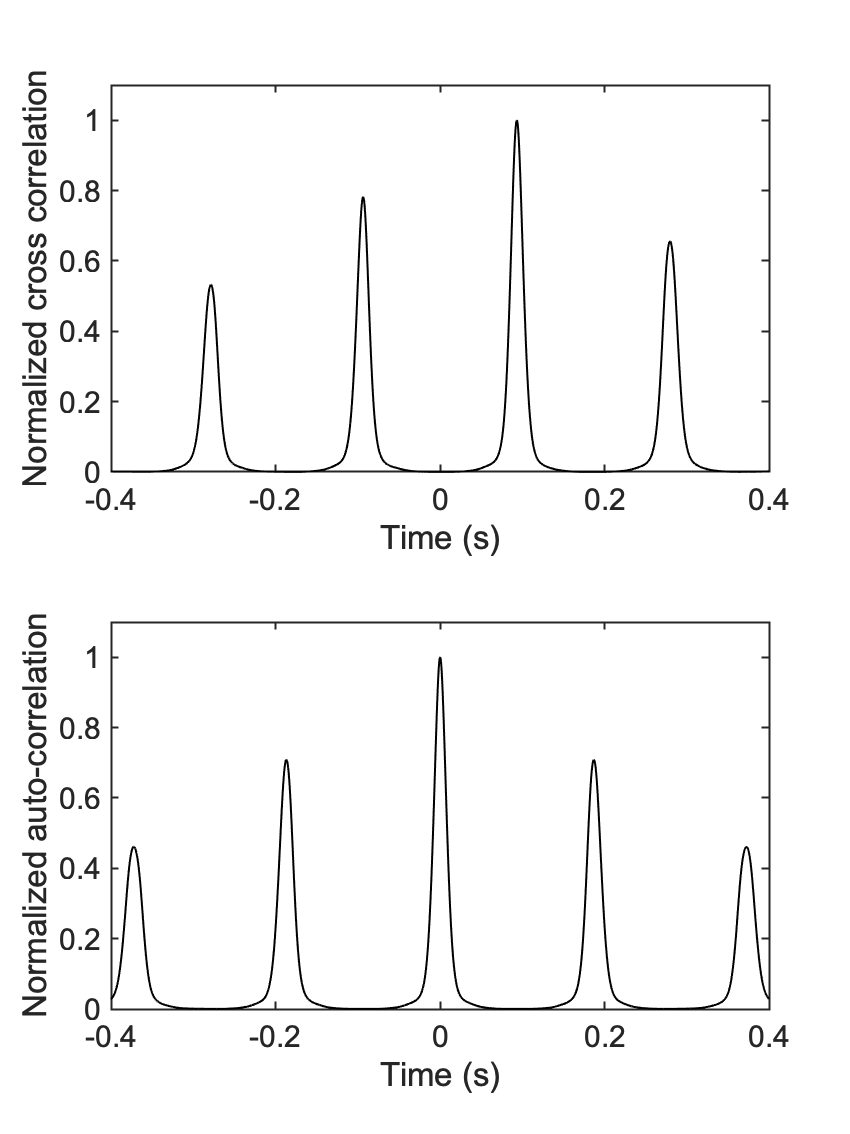

Supplement: Source code 1. [file elife-87055-code1.zip › code/5d.bmp]

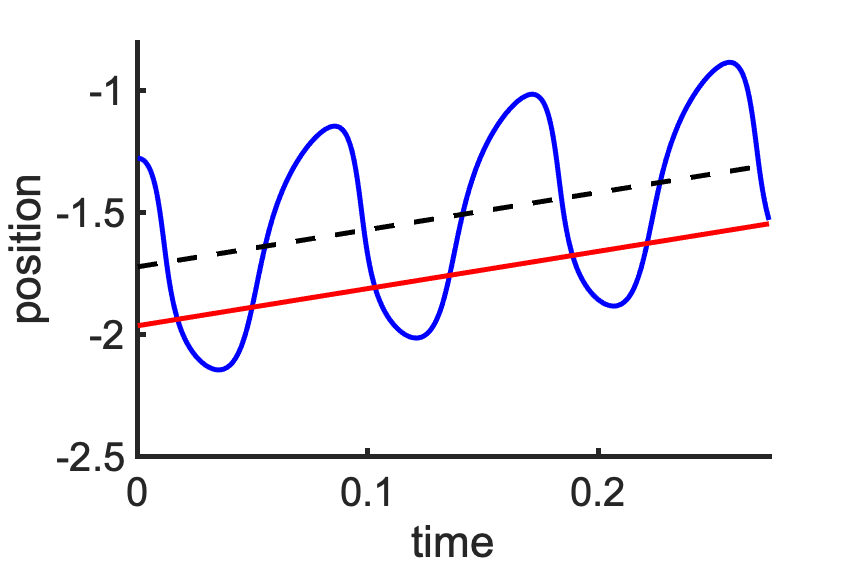

Supplement: Source code 1. [file elife-87055-code1.zip › code/fig2f.bmp]

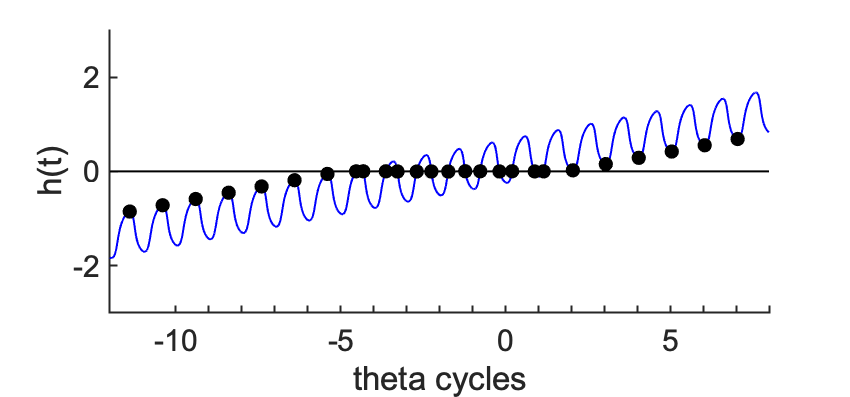

Supplement: Source code 1. [file elife-87055-code1.zip › code/fig3c.bmp]

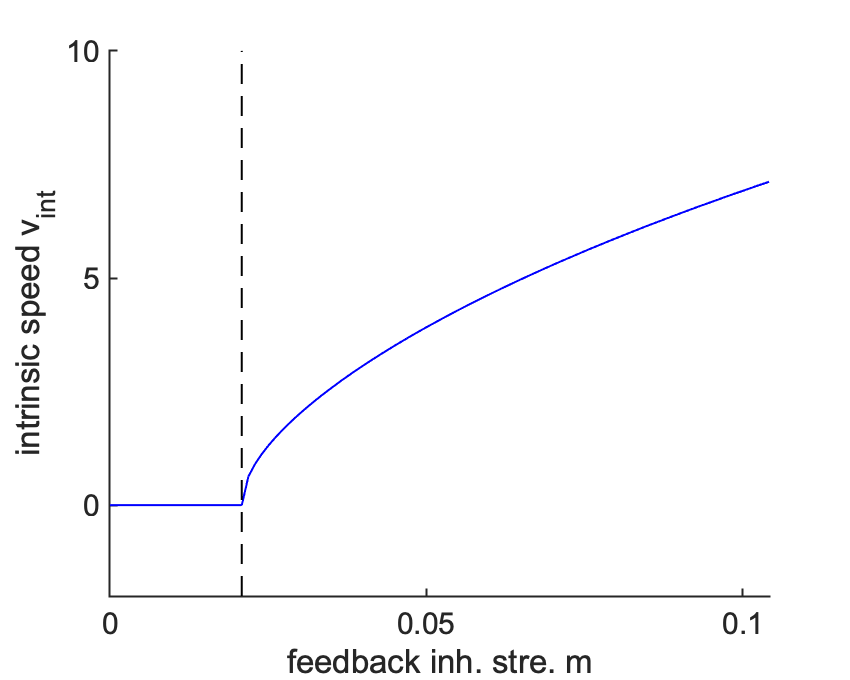

Supplement: Source code 1. [file elife-87055-code1.zip › code/fig2e.bmp]

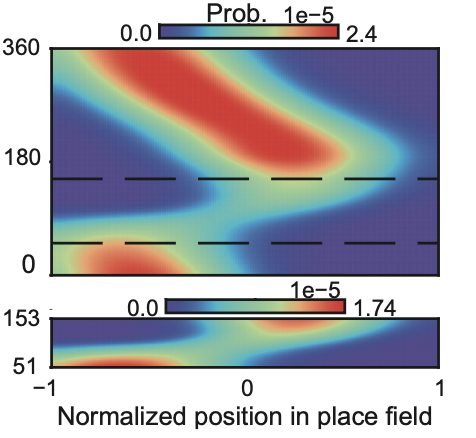

Supplement: Source code 1. [file elife-87055-code1.zip › code/fig4h.png]

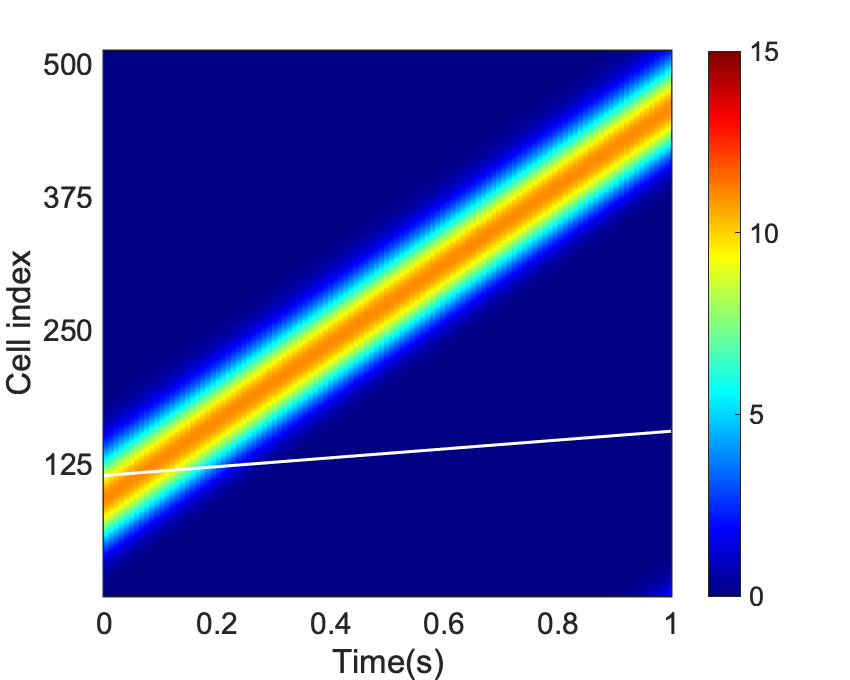

Supplement: Source code 1. [file elife-87055-code1.zip › code/fig2d.bmp]

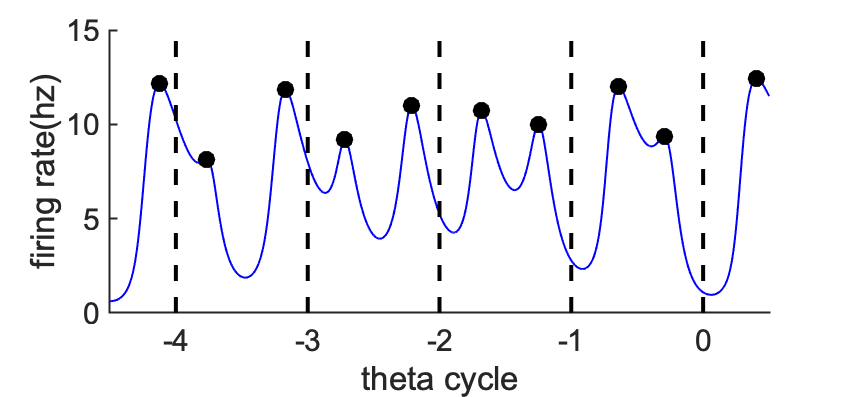

Supplement: Source code 1. [file elife-87055-code1.zip › code/fig3d_right_panel.bmp]

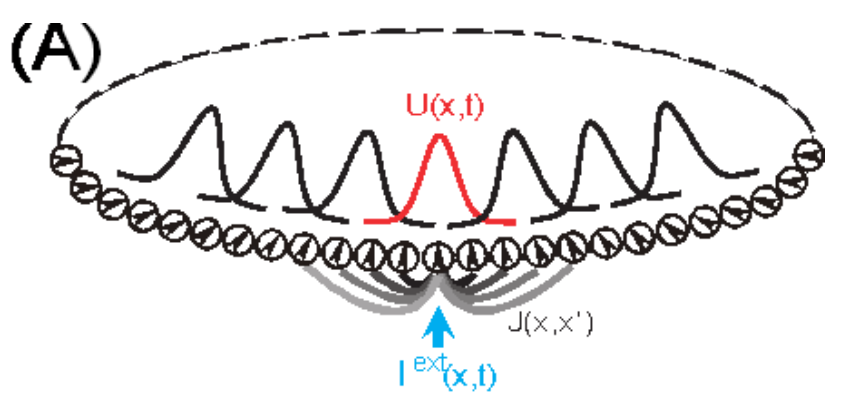

Supplement: Source code 1. [file elife-87055-code1.zip › code/μ£¬σæ╜σÉì.png]

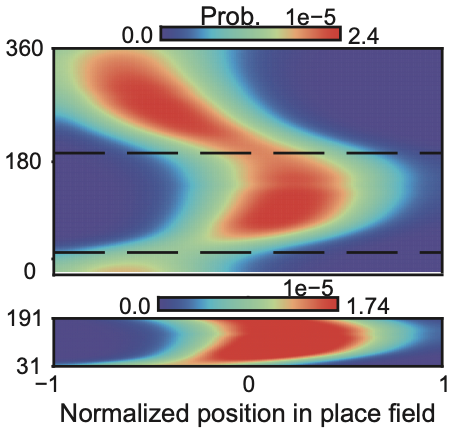

Supplement: Source code 1. [file elife-87055-code1.zip › code/fig4g.png]

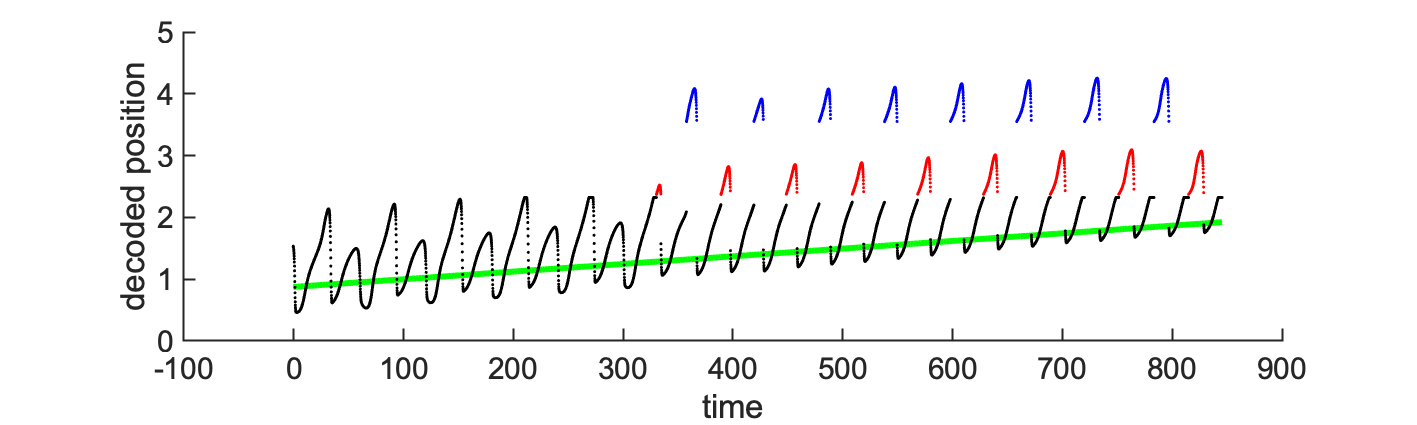

Supplement: Source code 1. [file elife-87055-code1.zip › code/fig5b_lower_panel.bmp]

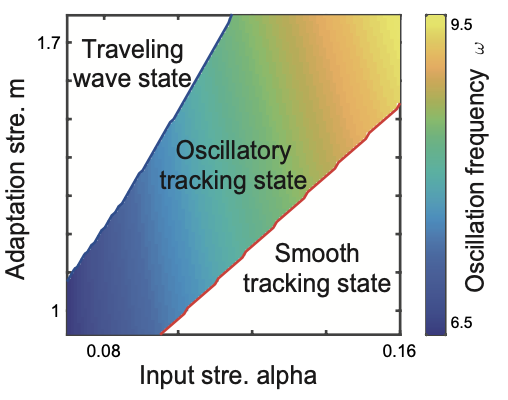

Supplement: Source code 1. [file elife-87055-code1.zip › code/fig2g.png]

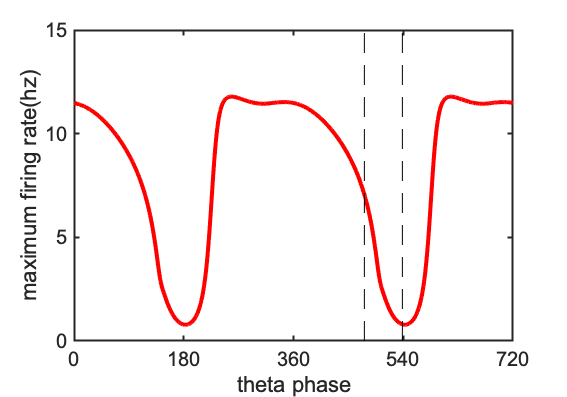

Supplement: Source code 1. [file elife-87055-code1.zip › code/fig4d_unimodal_cell.bmp]

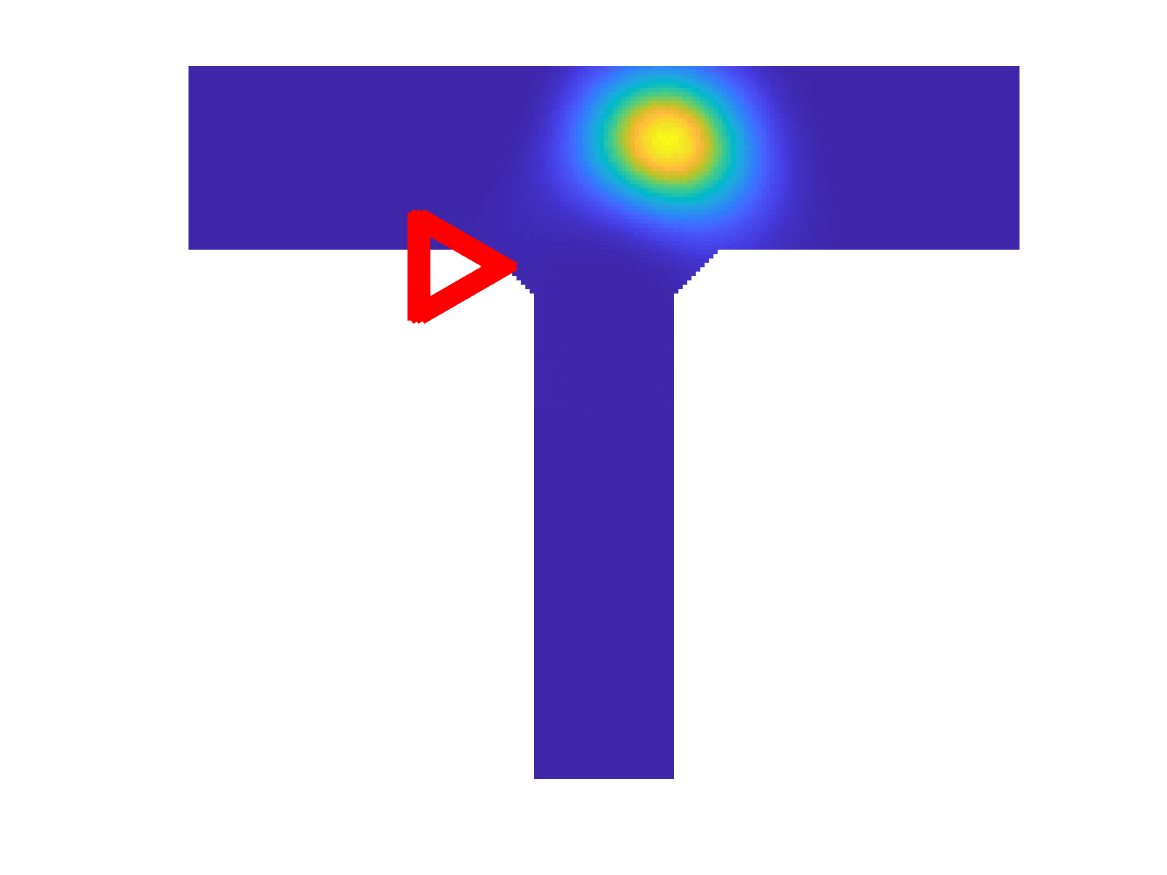

Supplement: Source code 1. [file elife-87055-code1.zip › code/fig5b_frames/251.bmp]

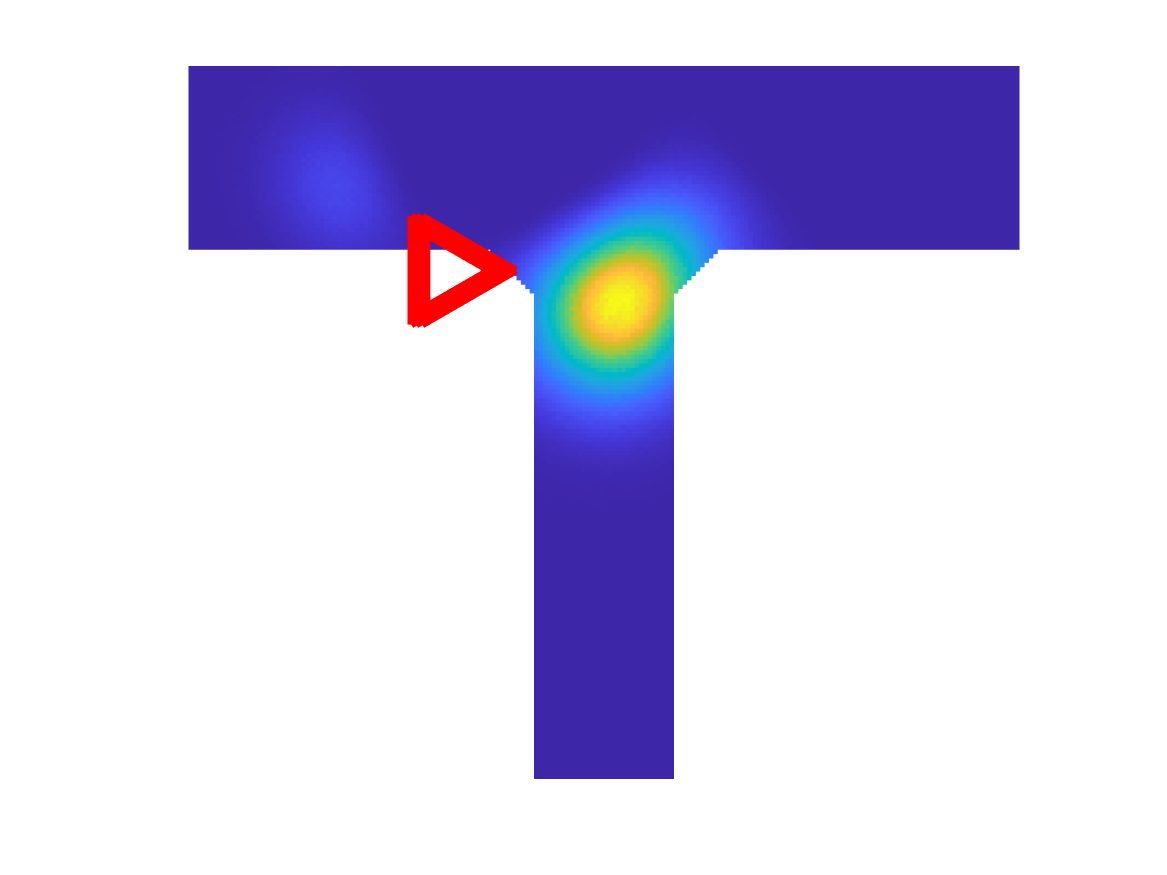

Supplement: Source code 1. [file elife-87055-code1.zip › code/fig5b_frames/245.bmp]

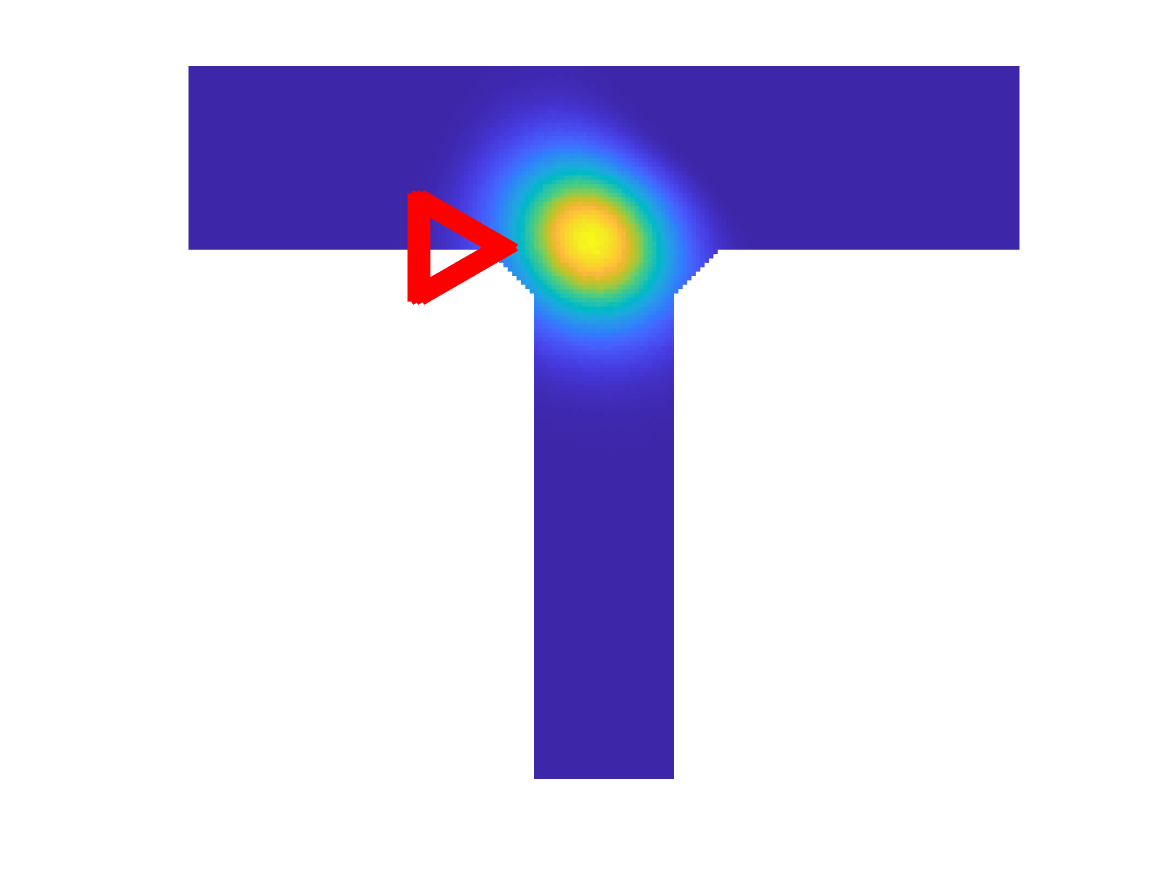

Supplement: Source code 1. [file elife-87055-code1.zip › code/fig5b_frames/279.bmp]

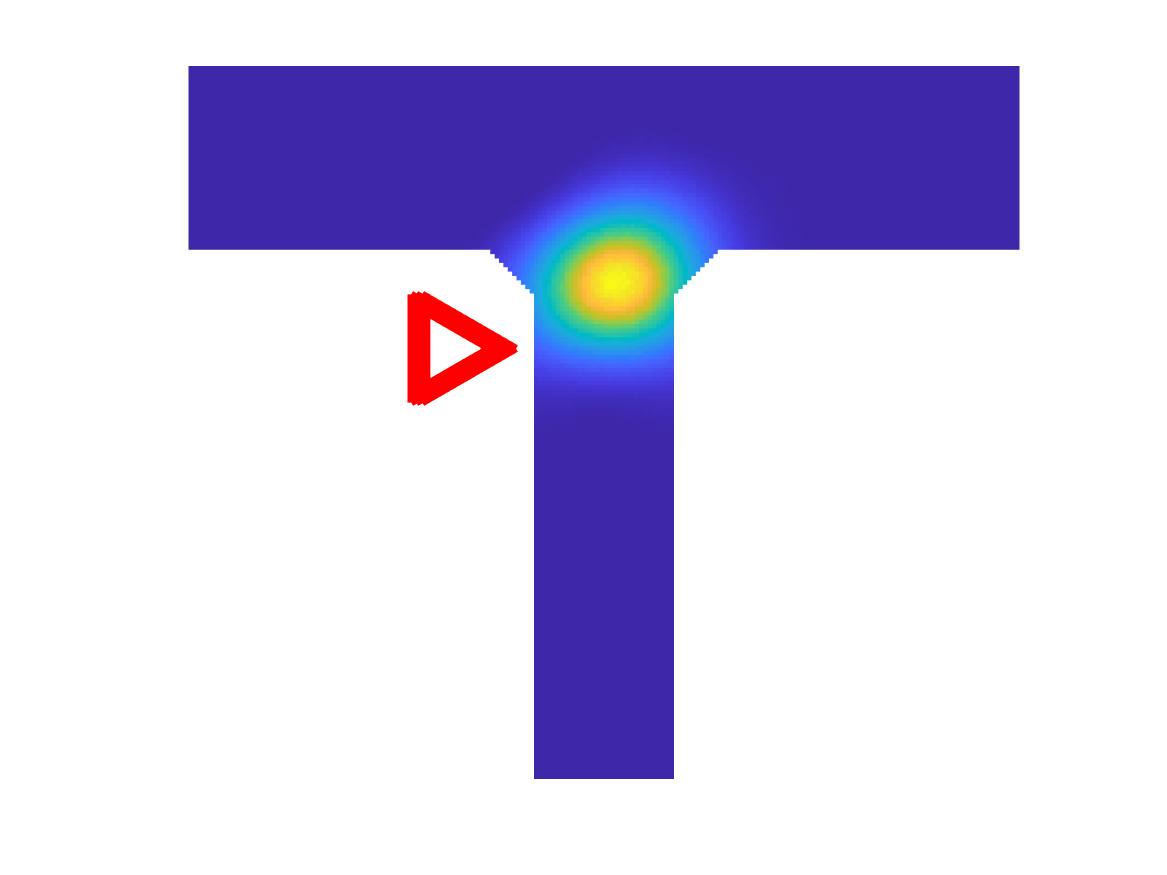

Supplement: Source code 1. [file elife-87055-code1.zip › code/fig5b_frames/127.bmp]

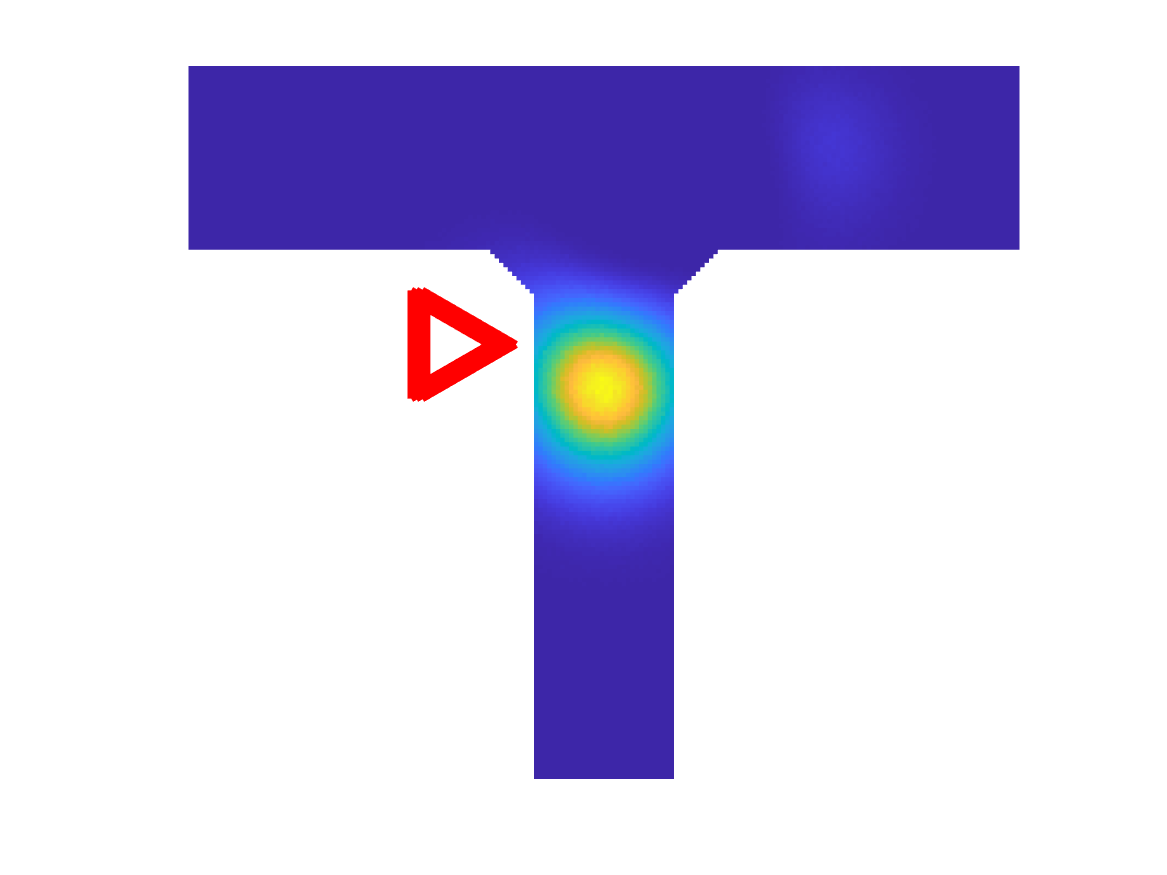

Supplement: Source code 1. [file elife-87055-code1.zip › code/fig5b_frames/133.bmp]

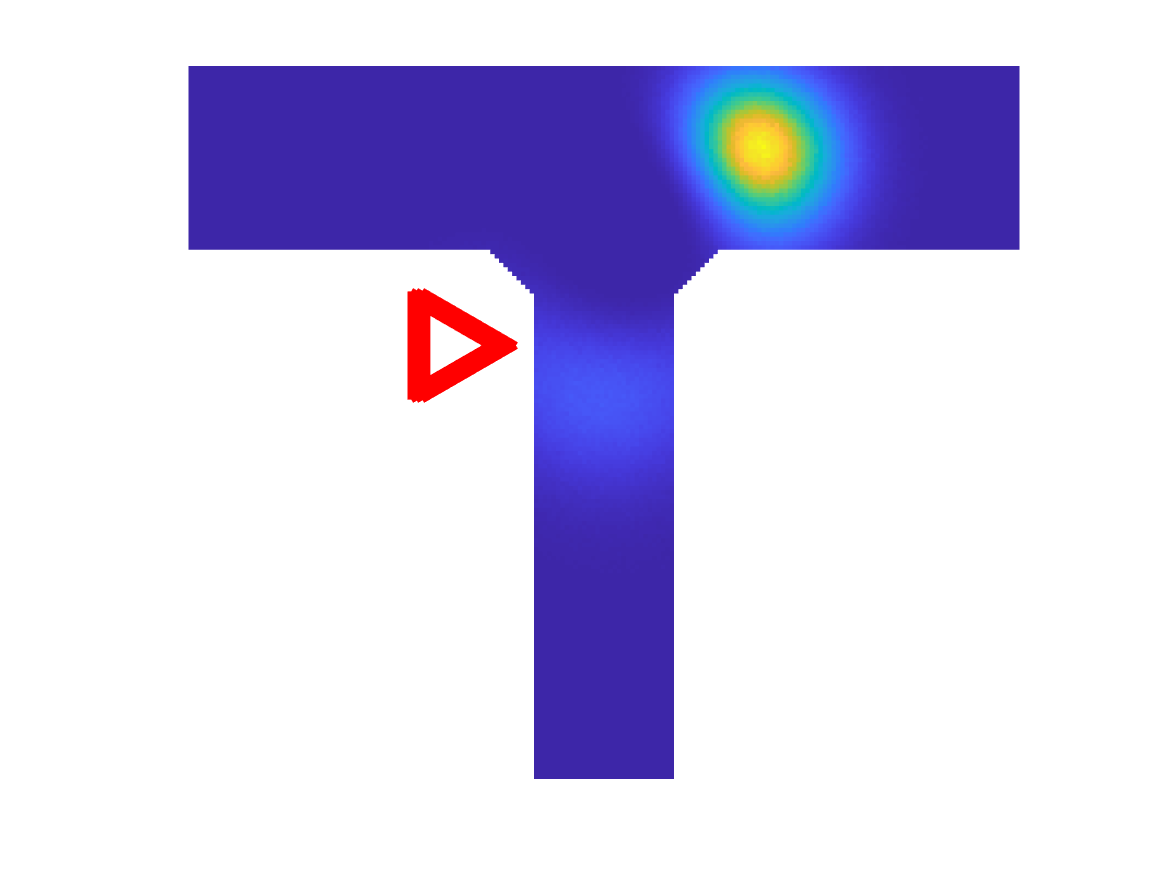

Supplement: Source code 1. [file elife-87055-code1.zip › code/fig5b_frames/132.bmp]

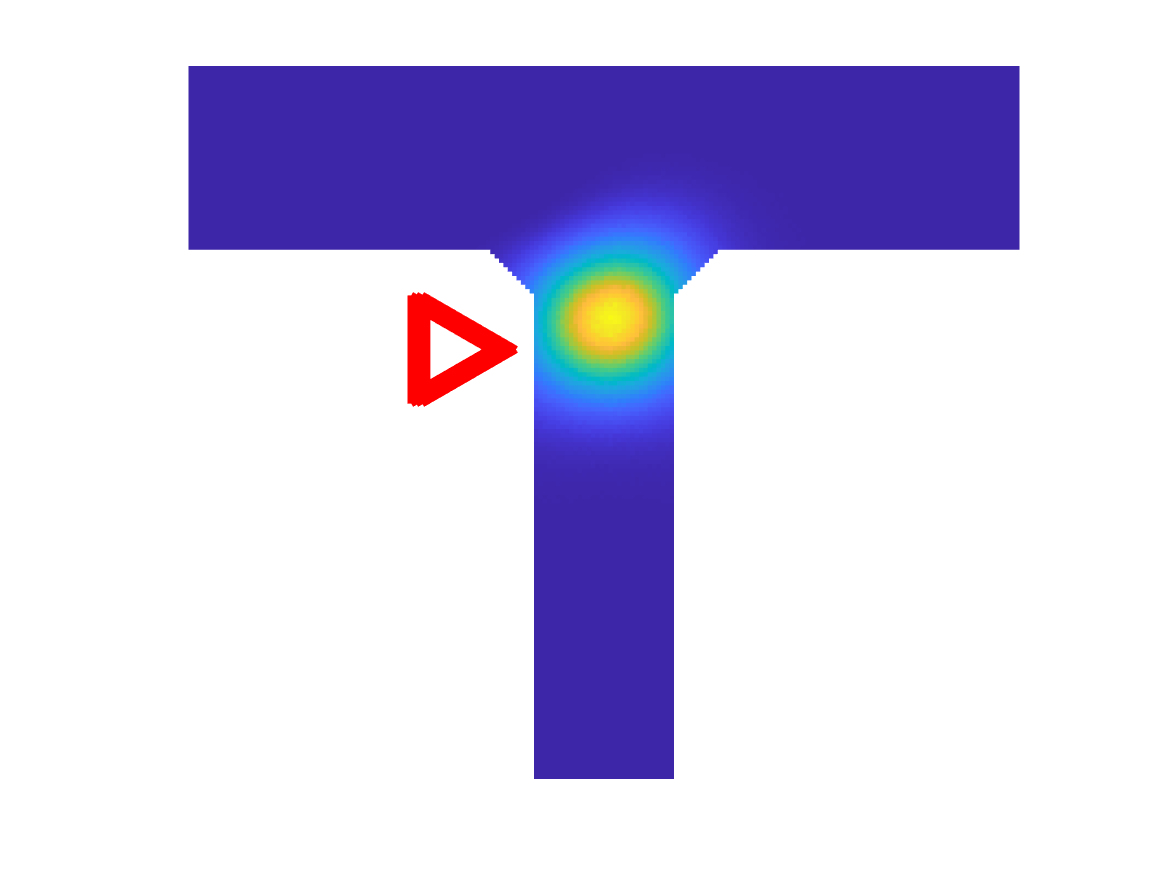

Supplement: Source code 1. [file elife-87055-code1.zip › code/fig5b_frames/126.bmp]

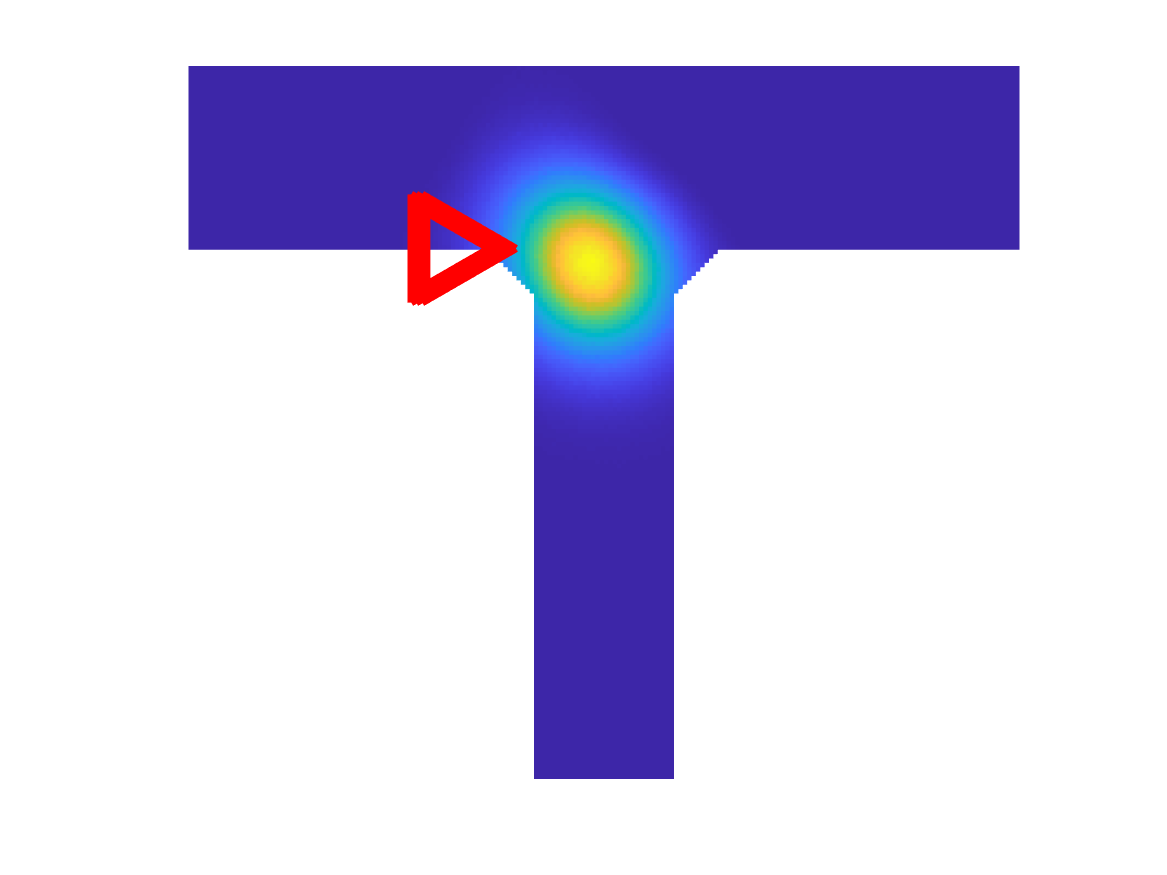

Supplement: Source code 1. [file elife-87055-code1.zip › code/fig5b_frames/278.bmp]

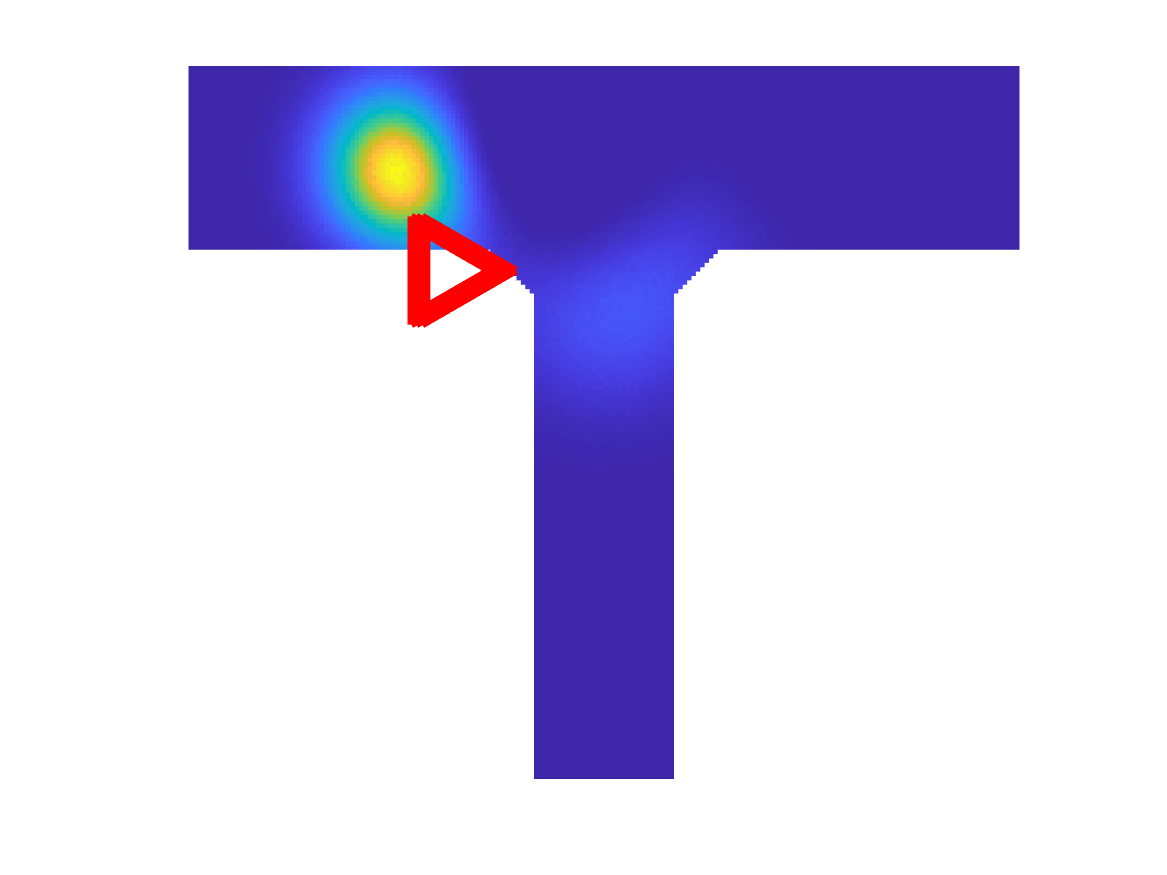

Supplement: Source code 1. [file elife-87055-code1.zip › code/fig5b_frames/244.bmp]

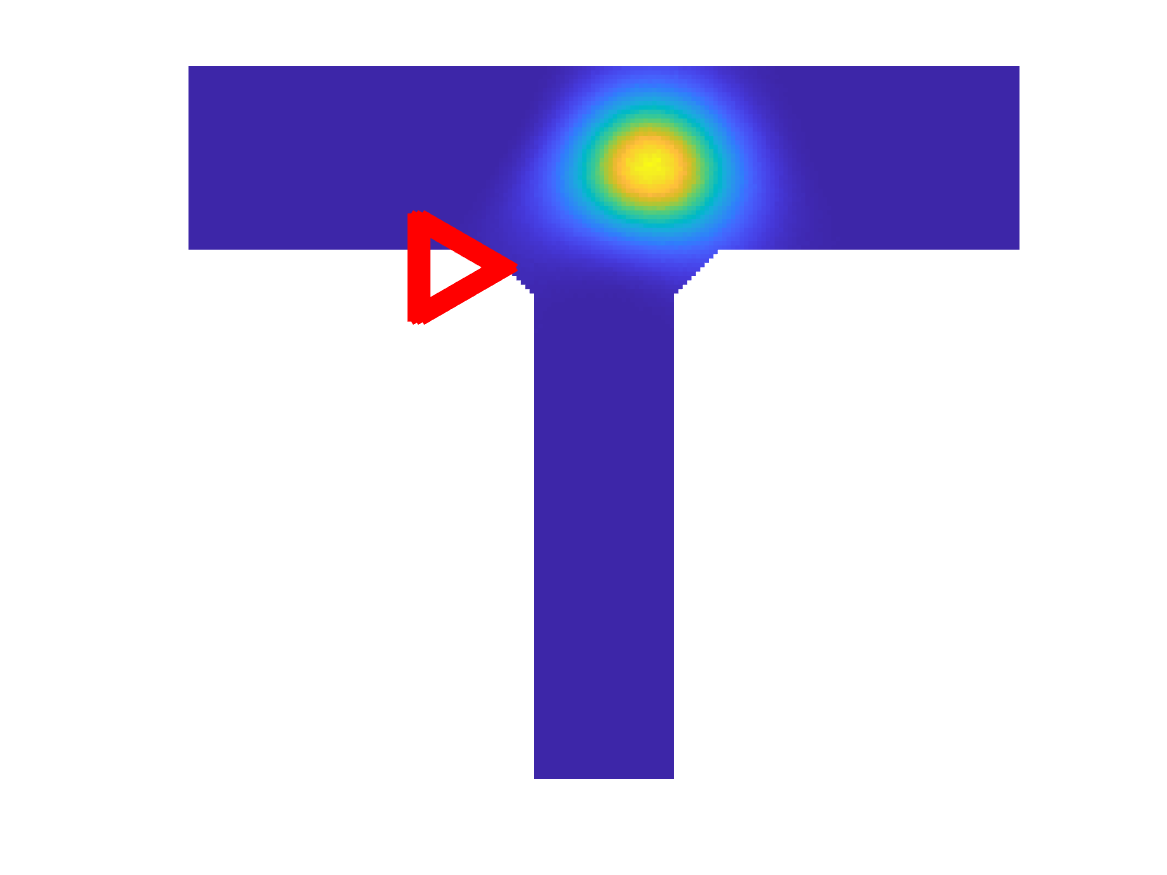

Supplement: Source code 1. [file elife-87055-code1.zip › code/fig5b_frames/250.bmp]

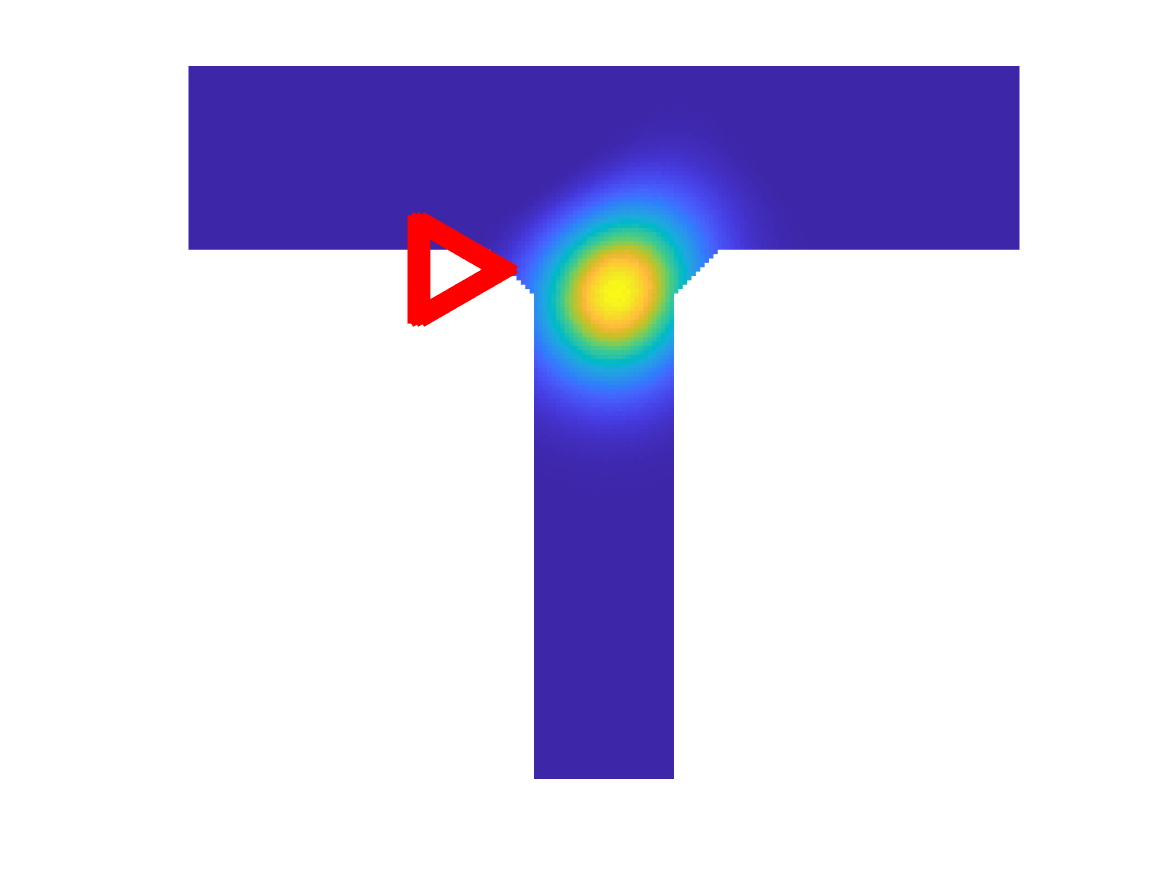

Supplement: Source code 1. [file elife-87055-code1.zip › code/fig5b_frames/246.bmp]

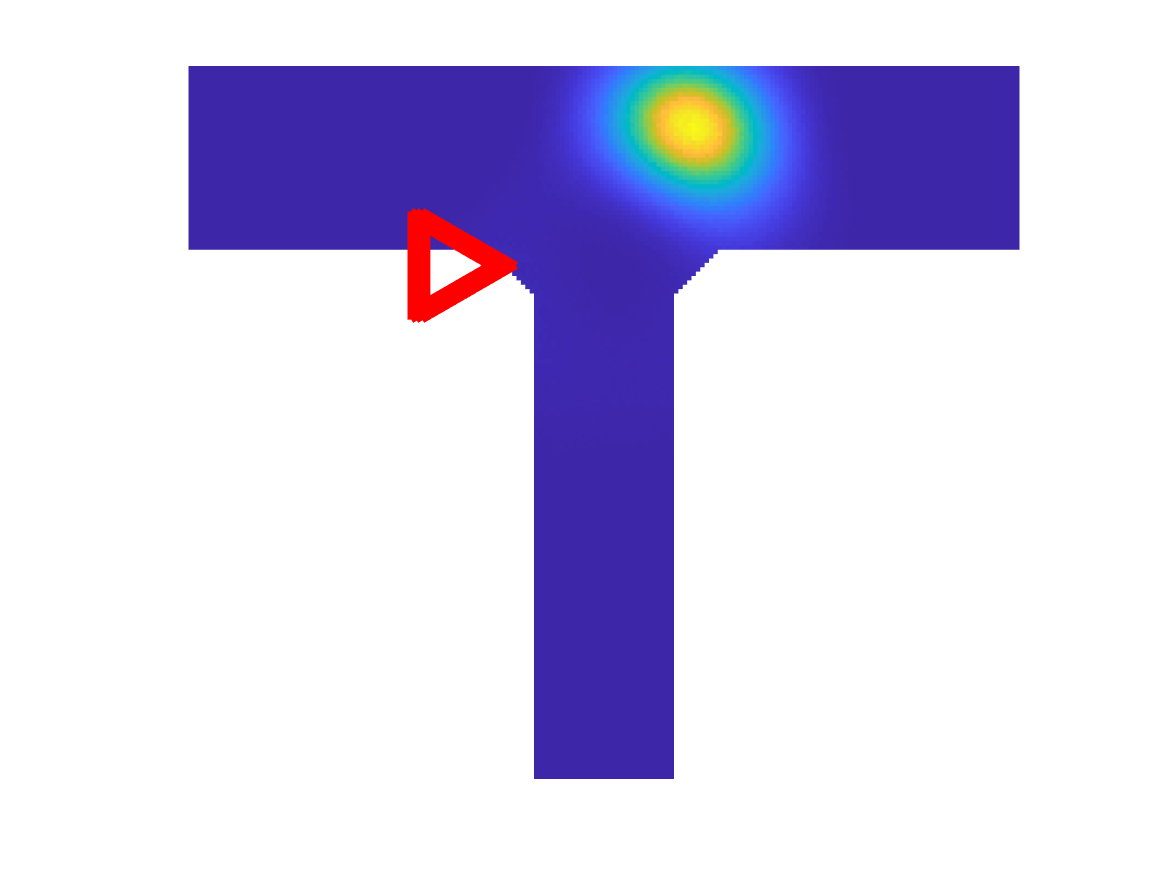

Supplement: Source code 1. [file elife-87055-code1.zip › code/fig5b_frames/252.bmp]

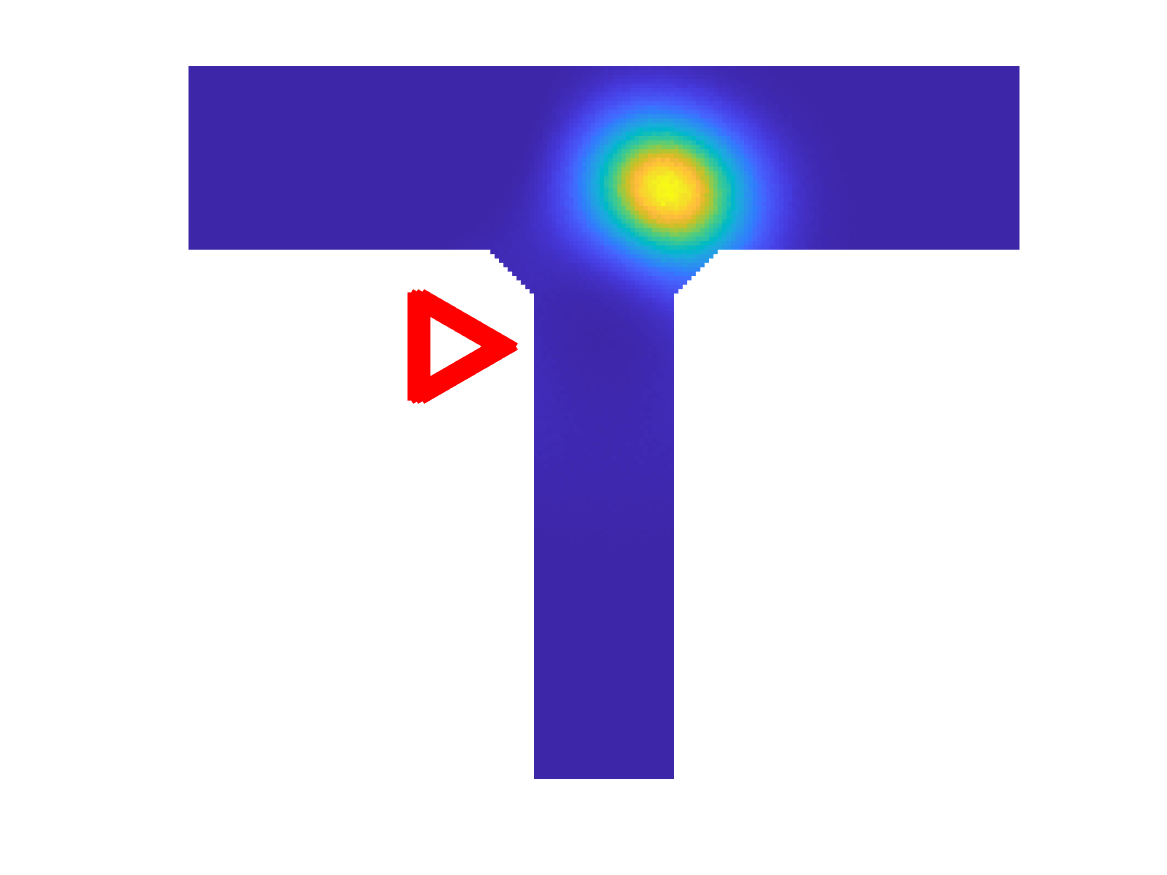

Supplement: Source code 1. [file elife-87055-code1.zip › code/fig5b_frames/130.bmp]

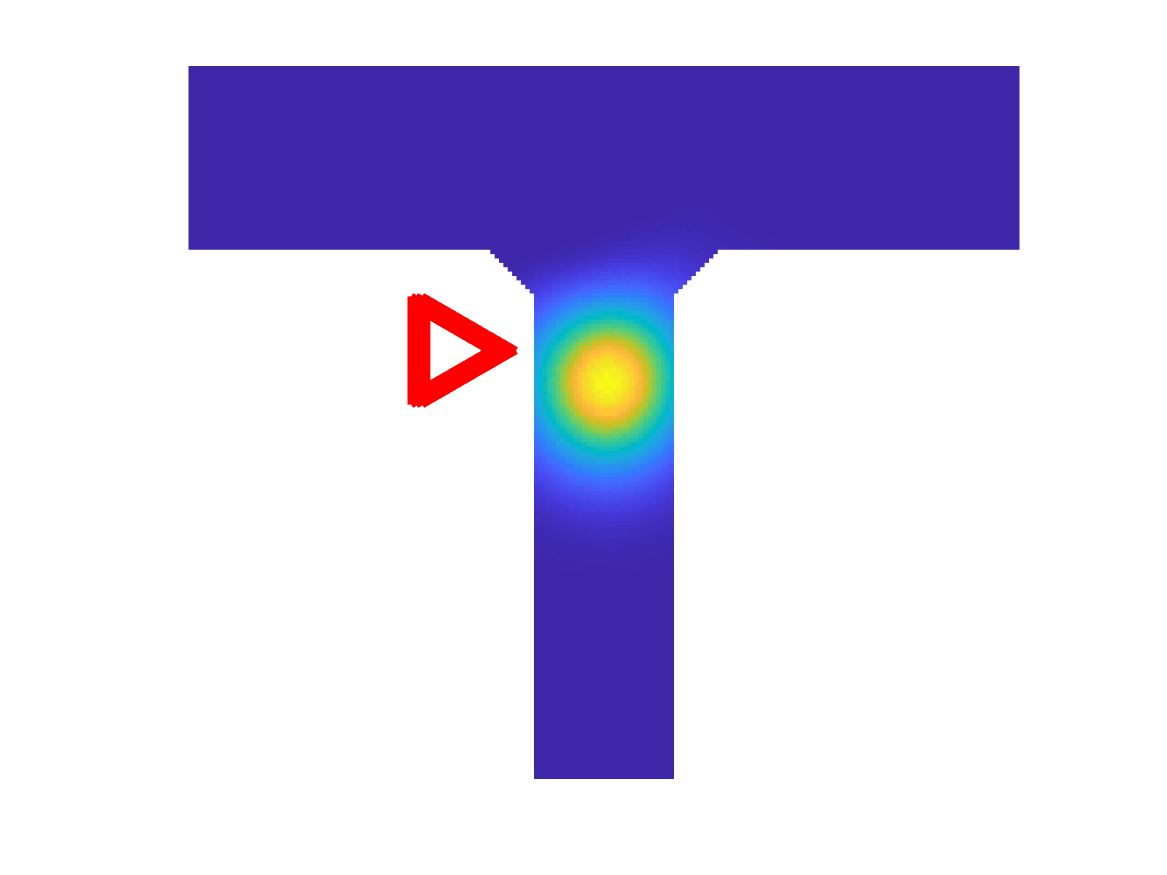

Supplement: Source code 1. [file elife-87055-code1.zip › code/fig5b_frames/124.bmp]

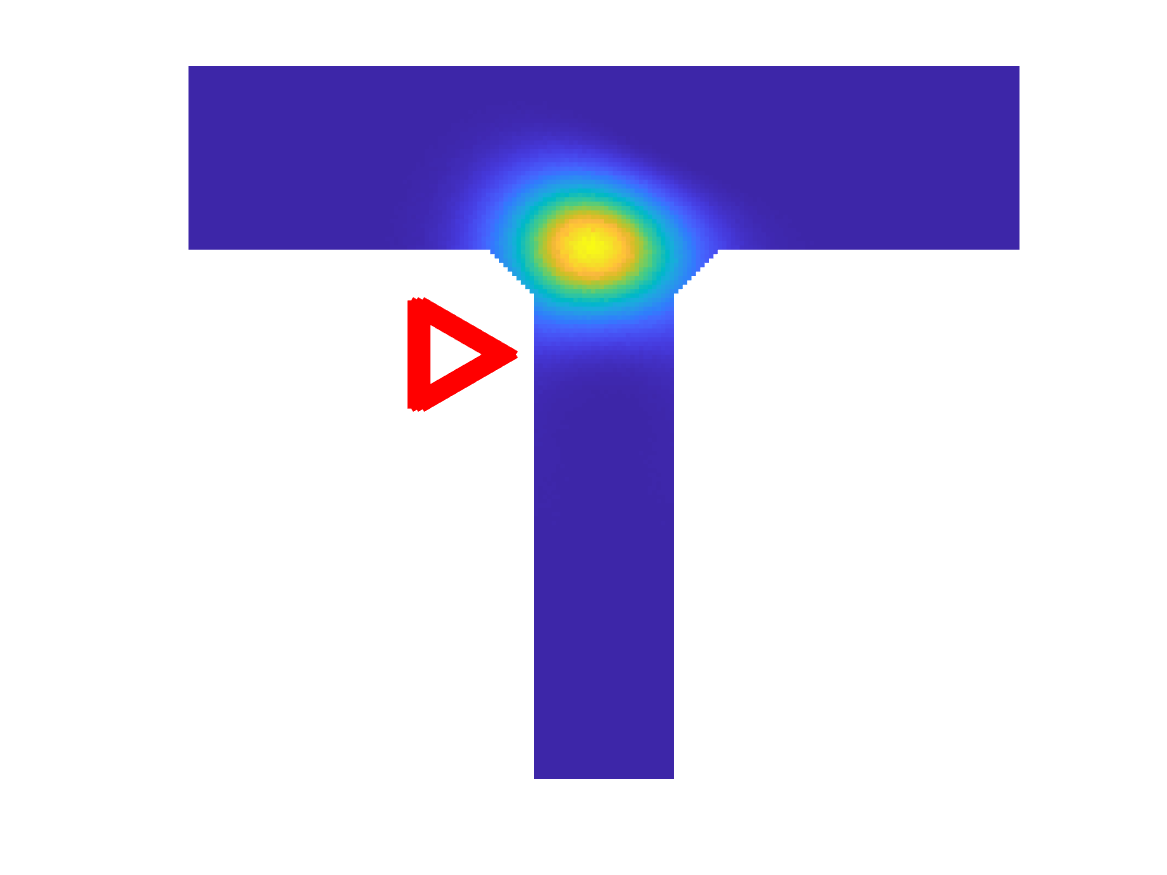

Supplement: Source code 1. [file elife-87055-code1.zip › code/fig5b_frames/118.bmp]

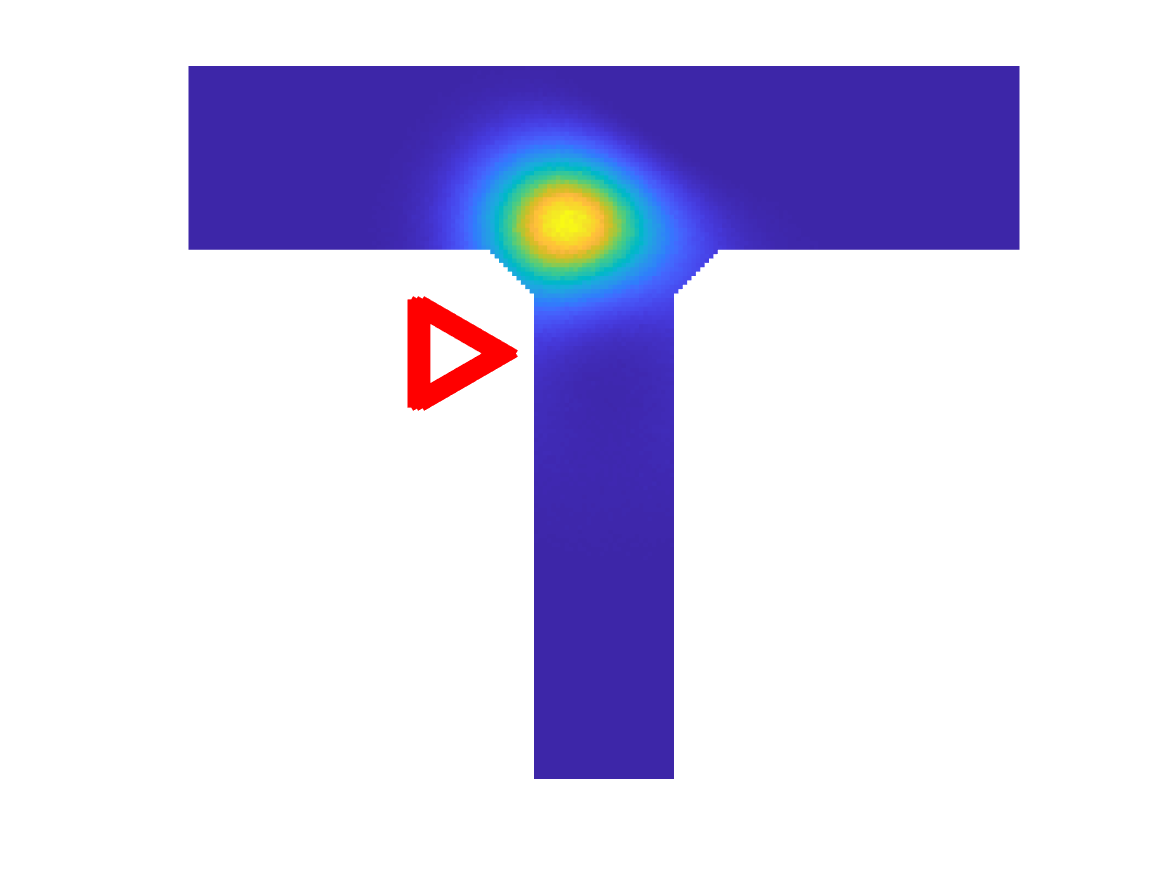

Supplement: Source code 1. [file elife-87055-code1.zip › code/fig5b_frames/119.bmp]

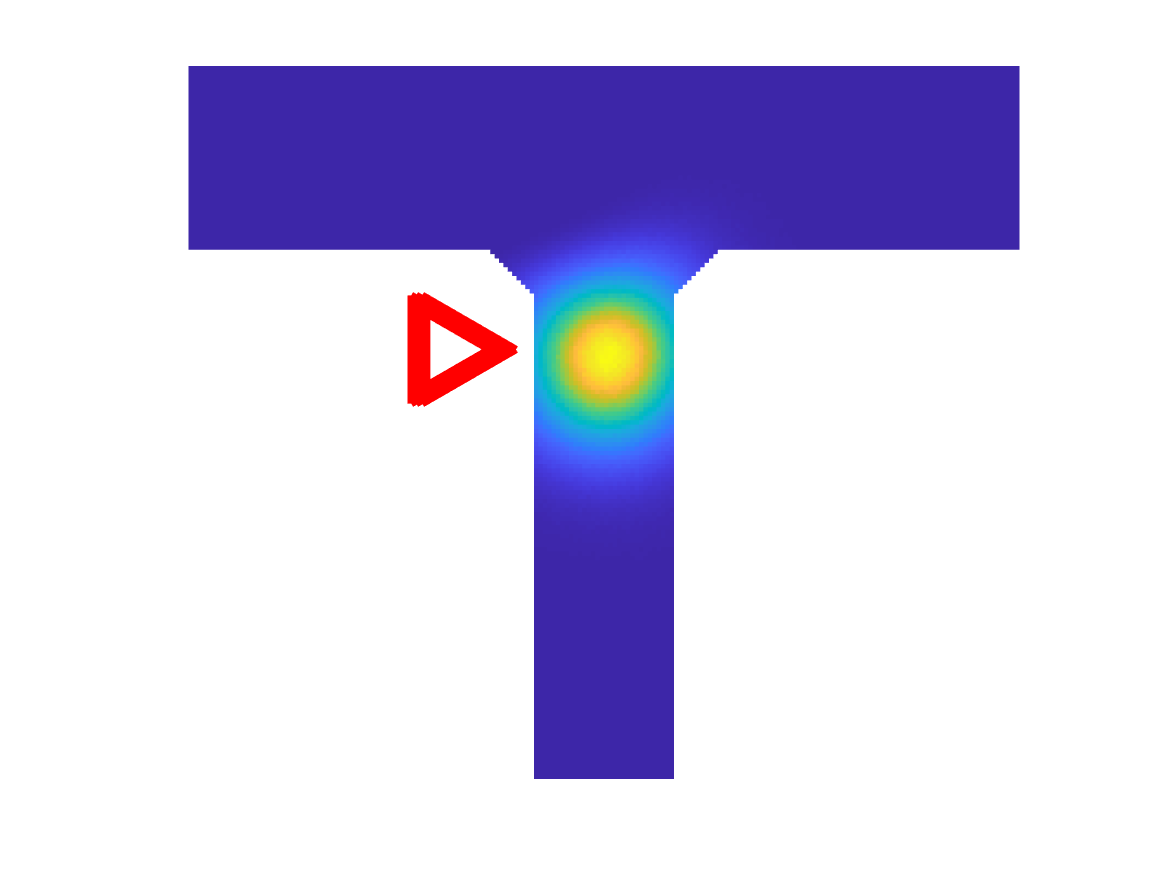

Supplement: Source code 1. [file elife-87055-code1.zip › code/fig5b_frames/125.bmp]

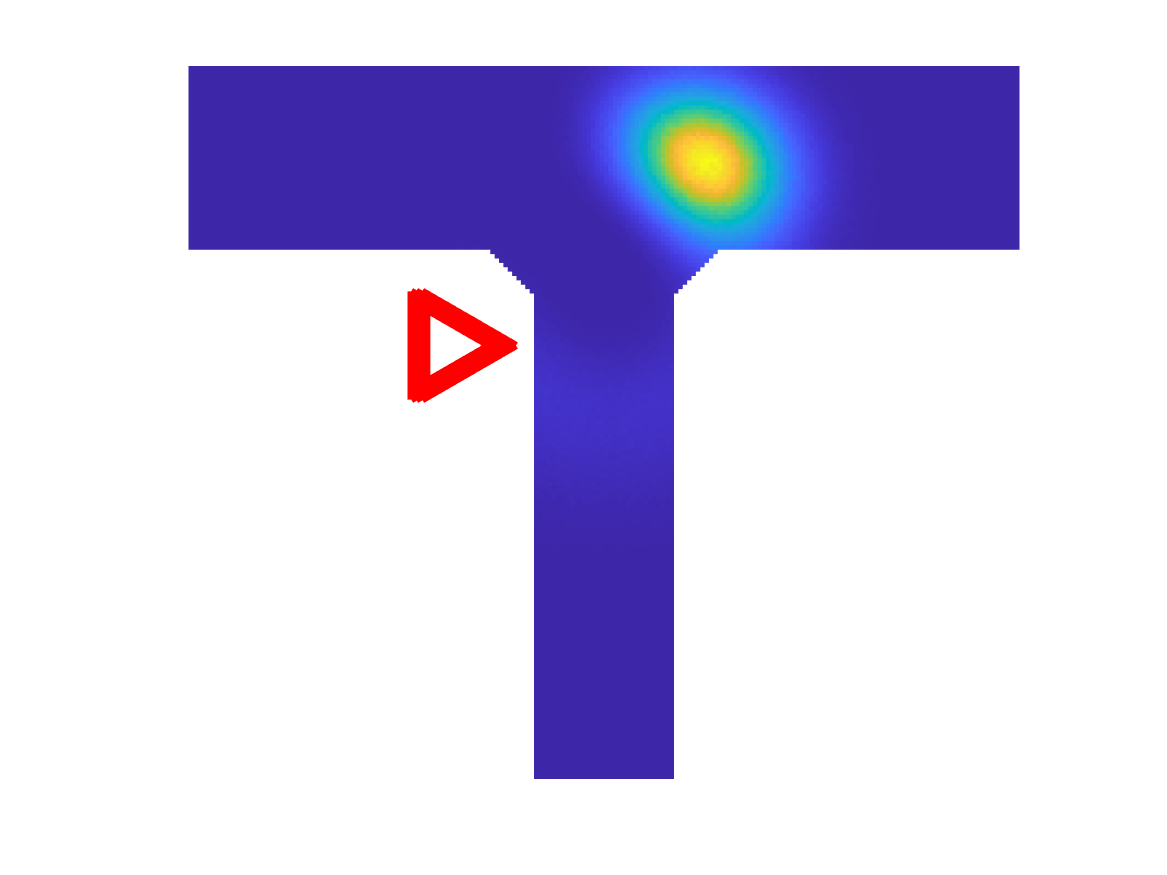

Supplement: Source code 1. [file elife-87055-code1.zip › code/fig5b_frames/131.bmp]

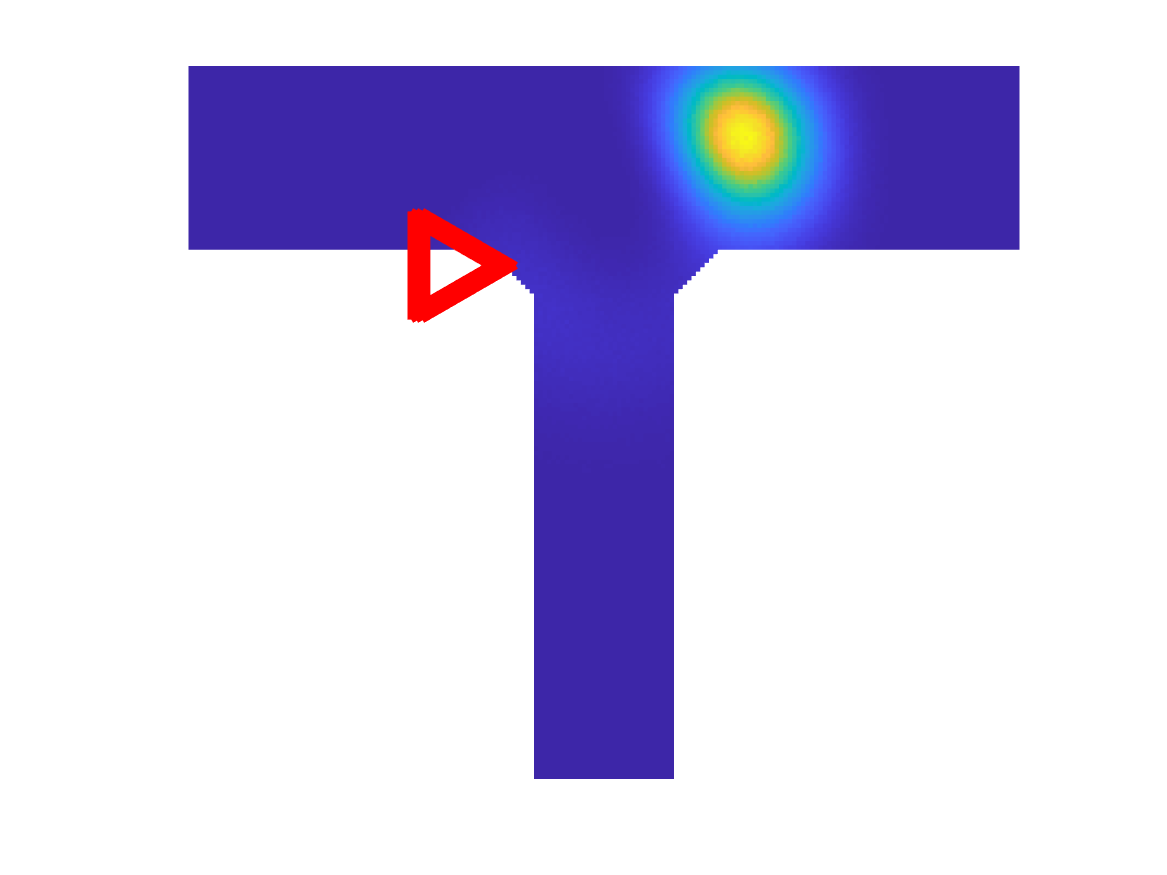

Supplement: Source code 1. [file elife-87055-code1.zip › code/fig5b_frames/253.bmp]

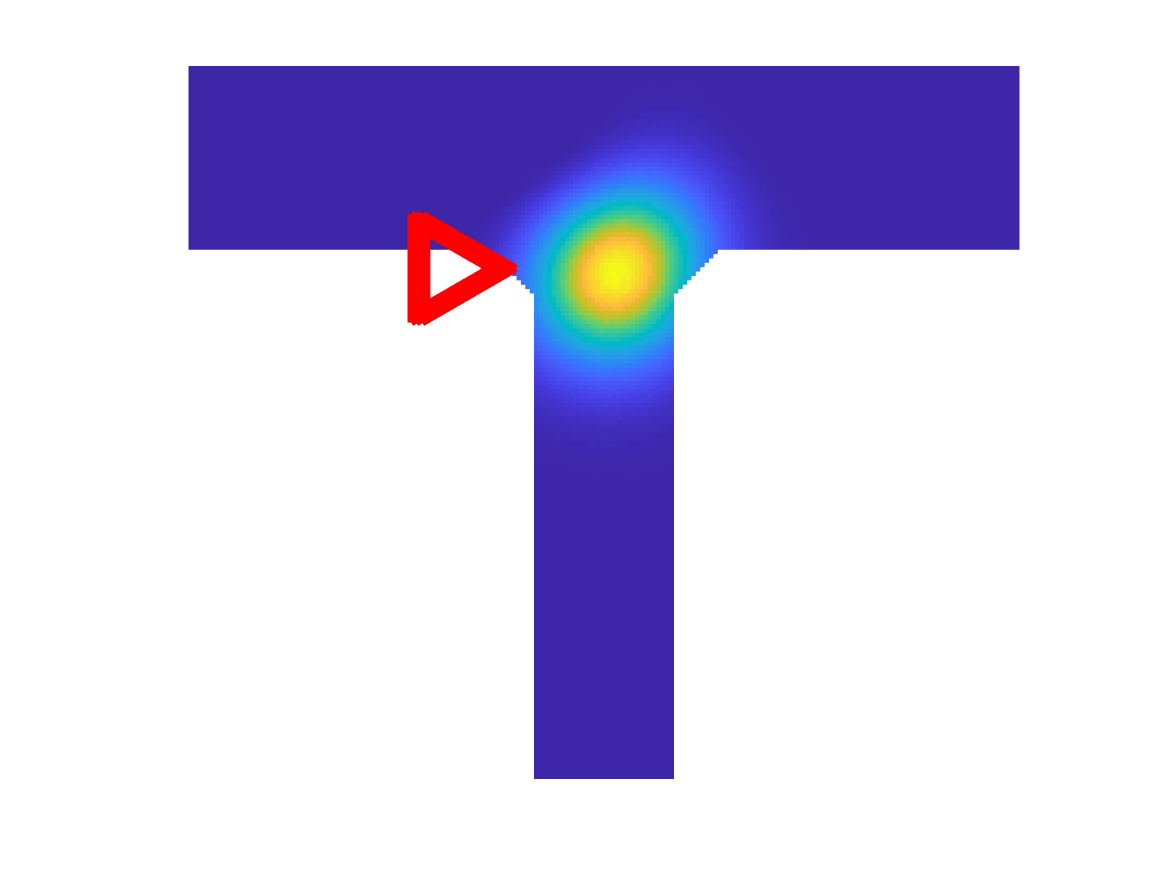

Supplement: Source code 1. [file elife-87055-code1.zip › code/fig5b_frames/247.bmp]

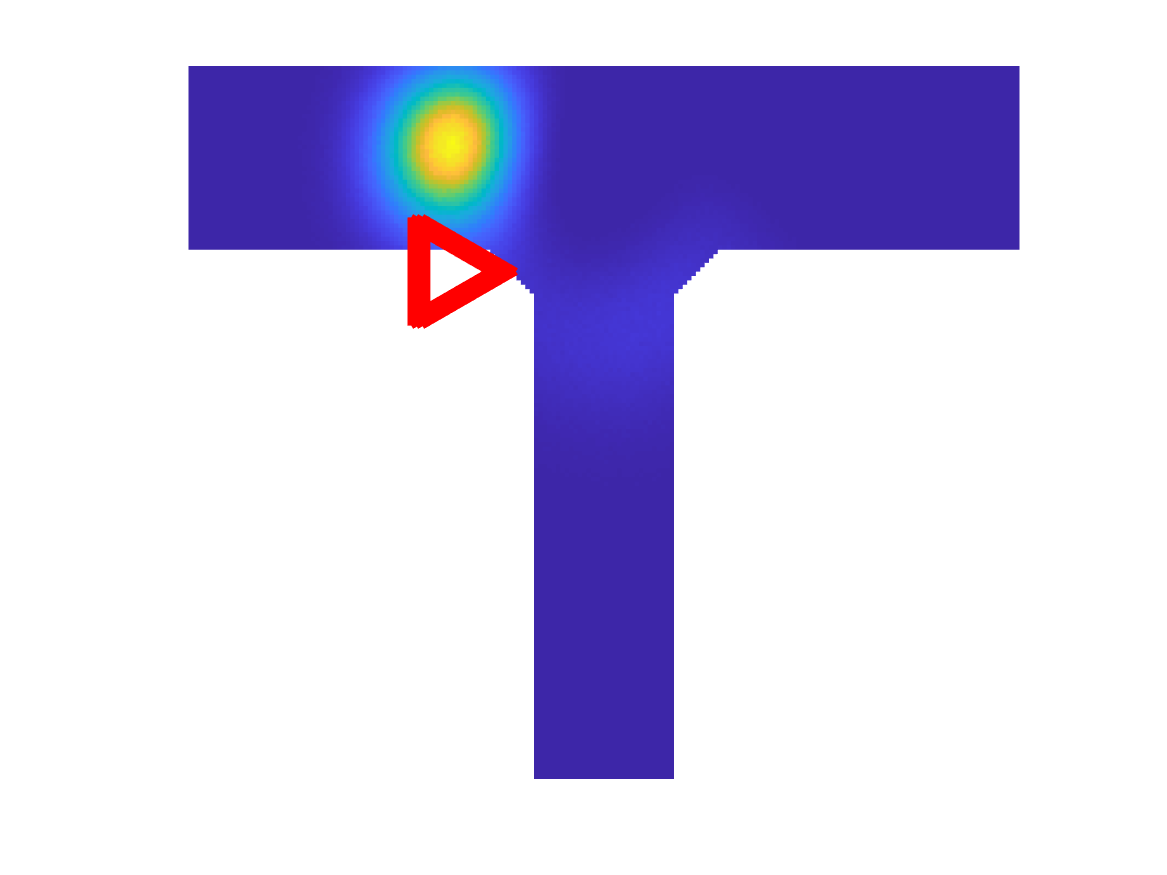

Supplement: Source code 1. [file elife-87055-code1.zip › code/fig5b_frames/243.bmp]

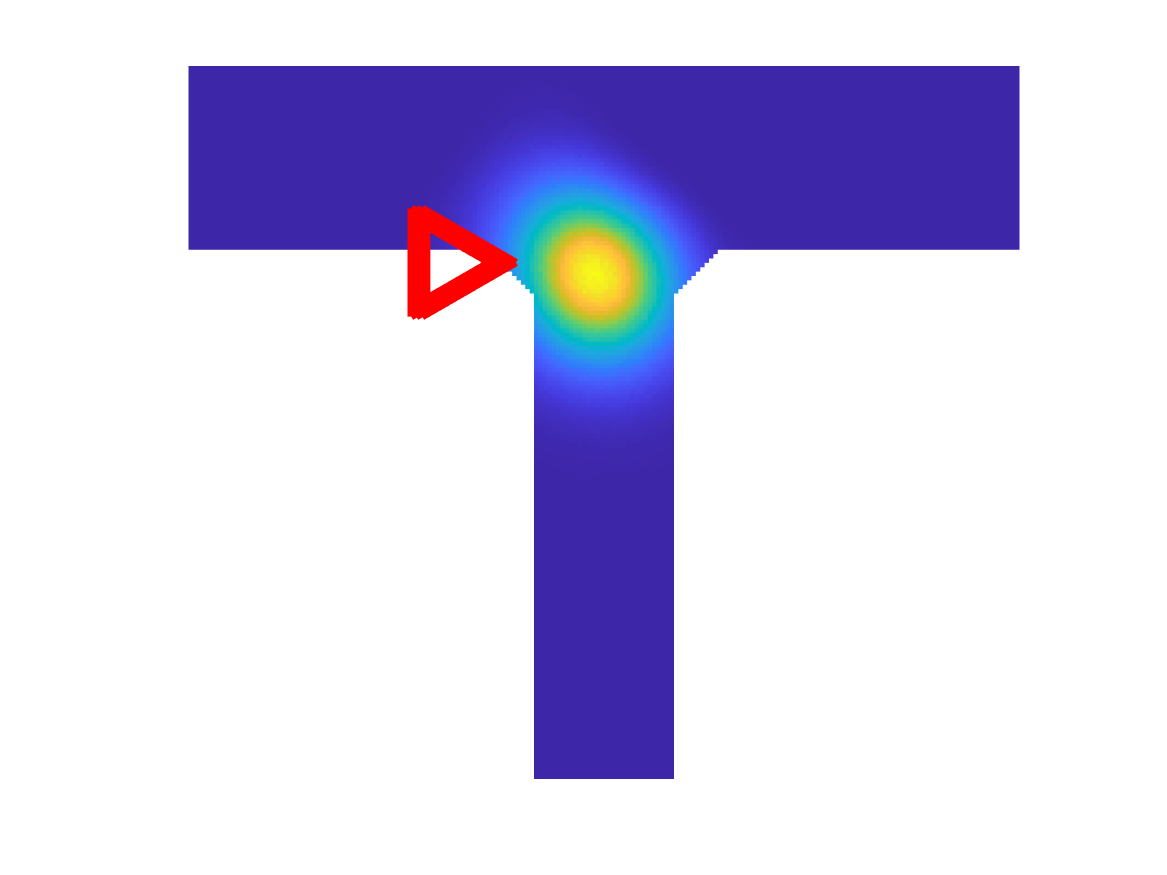

Supplement: Source code 1. [file elife-87055-code1.zip › code/fig5b_frames/257.bmp]

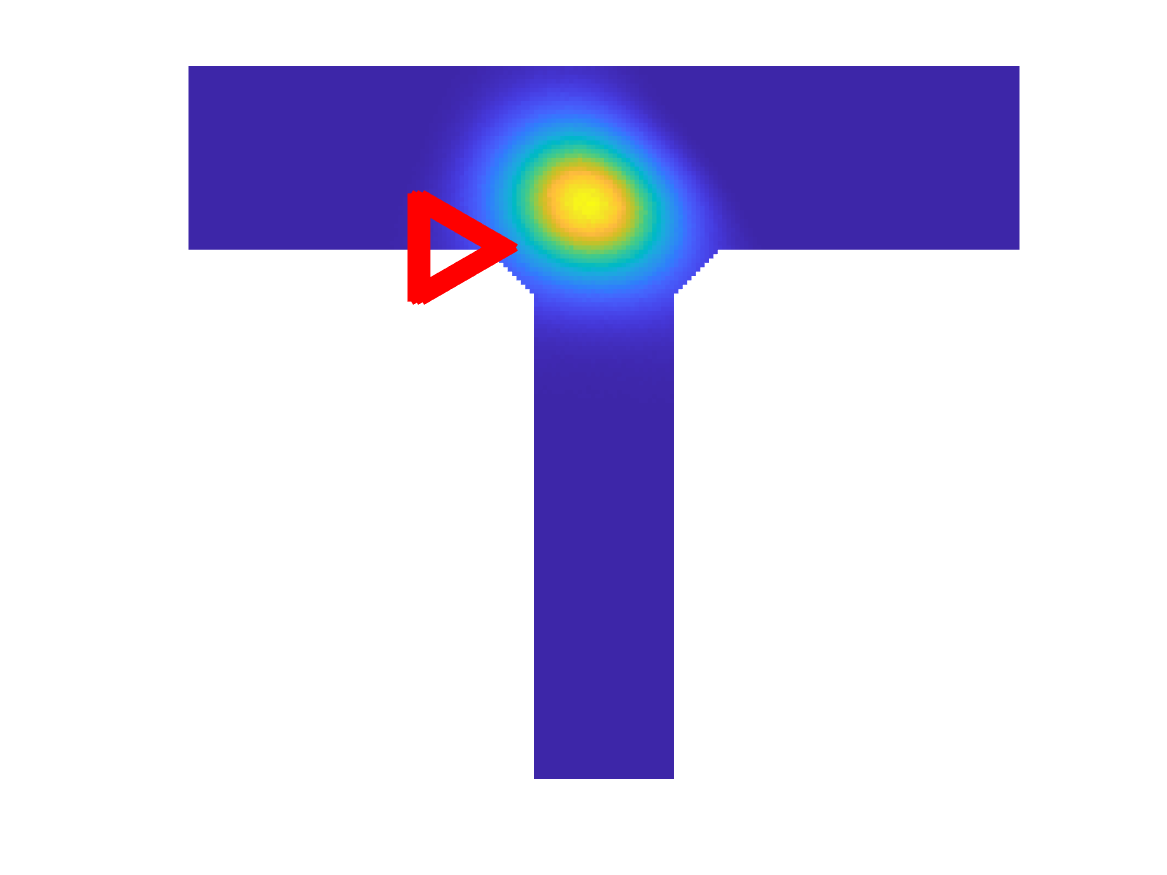

Supplement: Source code 1. [file elife-87055-code1.zip › code/fig5b_frames/280.bmp]

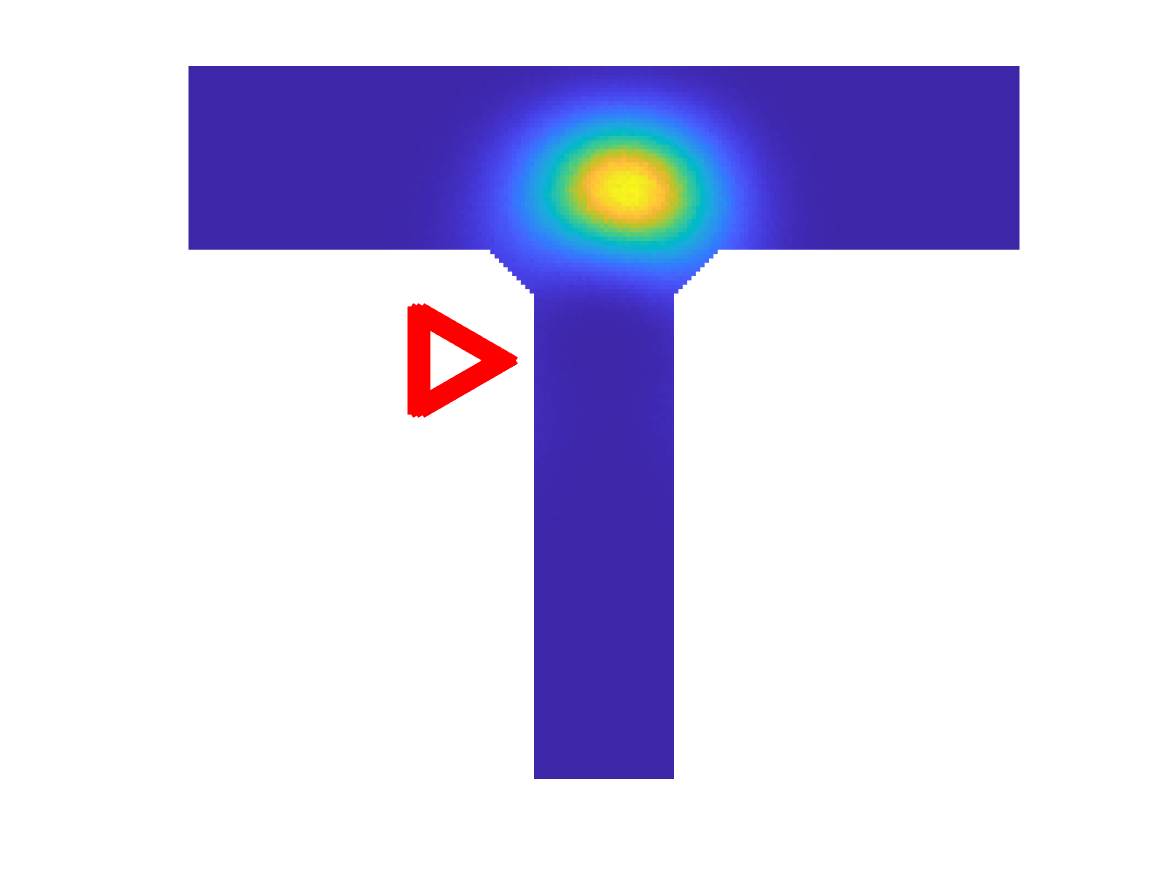

Supplement: Source code 1. [file elife-87055-code1.zip › code/fig5b_frames/109.bmp]

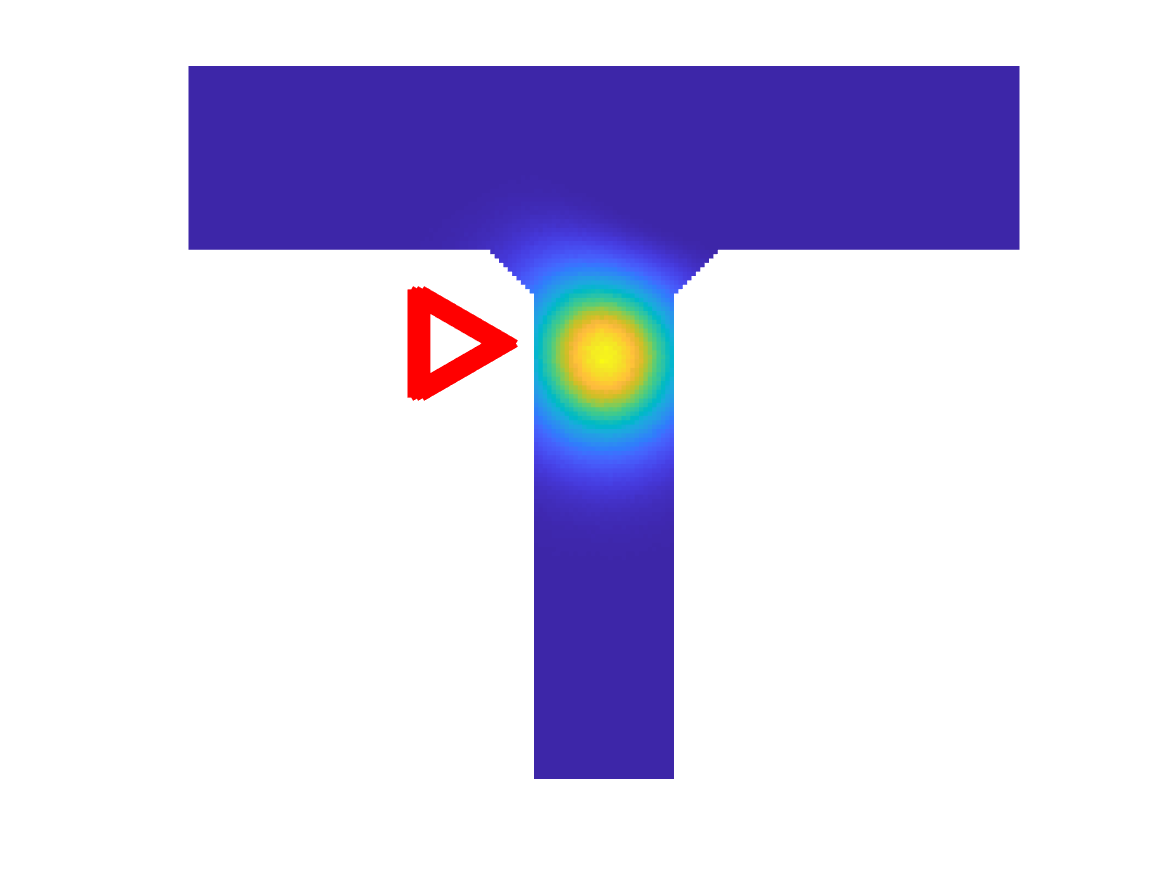

Supplement: Source code 1. [file elife-87055-code1.zip › code/fig5b_frames/135.bmp]

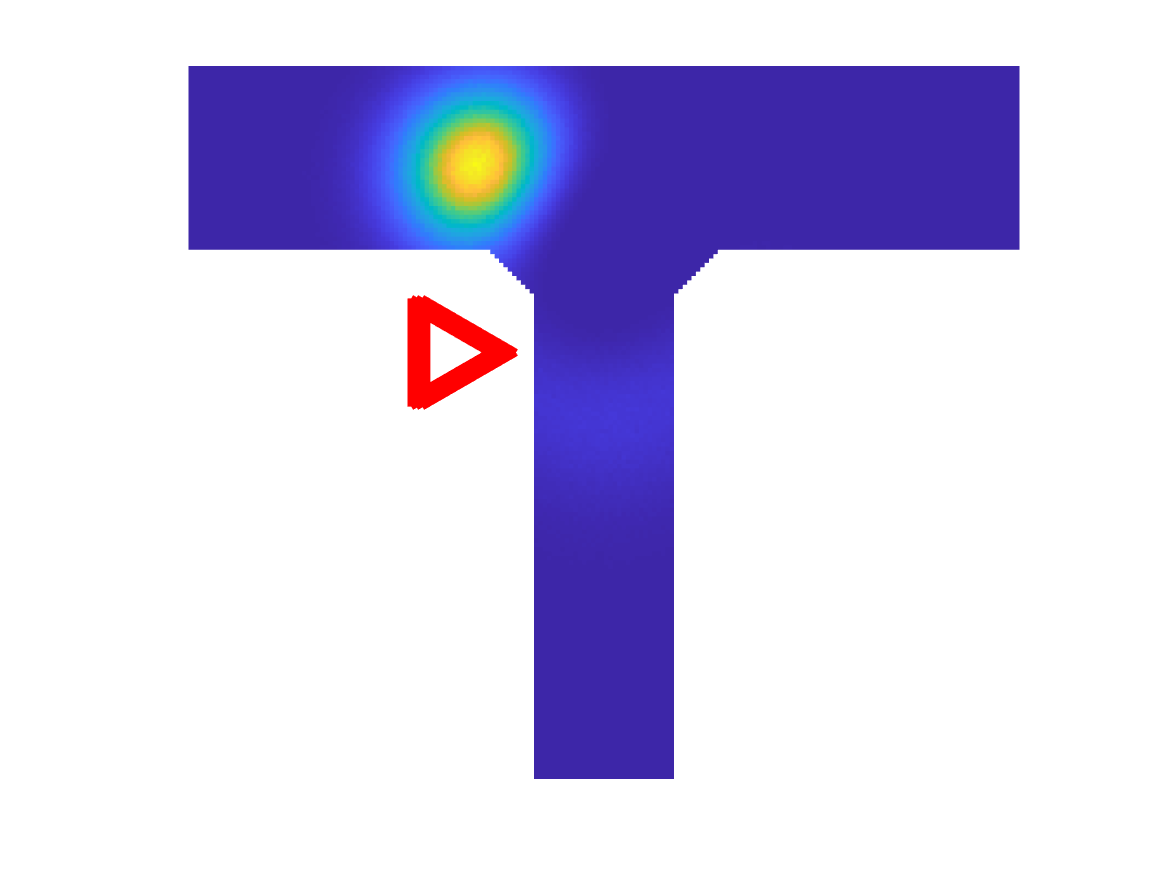

Supplement: Source code 1. [file elife-87055-code1.zip › code/fig5b_frames/121.bmp]

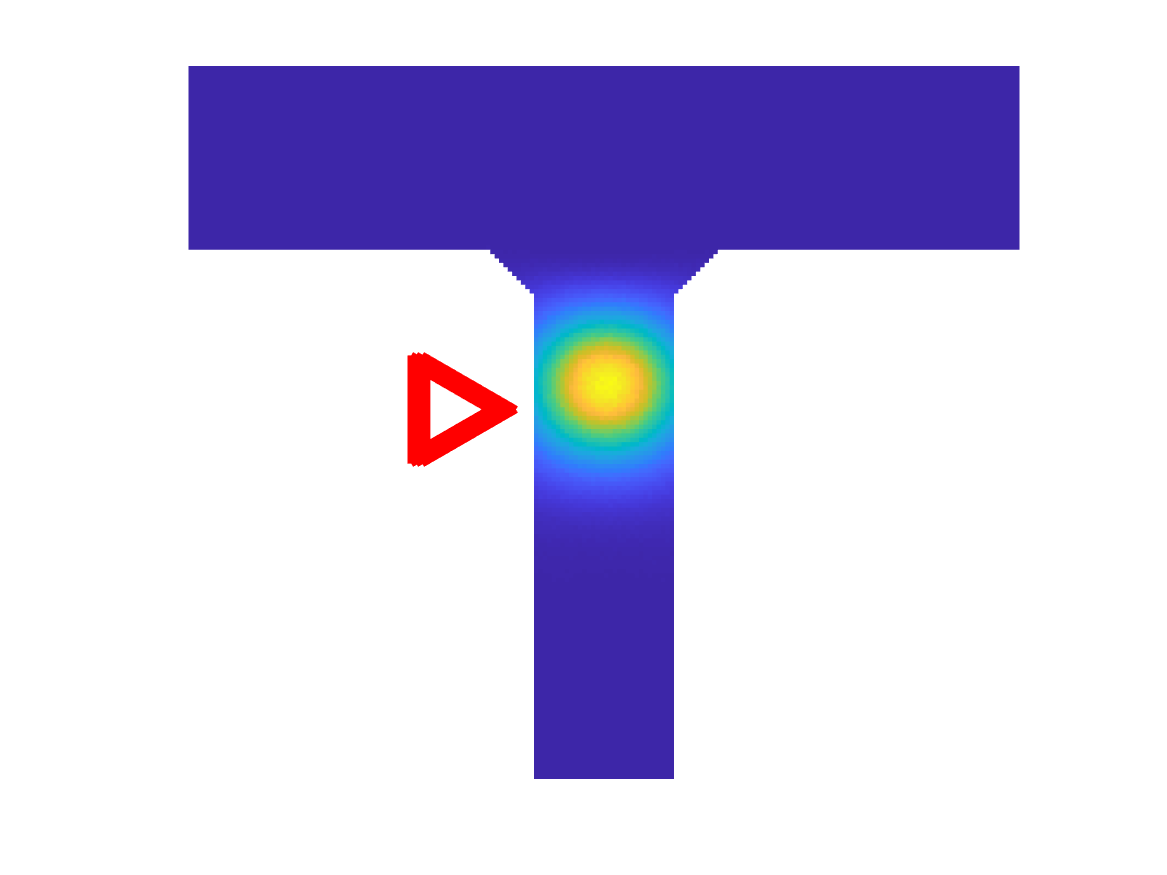

Supplement: Source code 1. [file elife-87055-code1.zip › code/fig5b_frames/35.bmp]

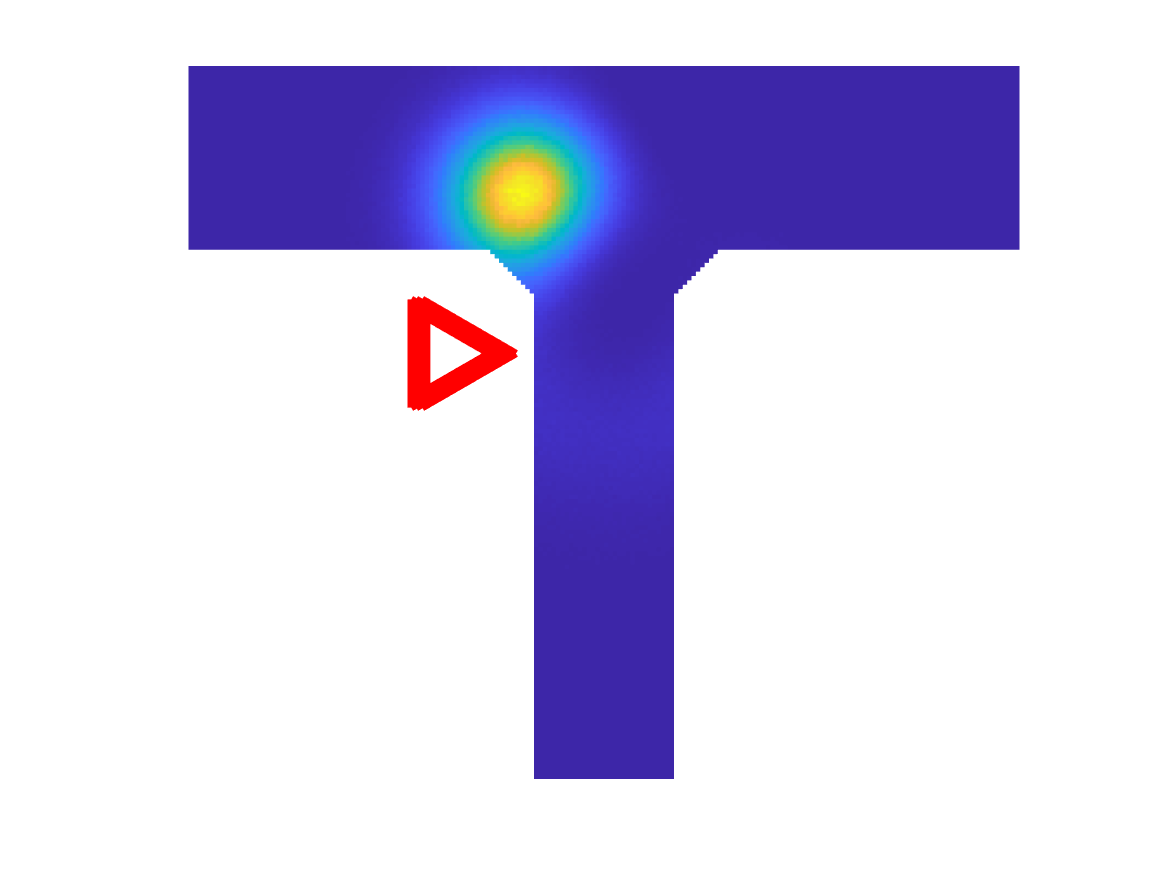

Supplement: Source code 1. [file elife-87055-code1.zip › code/fig5b_frames/120.bmp]

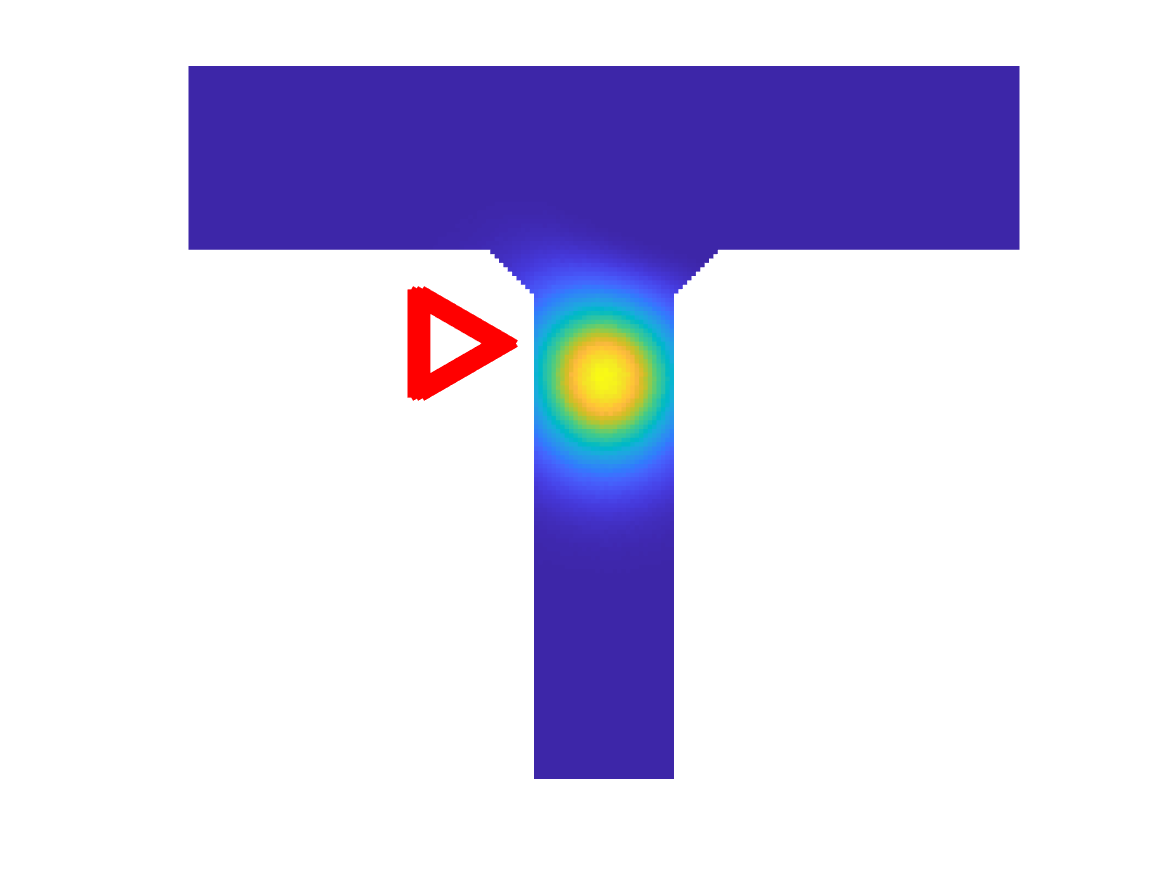

Supplement: Source code 1. [file elife-87055-code1.zip › code/fig5b_frames/134.bmp]

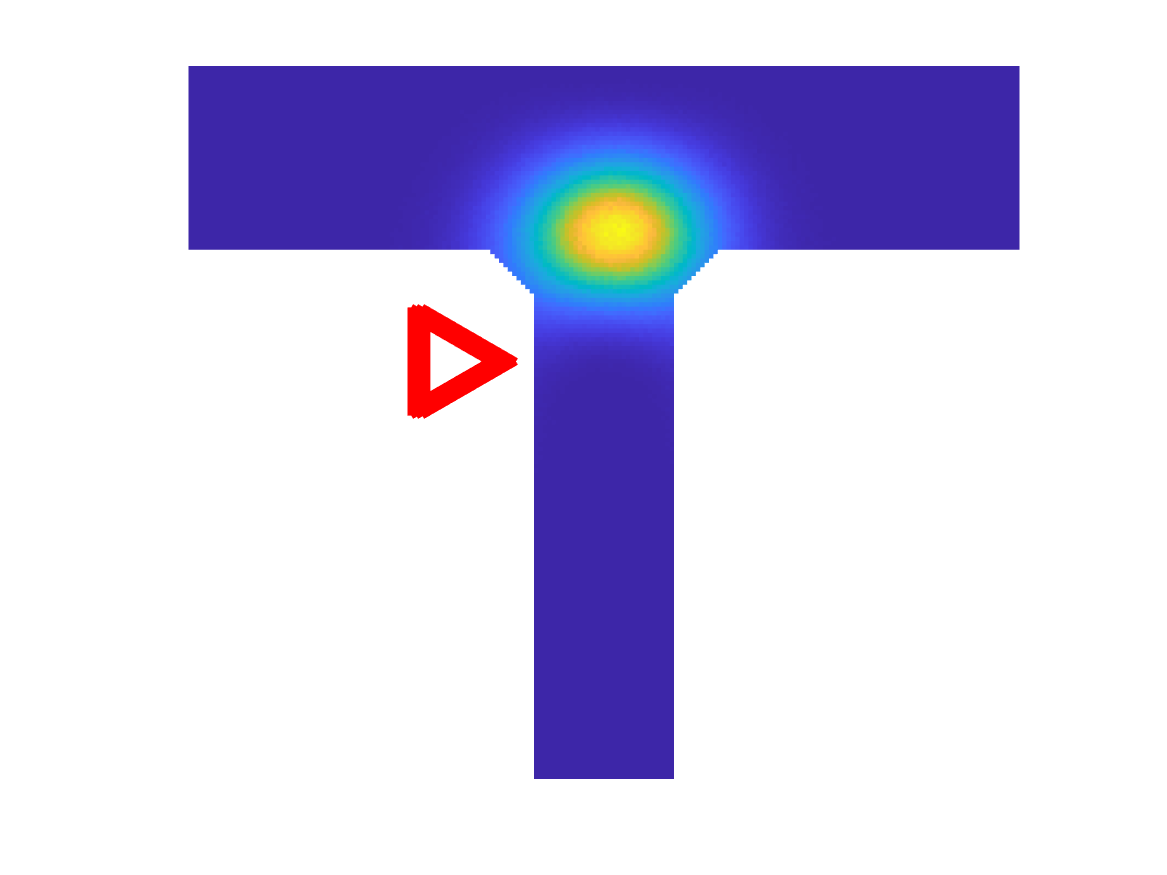

Supplement: Source code 1. [file elife-87055-code1.zip › code/fig5b_frames/108.bmp]

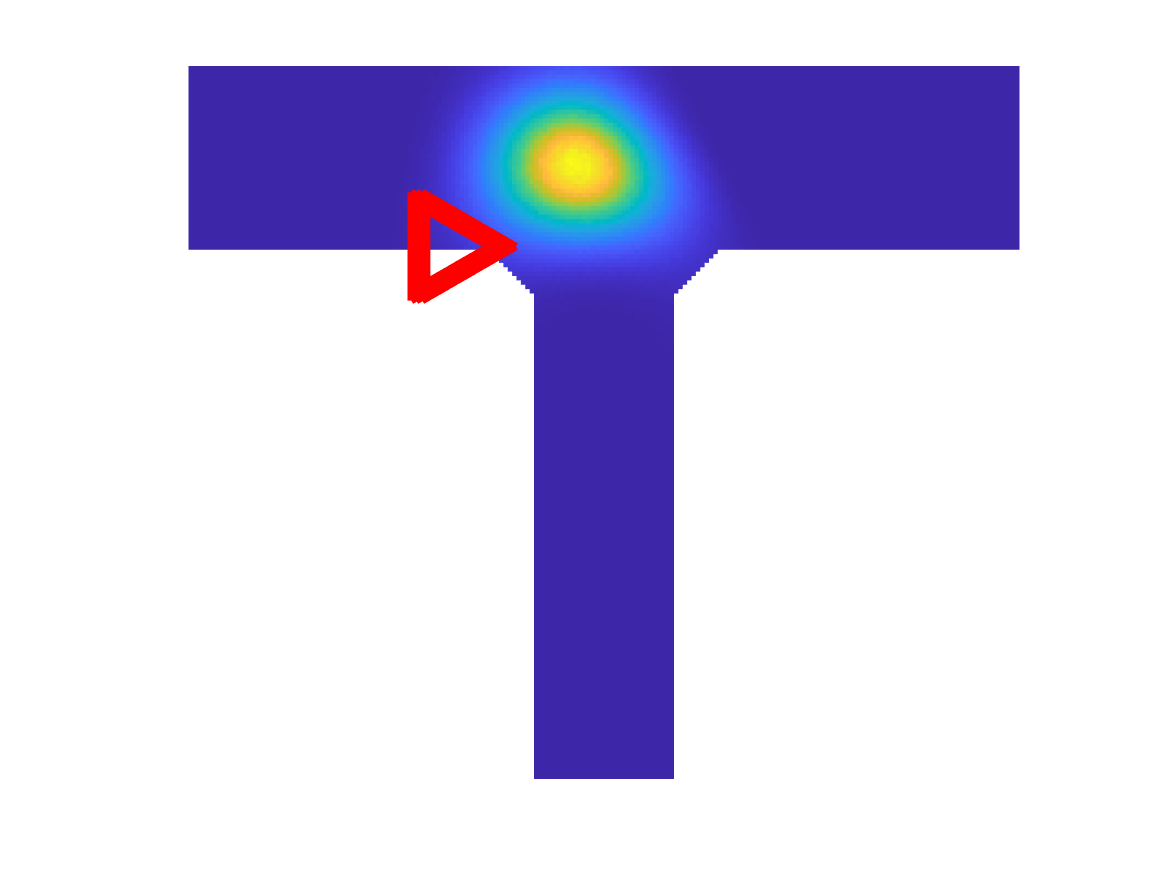

Supplement: Source code 1. [file elife-87055-code1.zip › code/fig5b_frames/281.bmp]

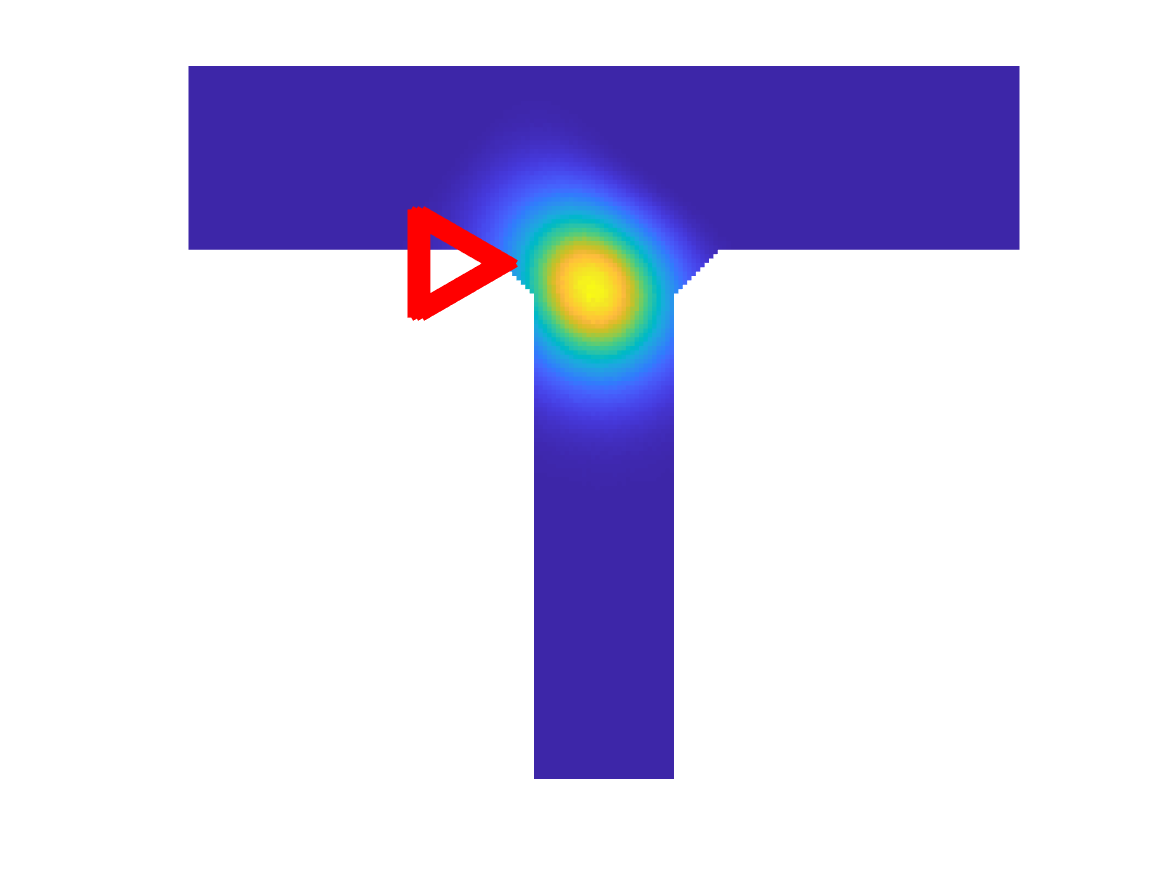

Supplement: Source code 1. [file elife-87055-code1.zip › code/fig5b_frames/256.bmp]

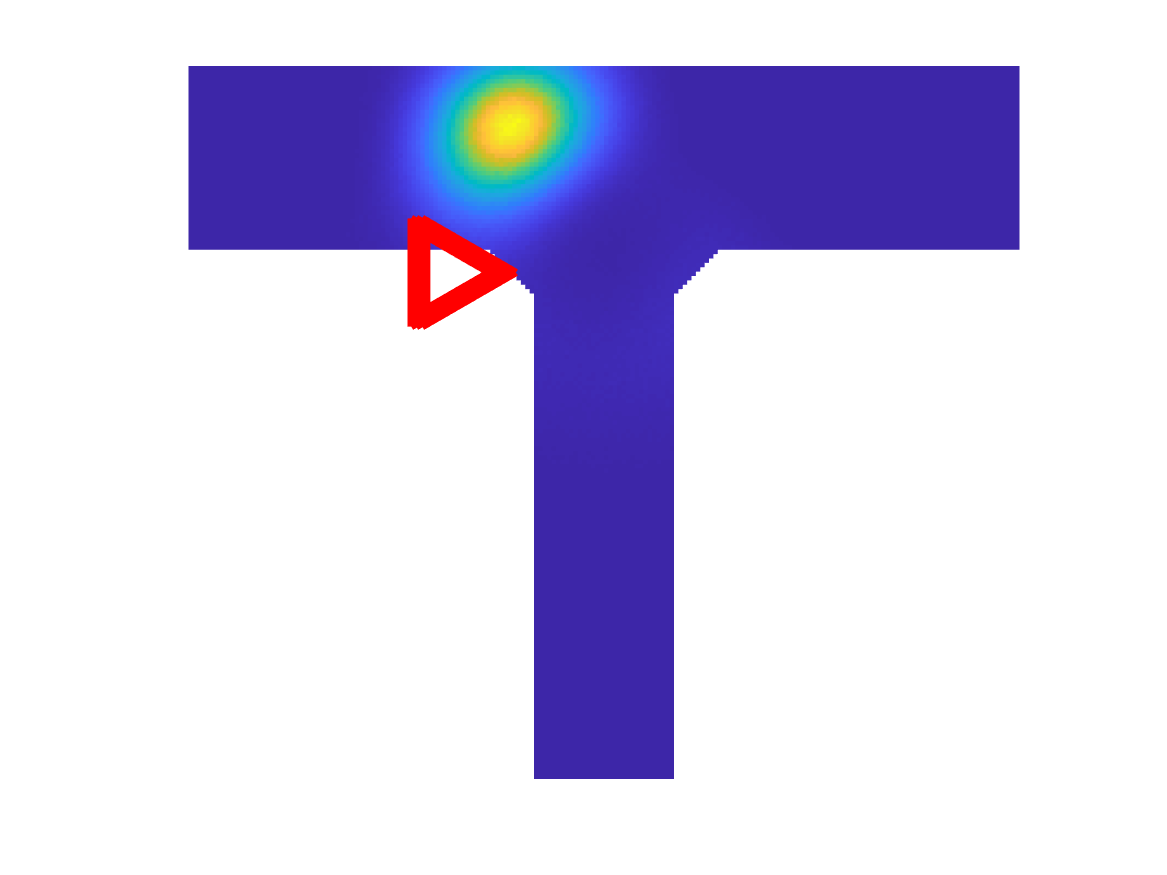

Supplement: Source code 1. [file elife-87055-code1.zip › code/fig5b_frames/242.bmp]

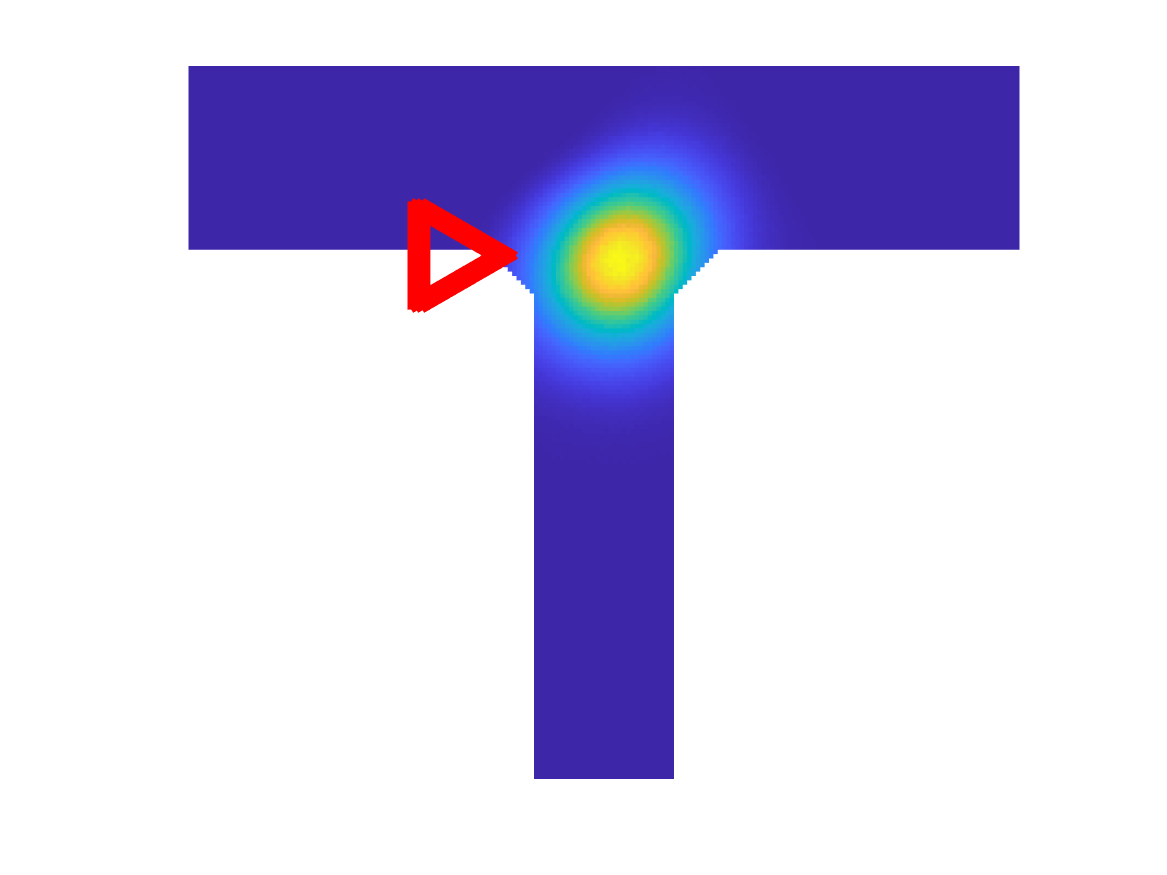

Supplement: Source code 1. [file elife-87055-code1.zip › code/fig5b_frames/268.bmp]

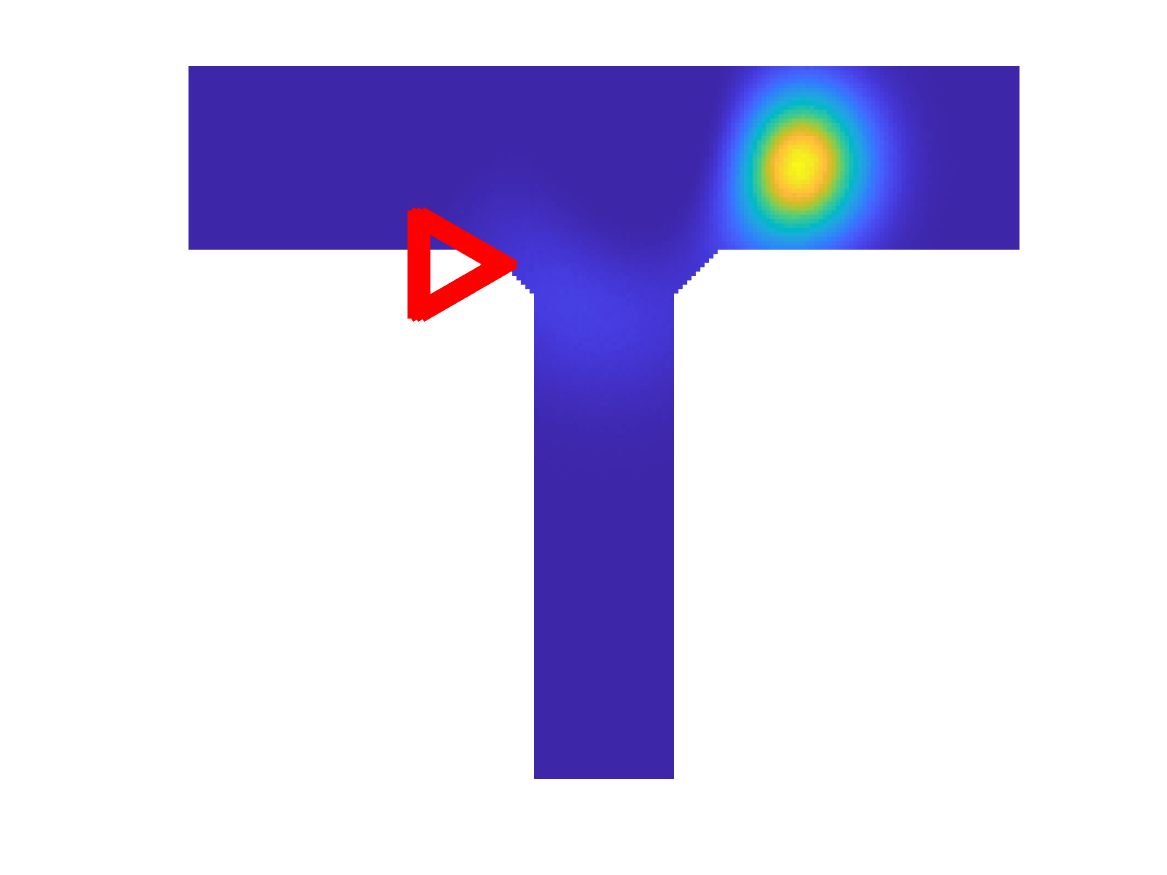

Supplement: Source code 1. [file elife-87055-code1.zip › code/fig5b_frames/254.bmp]

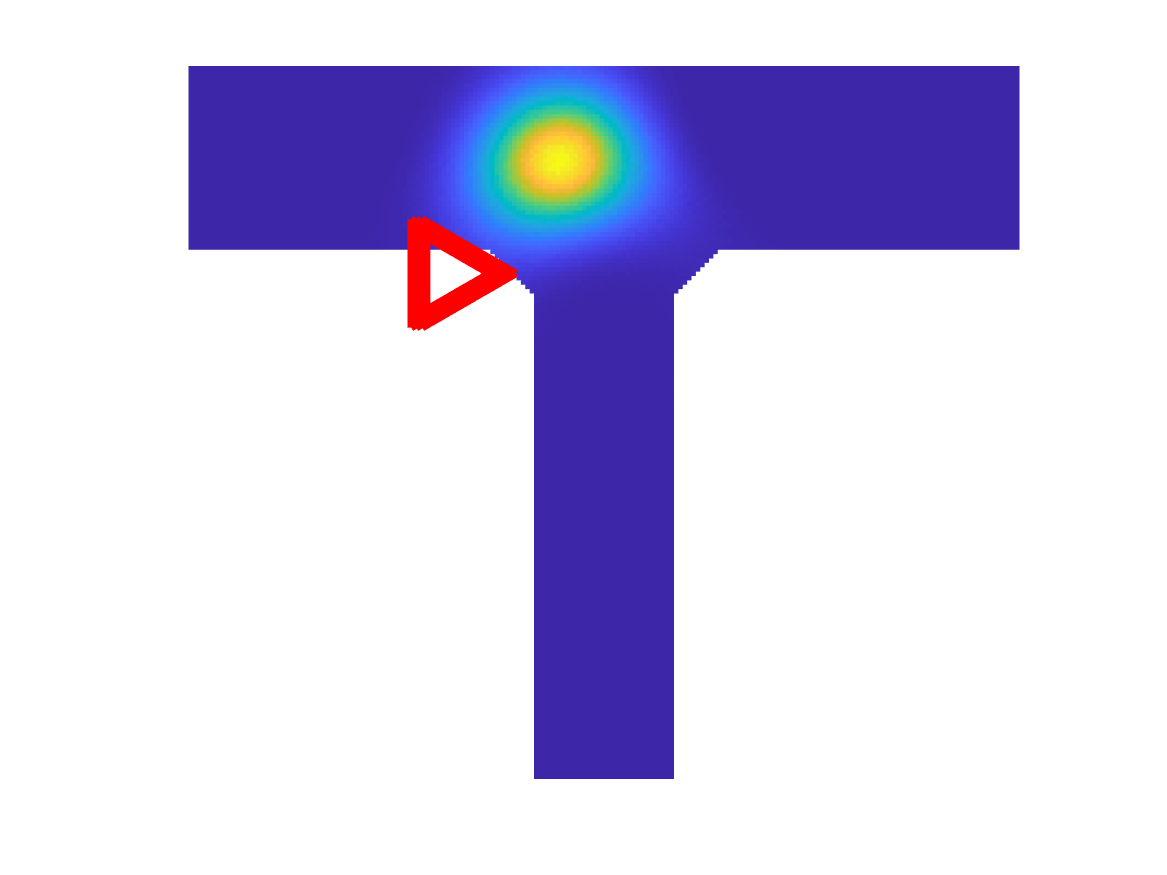

Supplement: Source code 1. [file elife-87055-code1.zip › code/fig5b_frames/240.bmp]

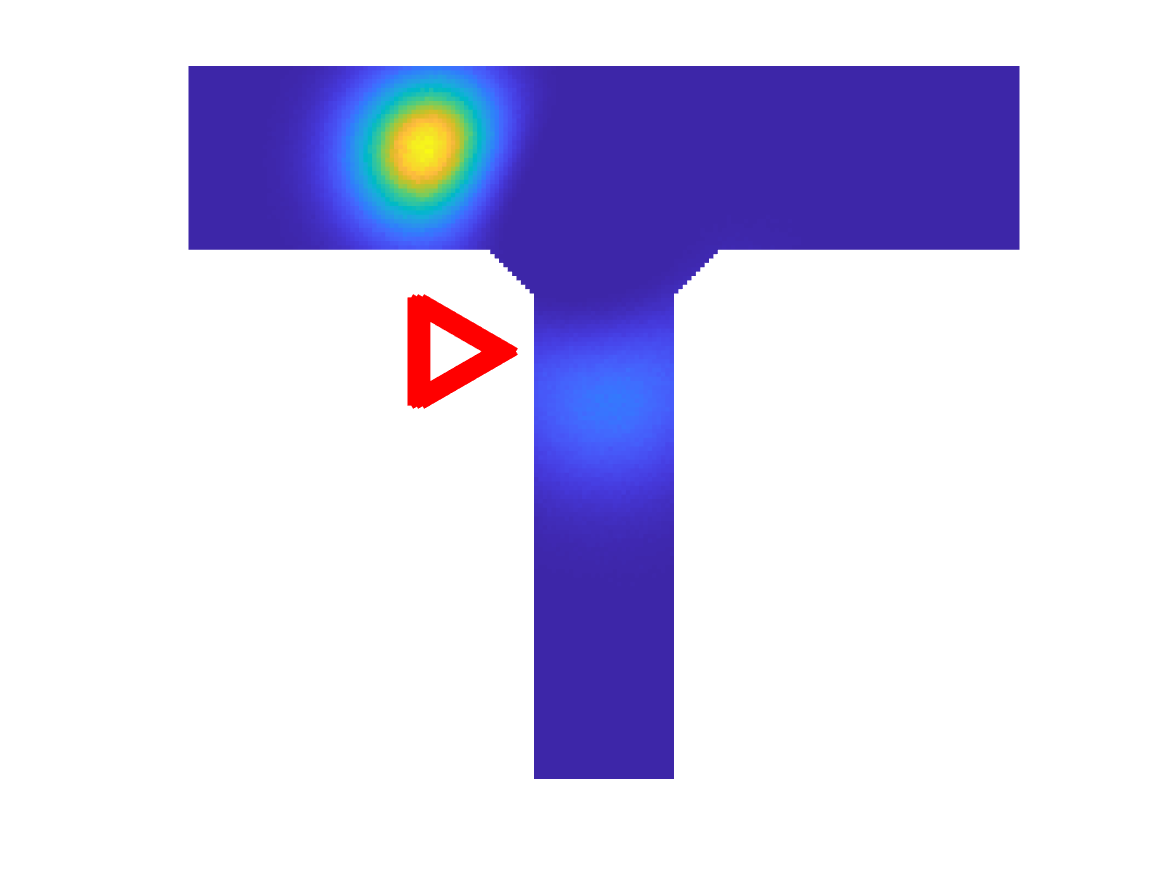

Supplement: Source code 1. [file elife-87055-code1.zip › code/fig5b_frames/122.bmp]

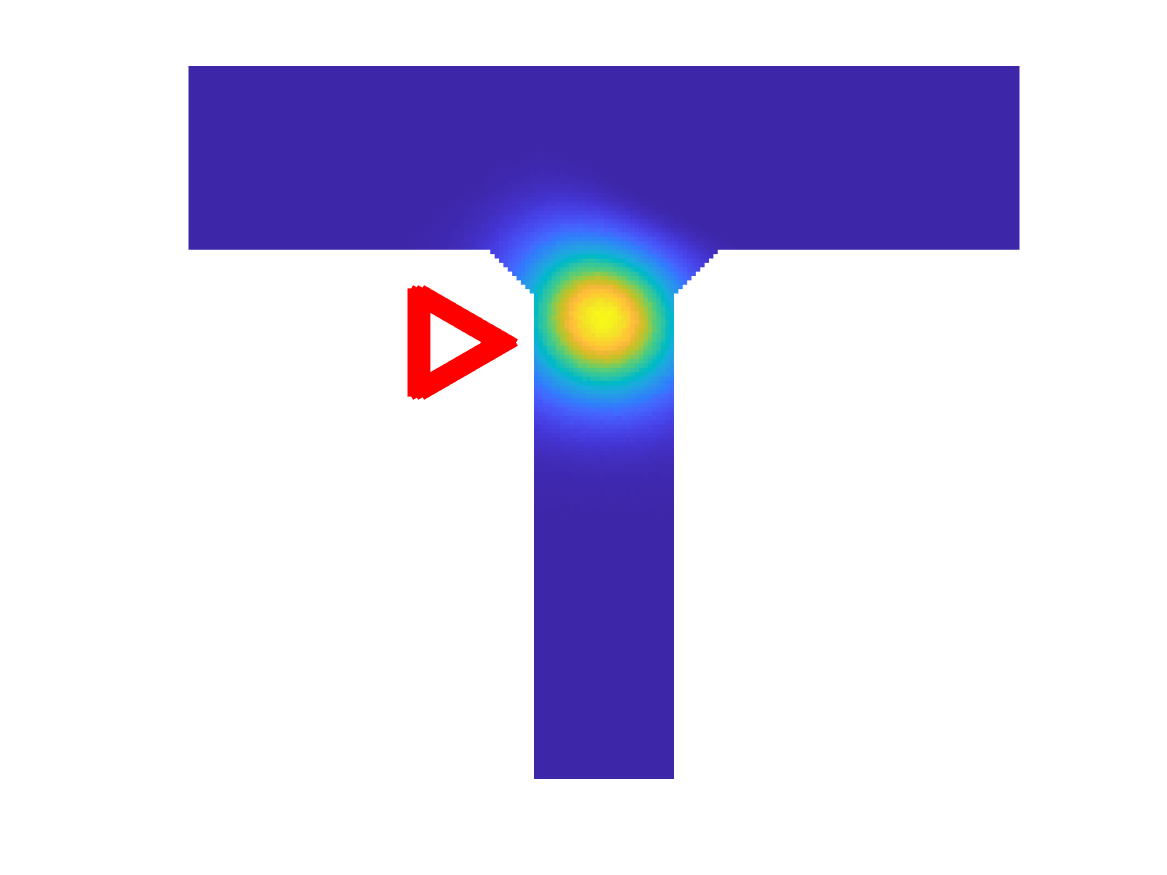

Supplement: Source code 1. [file elife-87055-code1.zip › code/fig5b_frames/136.bmp]

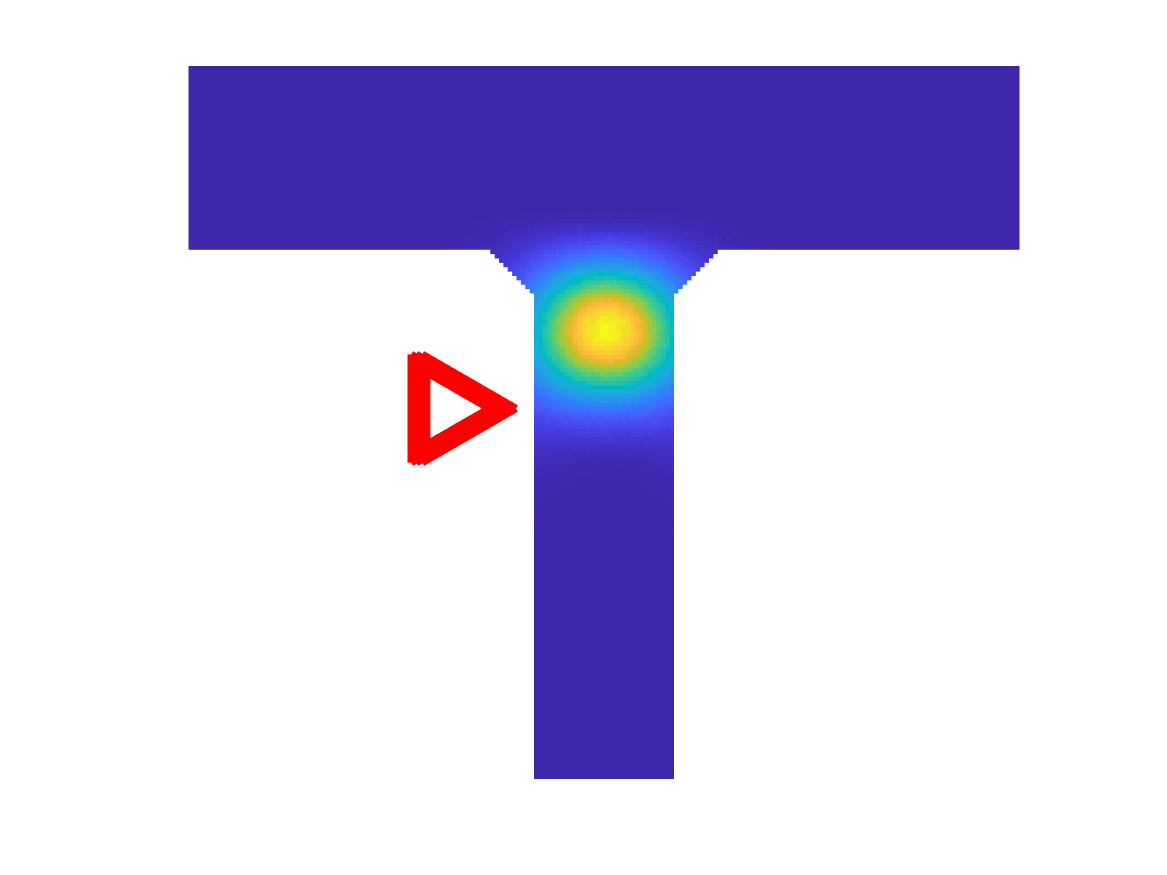

Supplement: Source code 1. [file elife-87055-code1.zip › code/fig5b_frames/37.bmp]

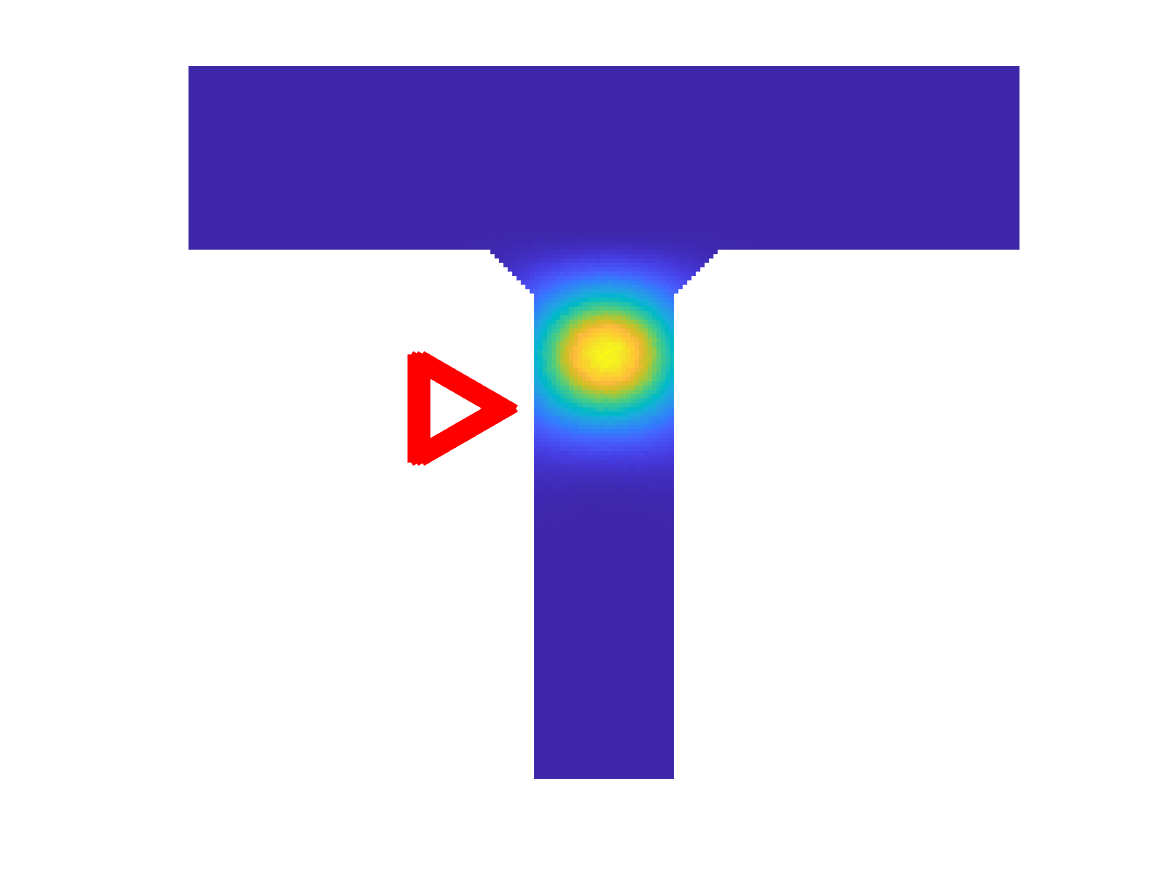

Supplement: Source code 1. [file elife-87055-code1.zip › code/fig5b_frames/36.bmp]

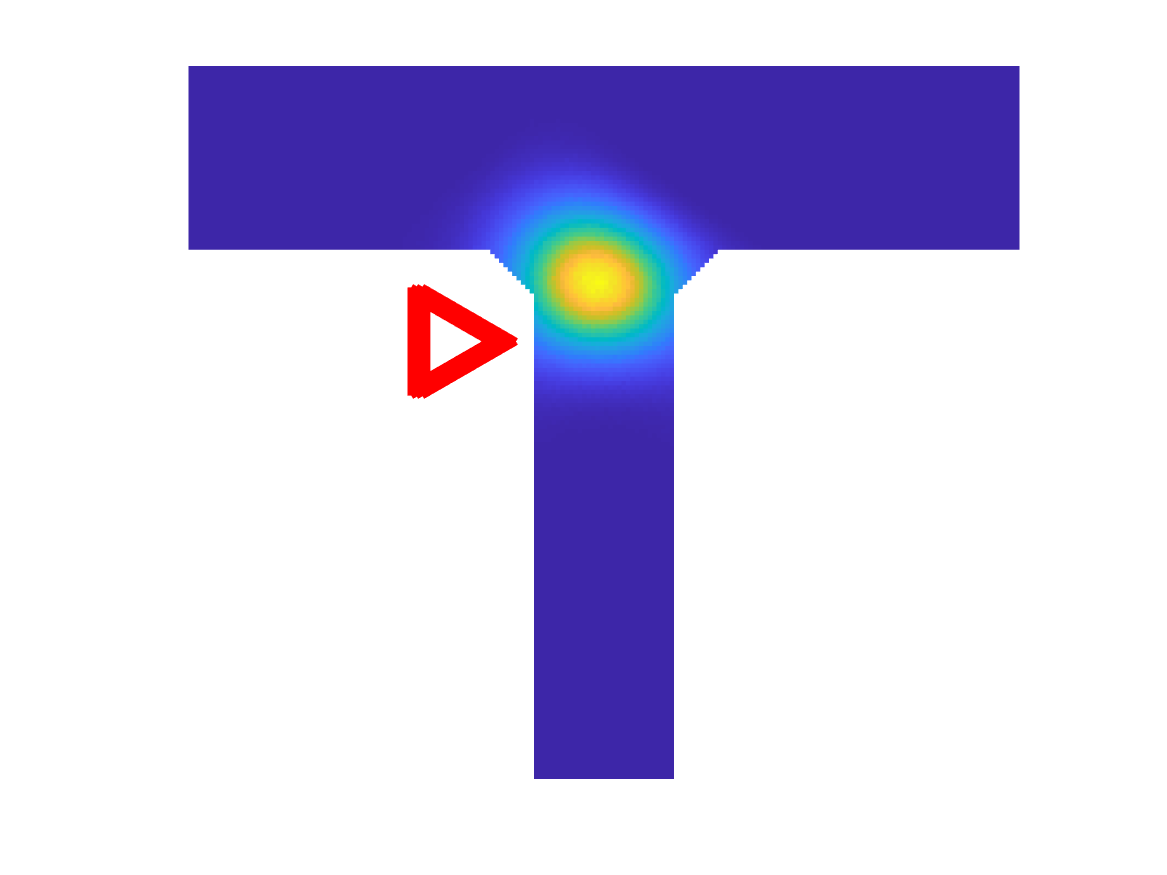

Supplement: Source code 1. [file elife-87055-code1.zip › code/fig5b_frames/137.bmp]

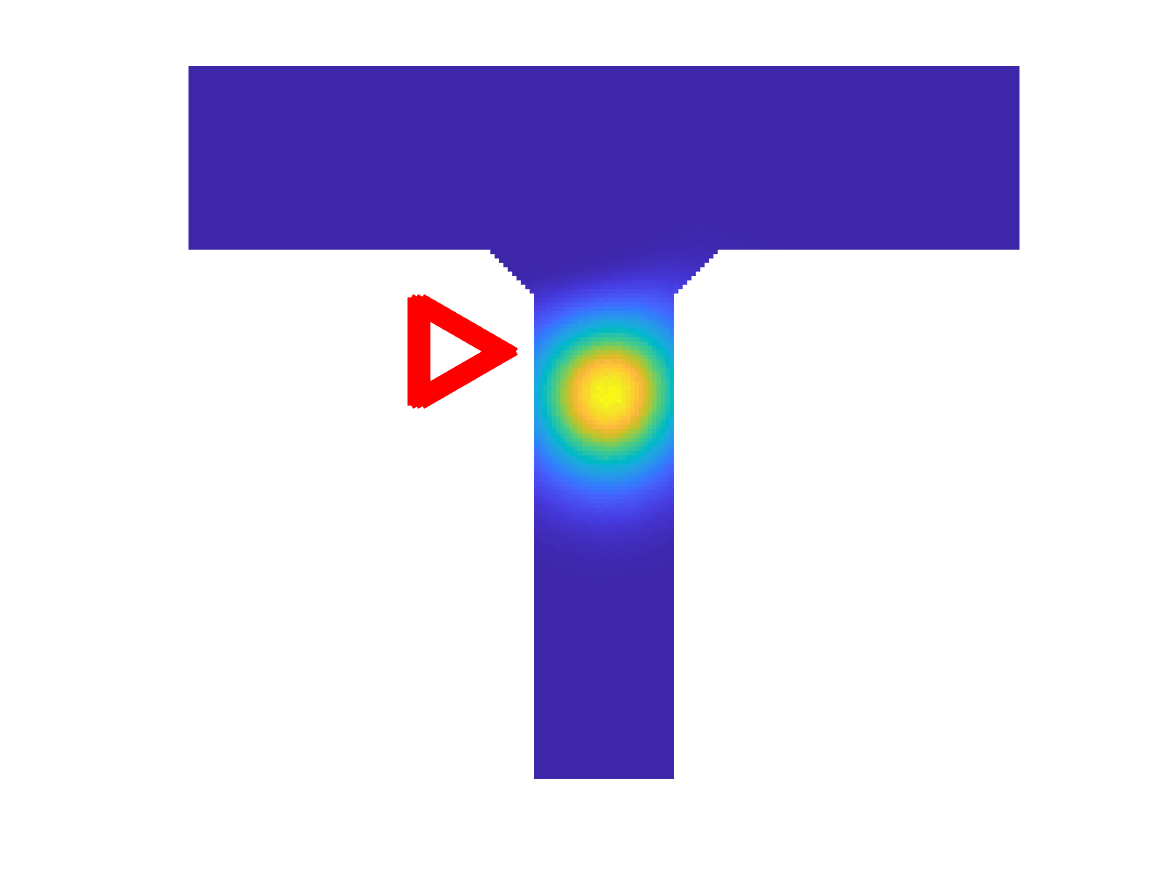

Supplement: Source code 1. [file elife-87055-code1.zip › code/fig5b_frames/123.bmp]

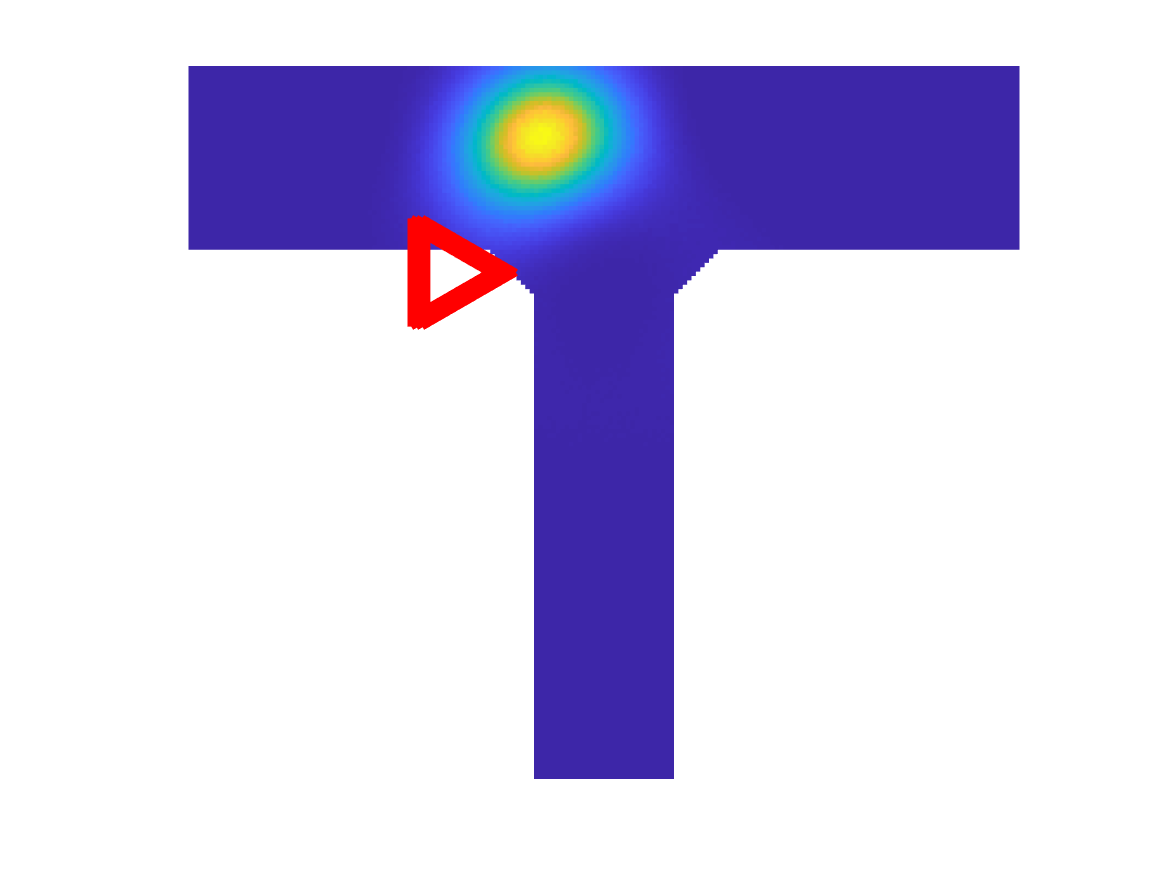

Supplement: Source code 1. [file elife-87055-code1.zip › code/fig5b_frames/241.bmp]

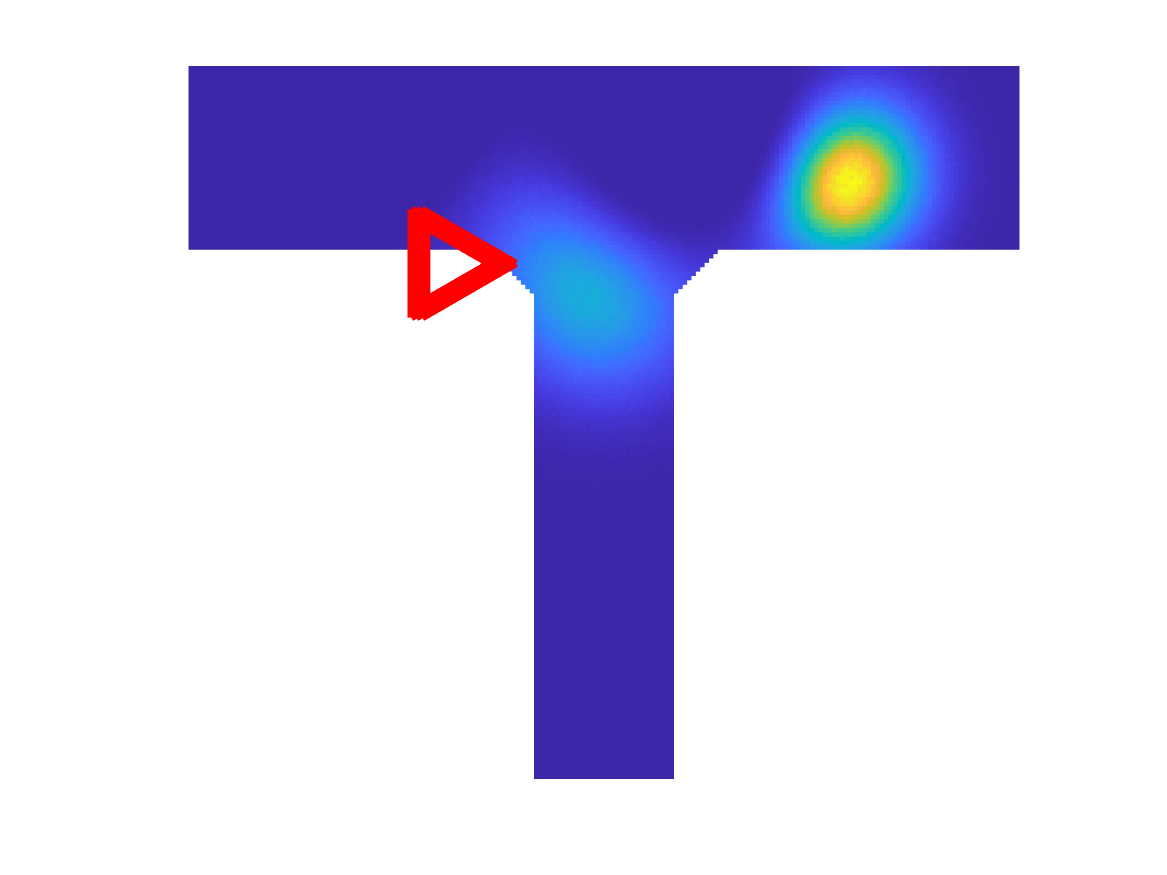

Supplement: Source code 1. [file elife-87055-code1.zip › code/fig5b_frames/255.bmp]

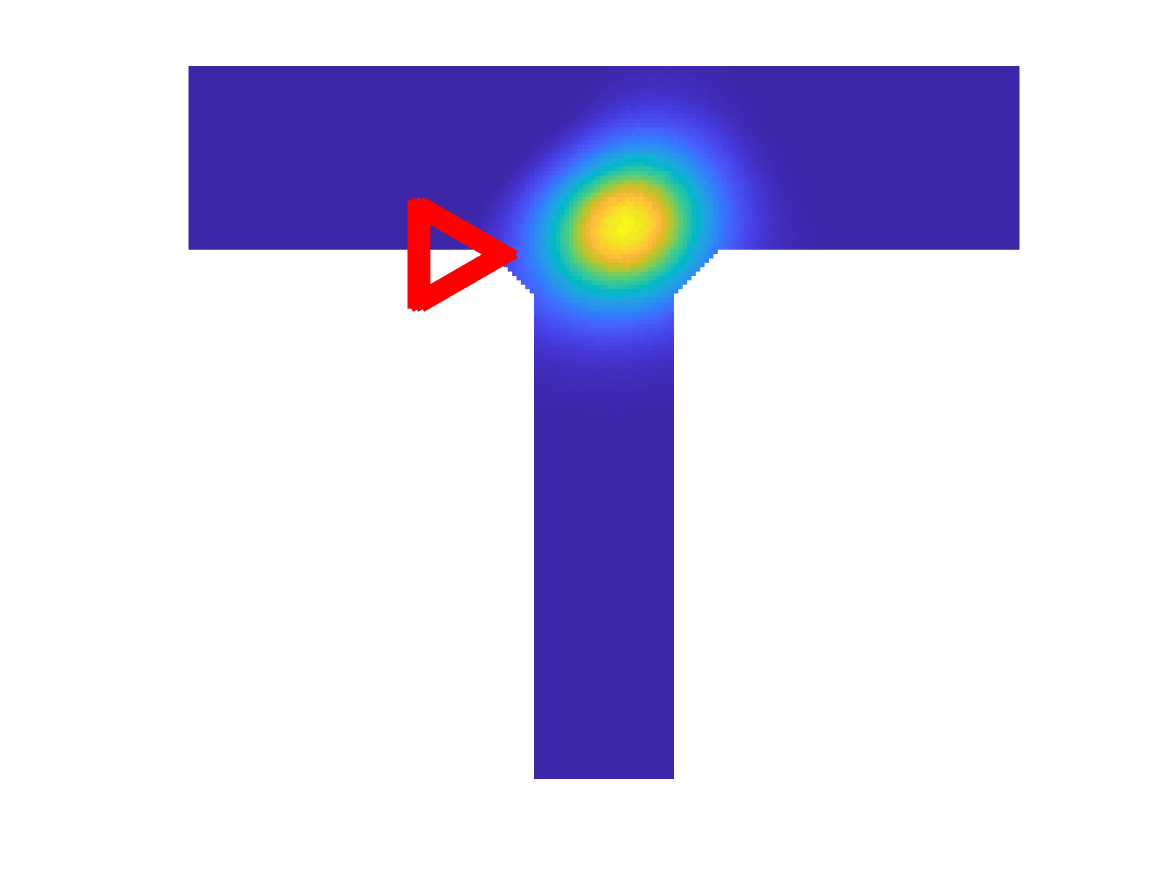

Supplement: Source code 1. [file elife-87055-code1.zip › code/fig5b_frames/269.bmp]

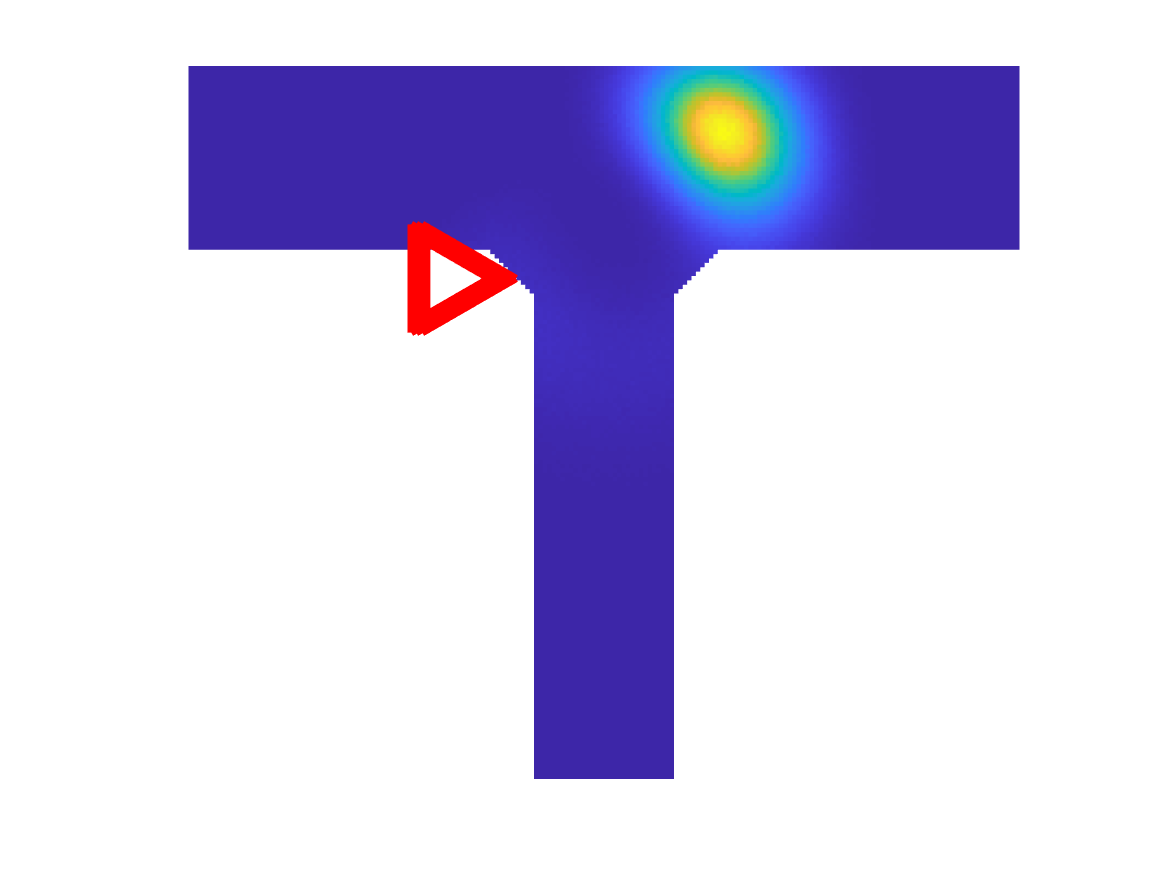

Supplement: Source code 1. [file elife-87055-code1.zip › code/fig5b_frames/232.bmp]

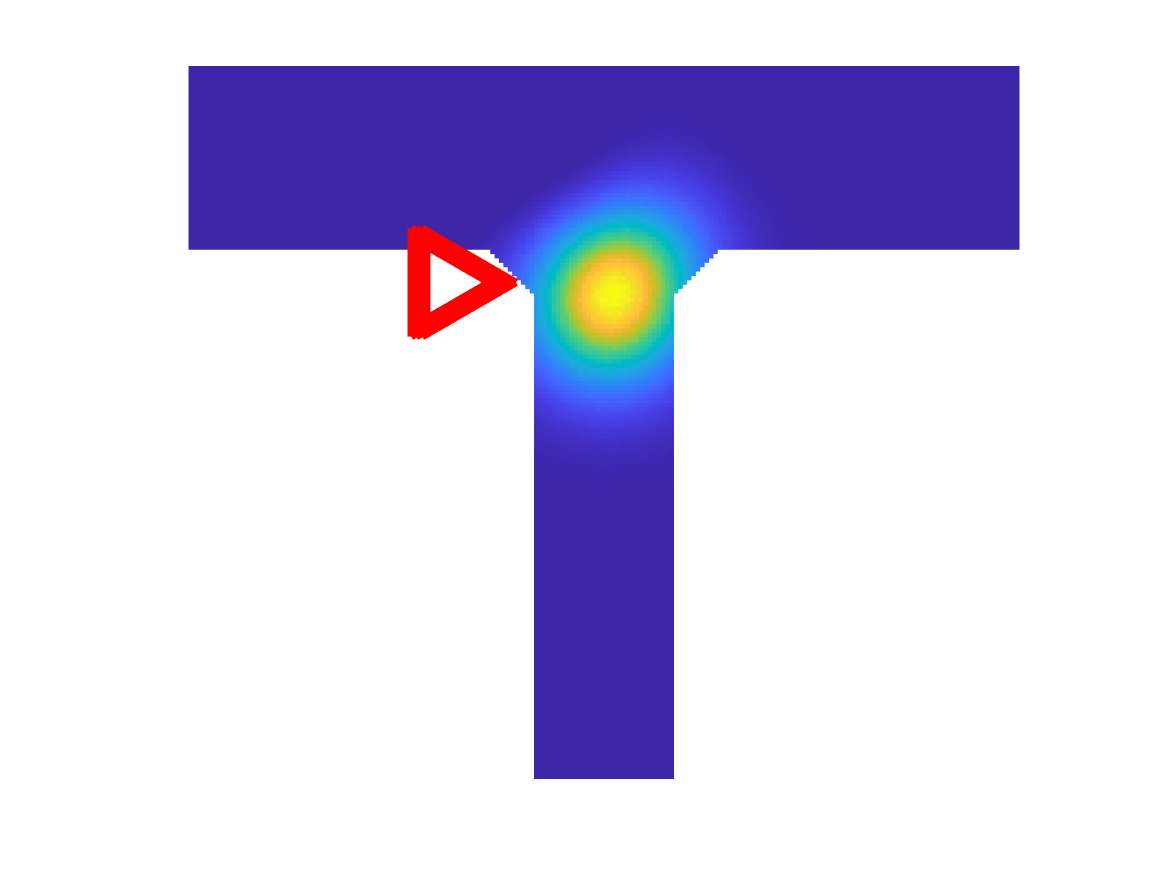

Supplement: Source code 1. [file elife-87055-code1.zip › code/fig5b_frames/226.bmp]

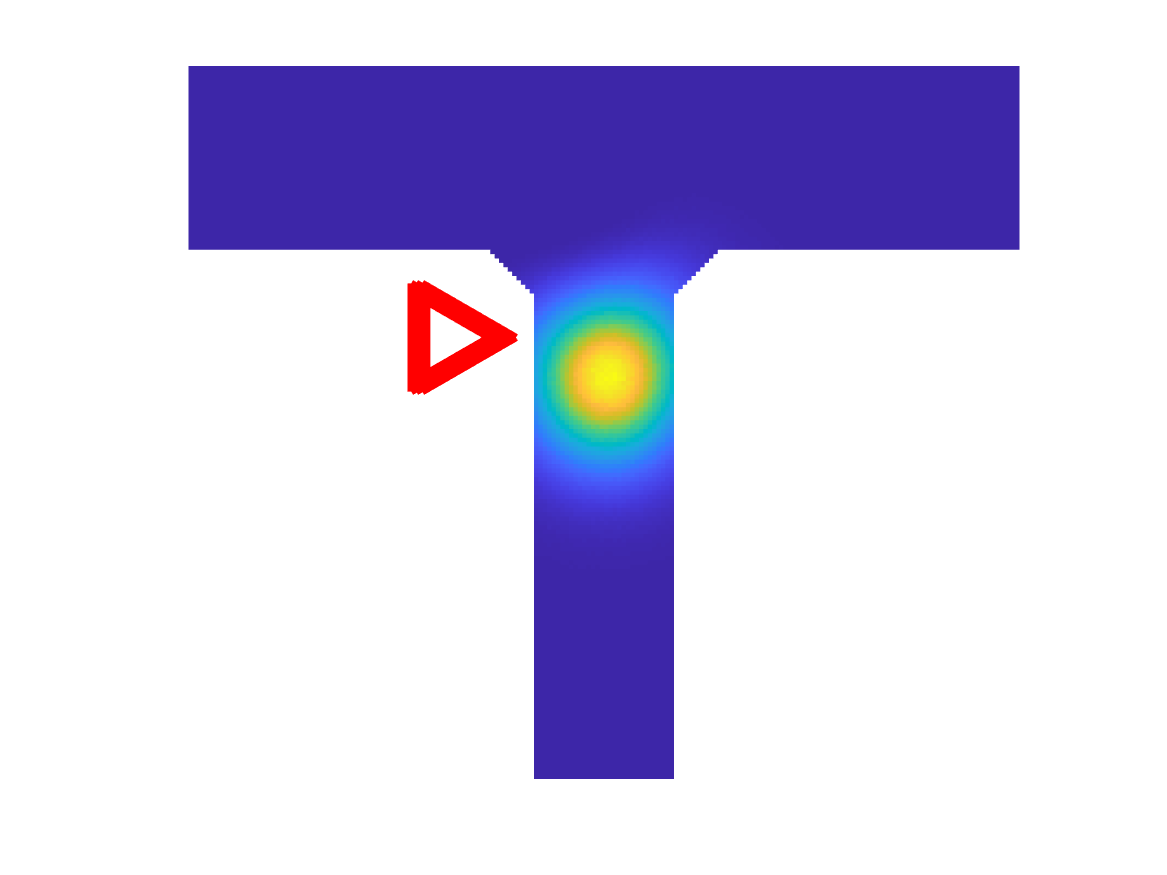

Supplement: Source code 1. [file elife-87055-code1.zip › code/fig5b_frames/144.bmp]

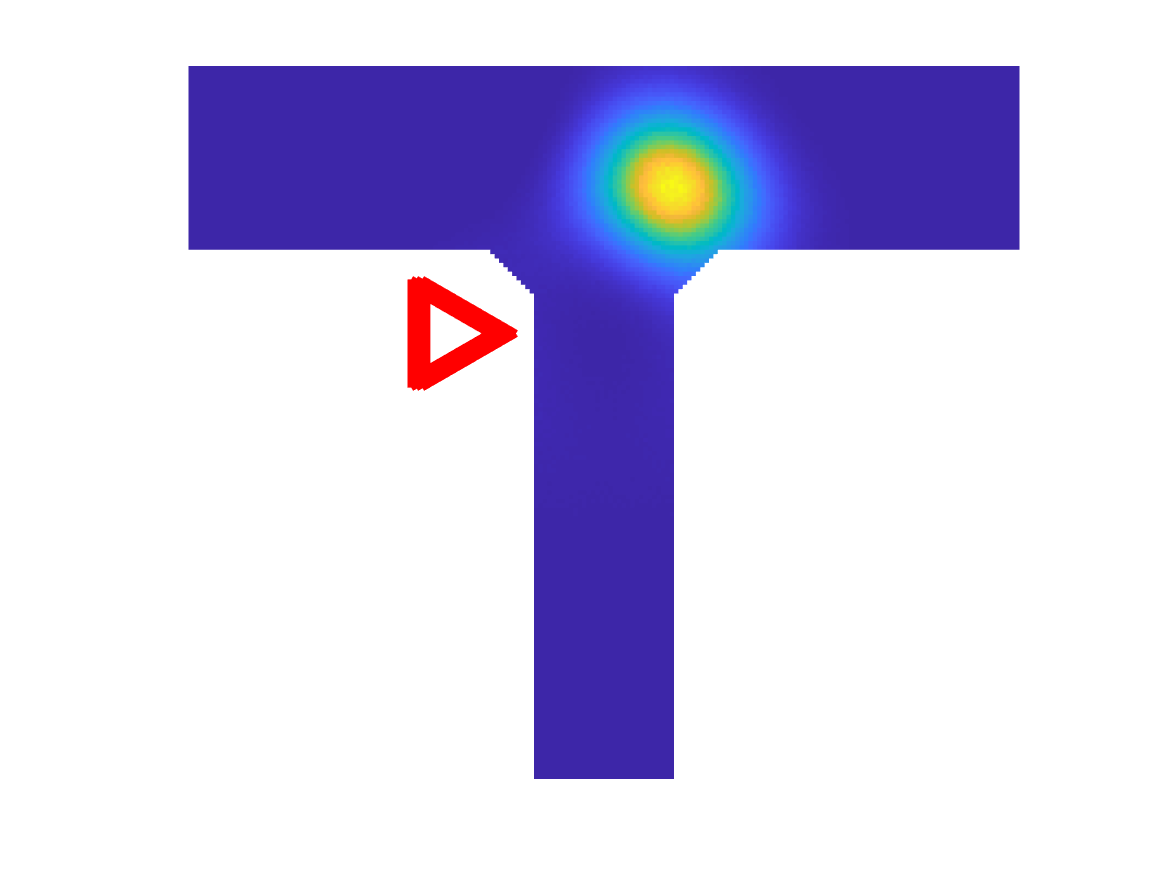

Supplement: Source code 1. [file elife-87055-code1.zip › code/fig5b_frames/150.bmp]

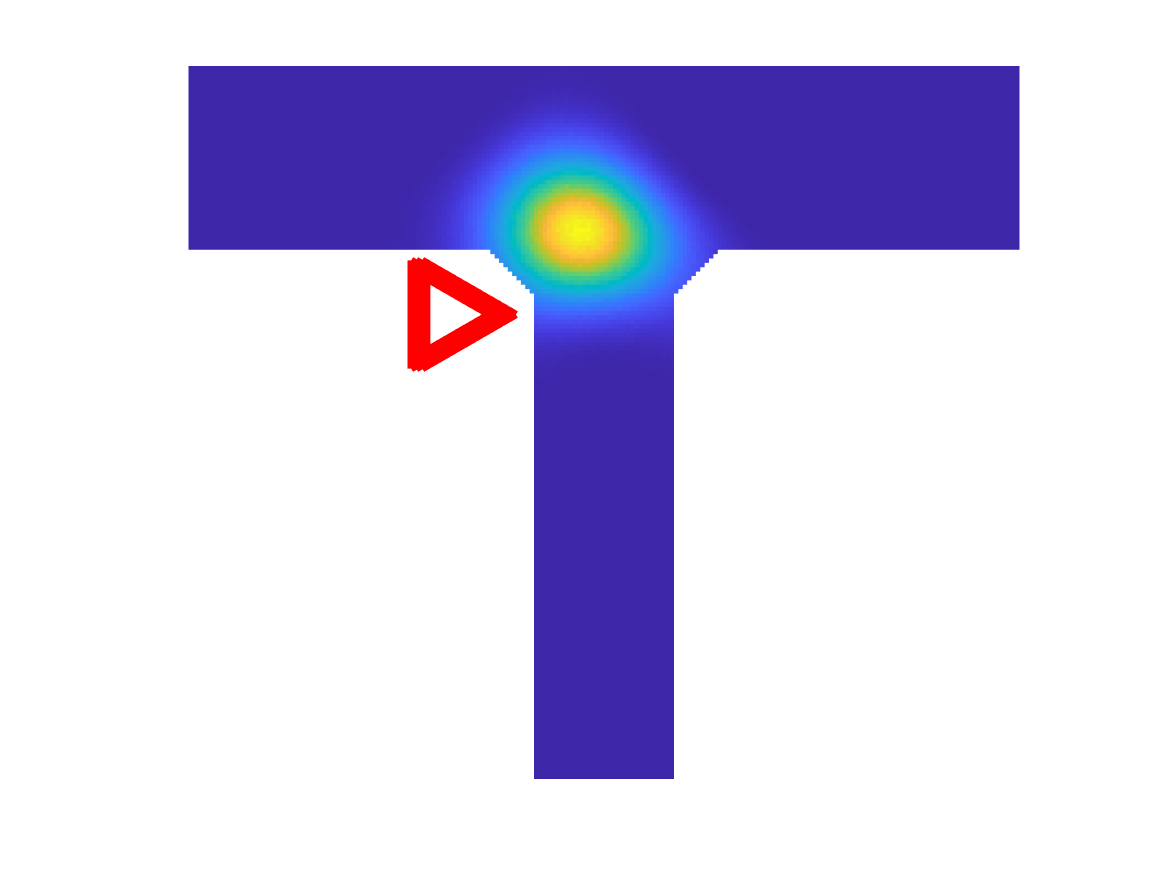

Supplement: Source code 1. [file elife-87055-code1.zip › code/fig5b_frames/178.bmp]

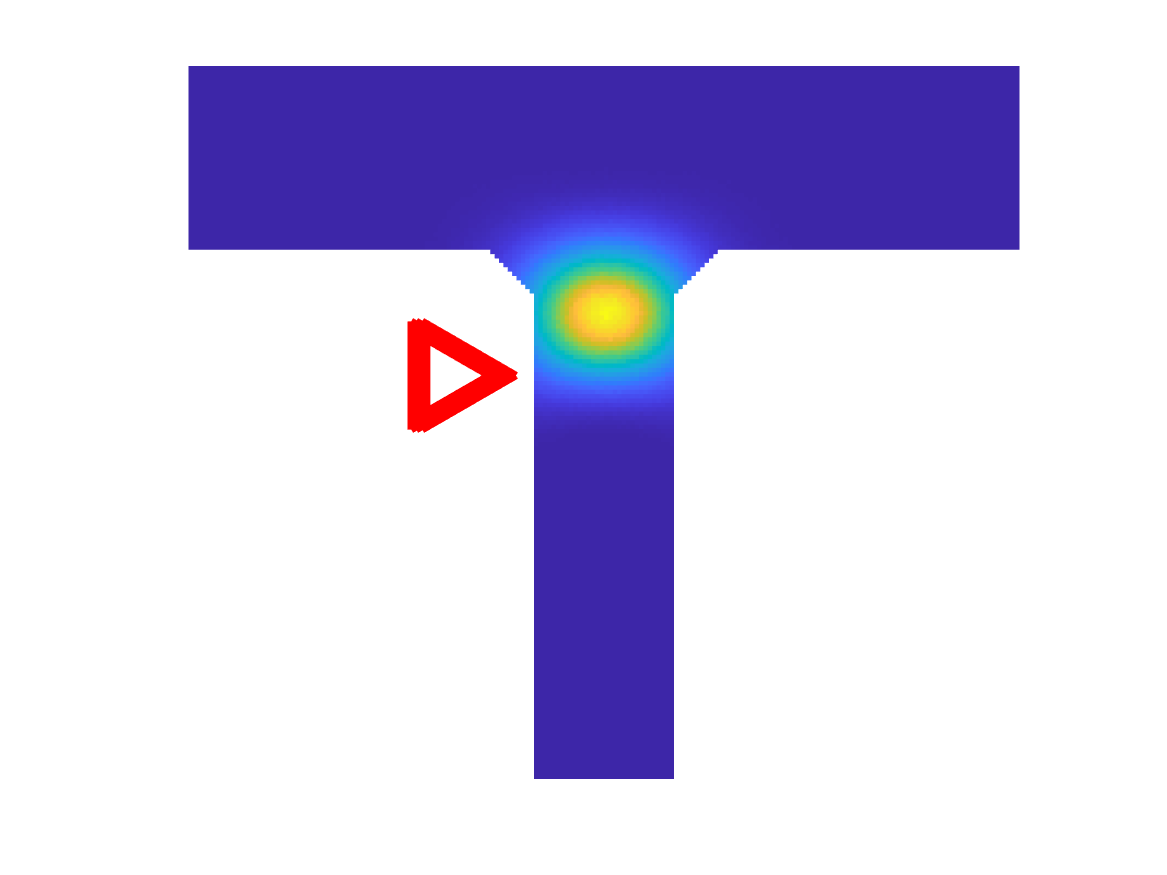

Supplement: Source code 1. [file elife-87055-code1.zip › code/fig5b_frames/86.bmp]

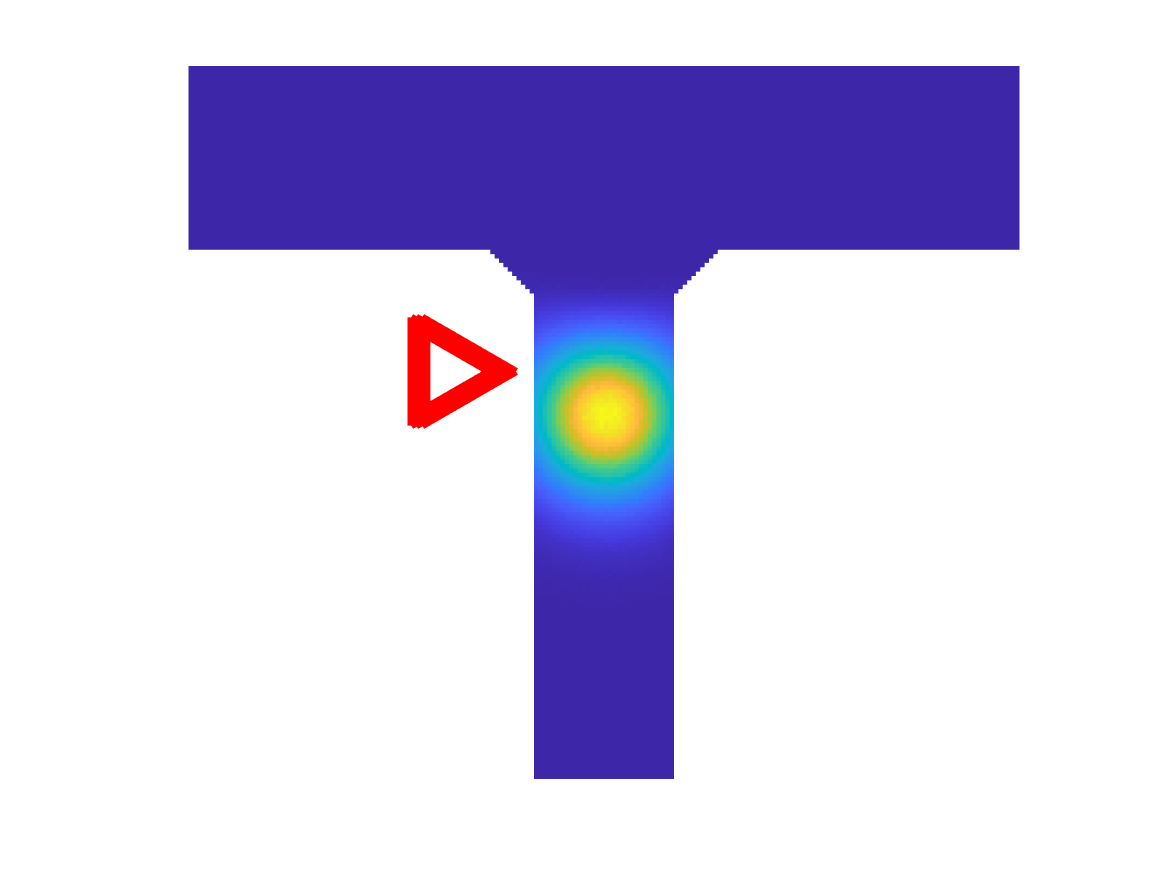

Supplement: Source code 1. [file elife-87055-code1.zip › code/fig5b_frames/92.bmp]

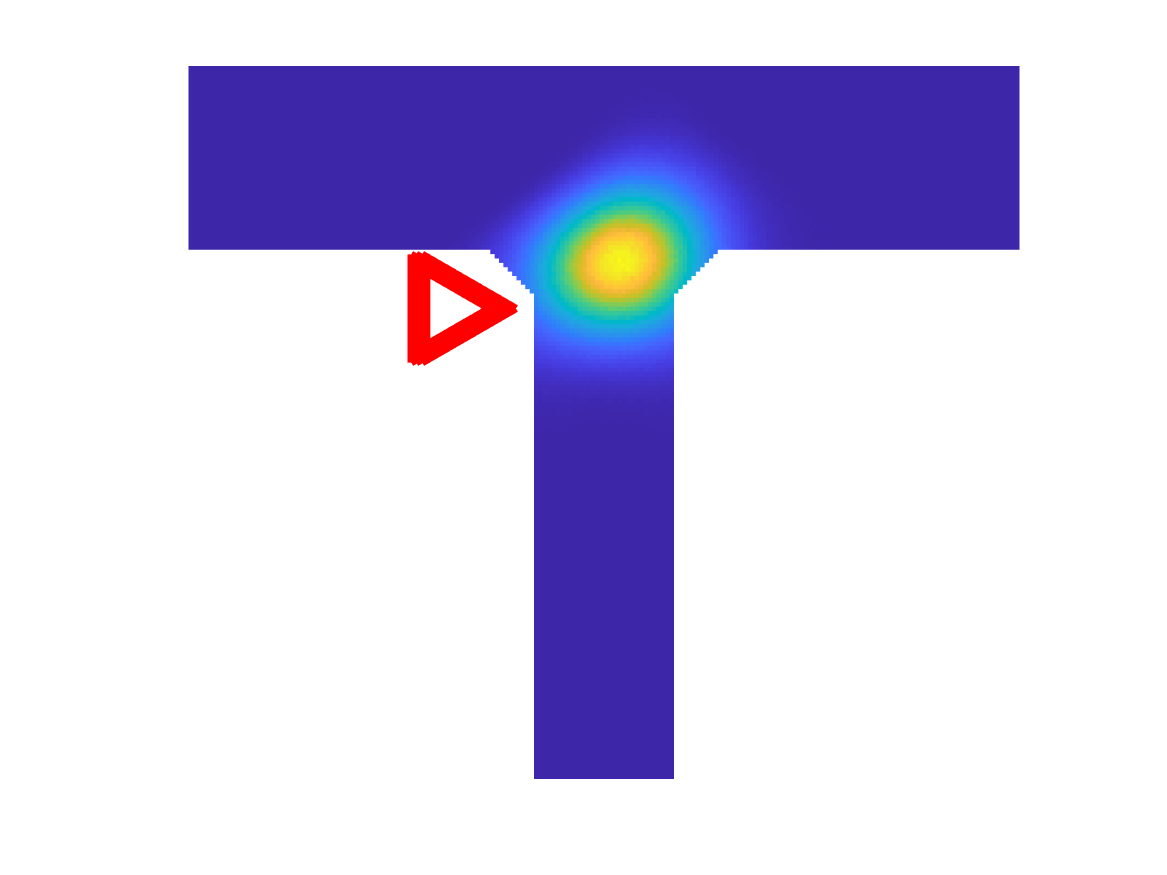

Supplement: Source code 1. [file elife-87055-code1.zip › code/fig5b_frames/187.bmp]

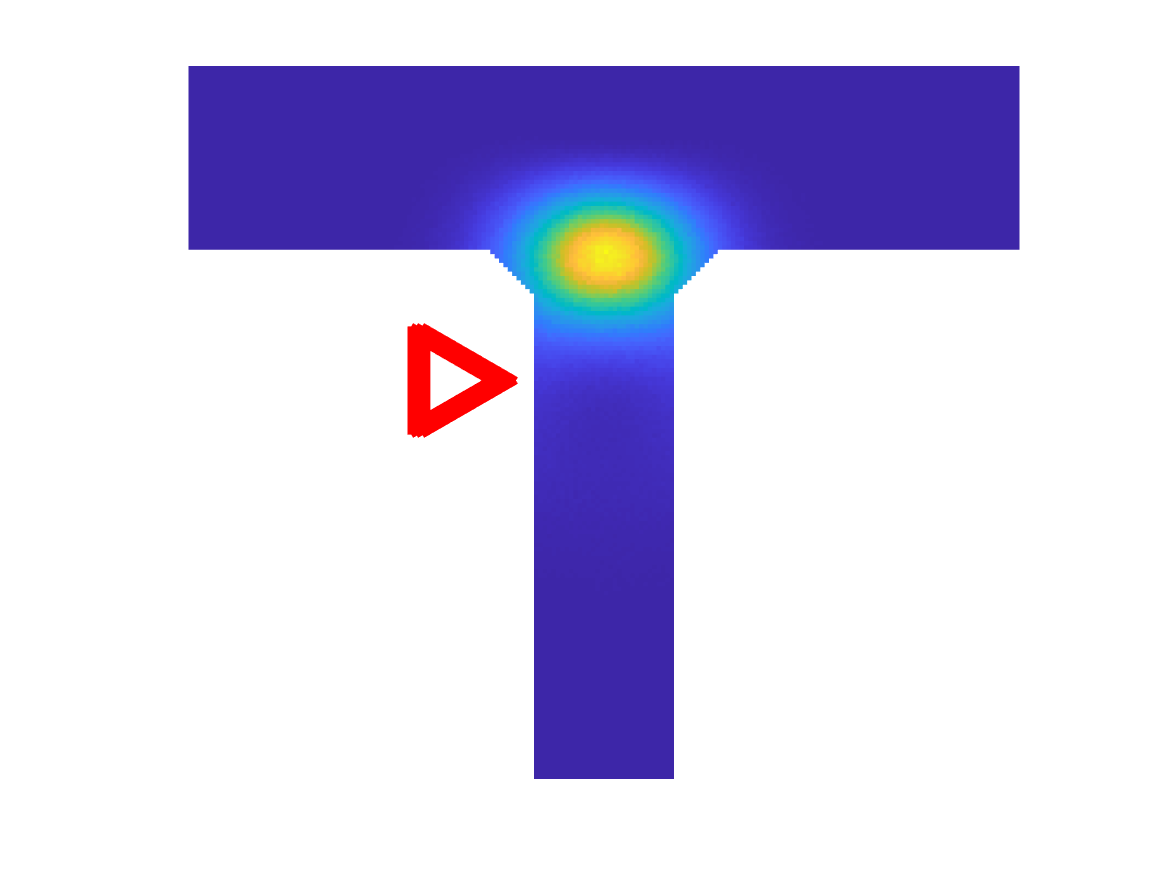

Supplement: Source code 1. [file elife-87055-code1.zip › code/fig5b_frames/79.bmp]

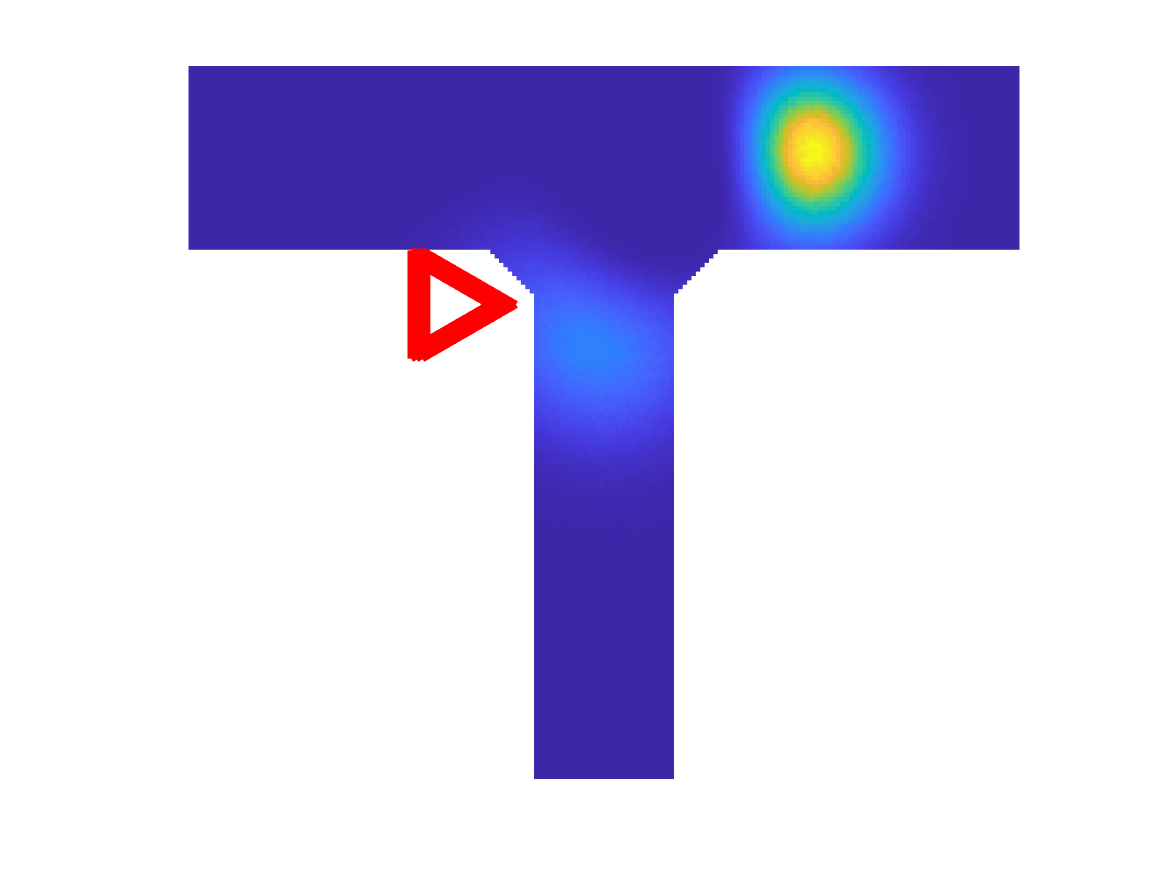

Supplement: Source code 1. [file elife-87055-code1.zip › code/fig5b_frames/193.bmp]

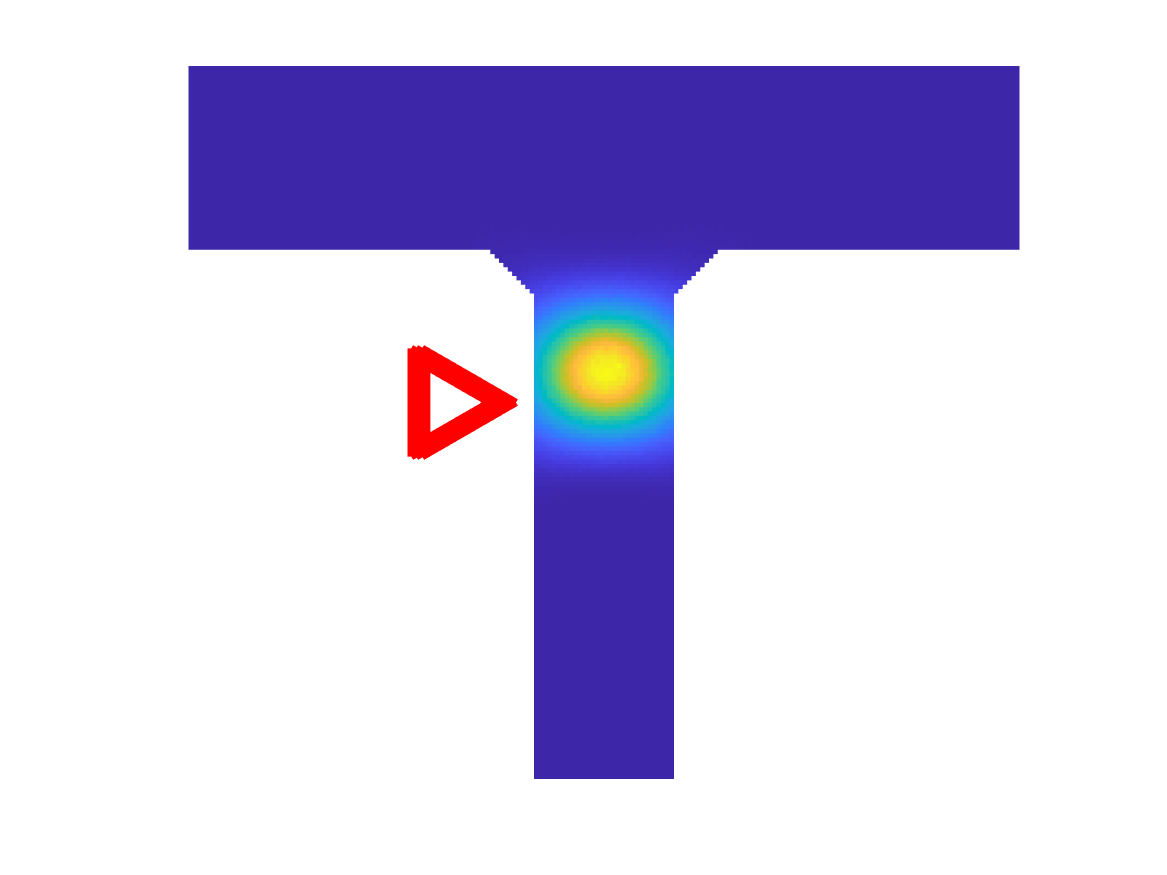

Supplement: Source code 1. [file elife-87055-code1.zip › code/fig5b_frames/45.bmp]

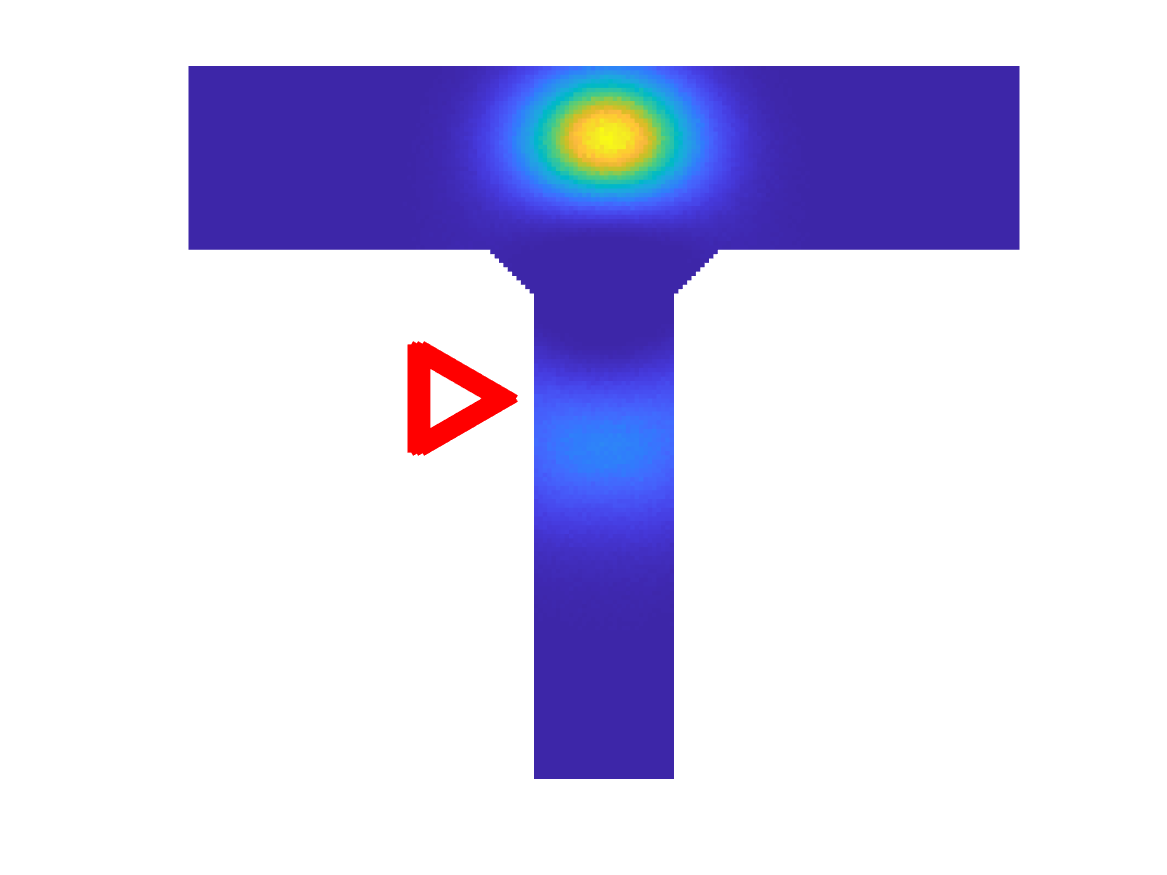

Supplement: Source code 1. [file elife-87055-code1.zip › code/fig5b_frames/51.bmp]

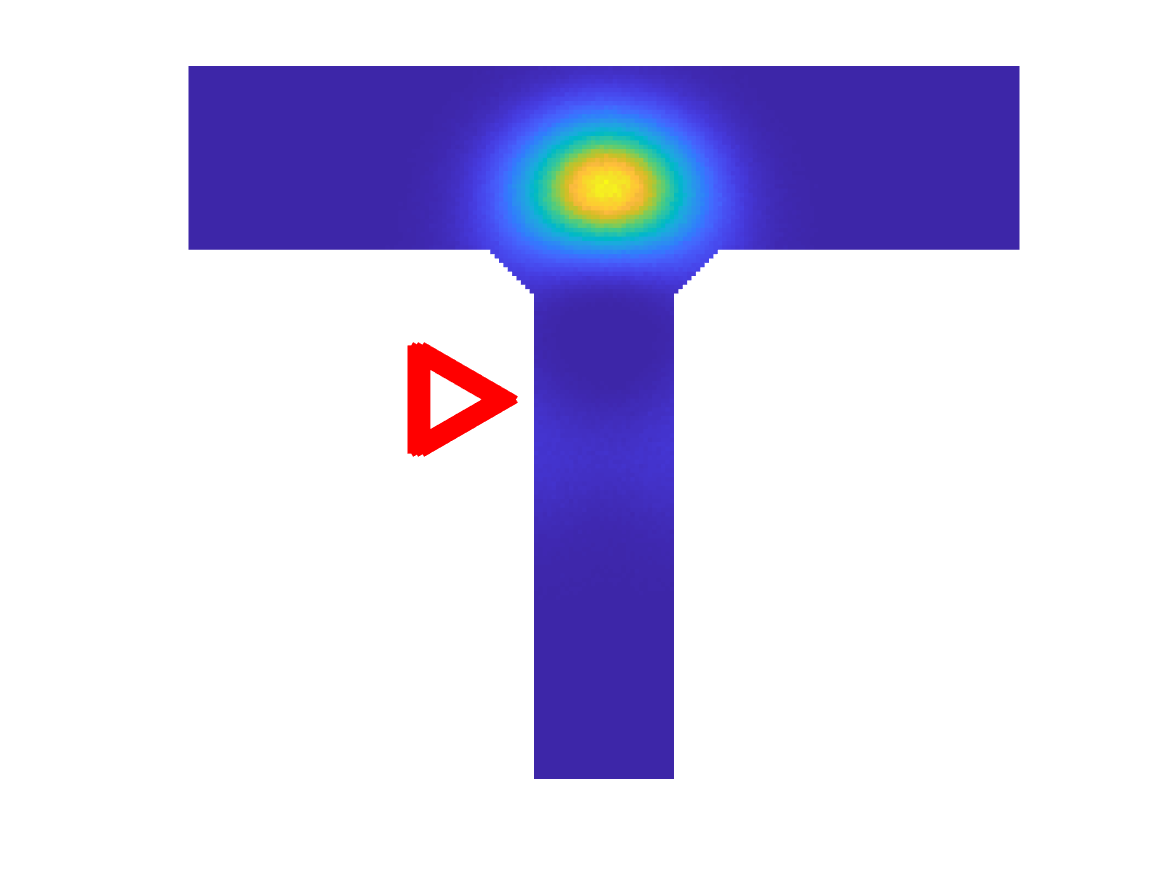

Supplement: Source code 1. [file elife-87055-code1.zip › code/fig5b_frames/50.bmp]

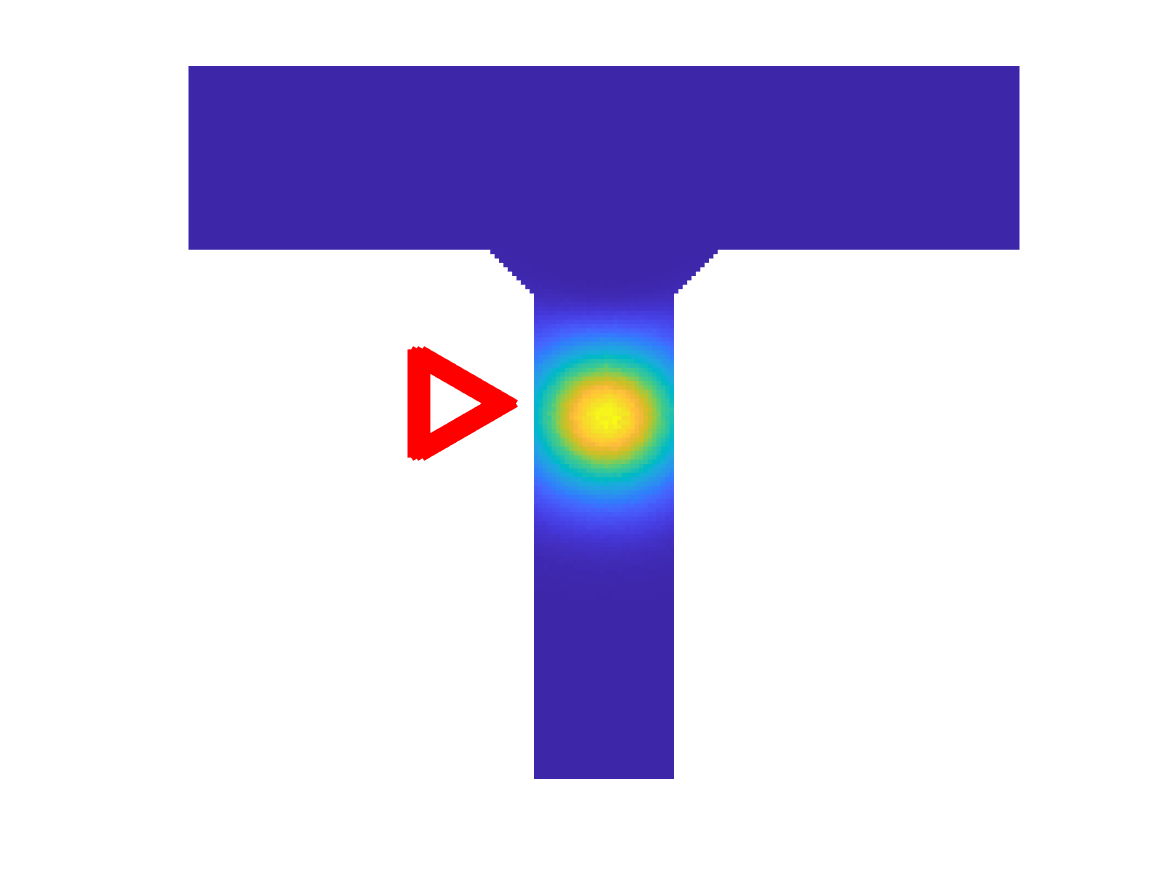

Supplement: Source code 1. [file elife-87055-code1.zip › code/fig5b_frames/44.bmp]

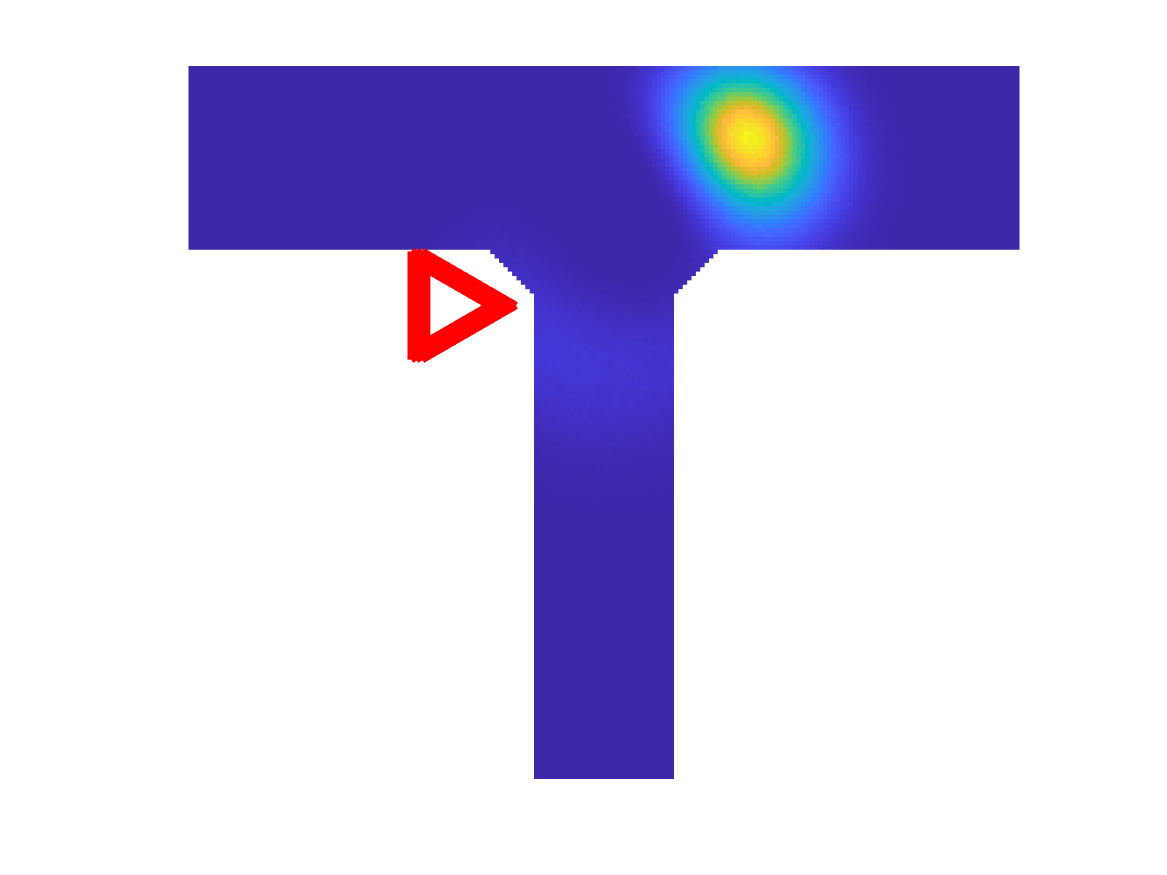

Supplement: Source code 1. [file elife-87055-code1.zip › code/fig5b_frames/192.bmp]

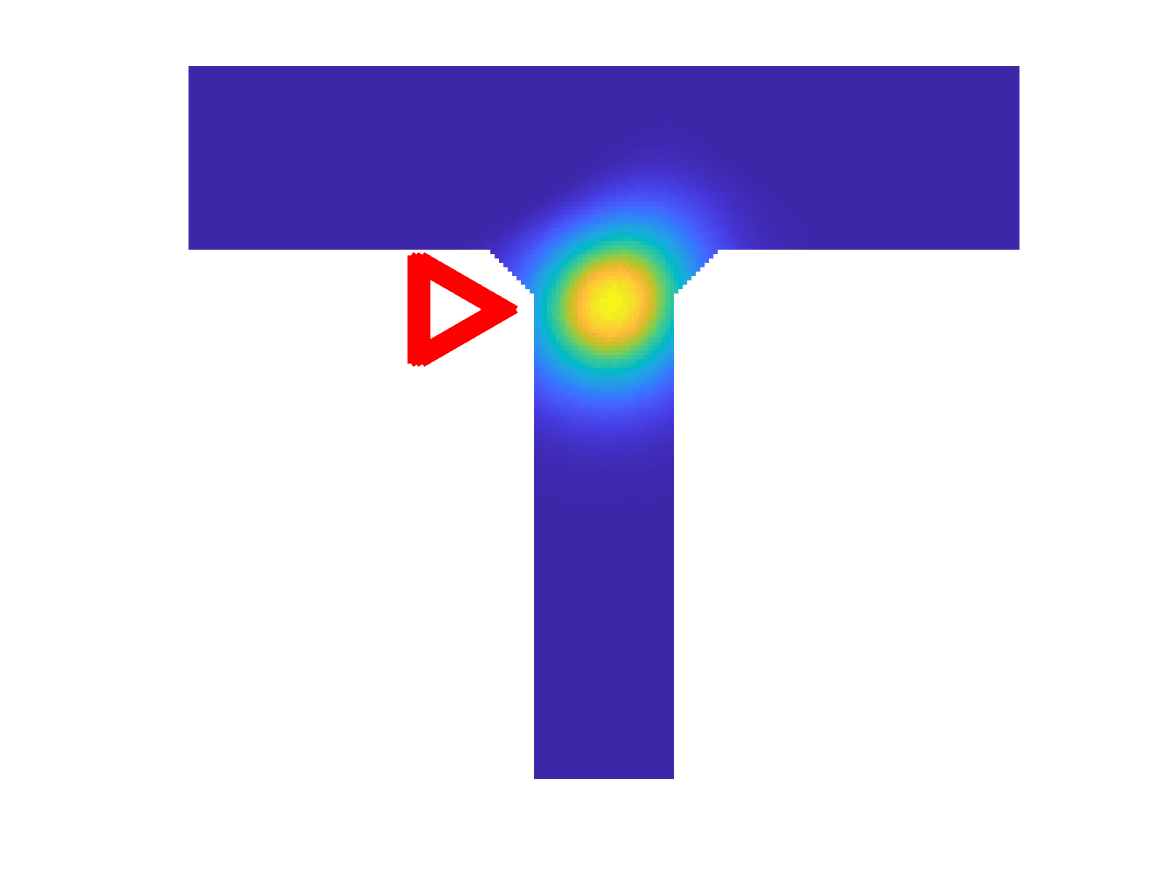

Supplement: Source code 1. [file elife-87055-code1.zip › code/fig5b_frames/186.bmp]

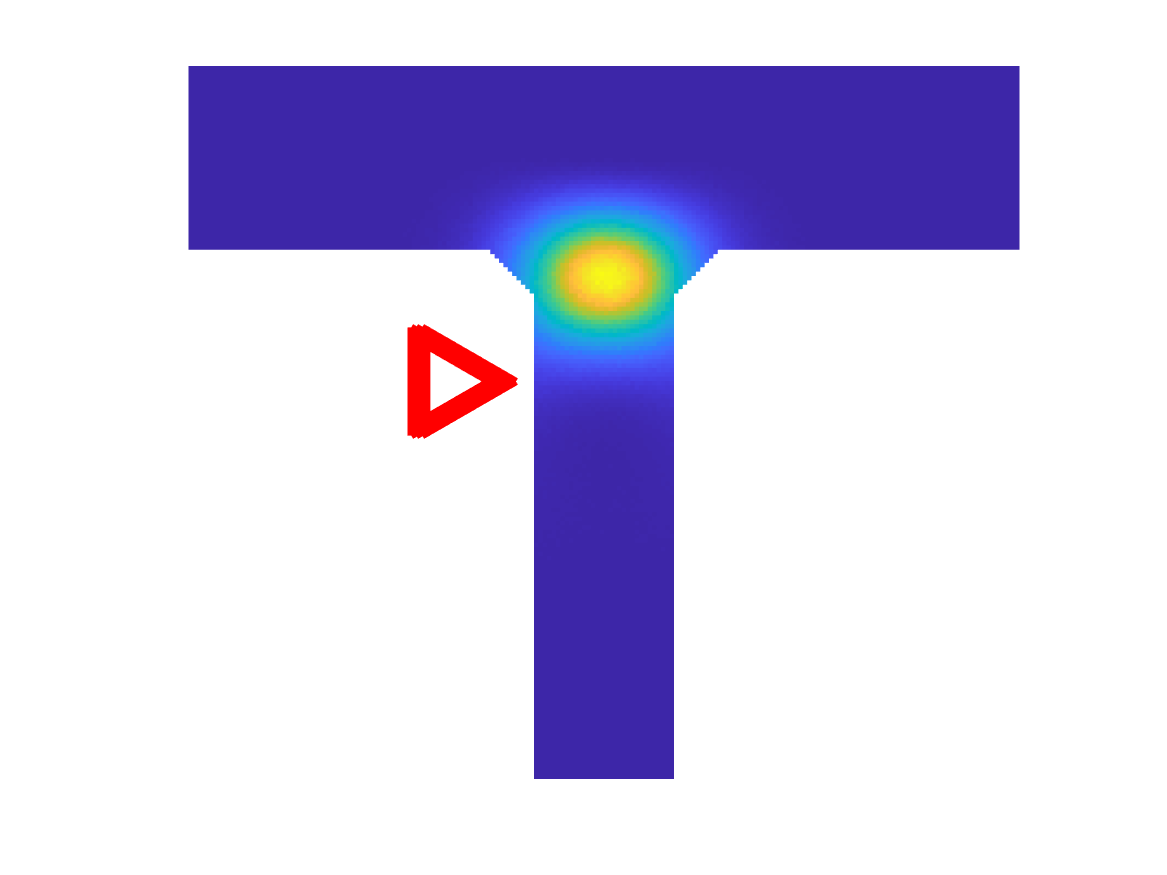

Supplement: Source code 1. [file elife-87055-code1.zip › code/fig5b_frames/78.bmp]

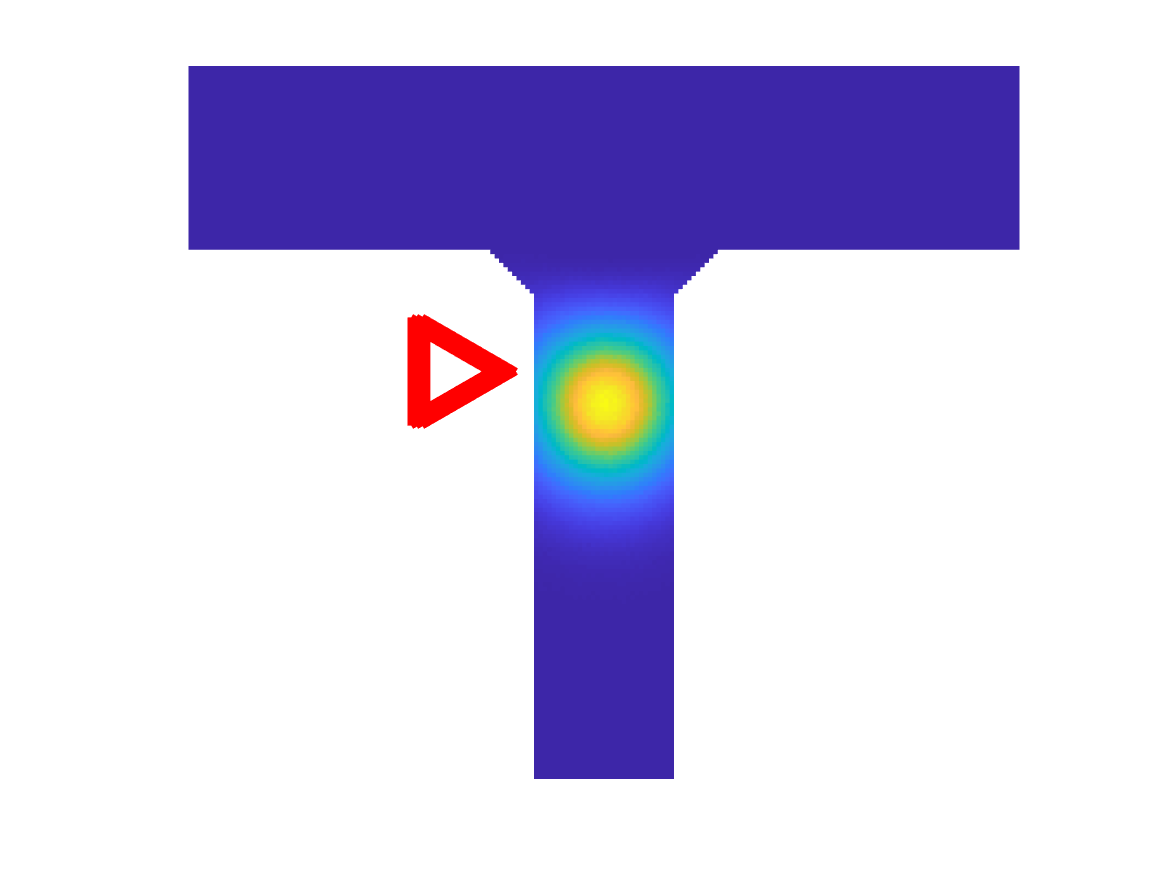

Supplement: Source code 1. [file elife-87055-code1.zip › code/fig5b_frames/93.bmp]

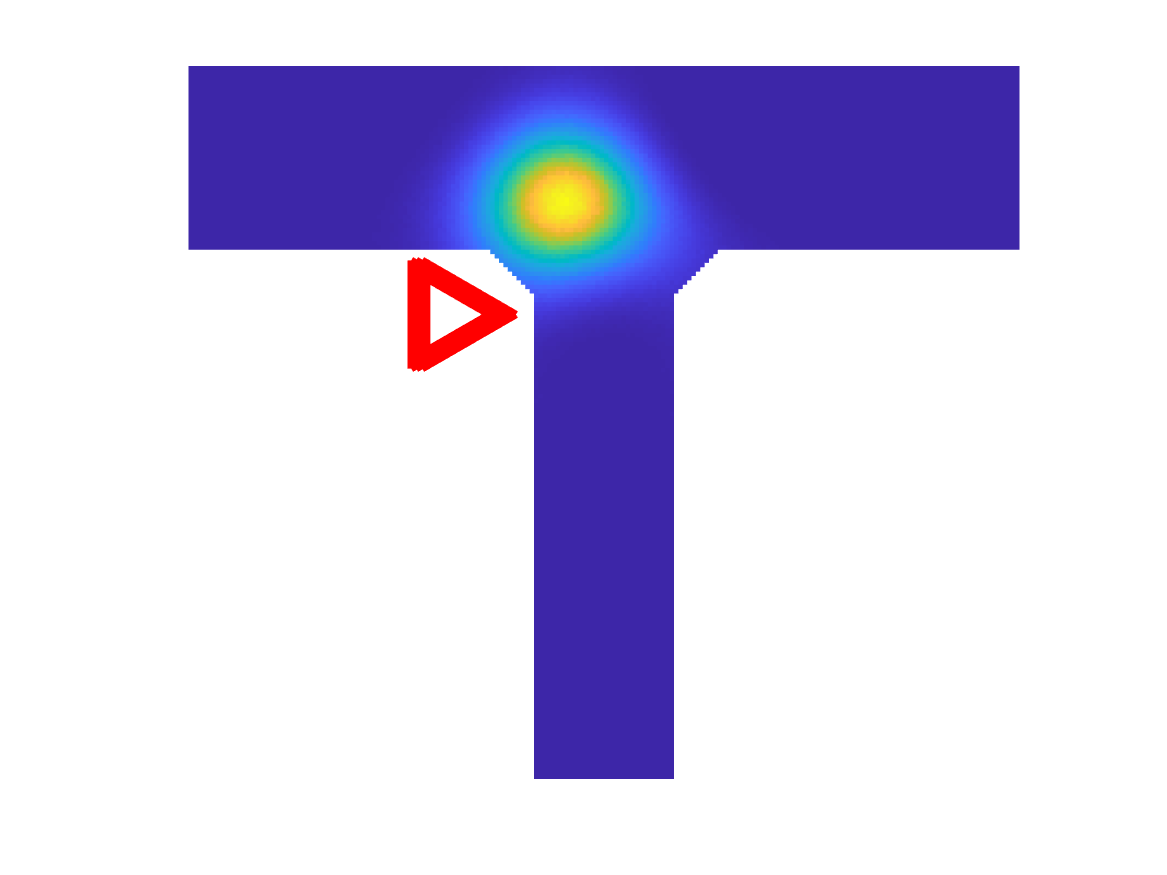

Supplement: Source code 1. [file elife-87055-code1.zip › code/fig5b_frames/179.bmp]

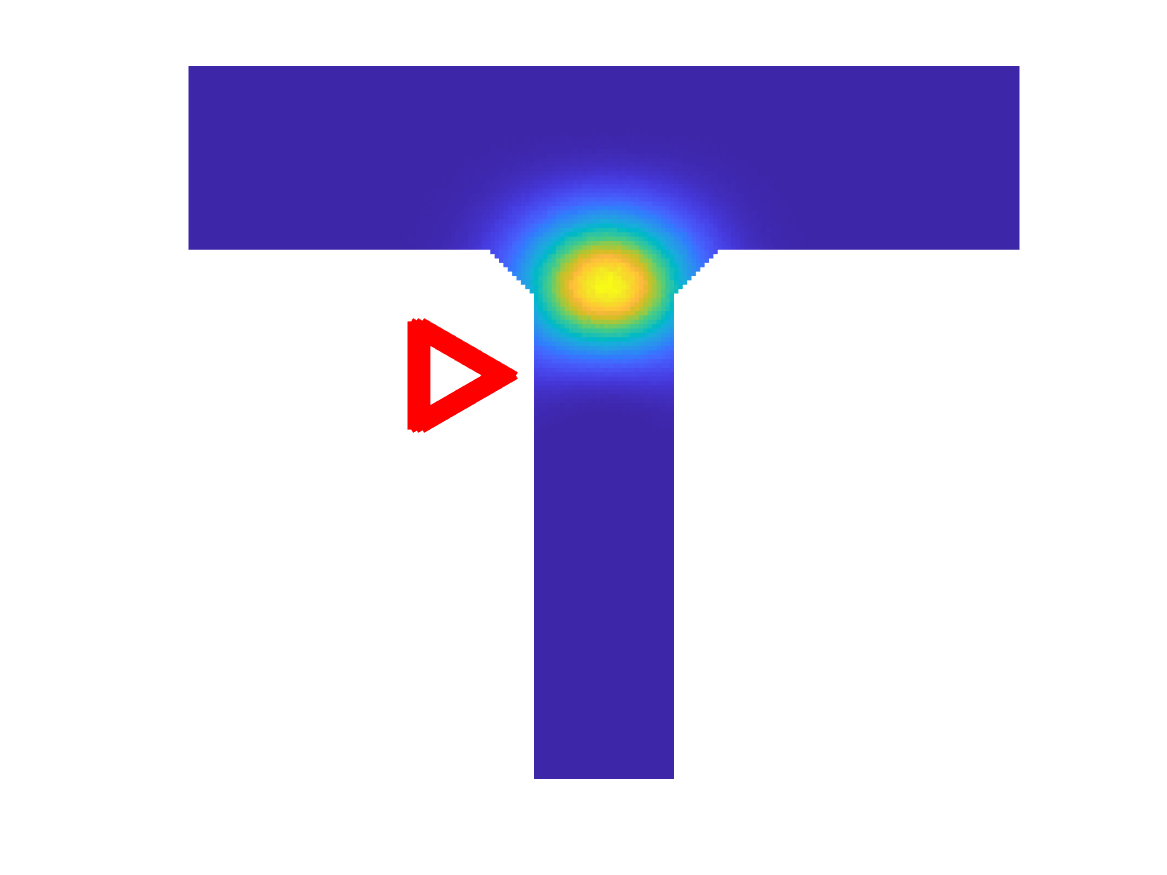

Supplement: Source code 1. [file elife-87055-code1.zip › code/fig5b_frames/87.bmp]

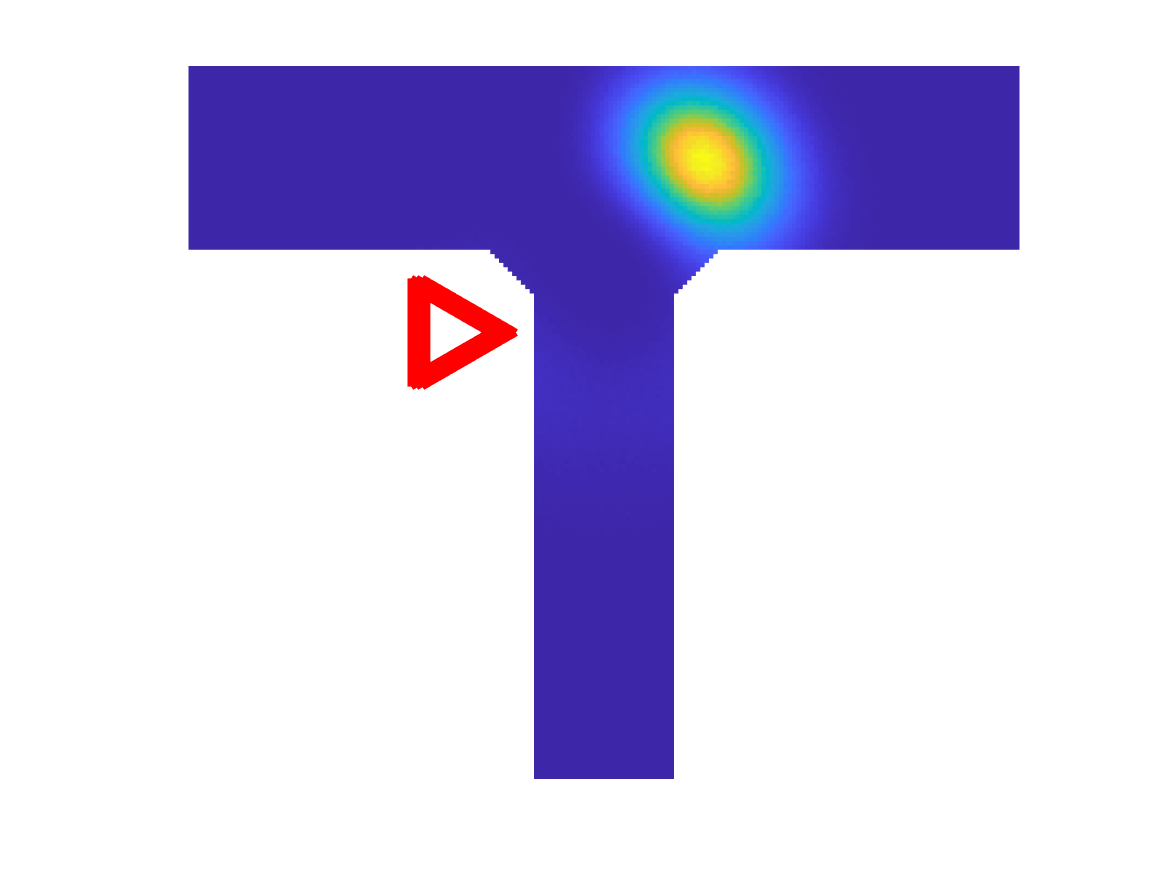

Supplement: Source code 1. [file elife-87055-code1.zip › code/fig5b_frames/151.bmp]

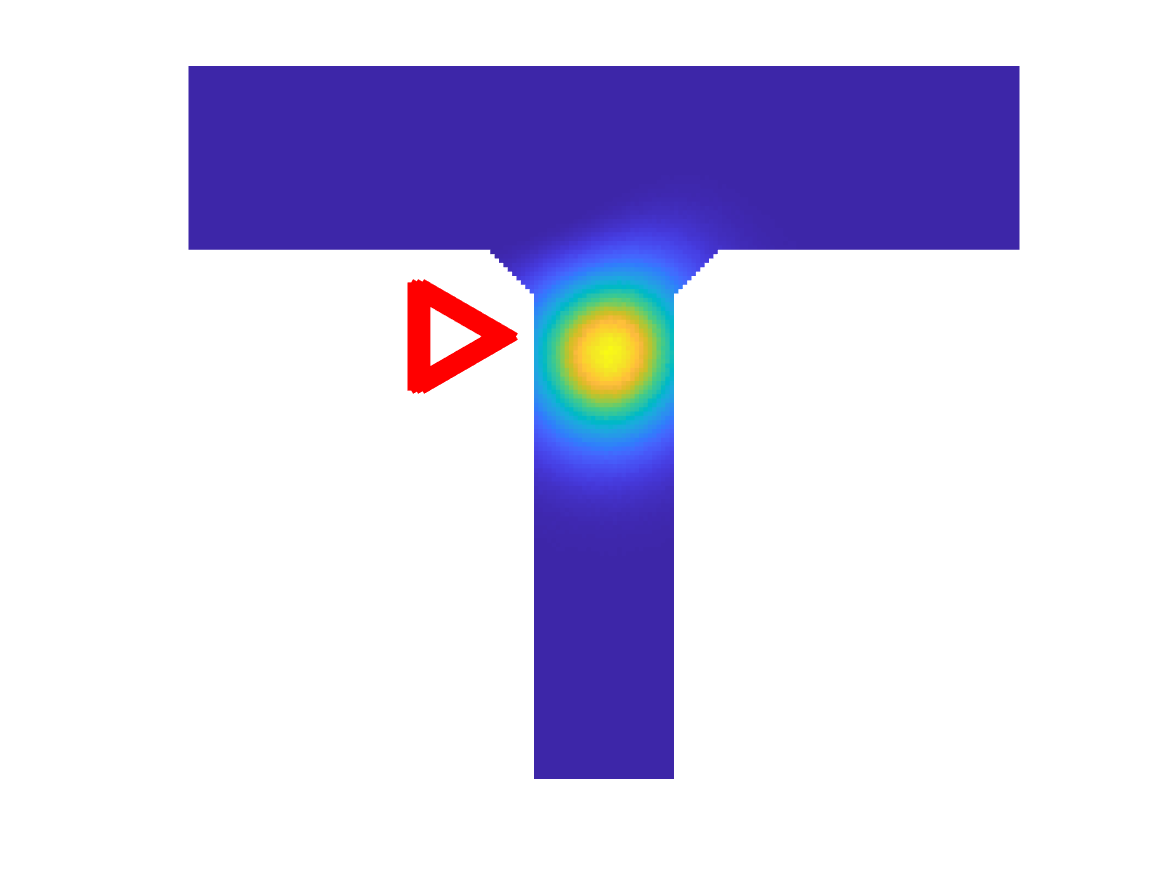

Supplement: Source code 1. [file elife-87055-code1.zip › code/fig5b_frames/145.bmp]

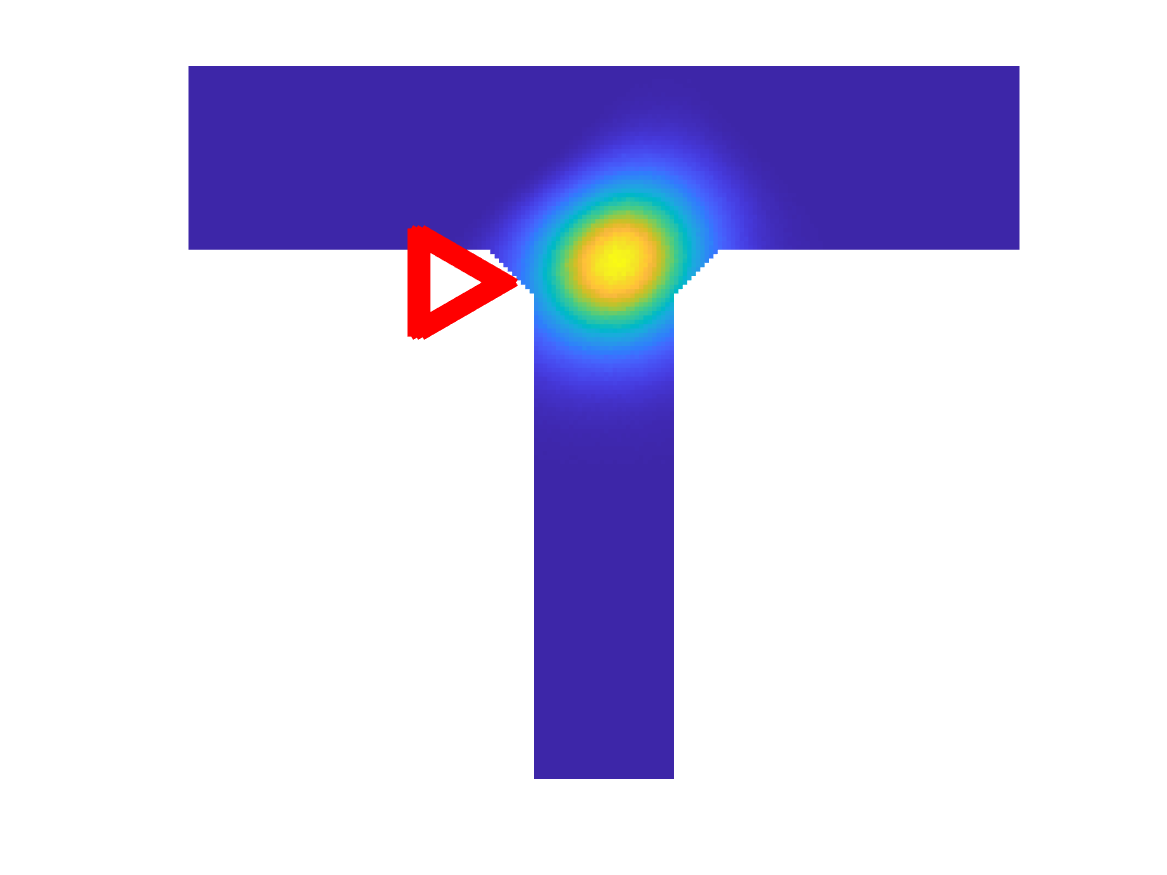

Supplement: Source code 1. [file elife-87055-code1.zip › code/fig5b_frames/227.bmp]

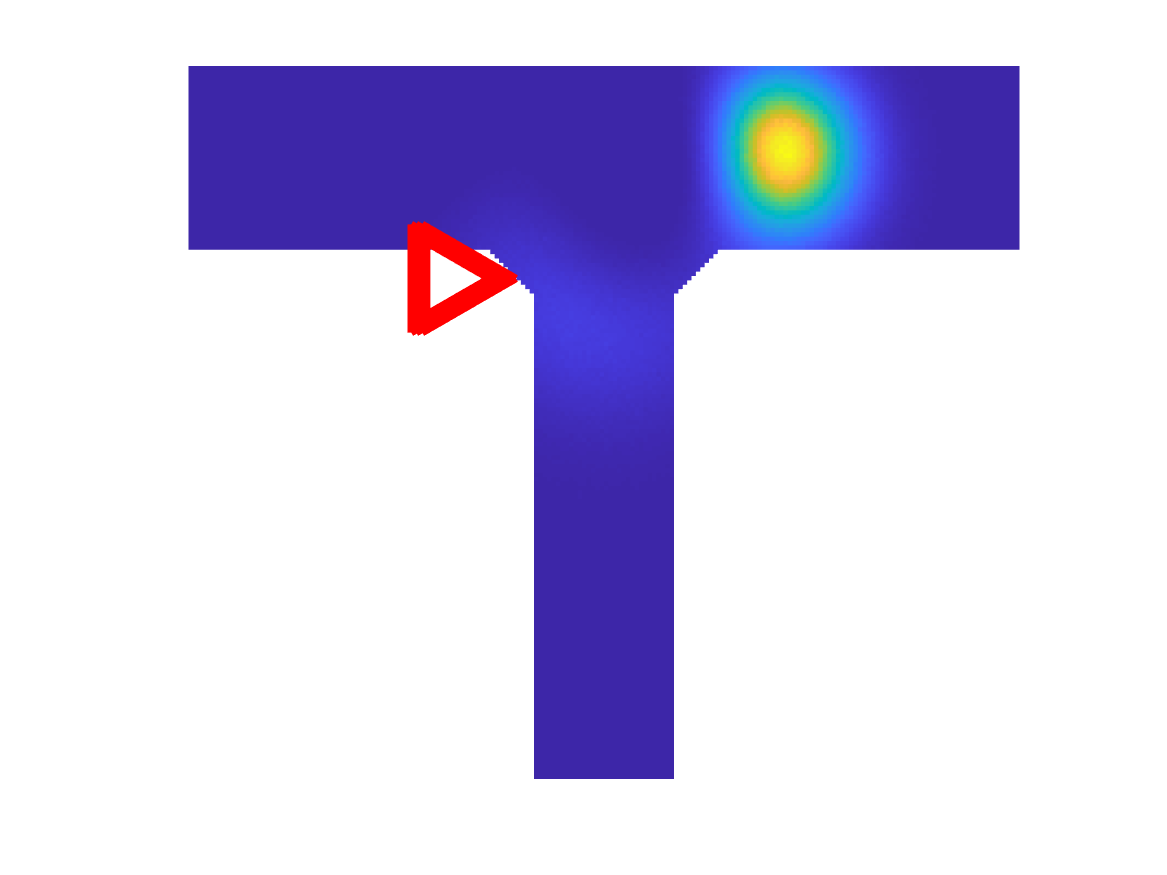

Supplement: Source code 1. [file elife-87055-code1.zip › code/fig5b_frames/233.bmp]

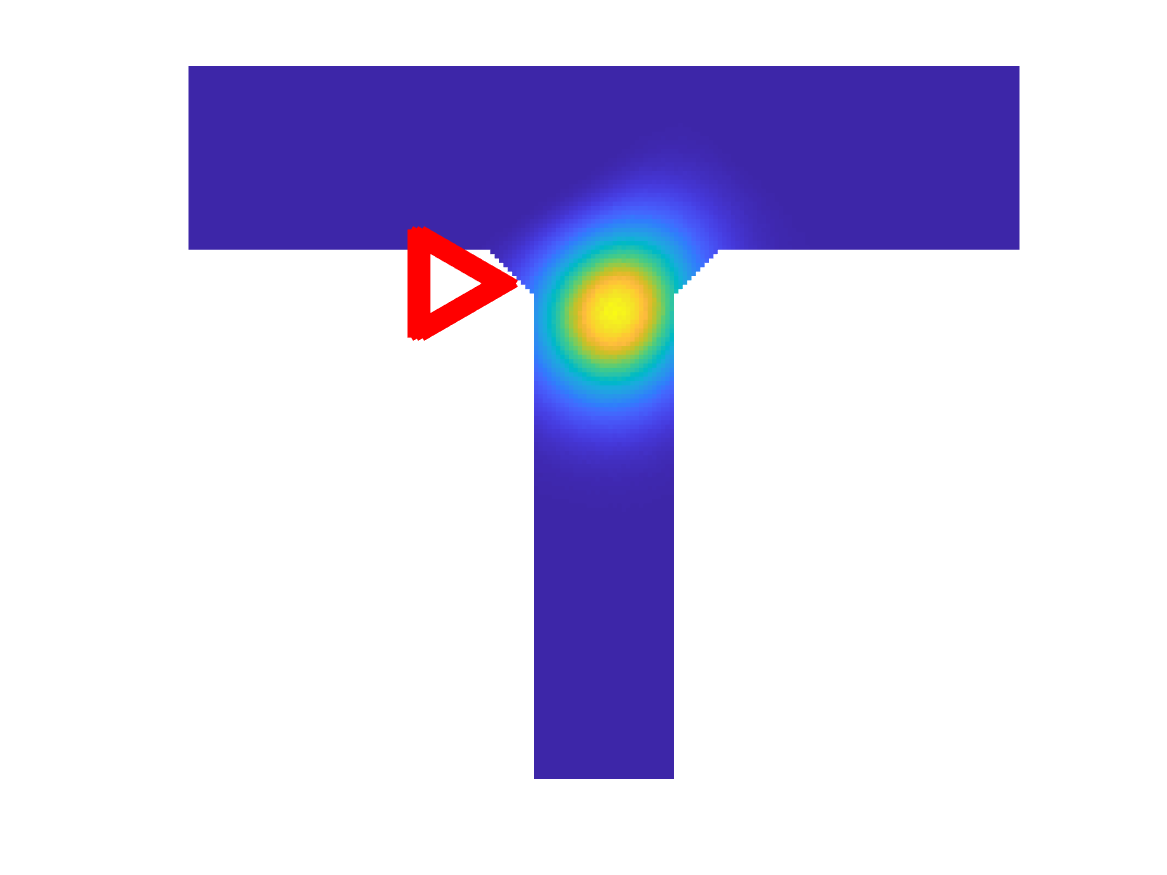

Supplement: Source code 1. [file elife-87055-code1.zip › code/fig5b_frames/225.bmp]

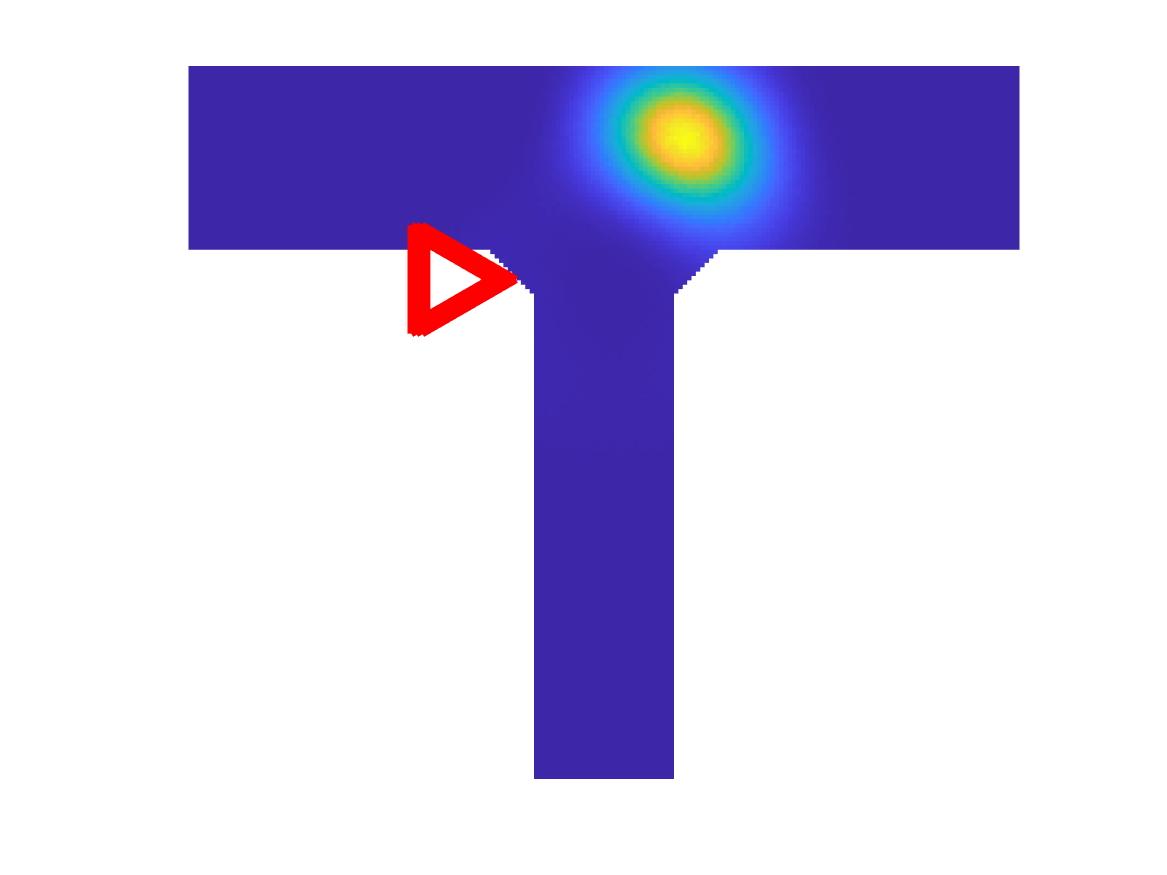

Supplement: Source code 1. [file elife-87055-code1.zip › code/fig5b_frames/231.bmp]

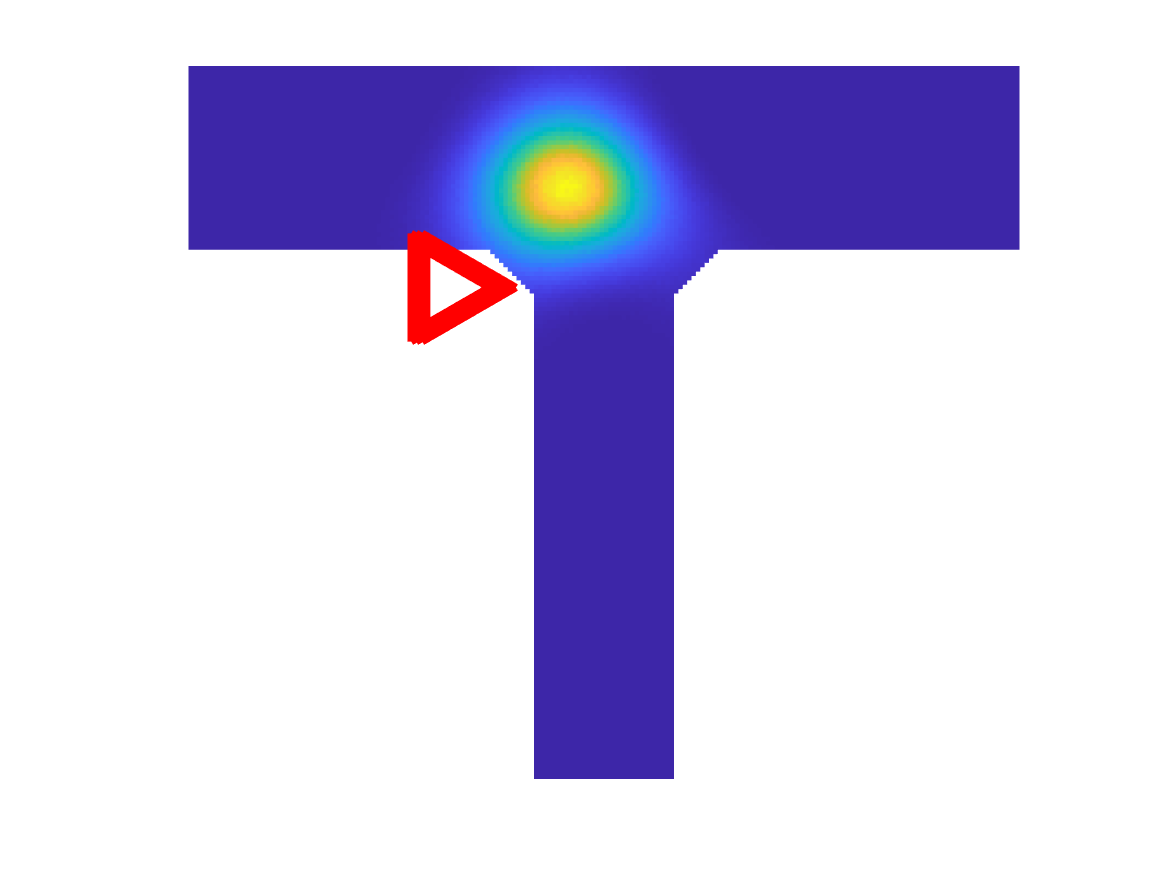

Supplement: Source code 1. [file elife-87055-code1.zip › code/fig5b_frames/219.bmp]

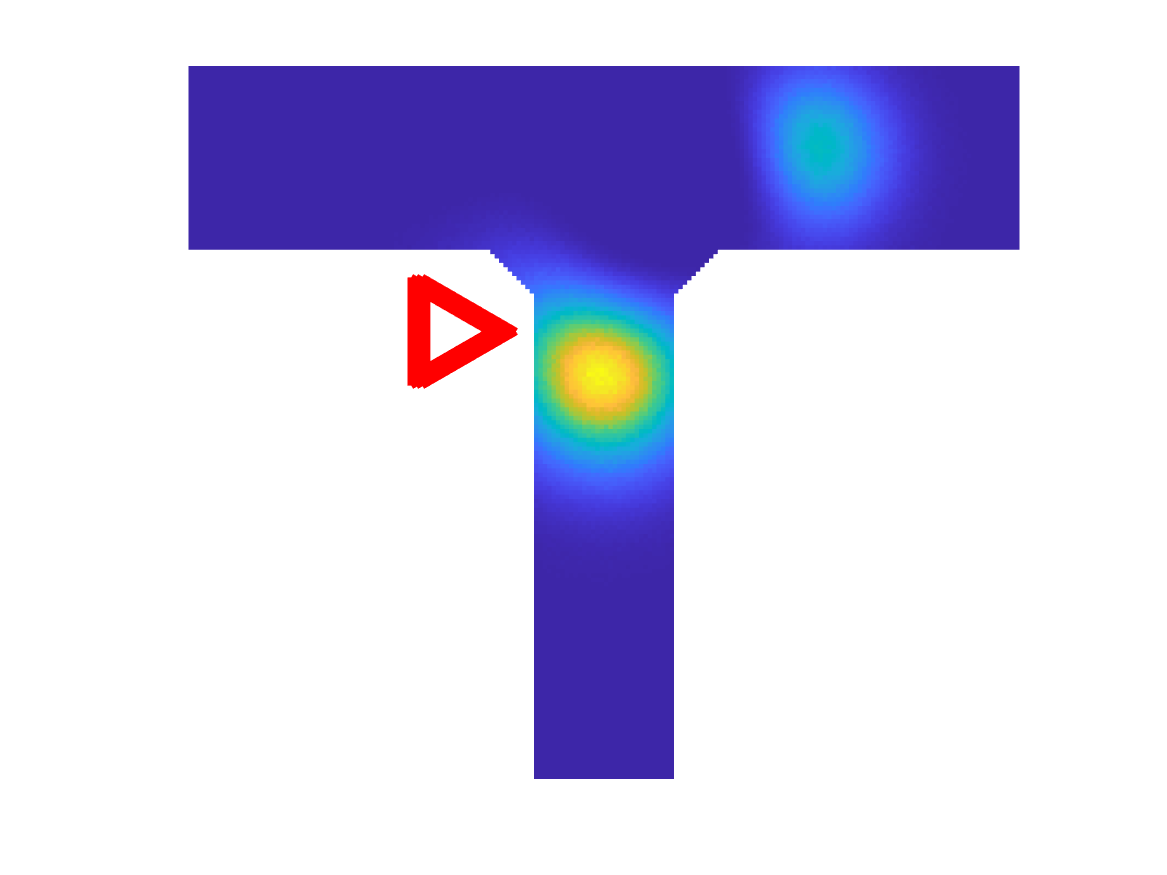

Supplement: Source code 1. [file elife-87055-code1.zip › code/fig5b_frames/153.bmp]

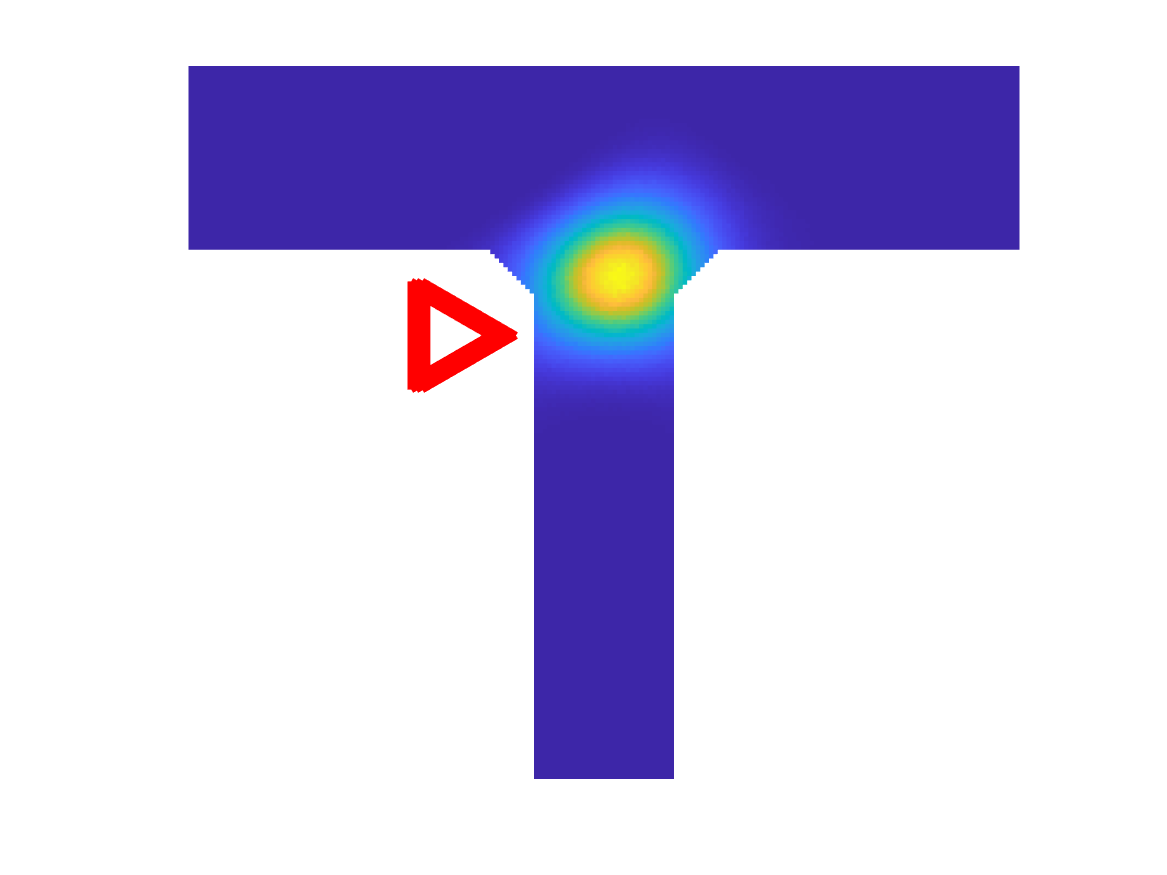

Supplement: Source code 1. [file elife-87055-code1.zip › code/fig5b_frames/147.bmp]

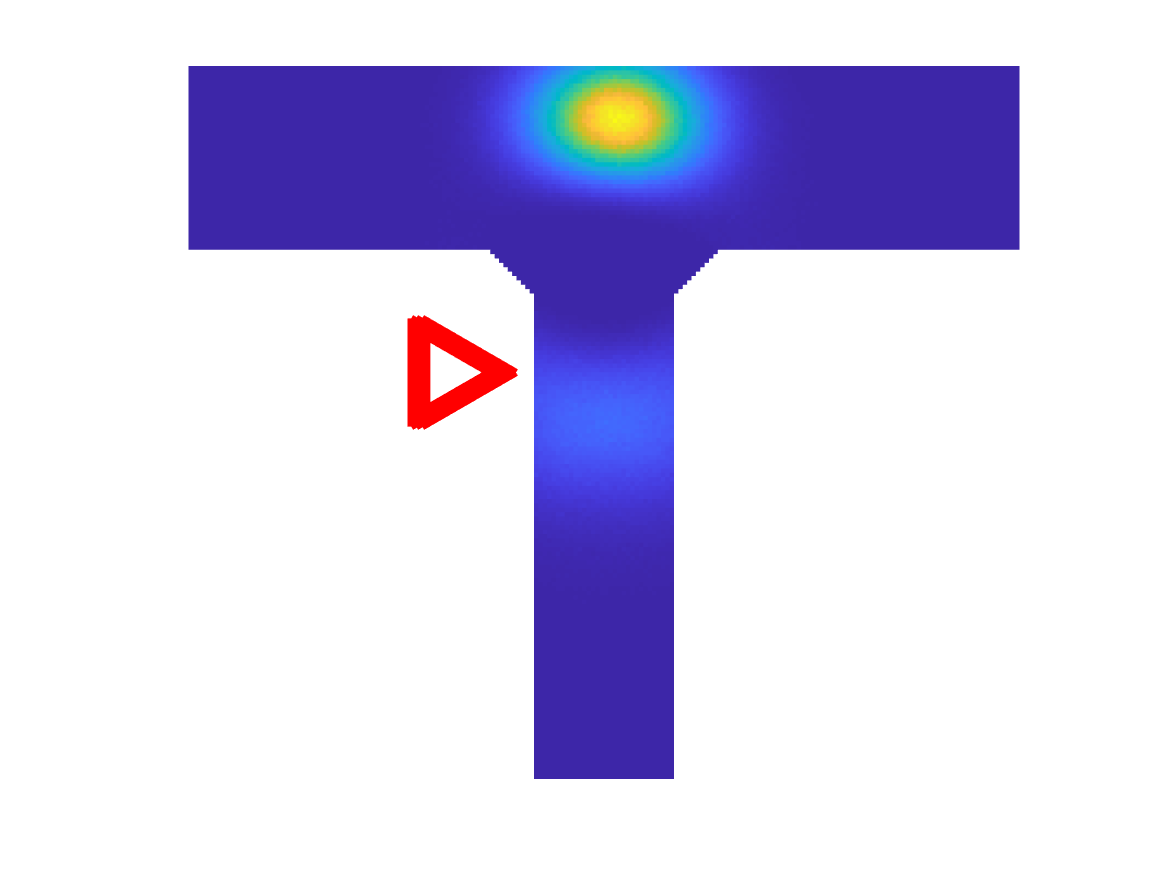

Supplement: Source code 1. [file elife-87055-code1.zip › code/fig5b_frames/91.bmp]
